# Catalytic Sulfur‐Atom Transfer Enabled by a Bis(disulfide) Titanium Complex

**DOI:** 10.1002/anie.5196140

**Published:** 2026-07-21

**Authors:** Sebastian Samir Cremer, Peter Coburger, Corinna Czernetzki, Gabriele Hierlmeier

**Affiliations:** ^1^ Institute of Inorganic Chemistry Julius‐Maximilians University of Würzburg Würzburg Germany; ^2^ Department of Inorganic Chemistry Technical University Munich Garching Germany

**Keywords:** atom‐transfer, metal–ligand cooperativity, small‐molecule activation, sulfur, titanium

## Abstract

Multi‐electron processes are key steps in the activation and valorization of small molecules. Due to titanium's electropositivity and the limited accessibility of low‐valent precursors, multi‐electron transformations and their application in atom‐transfer reactions involving this element remain comparatively underdeveloped. Herein, we report a novel four‐electron transformation from a Ti(IV) complex which enabled the formation of a rare titanium bis(disulfide) (Ti(S_2_)_2_) in a spiropentane motif. The fast and selective formation of this unique sulfur‐rich complex was leveraged for the catalytic synthesis of isothiocyanates from the corresponding isonitriles. Notably, this reaction is highly efficient even at ambient temperatures and tolerates oxygen‐containing functional groups, marking an advance over noble‐metal catalyzed methods. Detailed mechanistic studies including reaction kinetics and quantum chemical calculations provide insights into the sulfur‐atom transfer process and highlight the role of non‐covalent interactions. Overall, these results represent a new approach in the development of multi‐electron processes and atom‐transfer catalysis with group IV metals.

## Graphical abstract

Multi‐electron transformations with titanium are underexplored due to limited access to low‐valent precursors. The four‐electron activation of elemental sulfur by a Ti(IV) complex yields a rare titanium bis(disulfide) complex, enabling the efficient catalytic synthesis of isothiocyanates from isonitriles under mild conditions. Mechanistic studies reveal an outer‐sphere sulfur‐atom transfer pathway and highlight the role of non‐covalent interactions, advancing group IV atom‐transfer catalysis.

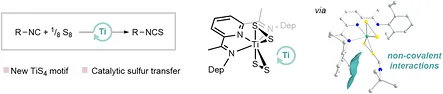

## 1 Introduction

**FIGURE 1:**
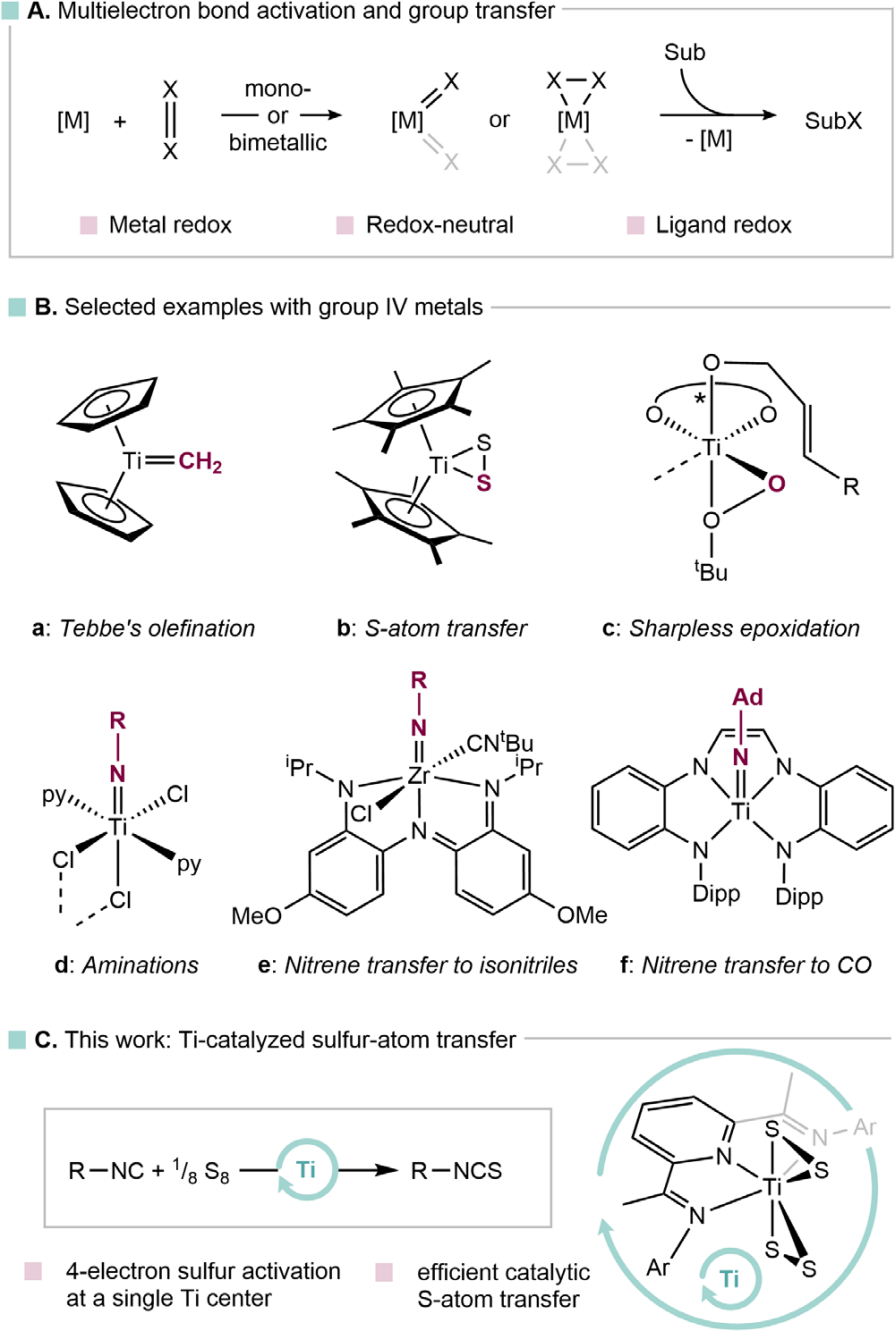
(A) Multi‐electron bond activation and group transfer. (B) Examples for stoichiometric (a and b) and catalytic (c–f) atom‐ and group‐ transfer reactivity with group IV metals. (C) This work: Catalytic S‐atom transfer by a titanium bis(disulfide) complex.

Multi‐electron processes are central to the activation of small molecules by transition metal complexes and are essential for their conversion into value‐added products through catalysis [1, 2, 3, 4]. Among these processes, atom‐ and group‐transfer reactions play a particularly important role. Examples include cyclopropanation [5], aziridination or amination [6, 7], and epoxidation reactions [8]. These typically begin with the activation of a suitable organic precursor, such as an azide or a diazo compound, by a low‐coordinate transition metal complex in either a mono‐ or bimetallic fashion. This generates a metal‐coordinated carbenoid, nitrene, or other moiety, which can then be transferred to an olefin, a C─H bond or other organic substrates (Figure 1A).

Late transition metal complexes are well‐established in nitrene transfer catalysis and usually mediate these transformations through redox changes at the metal center [6, 7]. Early transition metals, however, face inherent limitations due to their high electropositivity [9, 10, 11]. Stoichiometric atom‐ and group‐transfer reactivity with group IV metals is well documented, for example through Tebbe's reagent (Figure 1B,a) [12, 13], pnictogen transfer reactions [14, 15] or oxygen‐atom transfer (c) [16, 17, 18]. Importantly, sulfur atoms can also be transferred stoichiometrically from Cp*_2_Ti(S_2_) to phosphines as demonstrated by Bergman and Andersen (c) [19]. Nevertheless, catalytic examples remain scarce and several strategies have been explored to overcome these limitations [20]. One approach involves redox‐neutral mechanisms, in which reactions with a substrate occur without changing the metal's oxidation state. The Sharpless epoxidation (Figure 1B,c) provides a notable example of this concept [21, 22]. Redox‐non‐innocence has also been employed to facilitate nitrene transfer with titanium and zirconium. For instance, Tonks and coworkers demonstrated various aminations for the formation of, for example, substituted pyrroles [23, 24, 25], imines [26, 27], and carbodiimides [28, 29], in which substrate non‐innocence enabled reductive elimination and catalytic turnover. In contrast, Heyduk and Wolczanski utilized redox‐active supporting ligands which mediate redoxchemistry for catalytic nitrene transfer to isonitriles or carbon monoxide [30, 31, 32].

Despite these advances in nitrene‐transfer chemistry, redox‐neutral atom‐transfer reactions with titanium, apart from epoxidations, remain largely unexplored. To address this gap, we first aimed to achieve multi‐electron small molecule activation from Ti(IV) and subsequently leverage the resulting novel complexes for atom‐transfer catalysis. Building on previous work on pincer‐supported titanium complexes [33, 34], we recently demonstrated that pyridinediimine (PDI) supported titanium diorganyls undergo both radical and concerted reductive elimination reactions, enabled by metal–ligand cooperativity (MLC) [35]. We envisioned to extend this strategy to further small molecules by combination of reductive elimination and MLC, suggesting that (PDI)TiR_2_ (R = alkyl or aryl) complexes may serve as new titanium(0) synthons [36].

Herein, we report that (^Et^PDI)Ti(CH_2_Ph)_2_ enables a four‐electron activation of elemental sulfur to access a novel sulfur‐rich TiS_4_ motif, which was previously inaccessible by direct S_8_ activation with titanium. This transformation was subsequently leveraged to develop a titanium‐catalyzed synthesis of isothiocyanates from isonitriles under mild reaction conditions with broad functional group tolerance. Detailed experimental and quantum chemical studies show that S‐atom transfer proceeds through a redox‐neutral outer‐sphere mechanism enabled by non‐covalent substrate‐catalyst interactions.

## 2 Results and Discussions

### 2.1 Stoichiometric Sulfur Activation

**FIGURE 2:**
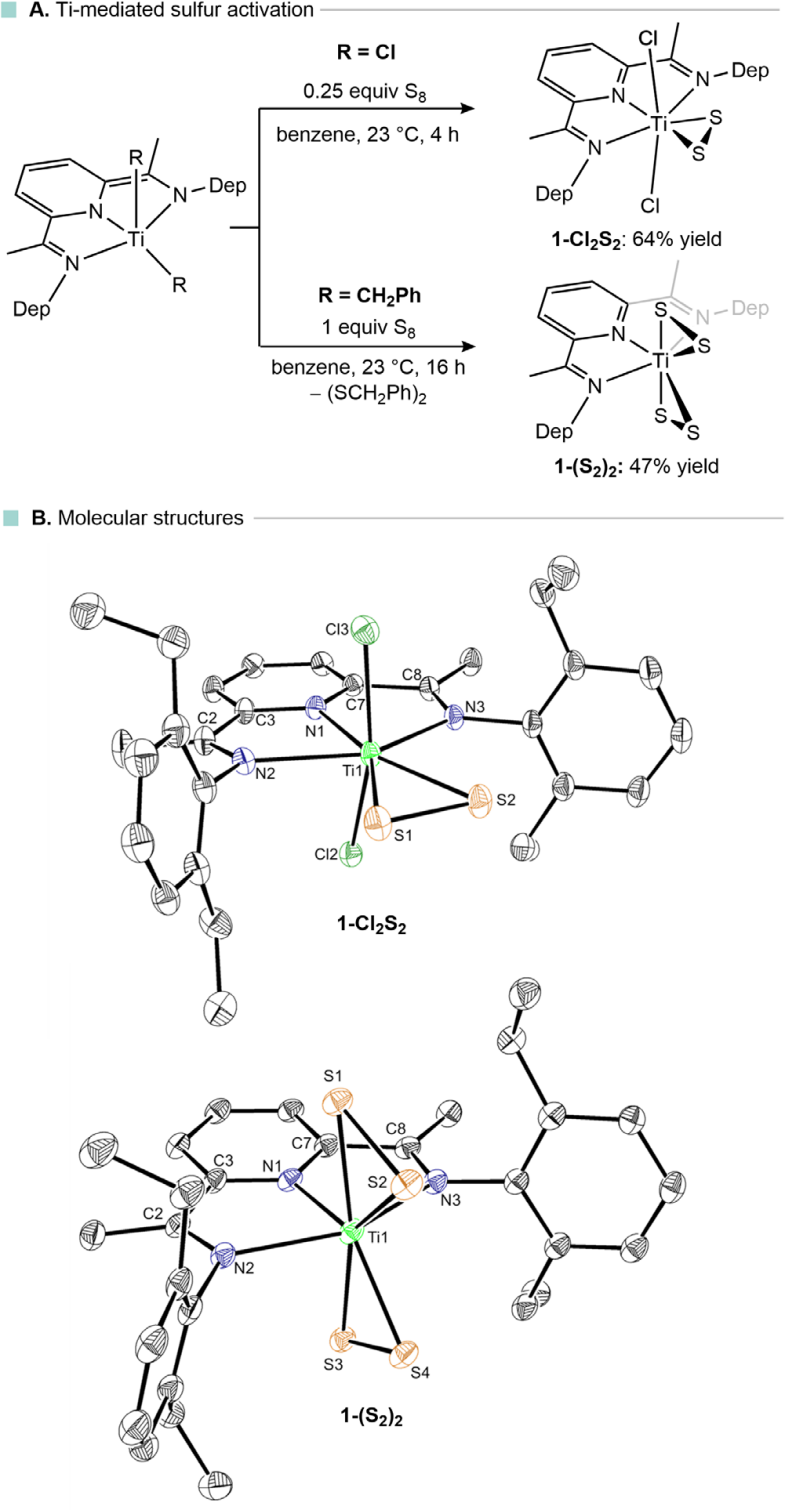
(A) Synthesis of (^Et^PDI)TiCl_2_S_2_ (**1‐Cl_2_S_2_**) and (^Et^PDI)Ti(S_2_)_2_ (**1‐(S_2_)_2_**) via activation of elemental sulfur. (B) Molecular structures of **1‐Cl_2_S_2_** and **1‐(S_2_)_2_** in the solid state with 30% probability ellipsoids, H atoms are omitted for clarity [40].

Before investigating the targeted four‐electron transformation from Ti(IV) with PDI complexes, the ability of this metal–ligand combination to mediate two‐electron transformations via metal–ligand cooperativity was examined. For this purpose, the titanium dichloride complex (^Et^PDI)TiCl_2_ (**1‐Cl_2_**, ^Et^PDI = (2,6‐Et_2_‐C_6_H_3_N═CMe)_2_C_5_H_3_N) was investigated [37]. Treatment of a benzene solution of **1‐Cl_2_** with 0.25 equiv of S_8_ resulted in the immediate formation of a leaf green precipitate. The corresponding disulfide complex **1‐Cl_2_S_2_** was obtained in 64% yield (Figure 2A). Single crystals suitable for X‐ray diffraction were obtained, confirming the molecular structure in the solid state and revealing an *η* ^2^‐(S_2_)^2−^‐ligand (Figure 2B). The Ti1─S1 (2.3228(7) Å), Ti1─S2 (2.3233(7) Å) and S1─S2 (2.0346(10) Å) bond lengths are consistent with those reported for related titanium(IV) disulfide complexes with S_2_‐moieties (e.g. (κ^2^­Tp)(κ^3^­Tp)Ti(S_2_): Ti−S: 2.3125(12) Å and 2.3365(12) Å; S−S: 2.0099(14) Å; Tp = hydrotris(pyrazol‐1‐yl)borate) [38]. Consistent with this assignment, the Δ‐value of the PDI ligand in **1‐Cl_2_S_2_** (0.17 Å) indicates a neutral PDI ligand in combination with Ti(IV), supporting MLC in the two‐electron activation of elemental sulfur [39].

Having established that MLC is accessible for this metal–ligand platform, the alkyl‐substituted PDI complex **1‐(CH_2_Ph)_2_** was next examined for sulfur activation (Figure 2A). Addition of one equivalent of S_8_ to a benzene solution of this complex led to the precipitation of a dark‐green solid. Single crystals suitable for X‐ray diffraction revealed the formation of a titanium bis(persulfide) complex **1‐(S_2_)_2_** featuring a spiropentane‐type motif (Figure 2B). Complex **1‐(S_2_)_2_** was isolated by filtration in 47% yield. Analysis of the reaction supernatant via GC‐MS revealed the formation of dibenzyldisulfide as the major organic product, accompanied by minor amounts of bibenzyl. These observations are consistent with MLC‐assisted sulfide formation at titanium, sulfur insertion into the Ti─C bonds and subsequent reductive elimination of dibenzyldisulfide, which is in agreement with isolated intermediates in chalcogen activation by Mindiola and coworkers [41, 42]. Alternatively, Ti─C or Ti─S bond homolysis pathways might be operative [43].

To the best of our knowledge, structurally verified (*η* ^2^‐S_2_)_2_Ti motifs have been reported only once by the Rauchfuss group, who isolated such titanium complexes from a binary titanium sulfide [TiS_∼9_, obtained from TiCl_4_ and ZnS_6_(TMEDA)] using Lewis bases such as 1‐methylimidazole or pyridine [44]. In contrast, the approach reported herein employs well‐defined titanium complexes and, importantly, uses elemental sulfur as a sulfur source, which is essential for its functionalization (*vide infra*). Furthermore, the formation of **1‐(S_2_)_2_** demonstrates that the combination of two two‐electron pathways, namely reductive elimination of labile ligands and redox‐active ligand participation, can facilitate a rare four‐electron transformation from a Ti(IV) complex in a single reaction. Notably, Fortier and coworkers demonstrated six‐ and even eight‐electron transformations with titanium in a bimetallic framework [45]. Analysis of the bond metric data reveals that, analogous to **1‐Cl_2_(S_2_)**, the Δ‐value for **1‐(S_2_)_2_** (0.16 Å) is consistent with a neutral PDI ligand, supporting metal–ligand cooperativity in the sulfur activation process [46, 47]. The Ti1─S1 and Ti1─S3 bonds [2.4043(10) Å, 2.4000(10) Å)] are elongated when compared to the respective Ti1─S2 and Ti1─S4 bonds [2.3301(10) Å, 2.3137(9) Å]. Nevertheless, these bond lengths are comparable to those reported by Rauchfuss and coworkers (2.4055(9) Å, 2.428(1) Å; 2.3484(9) Å, 2.3635(9) Å) [48]. Notably, (*η* ^2^‐S_2_)_2_M motifs are generally scarce, with structurally verified examples outside Rauchfuss’ work found only in the neighboring groups V [49, 50, 51] and VI [52, 53, 54].

**FIGURE 3:**
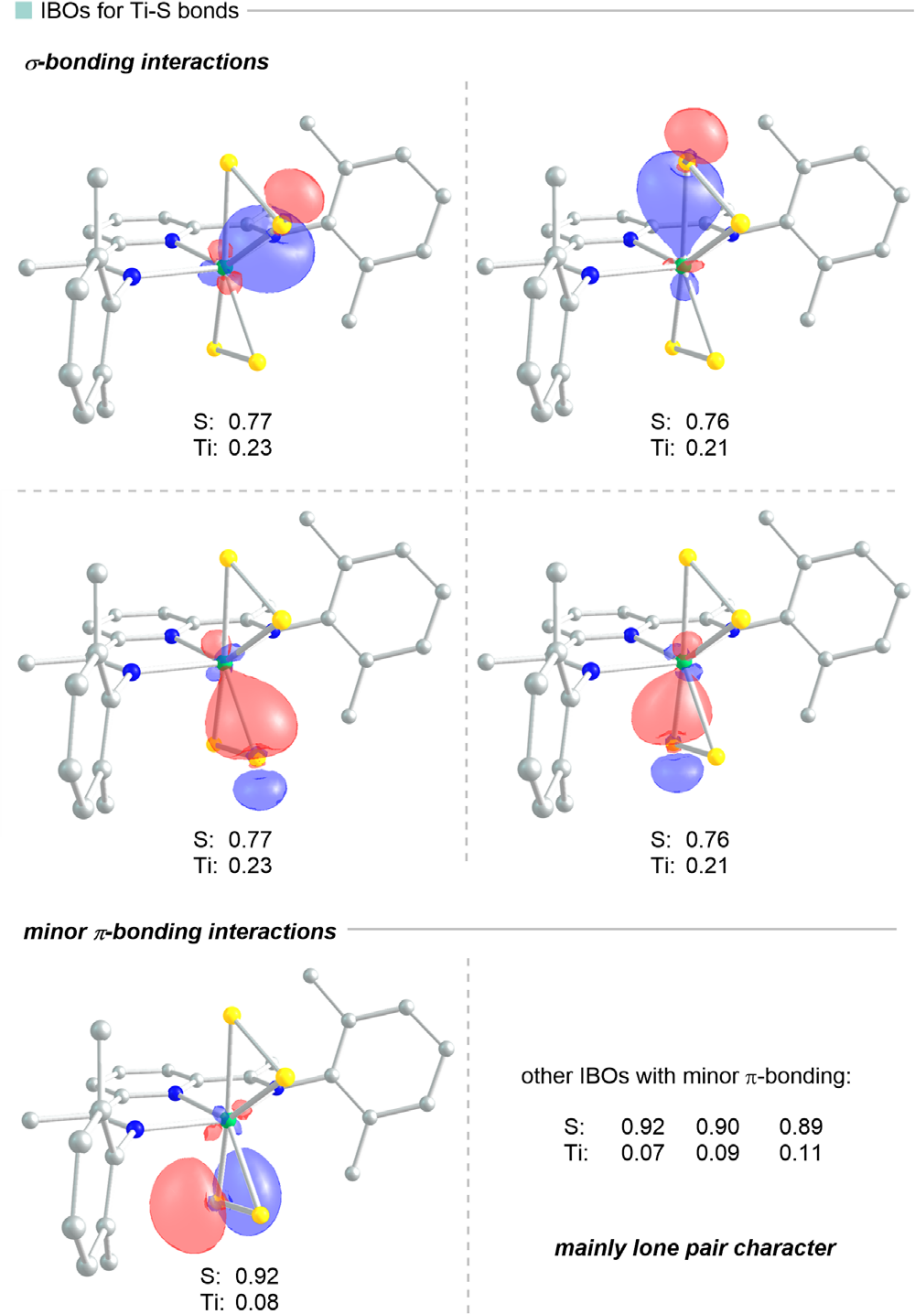
Analysis of TiS_4_ bonding in (^Me^PDI)Ti(S_2_)_2_ by DFT with IBOs; isocontour value for all orbitals with σ‐interactions is 0.06, for those with (minor) π‐interactions 0.08; IBO = intrinsic bond orbitals.

Having isolated this unusual and symmetric TiS_4_ complex, its Ti─S bonding was analyzed by quantum chemical calculations for the truncated model complex (^Me^PDI)Ti(S_2_)_2_ (**1’‐(S_2_)_2_**). DFT calculations at the PBE0/def2‐TZVP level for **1’‐(S_2_)_2_** reproduce the solid‐state molecular structure of **1‐(S_2_)_2_**, with calculated Ti─S bond length of 2.39, 2.39, 2.30, and 2.31 Å. Mayer bond orders of 0.89, 0.96, 0.97, and 1.03 are consistent with the presence of four Ti─S bonds. Analysis of the Ti─S bonding by intrinsic bond orbitals (IBOs) reveals interactions between titanium‐centered orbitals and p‐type lone pairs of the sulfur units, giving rise to four σ‐ bonds (Figure 3, top) and four additional interactions with minor π‐bonding character which are predominantly localized on sulfur (Figure 3, bottom). The highest occupied molecular orbitals in **1’‐(S_2_)_2_** can be rationalized with symmetry‐adapted linear combinations (SALCs) of the overall four (S_2_)^2−^ π* orbitals and their (partial) interaction with titanium‐centered orbitals (see Figures S49 and S50).

### 2.2 Catalytic Sulfur‐Atom Transfer

Given that the new complexes are rich in sulfur, their potential in atom‐transfer catalysis was evaluated next. While a few examples of transition metal‐catalyzed sulfur‐atom transfer to isonitriles have been reported [55, 56, 57, 58], titanium catalysts are currently absent [59, 60]. The synthesis of isothiocyanates from the corresponding isonitriles and elemental sulfur is particularly attractive due to its atom‐economical nature, whereas conventional routes typically rely on toxic reagents such as phosphoryl chloride or carbon disulfide [61]. Moreover, isothiocyanates are industrially relevant intermediates that serve as versatile building blocks for the construction of structurally complex sulfur‐ and nitrogen‐containing molecular architectures [62, 63] in addition to applications in biochemistry [64, 65] and medicine [66, 67]. Because the in situ formation of **1‐(S_2_)_2_** occurs rapidly and selectively, **1­(CH_2_Ph)_2_** was employed as a precatalyst. Reaction optimization was carried out using *tert*‐butyl isonitrile, as a sterically demanding substrate that can be conveniently monitored by ^1^H NMR spectroscopy (Table 1).

**TABLE 1:** Optimization of reaction conditions.

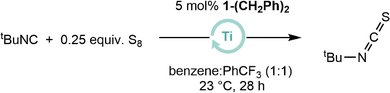		
Entry	Deviation from standard conditions^a^	Yield (%)^b^
1	None	>95
2	No **1‐(CH_2_Ph)_2_**	<5
3	60°C for 4 h	>95
4	**1‐(S_2_)_2_** instead of **1‐(CH_2_Ph)_2_**	>95
5	**1‐Cl_2_** instead of **1‐(CH_2_Ph)_2_**	<5
6	Only benzene‐*d*_6_ as solvent	47
7	Only PhCF_3_ as solvent	55

^a^ 7.6 µmol [Ti], 38.1 µmol S_8_, 152.5 µmol *tert*‐butylisonitrile, 250 µL benzene‐*d*_6_, 250 µL α,α,α‐trifluorotoluene.

^b^ Yields were determined via ^1^H NMR spectroscopy using HMDSO as internal standard.

Gratifyingly, the reaction with 5 mol% of **1‐(CH_2_Ph)_2_** afforded *tert*‐butyl isothiocyanate in quantitative (>95%) yields at 23°C within 28 h using a 1:1 mixture of benzene‐*d*_6_ and α,α,α‐trifluorotoluene as solvent (entry 1). Further conditions and catalysts were evaluated in order to elucidate the role of the titanium complex and reaction conditions. In the absence of **1‐(CH_2_Ph)_2_**, no product was detected (entry 2). Increasing the reaction temperature to 60°C significantly enhanced the catalytic activity, achieving full conversion within 4 h (entry 3). When the titanium bis(disulfide) complex **1‐(S_2_)_2_** was employed as catalyst, full conversion was achieved after just 24 h (entry 4). The dichloride complex **1‐Cl_2_** showed no significant product formation under the same conditions (entry 5). Changing the solvent to pure benzene‐*d*_6_ or α,α,α‐trifluorotoluene (entries 6 and 7) also resulted in a marked decrease in yield. Overall, **1‐(CH_2_Ph)_2_** is a highly active titanium‐based catalyst that shows catalytic activity in S‐atom transfer to *tert*‐butyl isonitrile under very mild reaction conditions. Notably, most conventional routes require harsh temperatures and conditions (e.g., Adam and coworkers: 56°C, 72 h with molybdenum catalyst or Sita and coworkers: 65°C, 18 h with Mo‐catalyst) [56, 57, 58] or the use of stoichiometric reagents [68]. The heavier chalcogens selenium and tellurium were also used as catalysts, but required the addition of triethylamine as a base and harsher conditions (65°C) [69, 70].

**FIGURE 4:**
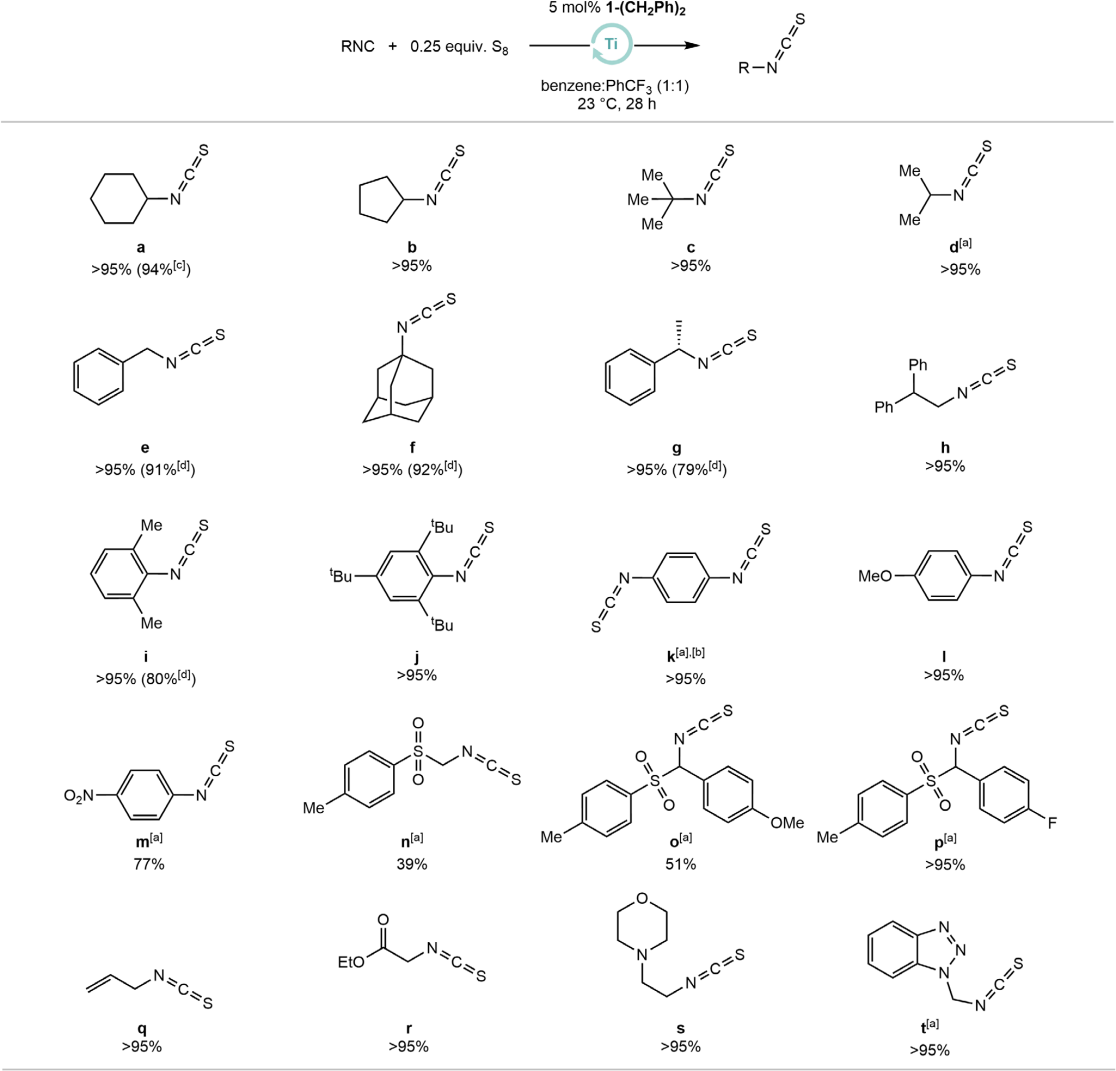
Substrate scope for the catalytic synthesis of isothiocyanates. Standard conditions: 7.6 µmol **1‐(CH_2_Ph)_2_**, 38.1 µmol S_8_, 152.5 µmol isonitrile, 250 µL benzene‐*d*_6_, 250 µL α,α,α‐trifluorotoluene. Yields were determined via ^1^H NMR spectroscopy using HMDSO as internal standard. Isolated yields are shown in brackets; ^[a]^ reaction was carried out at 60°C for 8 h; ^[b]^ 76.3 µmol isonitrile was utilized; ^[c]^ reaction was carried out on 3.67 mmol scale; and ^[d]^ reaction was carried out on 610.0 µmol scale.

To further evaluate the viability of **1‐(CH_2_Ph)_2_** as an effective sulfur‐atom transfer precatalyst, isonitriles featuring different steric profiles and functional groups were examined (Figure 4). Aliphatic isonitriles (**a**–**h**) bearing cyclohexyl, cyclopentyl, *iso*‐propyl, *tert*‐butyl, benzyl, adamantyl, (*S*)‐methylbenzyl, and diphenylmethyl substituents all underwent full conversion under the standard reaction conditions. Notably, the less sterically demanding *iso*‐propyl derivative (**d**) required heating to 60°C to achieve complete conversion. The synthesis of cyclohexyl isothiocyanate was performed on a 3.67 mmol scale, affording a 94% isolated yield of the product, along with 98% recovery of excess sulfur, demonstrating a straightforward work‐up.

Sterically encumbered aryl‐substituted isonitriles, including mesityl (**i**) and 2,4,6‐tri‐*tert*‐butylphenyl (**j**) derivatives, were also evaluated. Remarkably, these bulky substrates reached full conversion at 23°C. The bis(isonitrile) substrate **k** similarly underwent full conversion at 60°C, with formation of a mixed isonitrile–isothiocyanate intermediate observed by ^1^H NMR spectroscopy during the reaction. The selective formation of the corresponding bis(isothiocyanate) from **k** represents a significant advantage over traditional methods, which typically rely on stoichiometric reagents and rarely enable double isonitrile functionalization [61, 71, 72].

Electronically diverse substrates were also well tolerated: methoxy‐ (**l**) and even nitro‐substituted (**m**) isonitriles afforded the desired products efficiently. In contrast, sulfonyl‐containing isonitriles (**n**–**p**) generally resulted in lower yields. Allyl‐ (**q**) and ester‐substituted (**r**) isonitriles, however, were fully converted, providing the corresponding isothiocyanates in quantitative yields. Finally, heterocycle‐containing substrates, including morpholine (**s**) and benzotriazole (**t**) derivatives, also afforded excellent yields. The tolerance of oxygen‐containing functional groups, such as esters and nitro groups, is particularly noteworthy considering the pronounced oxophilicity of titanium [73].

**FIGURE 5:**
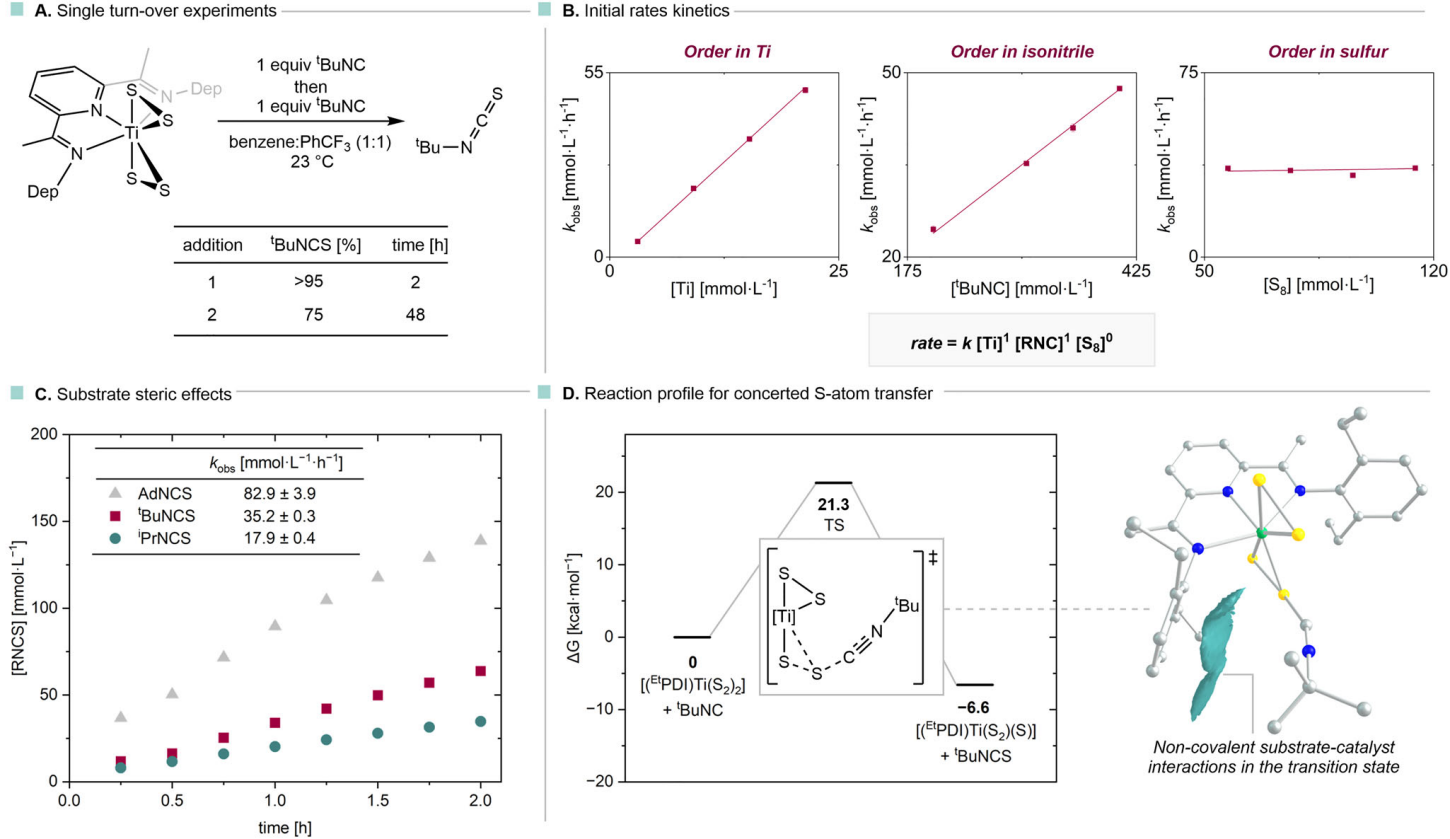
(A) Single turn‐over experiments utilizing **1‐(S_2_)_2_**. (B) Initial rates kinetics and rate law. (C) Steric effects of the substrate on reaction kinetics. (D) DFT‐calculated reaction profile for concerted S‐atom transfer and non‐covalent substrate–catalyst interactions.

To further elucidate the role of the four sulfur atoms in **1‐(S_2_)_2_** for catalysis, single turn‐over experiments were conducted. Treatment of a benzene/TFT solution of **1‐(S_2_)_2_** with one equivalent of *tert*‐butyl isonitrile resulted in full conversion to the isothiocyanate after 2 h. Upon addition of a second equivalent of the isonitrile to this solution, only 75% conversion to the isonitrile was observed after 48 h (Figure 5A). Hence, the first S‐atom transfer is fast and efficient and the second transfer reaction significantly slower with only partial conversion. This supports that **1‐(S_2_)_2_** is able to transfer a maximum of two sulfur‐atoms (in the absence of excess sulfur), presumably forming a titanium sulfido‐sulfide complex **1‐(S_2_)(S)** [41, 42]. However, given that sulfur activation and formation of **1‐(S_2_)_2_** occur rapidly in the presence of excess sulfur, the S‐atom transfer reaction is likely mediated by **1‐(S_2_)_2_**, which generates **1‐(S_2_)(S)** and subsequently reforms **1‐(S_2_)_2_** with elemental sulfur. Analysis of the titanium‐containing species following stoichiometric sulfur atom transfer with either *tert*‐butyl isonitrile or trimethylphosphine did not reveal the formation of a well‐defined product, likely due to the instability of **1‐(S_2_)(S)** in the absence of excess sulfur. To test this hypothesis, initial rate kinetic studies were performed with varying concentrations of titanium, ^t^BuNC, and sulfur (Figure 5B). The initial reaction rate increases linearly with increasing titanium concentration, indicating a first‐order dependence on titanium. Likewise, changes in the isonitrile concentration lead to a linear change in the initial rate, consistent with first‐order behavior in ^t^BuNC. In contrast, varying the sulfur concentration has little effect on the reaction rate, suggesting a zeroth‐order dependence on sulfur. Importantly, **1­(S_2_)_2_**, was identified as the resting state when monitoring the catalytic sulfur‐atom transfer reactions.

In light of the markedly different reaction times required for the catalytic conversion of *tert*‐butyl and *iso*‐propyl‐substituted isonitriles to their corresponding isothiocyanates (Figure 4), the reaction progress was monitored by ^1^H NMR spectroscopy. Notably, the reaction of ^t^BuNC proceeds substantially faster, with initial rates reaching 35.2 mmol L^−1^ h^−1^, in stark contrast to the *iso*‐propyl analogue (*k*_obs_ = 17.9 mmol L^−1^ h^−1^). This trend is further corroborated by the adamantyl‐substituted isonitrile, which exhibits an initial rate of 82.9 mmol L^−1^ h^−1^, significantly higher than that of the *tert*‐butyl analogue (Figure 5C). These observations suggest that increased substrate sterics accelerate the sulfur‐transfer process. However, the rate difference between the adamantyl and *tert*‐butyl derivatives cannot be rationalized by steric effects alone (*vide infra*).

To gain further insights in the key S‐atom transfer step, DFT calculations were performed at the PBE0/def2‐TZVPP//PBE0/def2‐TZVP level of theory using D4 dispersion correction. Two mechanistic scenarios, consistent with the experimental observations were considered: (1) Pre‐coordination of isonitrile to **1‐(S_2_)_2_** prior to C═S bond formation (inner‐sphere mechanism) and (2) concerted C═S bond formation without pre‐coordination (outer‐sphere mechanism). To evaluate the inner‐sphere pathway, the structure of **1‐(S_2_)_2_(^t^BuNC)** was optimized. Although this adduct is 13.4 kcal mol^−1^ less stable than the separated molecules **1‐(S_2_)_2_** and ^t^BuNC, a Curtin–Hammett scenario cannot be excluded. Potential energy surface scans along the C−S coordinate starting from both **1‐(S_2_)_2_(^t^BuNC)** and **1‐(S_2_)_2_** revealed a distinct maximum for pathway (2) (outer sphere) but no maximum for pathway (1) (inner sphere, see Figure S51). From the maximum identified on the potential energy surface for pathway (2), a transition state for sulfur transfer from **1‐(S_2_)_2_** to ^t^BuNC was located, with a calculated activation barrier of 21.3 kcal mol^−1^. This value is consistent with the reaction proceeding at ambient temperature (Figure 5D). Closer inspection of the transition state reveals substantial stabilizing dispersion interactions between the *tert*‐butyl group of the isonitrile and the 2,6‐diethylphenyl substituent on the ligand, contributing −7.0 kcal mol^−1^ [74]. This additional stabilization likely contributes to the higher reaction rate for adamantyl isonitrile relative to the *tert*‐butyl isonitrile.

**SCHEME 1:**
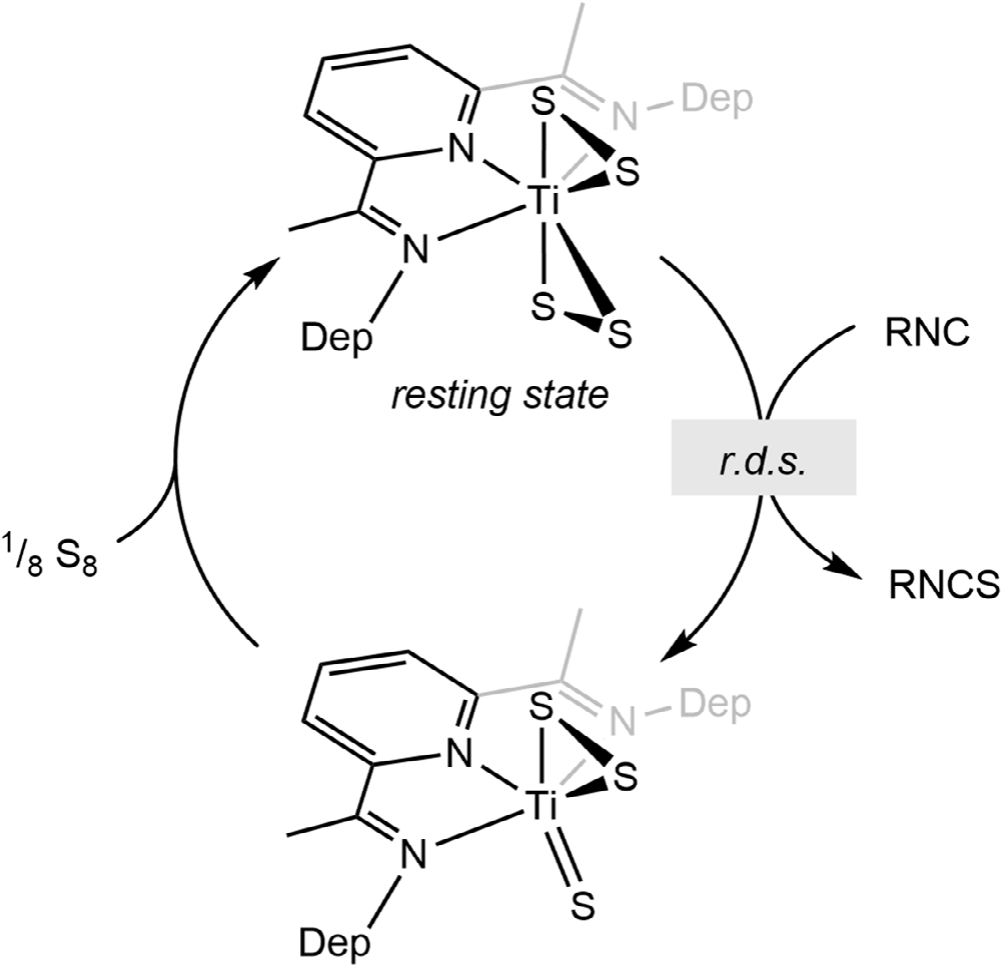
Proposed mechanism for Ti‐catalyzed S‐atom transfer to isonitriles.

Overall, single turn‐over experiments, resting‐state analysis, reaction kinetics, and quantum‐chemical calculations are consistent with the mechanism depicted in Scheme 1. **1‐(S_2_)_2_** is the resting state in catalysis, and sulfur‐atom transfer occurs in a concerted fashion, in contrast to previously reported catalysts that are proposed to operate via a stepwise mechanism [56, 57, 58]. Kinetic analysis reveals that the S‐atom transfer is the rate‐determining step in catalysis and is facilitated by non‐covalent substrate–catalyst interactions. In contrast to prior reports by Heyduk and Wolczanski, the transfer of a single sulfur atom here enables redox‐neutral catalysis without involvement of the metal or the redox‐active ligand [30, 31, 32].

## 3 Conclusion

In summary, a rare four‐electron transformation from a Ti(IV) center was achieved in a single reaction by combining reductive elimination of labile benzyl ligands with metal–ligand cooperativity enabled by a redox‐active pincer‐ligand. This strategy allows access to an unusual titanium bis(disulfide) complex, a sulfur‐rich structural motif that has previously been inaccessible in titanium‐mediated sulfur activation.

The unique TiS_4_ complex was subsequently leveraged for the catalytic functionalization of elemental sulfur in the catalytic conversion of isonitriles to isothiocyanates. To the best of our knowledge, this work represents the first example of a titanium‐based catalyst for sulfur‐atom transfer and the only such system that operates with high activity under ambient conditions utilizing elemental sulfur. A broad range of substrates was compatible with this methodology, delivering mostly quantitative yields and demonstrating tolerance toward oxygen‐containing functional groups as well as highly sterically demanding substituents.

Mechanistic insight was obtained from single turn‐over experiments, initial rate studies as well as kinetic studies with different substrates, which identify the TiS_4_ complex as the catalytically relevant sulfur‐transfer species and suggest a S‐atom transfer rate‐determining step. The redox‐active ligand plays a dual role in this reactivity by enabling selective formation of the TiS_4_‐motif directly from elemental sulfur and providing the steric environment required for S‐atom transfer [75, 76, 77]. Notably, transfer of a single sulfur atom allows redox‐neutral catalysis without engaging the metal or the redox‐active ligand via an outer‐sphere mechanism. This work therefore informs the future design of titanium‐based catalysts for atom‐transfer catalysis beyond the Sharpless epoxidation.

## Author Contributions

**Sebastian Samir Cremer**: investigation, writing – original draft, visualization. **Peter Coburger**: investigation. **Corinna Czernetzki**: investigation. **Gabriele Hierlmeier**: conceptualization, funding acquisition, writing – original draft, writing – review and editing, supervision.

## Conflicts of Interest

The authors declare no conflicts of interest.

## Supporting information



The authors have cited additional references within the Supporting Information [74, 78, 79, 80, 81, 82, 83, 84, 85, 86, 87, 88, 89, 90, 91, 92, 93, 94, 95, 96]. **Supporting File**: anie73686‐sup‐0001‐SuppMat.pdf.

## Data Availability

The data that supports the findings of this study are available in the Supporting Information of this article.
