# Supplementary material for: Catalytic Sulfur‐Atom Transfer Enabled by a Bis(disulfide) Titanium Complex
Source: Angew Chem Int Ed Engl. 2026 Jul 21;65(39):e5196140. doi: 10.1002/anie.5196140 (PMC13592309; doi:10.1002/anie.5196140)
Supplement: Supplementary file 1 [file ANIE-65-e5196140-s001.pdf]

Supplementary Information for

Catalytic Sulfur-Atom Transfer Enabled by a Bis(disulfide) Titanium Complex

Sebastian Samir Cremer, Peter Coburger, Corinna Czernetzki, Gabriele Hierlmeier

Institute of Inorganic Chemistry, Julius-Maximilians-Universität Würzburg, Am Hubland, 97074 Würzburg, Germany

E-mail: gabriele.hierlmeier@uni-wuerzburg.de

Table of Contents

1 Methods and Materials 2

2 Synthetic Procedures for Novel Complexes 3

3 Procedures for Catalytic Studies 5

4 NMR Spectra of Novel Complexes 15

5 NMR Spectra of Isothiocyanates 17

6 Larger Scale Reactions 38

7 Derivatization of (S)-Methylbenzyl Isothiocyanate 46

8 Single-Turnover Experiments 48

9 Kinetic Studies 52

10 X-ray Crystallography 64

11 Quantum Chemical Calculations 66

12 References 77

1 Methods and Materials

All manipulations were performed either under an atmosphere of dry argon or *in vacuo* using standard Schlenk line or glovebox techniques. Deuterated solvents were dried over molecular sieves and degassed by three freeze-pump-thaw cycles prior to use. All other solvents were distilled and degassed from appropriate drying agents. Both deuterated and non-deuterated solvents were stored under argon over activated 4 Å molecular sieves. NMR spectra were acquired either on a Bruker Avance 600 (operating at 600 MHz for ^{1}H and 151 MHz for ^{13}C), a Bruker Avance 500 (operating at 500 MHz for ^{1}H, 126 MHz for ^{13}C) or a Bruker Avance 400 NMR (operating at 400 MHz for ^{1}H and 100 MHz for ^{13}C). Chemical shifts (δ) are given in ppm and internally referenced to the carbon nuclei (^{13}C) or residual protons (^{1}H) of the solvent. Microanalyses (C, H, N, S) were performed on an Elementar Vario MICRO cube elemental analyzer at JMU Würzburg.

(^{Et}PDI)$TiCl_3$[1], (^{Et}PDI)$TiCl_2$[2], (^{Et}PDI)$Ti(CH_2Ph)_2$[3] and $Mg(CH_2Ph)_2$[2] were prepared following literature procedures. All other solid reagents were dried under reduced pressure and transferred in a Glovebox prior to use. Liquid reagents were degassed and dried over activated 4 Å molecular sieves and distilled under reduced pressure or filtered prior to use.

2 Synthetic Procedures for Novel Complexes

(^{Et}PDI)TiCl$_2$(S$_2$)

A vial was charged with (^{Et}PDI)TiCl$_2$ (45.0 mg, 82.7 µmol, 1 equiv.) and S_8 (6.4 mg, 24.8 µmol, 0.3 equiv.) before adding acetonitrile (2 mL). The reaction mixture was stirred at room temperature for 4 h, resulting in a colour change from dark green to light green. Subsequently, the reaction mixture was filtered through celite and dried under reduced pressure. The crude product was washed with toluene (3 mL) and all volatile components were removed *in vacuo* to afford (^{Et}PDI)TiCl$_2$(S$_2$) as a green solid. Single crystals suitable for X-ray diffraction were obtained by slow diffusion of *n*-hexane into a concentrated solution of *α,α,α*-trifluorotoluene (TFT) at room temperature.

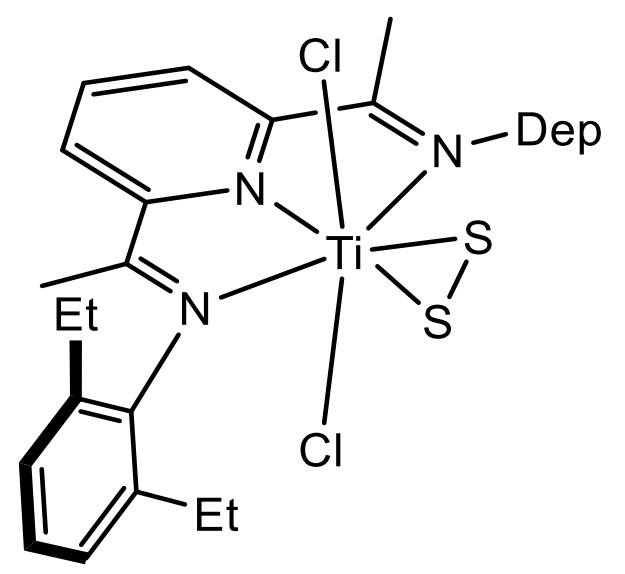

$C_{29}H_{35}Cl_2N_3S_2Ti$, 608.51 g/mol

Yield: 33 mg (64%)

^{1}H NMR (500 MHz, 298 K, acetonitrile-*d*$_3$) δ = 1.11 (t, ^3J = 7.5 Hz, 12H, C$H_{3,Et}$), 2.50 (s, 6H, C$H_{3,CN}$), 3.00-2.91 (m, 4H, C$H_{2,Et}$), 2.79-2.71 (m, 4H, C$H_{2,Et}$), 7.45−7.40 (m, 6H, CH_{Dep}), 8.40−8.38 (m, 2H, *m*-CH_{Pyr}), 8.51 (dd, ^3J = 8.5 Hz, 7.5 Hz, 1H, *p*-CH_{Pyr}) ppm.

^{13}C NMR {H} (151 MHz, 298 K, acetonitrile-*d*$_3$) δ = 15.07 (s, CH$_3$, *C*H$_{3,Et}$), 20.17 (s, CH$_3$, *C*H$_{3,CN}$), 25.06 (s, CH$_2$, *C*H$_{2,Et}$), 127.28 (s, CH, *m*-CH$_{Dep}$), 128.16 (s, CH, *p*-*C*H$_{Dep}$), 129.88 (s, CH, *m*-*C*H$_{Pyr}$), 136.93 (s, C$_q$, *C*$_{Pyr}$), 143.32 (s, CH, *p*-*C*H$_{Pyr}$), 149.69 (s, C$_q$, *C*N$_{Dep}$), 150.65 (s, C$_q$, *C*$_{Dep}$), 175.60 (s, C$_q$, *C*$_{CN}$) ppm.

Elemental analysis (%) calc. for $C_{29}H_{35}Cl_2N_3S_2Ti$ [487.19 g mol^{-1}]: C, 78.12; H, 6.89; N, 7.01; S 10.54. **Found**: C, 77.96; H, 7.12; N, 6.83; S 10.45.

(^{Et}PDI)Ti(S_2)$_2$

A vial was charged with (^{Et}PDI)Ti(CH_2Ph)$_2$ (50.0 mg, 76.3 µmol, 1 equiv.) and S_8 (19.6 mg, 76.3 µmol, 1 equiv.) before adding benzene (2 mL). The reaction mixture was stirred at room temperature for 4 h, resulting in a dark green suspension, which was filtered over Celite. The solid crude product was washed with benzene (3 x 1 mL) and the solid was extracted with acetonitrile. All volatile components were removed *in vacuo* to afford (^{Et}PDI)Ti(S_2)$_2$ as a green solid. Single crystals suitable for X-ray diffraction were obtained by slow diffusion of *n*−pentane into a concentrated solution of THF at −35 °C.

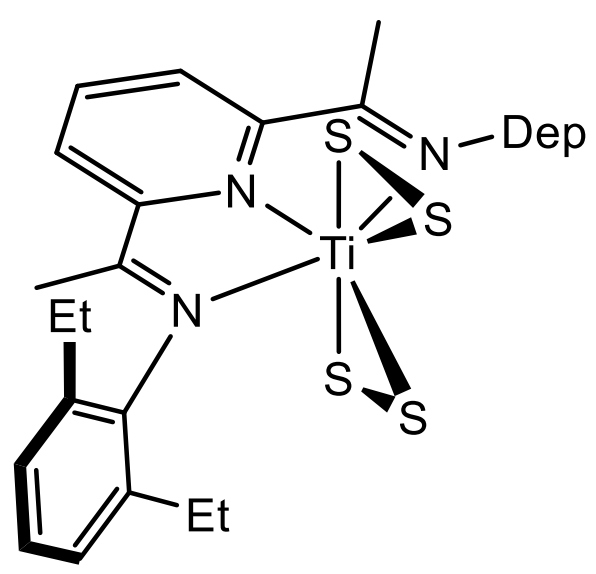

$C_{29}H_{35}N_3S_4Ti$, 601.73 g/mol

Yield: 21.5 mg (47%)

^{1}H NMR (500 MHz, 298 K, acetonitrile-*d*$_3$) δ = 0.94 (t, ^3J = 7.5 Hz, 12H, C$H_{3,Et}$), 2.11 (s, 6H, C$H_{3,CN}$), 2.16-2.08 (m, 4H, C$H_{2,Et}$), 2.43-2.35 (m, 4H, C$H_{2,Et}$), 6.92 (d, ^3J = 7.4 Hz, 4H, *m*-CH_{Dep}), 7.01 (dd, ^3J = 8.1 Hz, 7.1 Hz, 2H, *p*-CH_{Dep}), 8.52−8.50 (m, 2H, *m*-CH_{Pyr}), 8.64 (dd, ^3J = 8.6 Hz, 7.6 Hz, 1H, *p*-CH_{Pyr}) ppm.

^{13}C NMR {H} (151 MHz, 298 K, acetonitrile-*d*$_3$) δ = 15.73 (s, CH_3, $CH_{3,Et}$), 19.24 (s, CH_3, $CH_{3,CN}$), 24.37 (s, CH_2, $CH_{2,Et}$), 126.46 (s, CH, *m*-CH_{Dep}), 127.43 (s, CH, *p*-CH_{Dep}), 130.80 (s, CH, *m*-CH_{Pyr}), 135.80 (s, C_q, C_{Pyr}), 142.10 (s, CH, *p*-CH_{Pyr}), 147.09 (s, C_q, CN_{Dep}), 150.87 (s, C_q, C_{Dep}), 174.36 (s, C_q, C_{CN}) ppm.

Elemental analysis (%) calc. for $C_{29}H_{35}N_3S_4Ti$ [601.73 g mol^{-1}]: C, 57.89; H, 5.86; N, 6.98; S 21.31. **Found**: C, 56.59; H, 5.73; N, 6.48; S 21.41.

3 Procedures for Catalytic Studies

Yields were determined relative to the internal standard integral using characteristic signals of the corresponding substrate.

General Procedure A

A J Young tube was charged with (^{Et}PDI)Ti(CH_2Ph)$_2$ (5 mg, 7.6 µmol, 0.05 equiv.) and sulfur (9.8 mg, 38.1 µmol, 0.25 equiv.) before adding benzene-d_6 (250 µL) and α,α,α-trifluorotoluene (250 µL). Subsequently, the isonitrile (152.5 µmol, 1 equiv.) and hexamethyldisiloxane (HMDSO, 5 µL, 23.5 µmol, internal standard) were added. The reactions were carried out at 23 °C and monitored via ^{1}H NMR spectroscopy.

General Procedure B

A J Young tube was charged with (^{Et}PDI)Ti(CH_2Ph)$_2$ (5 mg, 7.6 µmol, 0.05 equiv.) and sulfur (9.8 mg, 38.1 µmol, 0.25 equiv.) before adding benzene-d_6 (250 µL) and α,α,α-trifluorotoluene (250 µL). The respective isonitrile (152.5 µmol, 1 equiv.) was added before, lastly, hexamethyldisiloxane (HMDSO, 5 µL, 23.5 µmol, internal standard) was added as internal standard. The reactions were carried out at 60 °C and monitored via ^{1}H NMR spectroscopy.

Note: Using 0.17 equiv. of sulfur resulted in a significantly slower reaction with only 55% product formation after 28 h at 23 °C using ^{t}BuNC as substrate following **General Procedure A**.

Isothiocyanatocyclohexane

The title compound was synthesized following **General Procedure A** in >95% yield.

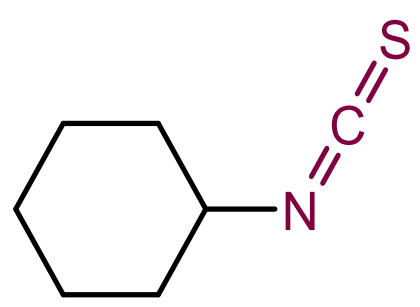

^{1}H NMR (500 MHz, 298 K, benzene-d_6) δ = 1.17-0.87 (m, 6H, C$H_{2,Cy}$), 1.36-1.30 (m, 4H, CH_2CH), 3.00-2.95 (m, 1H, C*H*) ppm.

^{13}C NMR {H} (151 MHz, 298 K, benzene-d_6) δ = 23.31 (s, CH_2, *C*$H_2CH_2CH_2$CH), 25.08 (s, CH_2, *C*H_2CH_2CH), 32.20 (s, CH_2, *C*H_2CH), 55.55 (s, CH, *C*H), 131.84 (s, C_q, N*C*S, overlapping with TFT signal, assigned *via* HMBC) ppm.

The analytical data is consistent with previous reports.[4]

Isothiocyanatocyclopentane

The title compound was synthesized following **General Procedure A** in >95% yield.

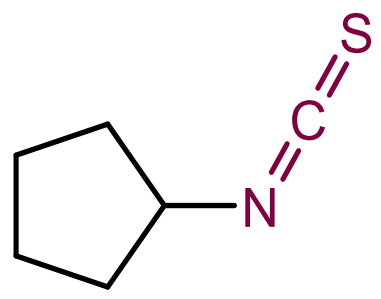

^{1}H NMR (500 MHz, 298 K, benzene-d_6) δ = 1.16-1.08 (m, 2H, CH_2)1.37-1.27 (m, 2H, CH_2), 1.45-1.40 (m, 4H, CH_2), 2.06 (pent, ^3J = 7.5 Hz, 1H, CH) ppm.

^{13}C NMR {H} (151 MHz, 298 K, benzene-d_6) δ = 24.86 (s, CH_2, CH$_2$CH), 28.06 (s, CH, CH), 31.25 (s, CH_2, CH$_2$CH), 123.04 (s, C_q, NCS) ppm.

The analytical data is consistent with previous reports.[5]

2-Isothiocyanato-2-methylpropane

The title compound was synthesized following **General Procedure A** in >95% yield.

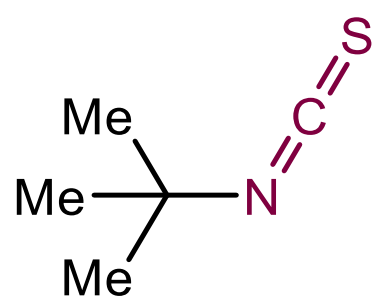

^{1}H NMR (500 MHz, 298 K, benzene-d_6) δ = 0.94 (s, 9H, CH_3) ppm.

^{13}C NMR {H} (151 MHz, 298 K, benzene-d_6) δ = 30.10 (s, CH_3, CH$_3$), 58.21 (s, C_q, C(CH$_3$)$_3$), 131.85 (s, C_q, NCS, overlapping with TFT signal, assigned *via* HMBC) ppm.

The analytical data is consistent with previous reports.[4]

2-Isothiocyanatopropane

The title compound was synthesized following **General Procedure B** in >95% yield.

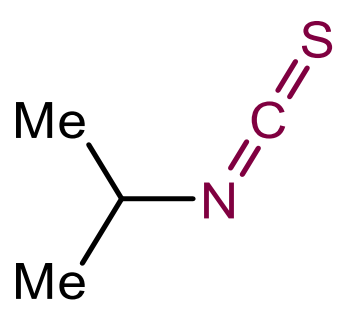

^{1}H NMR (500 MHz, 298 K, benzene-d_6) δ = 0.76 (d, ^3J = 6.5 Hz, 6H, CH_3), 3.14 (ps-spt, ^3J = 6.5 Hz, 1H, CH) ppm.

^{13}C NMR {H} (151 MHz, 298 K, benzene-d_6) δ = 22.99 (s, CH_3, CH$_3$), 49.58 (s, CH, CH), 131.49 (s, C_q, NCS) ppm.

The analytical data is consistent with previous reports.[6]

(Isothiocyanatomethyl)benzene

The title compound was synthesized following **General Procedure A** in >95% yield.

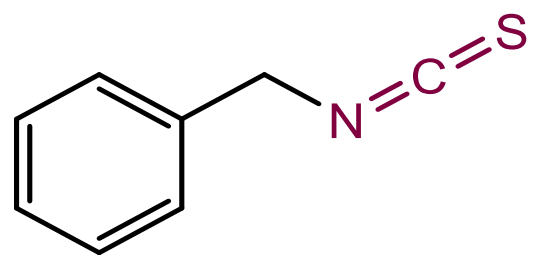

^{1}H NMR (500 MHz, 298 K, benzene-*d*$_6$) δ = 3.85 (s, 2H, C*H*$_{2Ph}$), 6.84-6.82 (m, 2H, *o*/*m*-C*H*$_{Ar}$), 7.07 (*o*/*m*-C*H*$_{Ar}$, overlapping with TFT signal, assigned *via* HSQC and HMBC), 7.15 (*p*-CH$_{Ph}$, overlapping with benzene-*d*$_6$ signal, assigned *via* HSQC and HMBC) ppm.

^{13}C NMR {H} (151 MHz, 298 K, benzene-*d*$_6$) δ = 48.26 (s, CH$_2$, *C*H$_{2Ph}$), 126.88 (s, CH, *o*/*m*-*C*H$_{Ph}$), 128.34 (s, CH, *p*-*C*H$_{Ph}$), 128.99 (s, CH, *o*/*m*-*C*H$_{Ph}$), 133.33 (s, br, C$_q$, N*C*S), 134.72 (s, C$_q$, *C*$_{Ph}$) ppm.

The analytical data is consistent with previous reports.[4]

(3s,5s,7s)-1-Isothiocyanatoadamantane

The title compound was synthesized following **General Procedure A** in >95% yield.

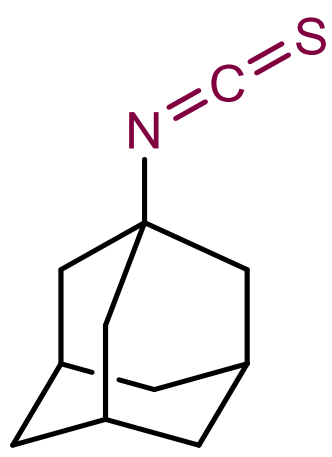

^{1}H NMR (500 MHz, 298 K, benzene-*d*$_6$) δ = 1.32-1.24 (m, 6H, CHC*H*$_2$CH), 1.57 (d, ^3J = 2.6 Hz, 6H, C*H*$_2$CNCS), 1.69-1.65 (m, 3H, C*H*) ppm.

^{13}C NMR {H} (151 MHz, 298 K, benzene-*d*$_6$) δ = 29.57 (s, CH, *C*H), 35.50 (s, CH$_2$, CH*C*H$_2$CH), 43.76 (s, CH$_2$, *C*H$_2$CNCS), 58.58 (s, C$_q$, *C*NCS), 129.77 (s, C$_q$, N*C*S) ppm.

The analytical data is consistent with previous reports.[7]

1-((Isothiocyanatomethyl)sulfonyl)-4-methylbenzene

The title compound was synthesized following **General Procedure B** in 39% yield.

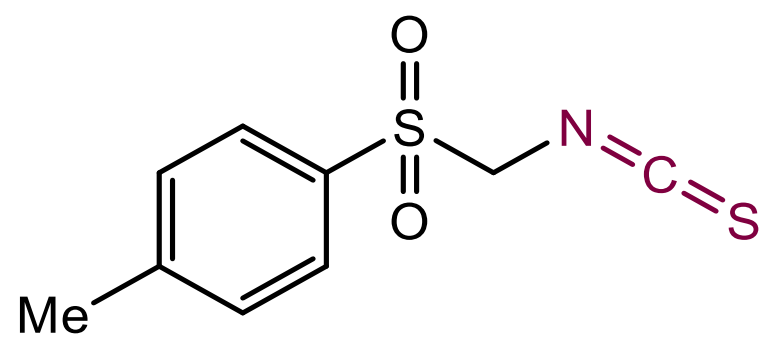

^{1}H NMR (500 MHz, 298 K, benzene-d_6) δ = 1.95 (s, 3H, CH_3), 3.77 (s, 2H, CH_2NCS), 6.86-6.82 (m, 2H, *m*-CH_{Ar}), 7.67-7.64 (m, 2H, *o*-CH_{Ar}) ppm.

^{13}C NMR {H} (151 MHz, 298 K, benzene-d_6) δ = 21.07 (s, CH_3, CH_3, overlapping with isonitrile signal), 60.60 (s, CH_2, CH_2), 129.23 (s, CH, *m*-CH_{Ar}), 130.36 (s, CH, *o*-CH_{Ar}), 133.99 (s, C_q, CSO_2), 146.28 (s, C_q, CCH_3) ppm.

Signal of the SCN-carbon was not observed, concurring with analytical data from previous reports.[7]

Ethyl 2-isothiocyanatoacetate

The title compound was synthesized following **General Procedure B** in >95% yield.

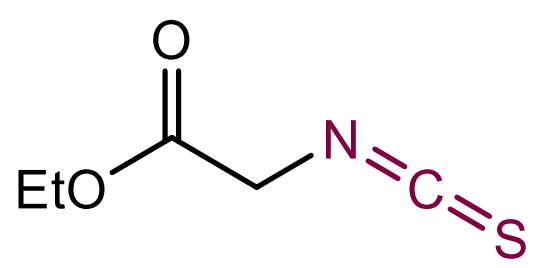

^{1}H NMR (500 MHz, 298 K, benzene-d_6) δ = 0.91 (t, ^3J = 7.2 Hz, 3H, C$H_{3,Et}$), 3.28 (s, 2H, CH_2NCS), 3.84 (q, ^3J = 7.2 Hz, 2H, C$H_{2,Et}$) ppm.

^{13}C NMR {H} (151 MHz, 298 K, benzene-d_6) δ = 13.70 (s, CH_3, $CH_{3,Et}$), 45.71 (s, CH_2, CH_2NCS), 62.18 (s, CH_2, $CH_{2,Et}$), 138.49 (s, C_q, NCS), 165.81 (s, C_q, CO_2Et) ppm.

The analytical data is consistent with previous reports.[4]

1-Isothiocyanato-4-methoxybenzene

The title compound was synthesized following **General Procedure A** in >95% yield.

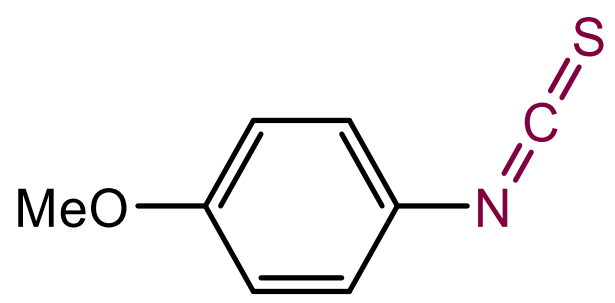

^{1}H NMR (500 MHz, 298 K, benzene-*d*$_6$) δ = 3.24 (s, CH_3, O*C**H*$_3$), 6.43-6.40 (m, 2H, CH_{Ar}), 6.64-6.60 (m, 2H, CH_{Ar}) ppm.

^{13}C NMR {H} (151 MHz, 298 K, benzene-*d*$_6$) δ = 54.82 (s, CH_3, O*C*H_3), 114.89 (s, CH, *C*H_{Ar}), 123.90 (s, C_q, *C*NCS, overlapping with TFT signal, assigned *via* HMBC), 126.92 (s, CH, *C*H_{Ar}), 135.18 (s, C_q, N*C*S), 159.03 (s, C_q, *C*OCH_3) ppm.

The analytical data is consistent with previous reports.[7]

2-Isothiocyanato-1,3-dimethylbenzene

The title compound was synthesized following **General Procedure A** in >95% yield.

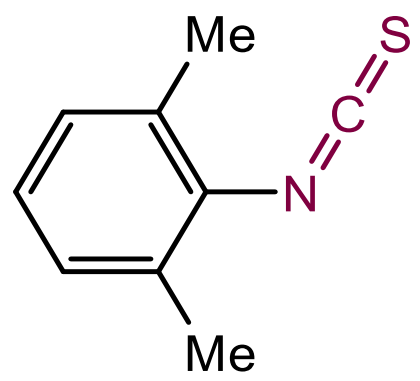

^{1}H NMR (500 MHz, 298 K, benzene-*d*$_6$) δ = 1.99 (s, 6H, C*H*$_3$), 6.66 (dq, ^3J = 7.5 Hz, ^4J = 0.6 Hz, 2H, *m*-C*H*), 6.75 (dd, ^3J = 7.5 Hz, 1H, *p*-C*H*) ppm.

^{13}C NMR {H} (151 MHz, 298 K, benzene-*d*$_6$) δ = 18.18 (s, CH_3, *C*H_3), 127.05 (s, CH, *p*-*C*H), 128.15 (s, CH, *m*-*C*H), 129.96 (s, C_q, *C*NCS), 135.19 (s, C_q, *C*CH_3),136.53 (s, C_q, N*C*S) ppm.

The analytical data is consistent with previous reports.[4]

1,3,5-tri-*tert*-Butyl-2-isothiocyanatobenzene

The title compound was synthesized following **General Procedure A** in >95% yield.

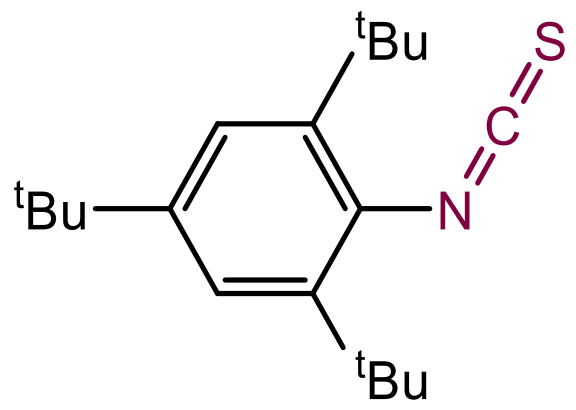

^{1}H NMR (500 MHz, 298 K, benzene-d_6) δ = 1.23 (s, 9H, *p*-C(CH_3)$_3$), 1.40 (s, 18H, *o*-C(CH_3)$_3$), 7.40 (s, 2H, CH) ppm.

^{13}C NMR {H} (151 MHz, 298 K, benzene-d_6) δ = 30.09 (s, CH_3, *o*-C(*C*H_3)$_3$), 31.23 (s, CH_3, *p*-C(*C*H_3)$_3$), 35.34 (s, C_q, *p*-*C*(CH_3)$_3$), 35.83 (s, C_q, *o*-*C*(CH_3)$_3$), 122.27 (s, CH, *C*H), 124.99 (s, C_q, *C*NCS), 134.04 (s, br, C_q, N*C*S), 147.34 (s, C_q, *o*-*C*C(CH_3)$_3$), 149.90 (s, C_q, *p*-*C*C(CH_3)$_3$) ppm.

The analytical data is consistent with previous reports.[8]

1,4-Diisothiocyanatobenzene

The title compound was synthesized following **General Procedure B** in >95% yield.

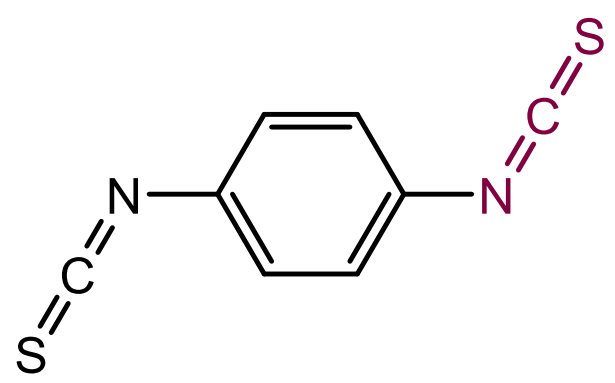

^{1}H NMR (500 MHz, 298 K, benzene-d_6) δ = 6.34 (s, 4H, CH_{Ar}) ppm.

^{13}C NMR {H} (151 MHz, 298 K, benzene-d_6) δ = 126.10 (s, CH, *C*H_{Ar}), 129.61 (s, C_q, *C*NCS), 137.37 (s, C_q, N*C*S) ppm.

The analytical data is consistent with previous reports.[9]

(*S*)-(-)-α-Methylbenzylisothiocyanate

The title compound was synthesized following **General Procedure A** in >95% yield.

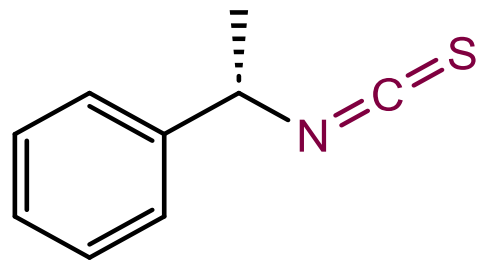

^{1}H NMR (600 MHz, 298 K, benzene-d_6) δ = 1.11 (d, ^3J = 6.8 Hz, 3H, CH_3), 4.20 (q, ^3J = 6.8 Hz, 1H, C*H*), 6.93-6.91 (m, 2H, *m*-CH_{Ar}), 7.04 (*o/p*-CH_{Ar}, overlapping with TFT signal, assigned via HSQC and HMBC) ppm.

^{13}C NMR {H} (151 MHz, 298 K, benzene-d_6) δ = 24.48 (s, CH_3, CH_3), 57.22 (s, CH, *C*H), 125.54 (s, CH, *o*/*m*-CH_{Ph}), 128.27 (s, CH, *p*-CH_{Ph}), 129.03 (s, CH, *o*/*m*-CH_{Ph}), 133.73 (s, br, C_q, N*C*S), 140.67 (s, C_q, C_{Ph}) ppm.

2-Morpholinoethylisothiocyanate

The title compound was synthesized following **General Procedure A** in >95% yield.

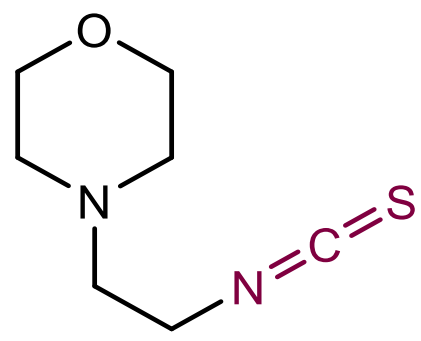

^{1}H NMR (600 MHz, 298 K, benzene-d_6) δ = 1.97 (t, ^3J = 6.1 Hz, 2H, CH_{2Et}), 2.02-2.00 (m. 4H, CH_{2Morph}), 2.80 ((t, ^3J = 6.1 Hz, 2H, CH_{2Et}), 2.47-2.46 (m, 4H, CH_{2Morph}) ppm.

^{13}C NMR {H} (151 MHz, 298 K, benzene-d_6) δ = 42.66 (s, CH_2, CH_{2Et}), 53.41 (s, CH_2, CH_{2Morph}), 57.58 (s, CH_2, CH_{2Et}), 66.94 (s, CH_2, CH_{2Morph}), 134.21 (s, br, C_q, N*C*S) ppm.

The analytical data is consistent with previous reports.[4]

α-Tosyl-(4-methoxybenzyl) isothiocyanate

The title compound was synthesized following **General Procedure B** in 51% yield.

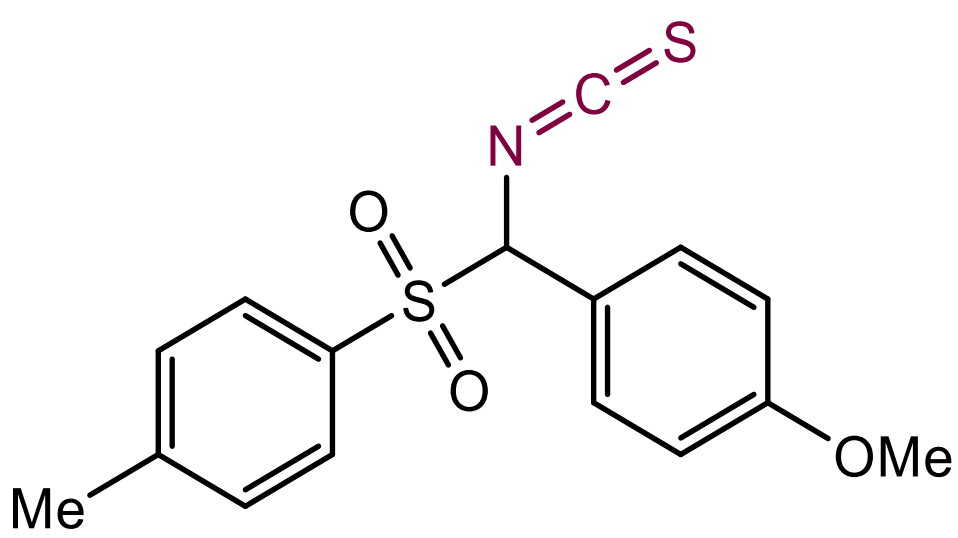

^{1}H NMR (600 MHz, 298 K, benzene-d_6) δ = 1.95 (s, 3H, CH_{3Me}), 3.31 (s, 3H, CH_{3OMe}), 5.17 (s, 1H, CH), 6.57-6.56 (m, 2H, CH_{Ar}), 6.81-6.80 (m, 2H, CH_{Ar}), 7.08 (CH$_{Ar}$, overlapping with TFT signal, assigned via HSQC) 7.57-7.56 (m, 2H, CH_{Ar}) ppm.

^{13}C NMR {H} (151 MHz, 298 K, benzene-d_6) δ = 21.12 (s, CH$_3$, CH$_{2Me}$), 54.76 (s, CH$_3$, CH$_{3OMe}$), 79.17 (s, CH, CH), 114.19 (s, CH, CH$_{Ar}$), 121.17 (s, C$_q$, CCH), 129.97 (s, CH, CH$_{Ar}$), 130.26 (s, CH, CH$_{Ar}$), 130.29 (s, CH, CH$_{Ar}$), 132.61 (s, Cq, CSO$_2$), 146.08 (s, C$_q$, CCH$_3$), 141.12 (s, br, C$_q$, NCS), 161.72 (s, C$_q$, COMe) ppm.

The analytical data is consistent with previous reports.[9]

α-Tosyl-(4-fluorobenzyl) isothiocyanate

The title compound was synthesized following **General Procedure B** in >95% yield.

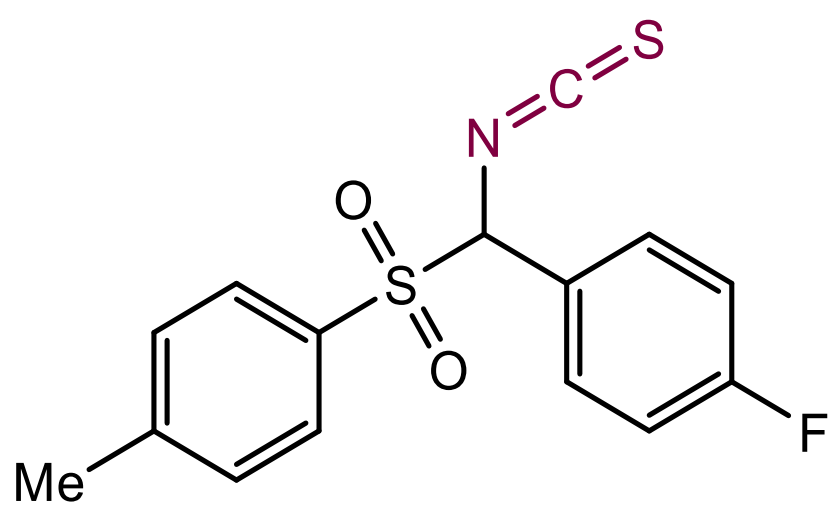

^{1}H NMR (600 MHz, 298 K, benzene-d_6) δ = 1.95 (s, 3H, CH_{3Me}), 5.10 (s, 1H, CH), 6.42-6.51 (m, 2H, CH_{Ar}), 6.79-6.78 (m, 2H, CH_{Ar}), 6.97-6.95 (m, CH, *m*-CH_{PhF}), 7.49-7.47 (m, 2H, *o*-CH_{PhCF}) ppm.

^{13}C NMR {H} (151 MHz, 298 K, benzene-d_6) δ = 21.10 (s, CH$_3$, CH$_{3Me}$), 78.71 (s, CH, CH), 115.58 (s, CH, CH$_{Ar}$), 115.73 (s, CH, CH$_{Ar}$), 130.18 (d, $^2J_{C\text{-}F}$ = 42.0 Hz, CH, *o*-CH$_{PhF}$), 130.77 (d, $^3J_{C\text{-}F}$ = 8.5 Hz, CH, *m*-CH$_{PhF}$, one doublet-signal is overlapping with TFT signal, assigned via 90 DEPT and 135 DEPT), 132.08 (s, C$_q$, CPhF), 136.77 (s, C$_q$, CSO$_2$), 141.88 (s, br, C$_q$, NCS), 146.32 (s, C$_q$, CCH$_3$), 164.23 (d, $^1J_{C\text{-}F}$ = 250.3 Hz, C$_q$, CF) ppm.

^{19}F NMR {^{1}H} (565 MHz, 298 K, benzene-d_6) δ = −110.52 - −110.57 (m, F) ppm.

The analytical data is consistent with previous reports.[9]

2,2-Diphenylethylisothiocyanate

The title compound was synthesized following **General Procedure A** in >95% yield.

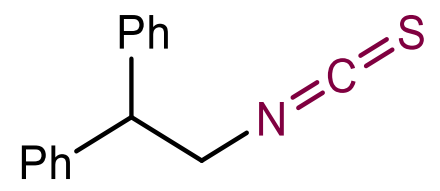

^{1}H NMR (600 MHz, 298 K, benzene-d_6) δ = 3.37 (d, ^3J = 7.5 Hz, 2H, CH_{2Et}), 3.84 (t, ^3J = 7.5 Hz, 1H, CH_{Et}), 6.90-6.89 (m, 4H, *o*-CH_{Ph}), 7.02 (*m*/*p*-CH_{Ph}, overlapping with TFT signal, assigned via HMBC). 7.08 (*m*/*p*-CH_{Ph}, overlapping with TFT signal, assigned via HMBC) ppm.

^{13}C NMR {H} (151 MHz, 298 K, benzene-d_6) δ = 49.11 (s, CH_2, CH_{2Et}), 51.66 (s, CH, CH_{Et}), 127.45 (s, CH, *p*-CH_{Ar}), 128.18 (s, CH, *o*-CH_{Ar}), 128.99 (s, CH, *m*-CH_{Ar}), 132.89 (s, br, C_q, N*C*S), 140.81 (s, C_q, C_{qAr}) ppm.

The analytical data is consistent with previous reports.[4]

1-Isothiocyanato-4-nitrobenzene

The title compound was synthesized following **General Procedure B** in 77% yield.

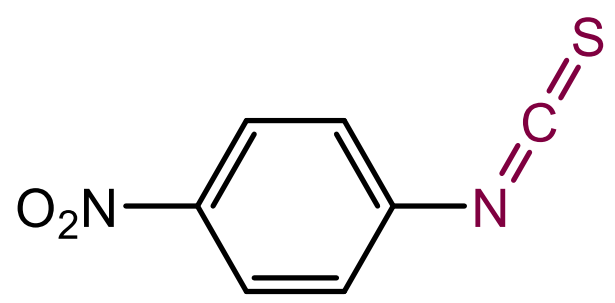

^{1}H NMR (600 MHz, 298 K, benzene-d_6) δ = 6.58-6.56 (m, CH, CH_{Ar}), 7.53-7.52 (m, CH, CH_{Ar}) ppm.

^{13}C NMR {H} (151 MHz, 298 K, benzene-d_6) δ = 124.59 (s, CH, CH_{Ar}), 127.07 (s, CH, CH_{Ar}), 140.21 (s, C_q, N*C*S), 147.44 (s, C_q, *C*NCS), 171.83 (s, C_q, *C*NO_2) ppm.

The analytical data is consistent with previous reports.[4]

Allylisothiocyanate

The title compound was synthesized following **General Procedure A** in >95% yield.

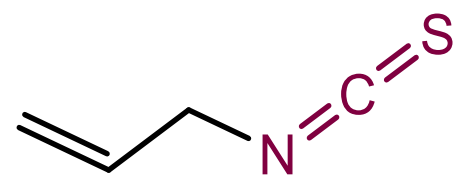

^{1}H NMR (500 MHz, 298 K, benzene-d_6) δ = 1.32-1.30 (m, overlapping signals CH_{2NCS} and CH_{Allyl}, assigned via ^{1}H,^{1}H-COSY and HSQC), 4.91-4.86 (m, 1H, CH_{Allyl}), 5.18-5.16 (m, 1H, CH, CH_{CH2NCS}) ppm.

^{13}C NMR {H} (151 MHz, 298 K, benzene-d_6) δ = 12.15 (s, CH_2, CH_{2NCS}), 116.10 (s, CH, CH_{CH2NCS}), 127.26 (s, CH, CH_{Allyl}), 135.43 (s, C_q, N*C*S) ppm.

The analytical data is consistent with previous reports.[4]

(1*H*-Benzotriazol-1-yl)methylisothiocyanate

The title compound was synthesized following **General Procedure B** in >95% yield.

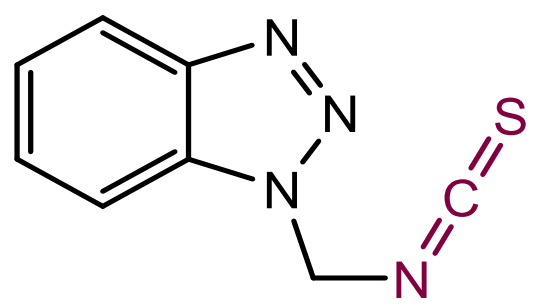

^{1}H NMR (500 MHz, 298 K, benzene-d_6) δ = 5.23 (s, 2H, CH_2), 6.96-6.94 (m, 1H, CH_{Ar}), 7.00 (CH_{Ar}, overlapping with TFT signal, assigned via ^{1}H,^{1}H-COSY and HSQC), 7.34 (CH_{Ar}, overlapping with TFT signal, assigned via ^{1}H,^{1}H-COSY and HSQC), 7.82-7.80 (m, 1H, CH_{Ar}) ppm.

^{13}C NMR {H} (151 MHz, 298 K, benzene-d_6) δ = 55.32 (s, CH_2, CH_2), 109.07 (s, CH, CH_{Ar}), 120.53 (s, CH, CH_{Ar}), 124.60 (s, CH, CH_{Ar}), 128.39 (s, CH, CH_{Ar}), 132.39 (s, C_q, C_{qAr}), 142.55 (s, br. C_q, N*C*S), 146.83 (s, C_q, C_{qAr}) ppm.

The analytical data is consistent with previous reports.[4]

4 NMR Spectra of Novel Complexes

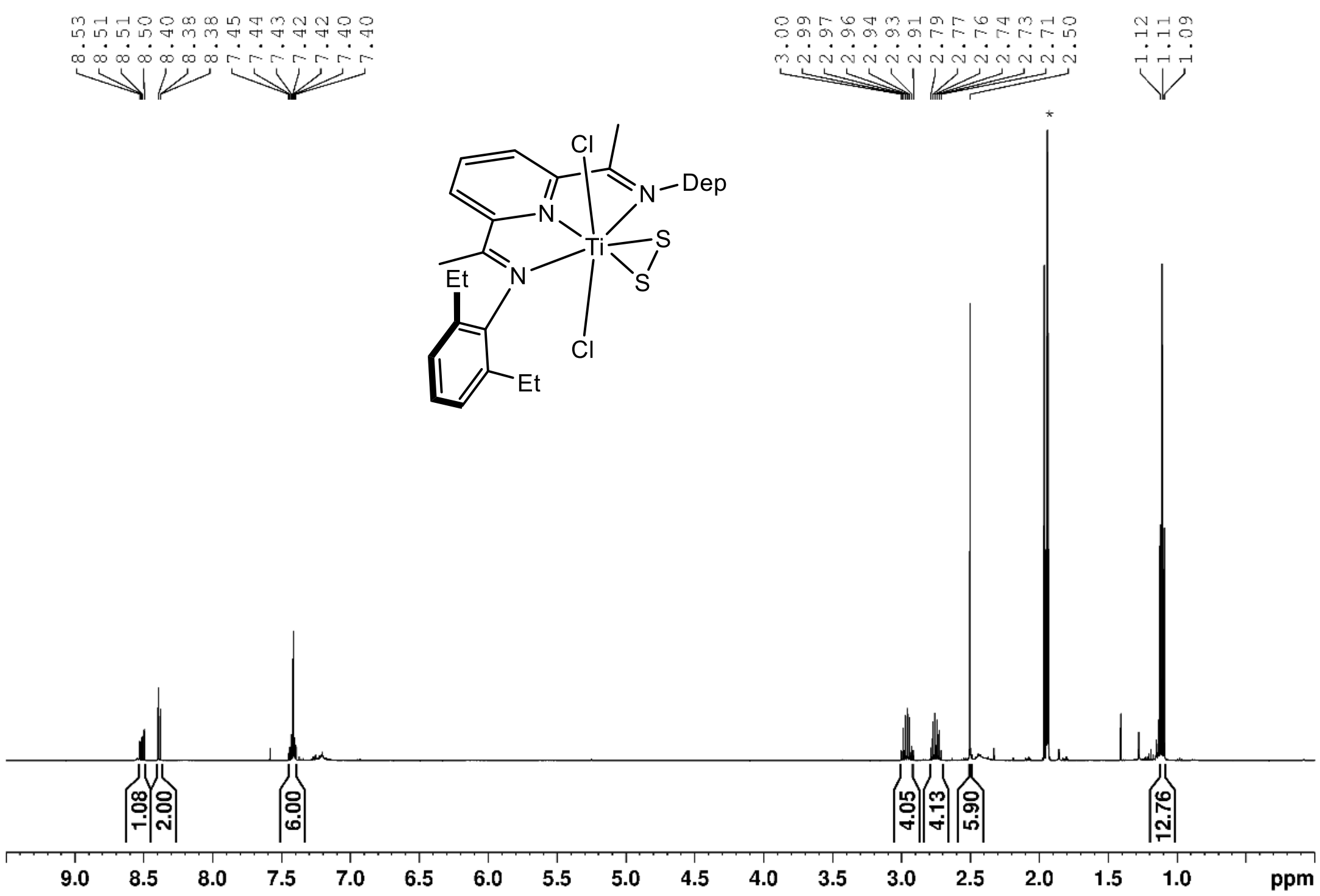

Figure S 1. ^{1}H NMR spectrum of ^{Et}PDITiCl2S2 (500 MHz, 298 K, acetonitrile-d_3). Residual signals of deuterated solvent are marked with an asterisk (*).

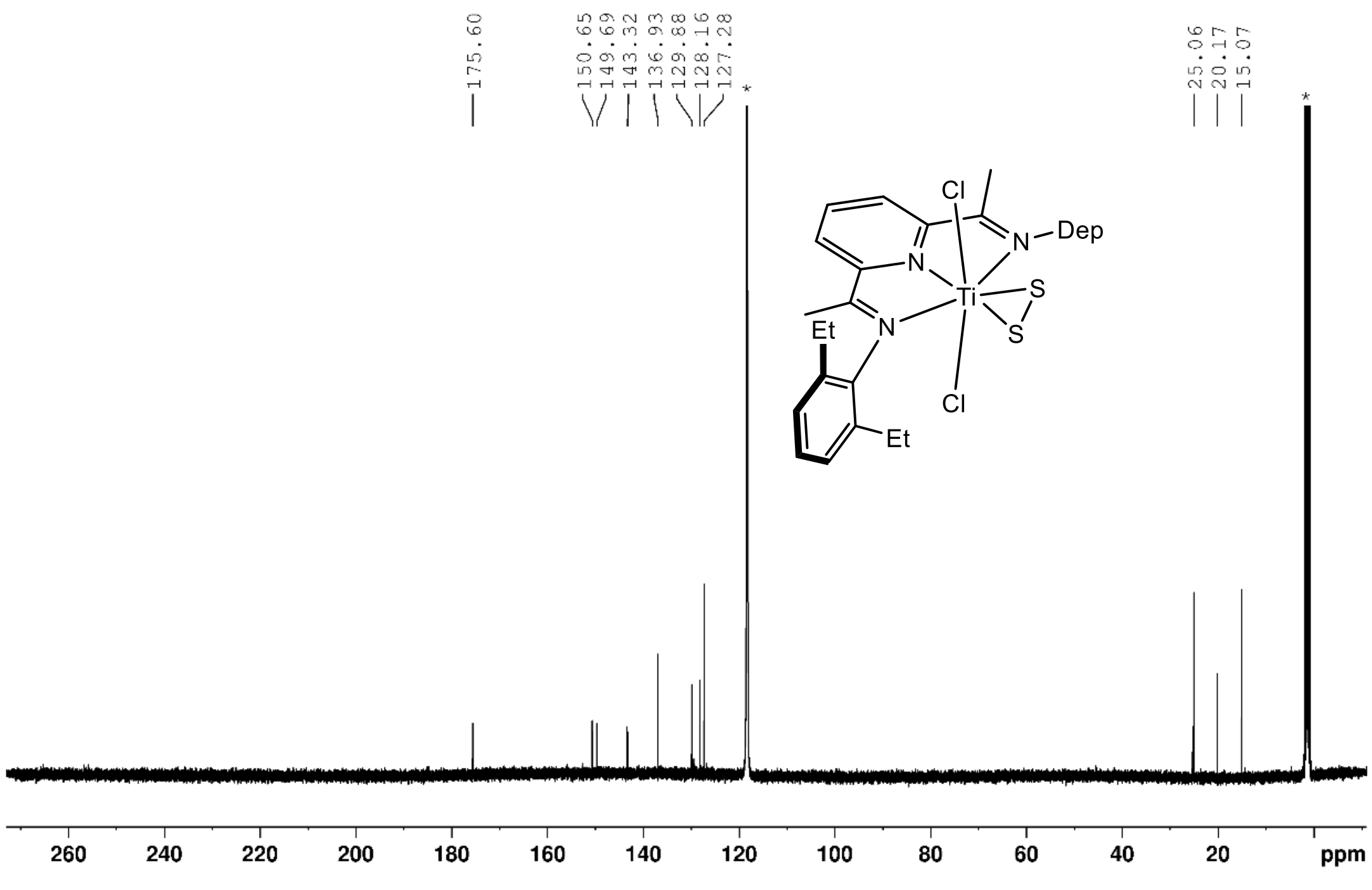

Figure S 2. ^{13}C NMR spectrum of ^{Et}PDITiCl$_2$S$_2$ (126 MHz, 298 K, acetonitrile-d_3). Residual signals of deuterated solvent are marked with an asterisk (*).

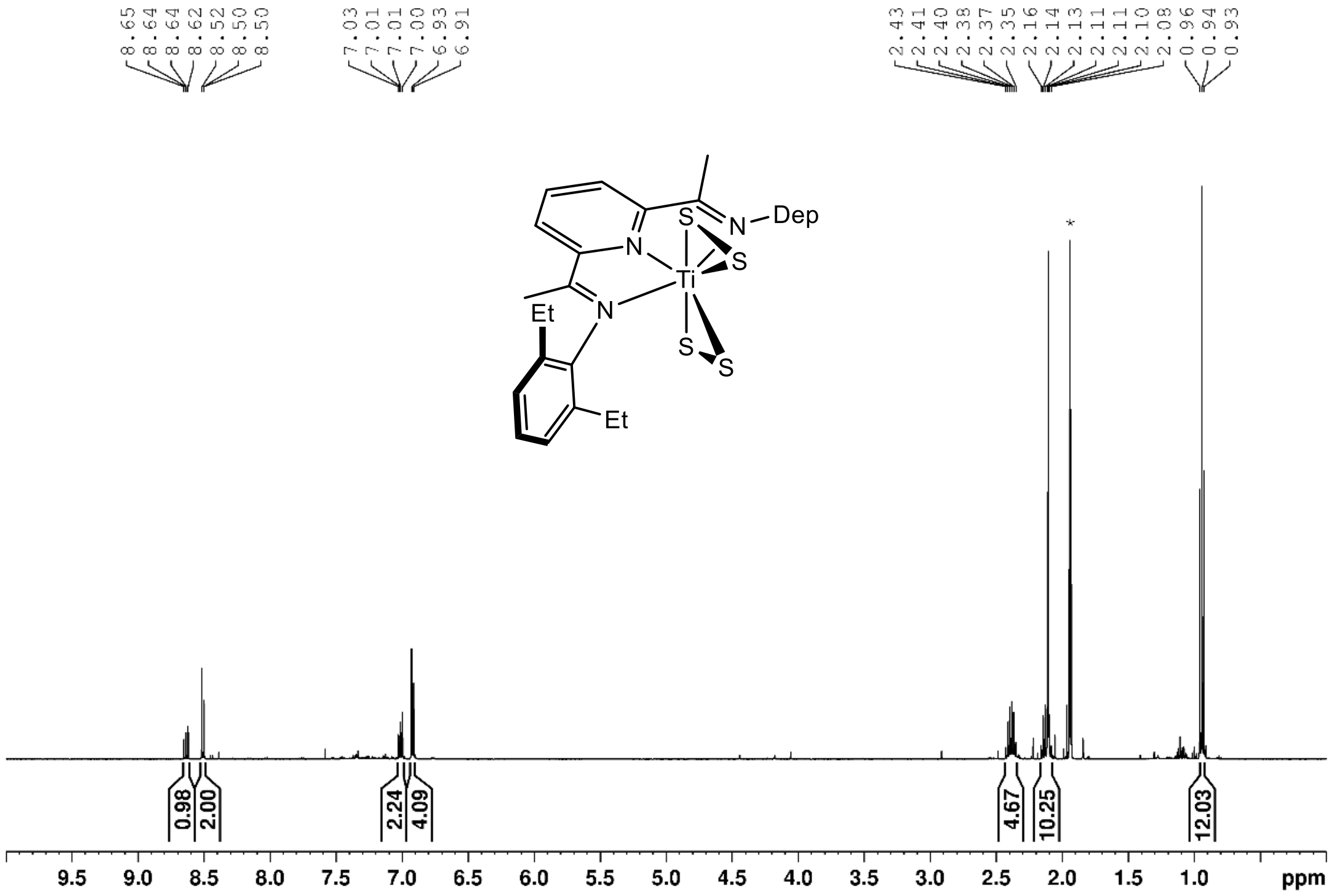

Figure S 3. ^{1}H NMR spectrum of ^{Et}PDITi$(S_2)_2$ (500 MHz, 298 K, acetonitrile-d_3). Residual signals of deuterated solvent are marked with an asterisk (*).

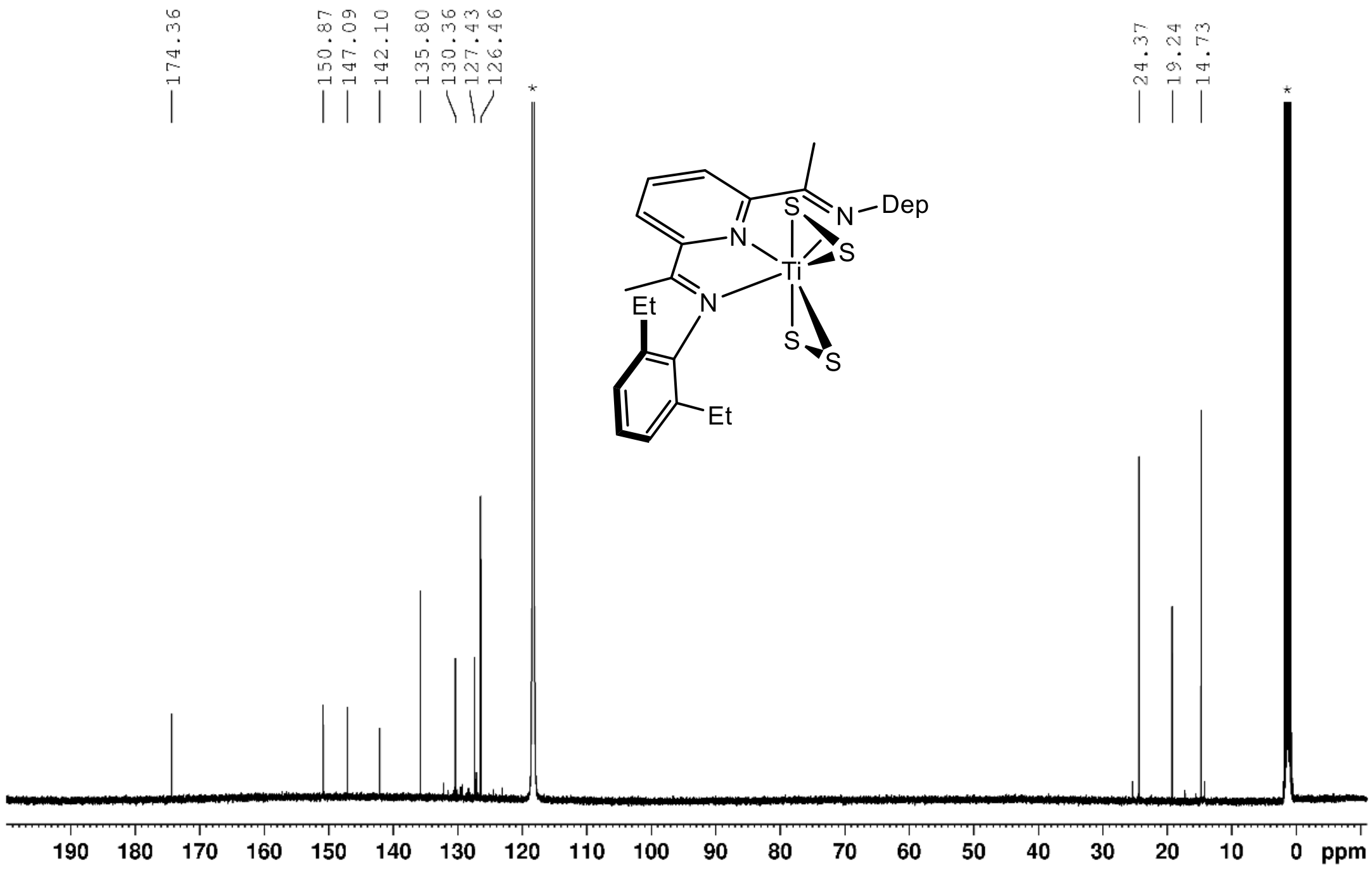

Figure S 4. ^{13}C NMR spectrum of ^{Et}PDITi$(S_2)_2$ (126 MHz, 298 K, acetonitrile-d_3). Residual signals of deuterated solvent are marked with an asterisk (*).

5 NMR Spectra of Isothiocyanates

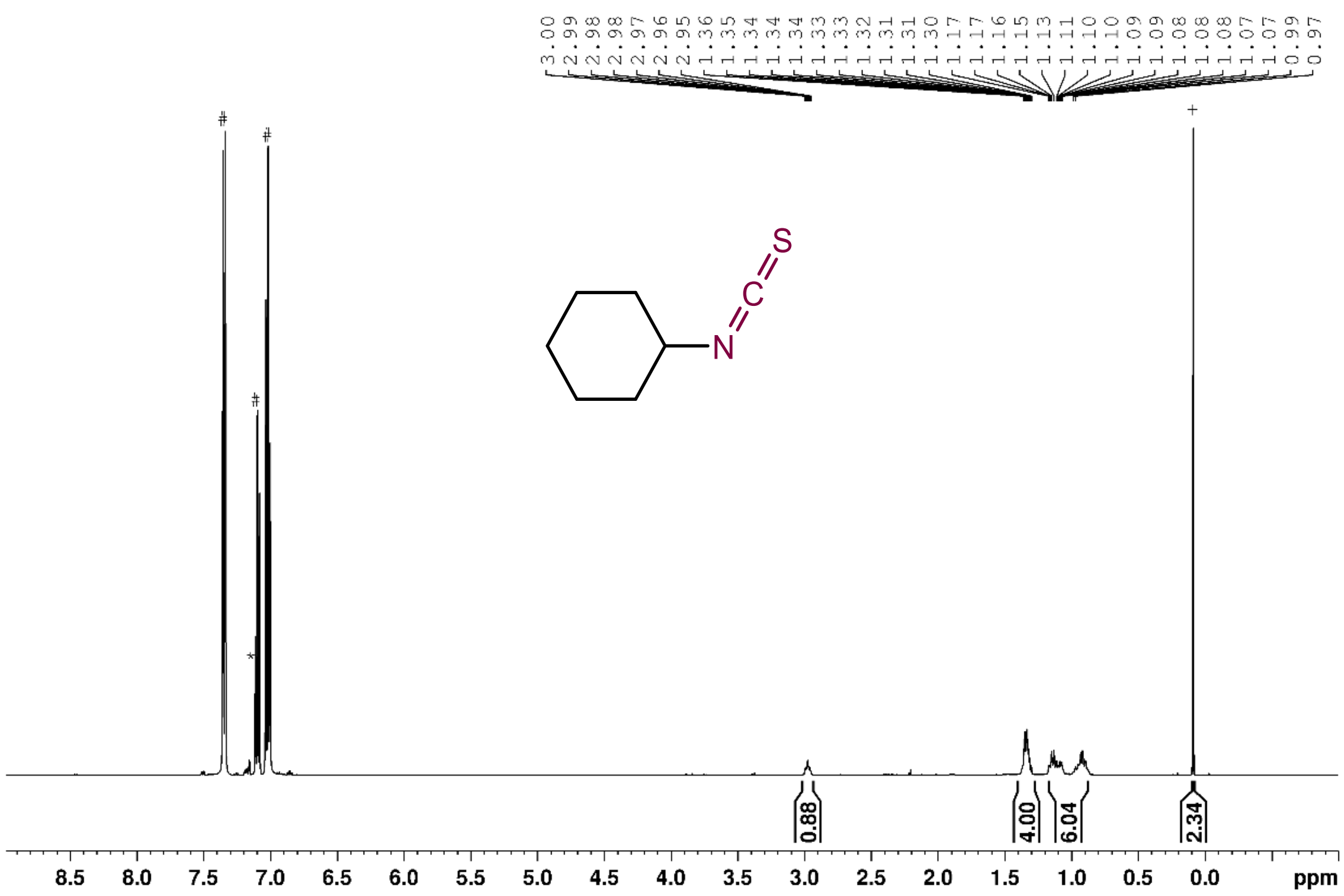

Figure S 5. ^{1}H NMR spectrum of Isothiocyanatocyclohexane (500 MHz, 298 K, benzene-d_6). Residual signals of deuterated solvent (*), TFT (#) and HMDSO (+) are marked.

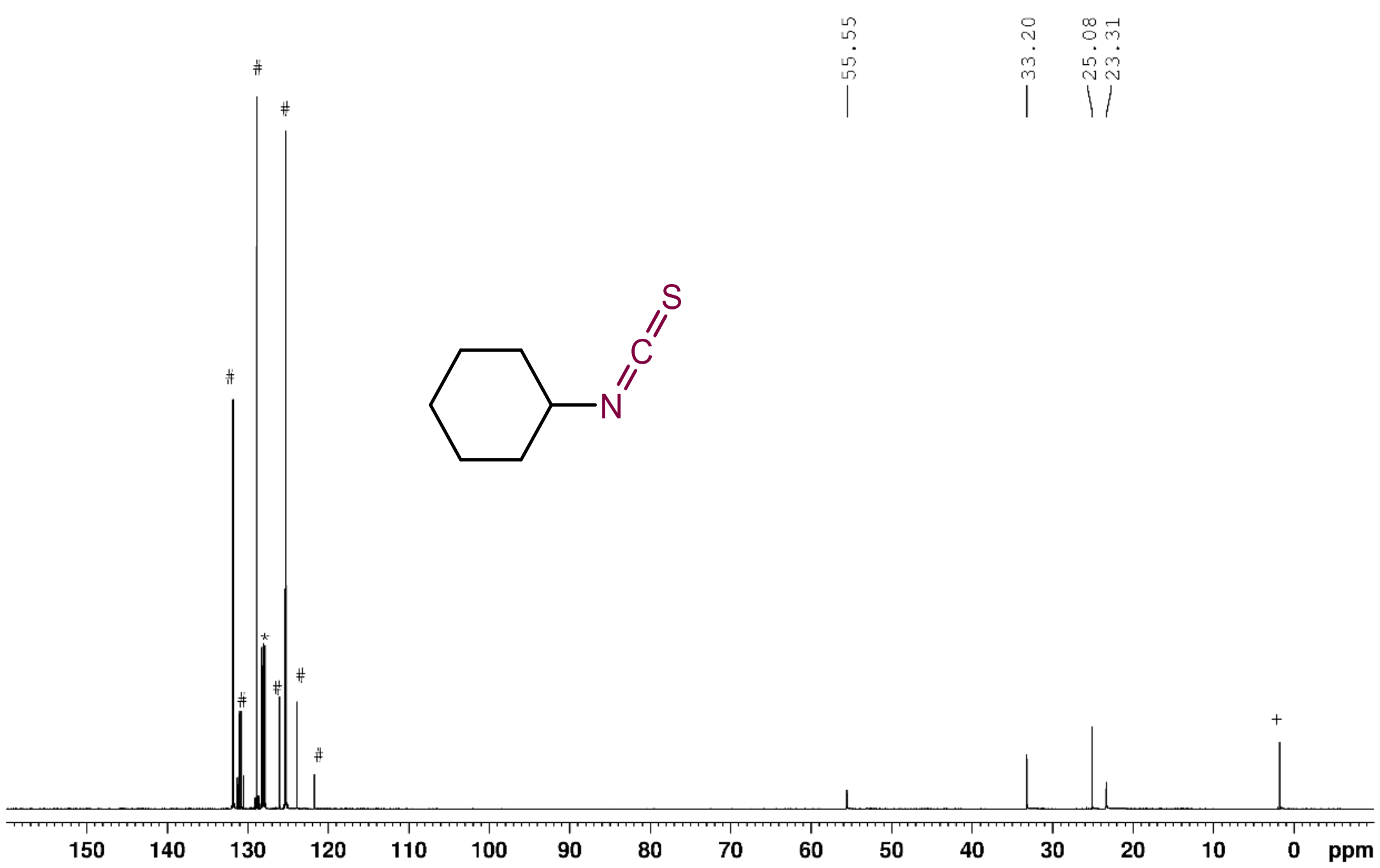

Figure S 6. ^{13}C NMR spectrum *of* Isothiocyanatocyclohexane (126 MHz, 298 K, benzene-d_6). Residual signals of deuterated solvent (*), TFT (#) and HMDSO (+) are marked.

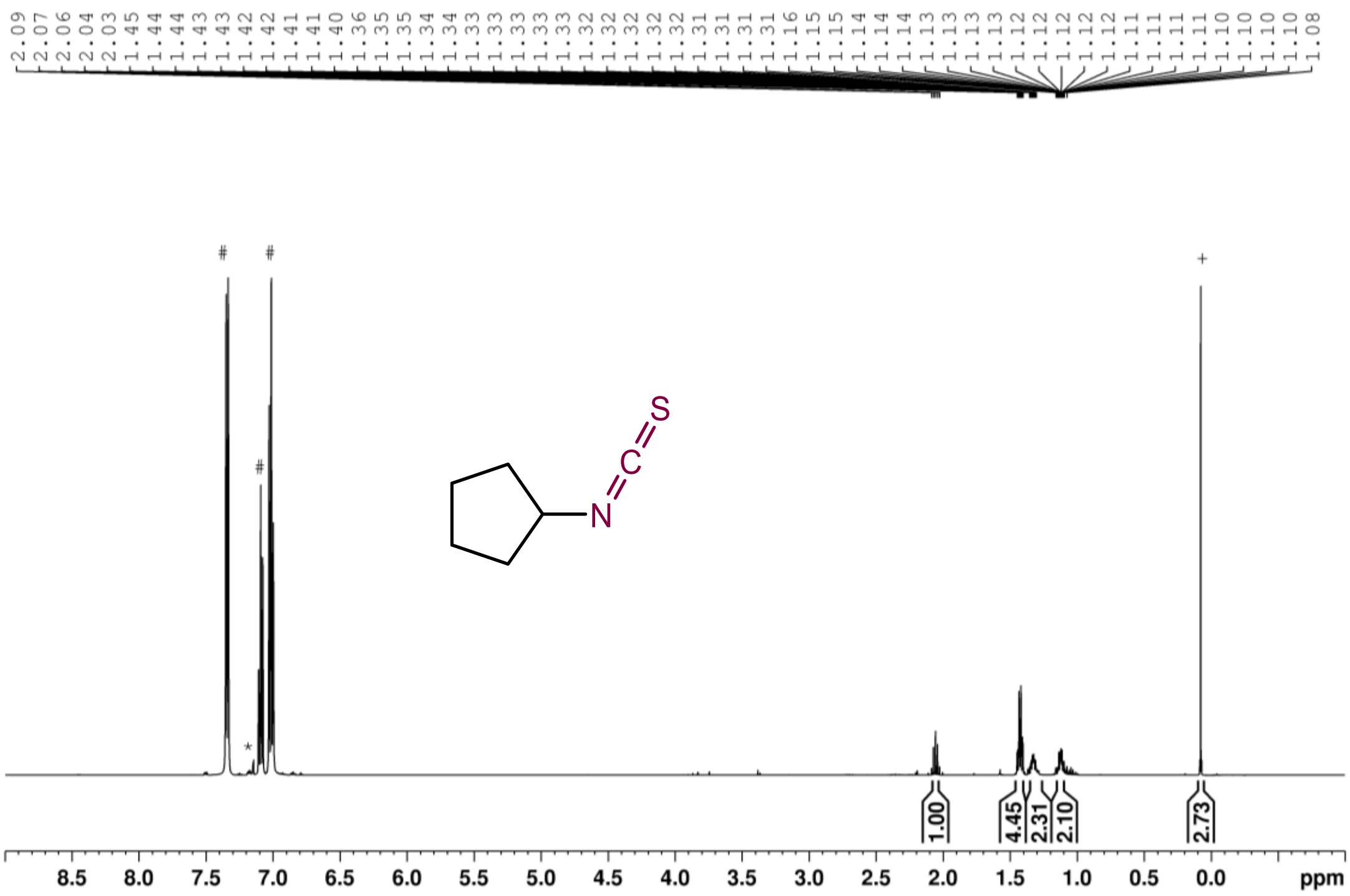

Figure S 7. ^{1}H NMR spectrum of Isothiocyanatocyclopentane (500 MHz, 298 K, benzene-d_6). Residual signals of deuterated solvent (*), TFT (#) and HMDSO (+) are marked.

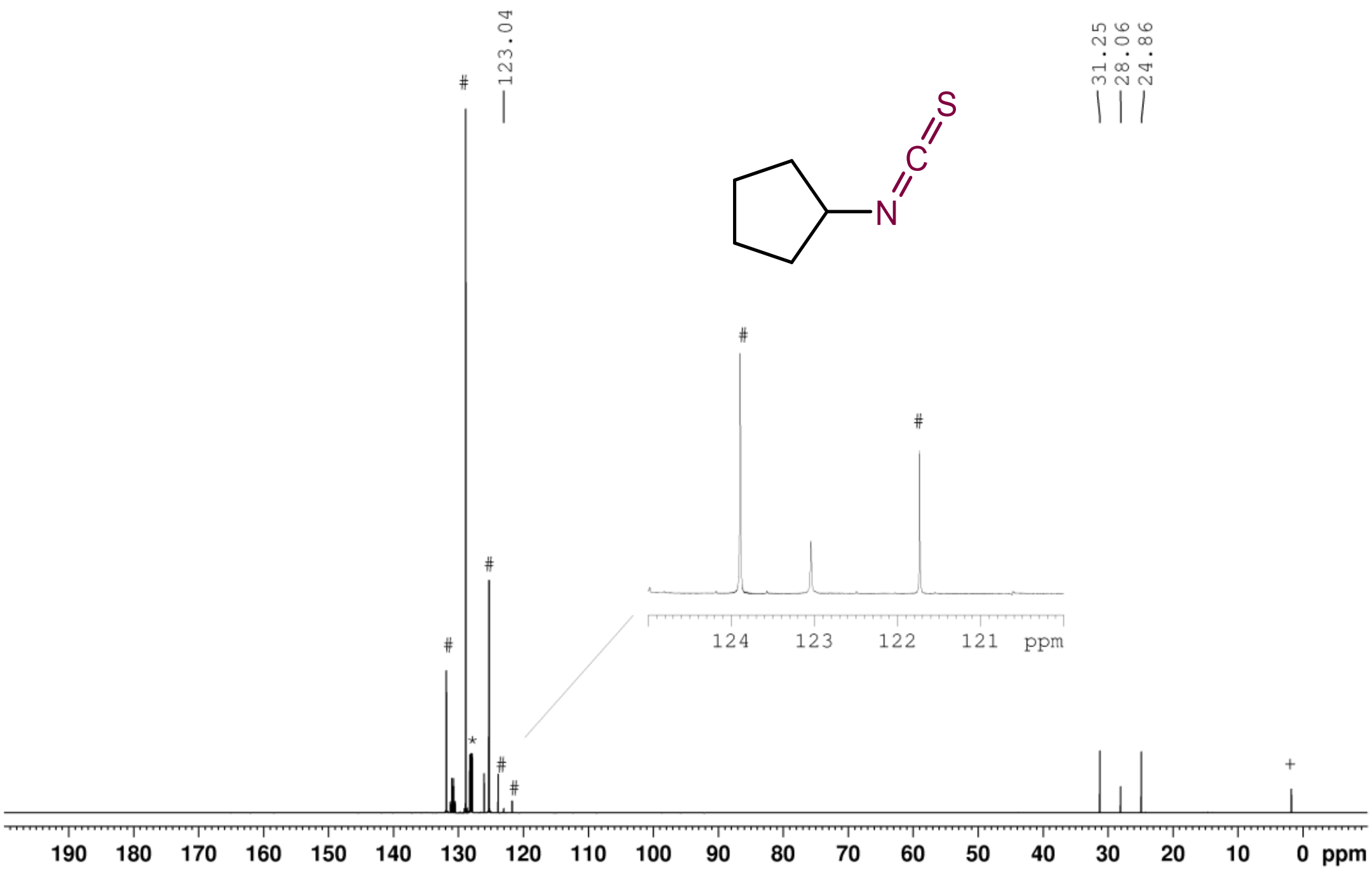

Figure S 8. ^{13}C NMR spectrum of Isothiocyanatocyclopentane (126 MHz, 298 K, benzene-d_6). Residual signals of deuterated solvent (*), TFT (#) and HMDSO (+) are marked.

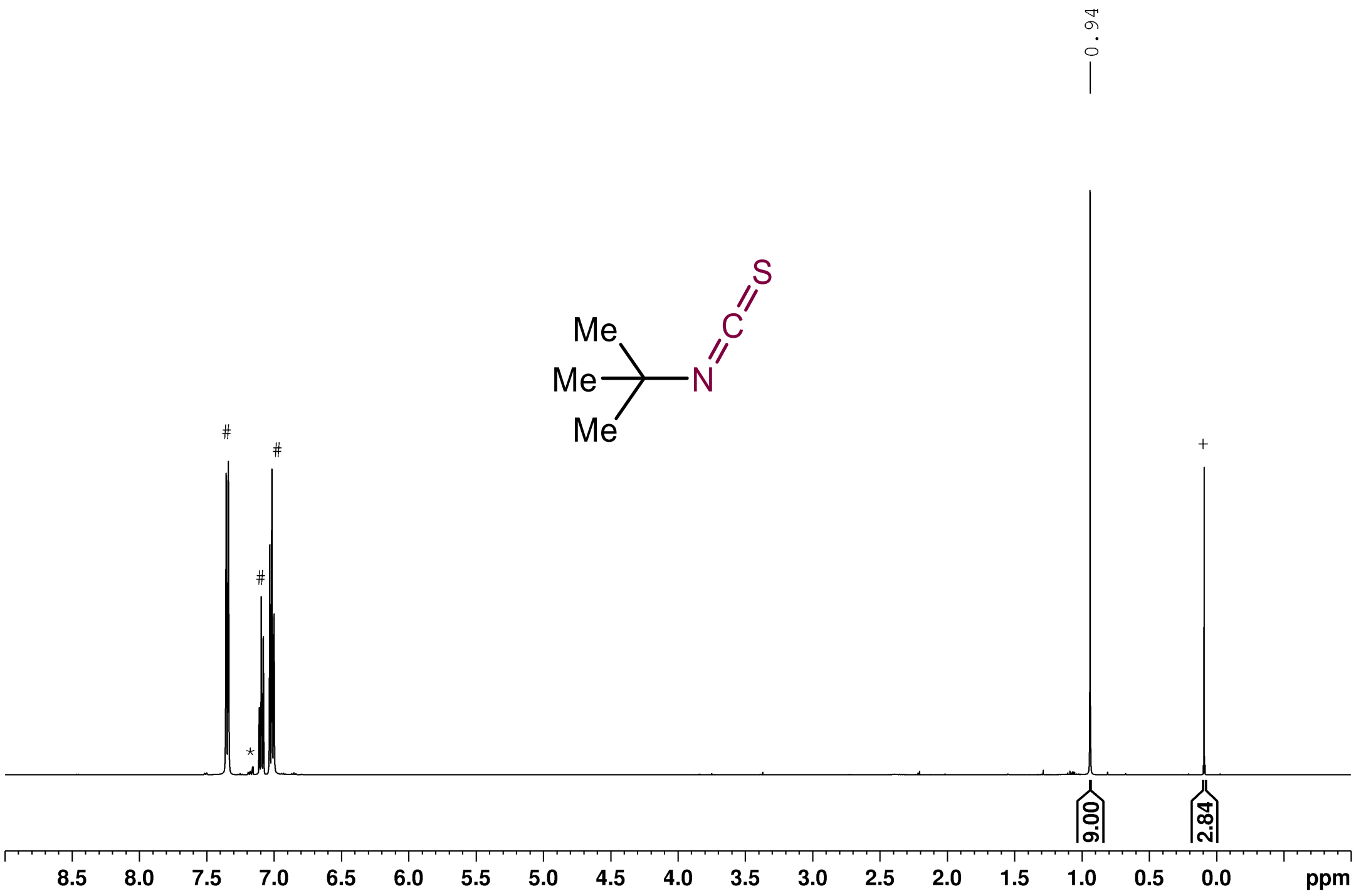

Figure S 9. ^{1}H NMR spectrum of 2-Isothiocyanato-2-methylpropane (500 MHz, 298 K, benzene-d_6). Residual signals of deuterated solvent (*), TFT (#) and HMDSO (+) are marked.

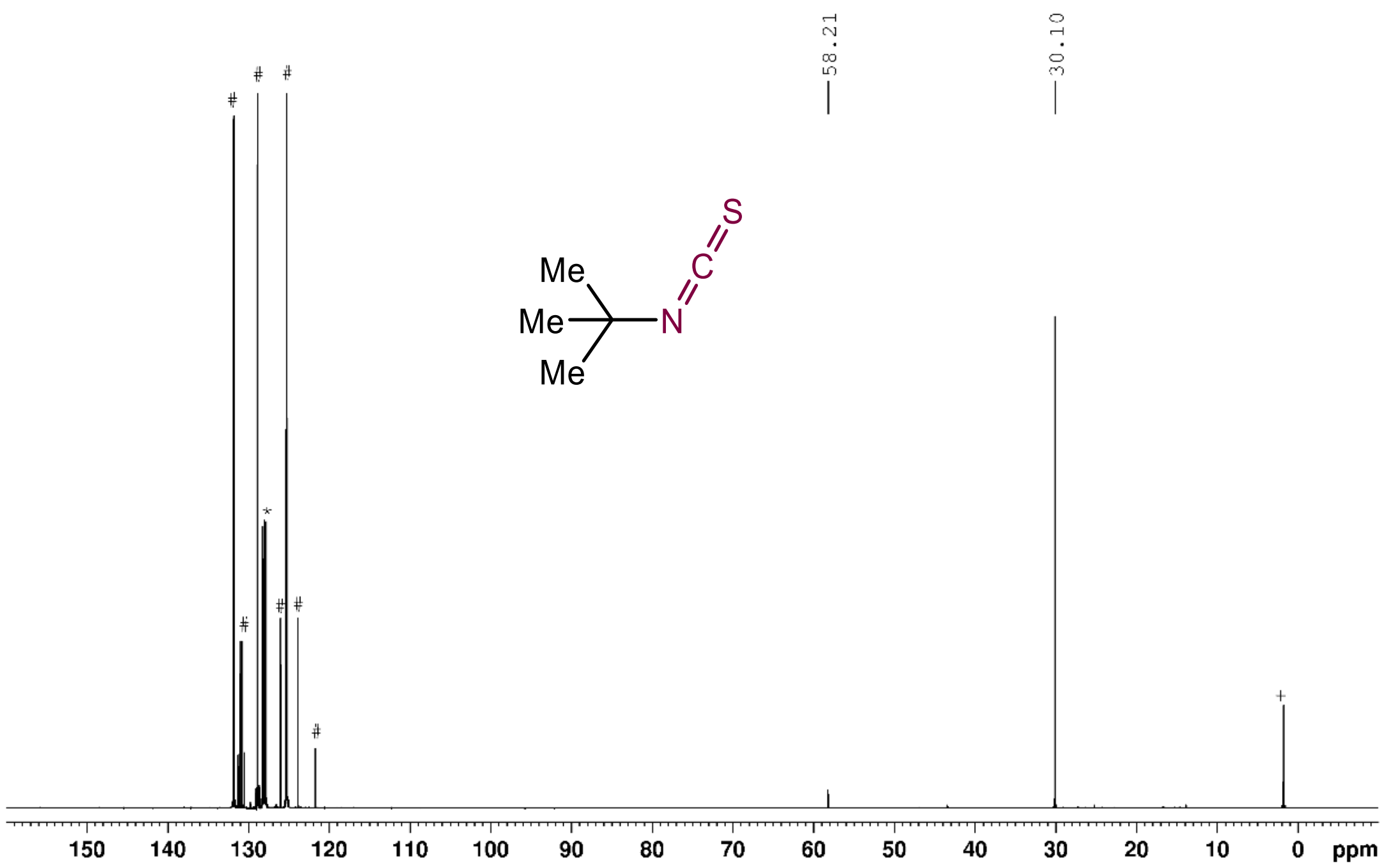

Figure S 10. ^{13}C NMR spectrum of 2-Isothiocyanato-2-methylpropane (126 MHz, 298 K, benzene-d_6). Residual signals of deuterated solvent (*), TFT (#) and HMDSO (+) are marked.

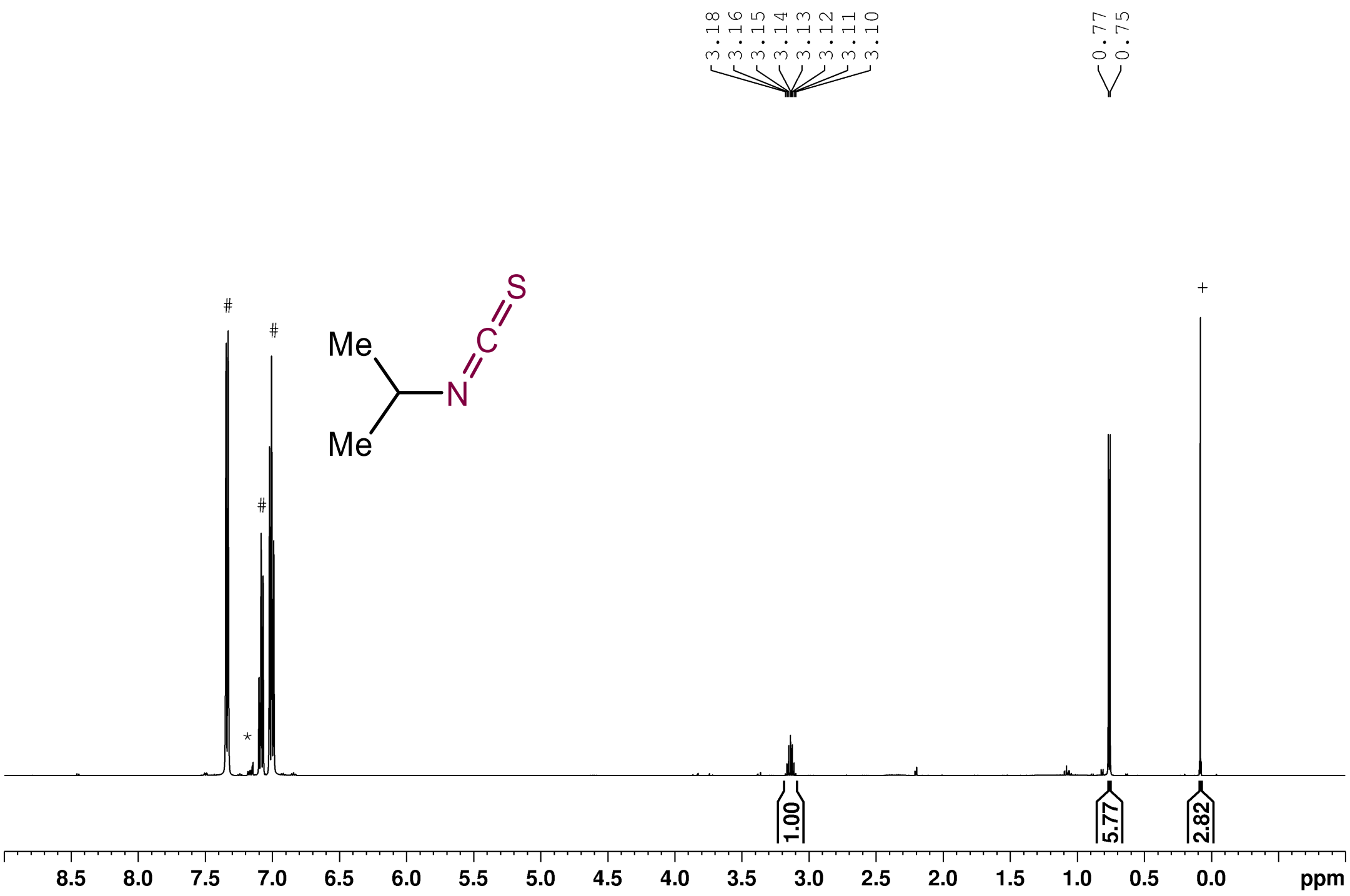

Figure S 11. ^{1}H NMR spectrum of 2-Isothiocyanatopropane (500 MHz, 298 K, benzene-d_6). Residual signals of deuterated solvent (*), TFT (#) and HMDSO (+) are marked.

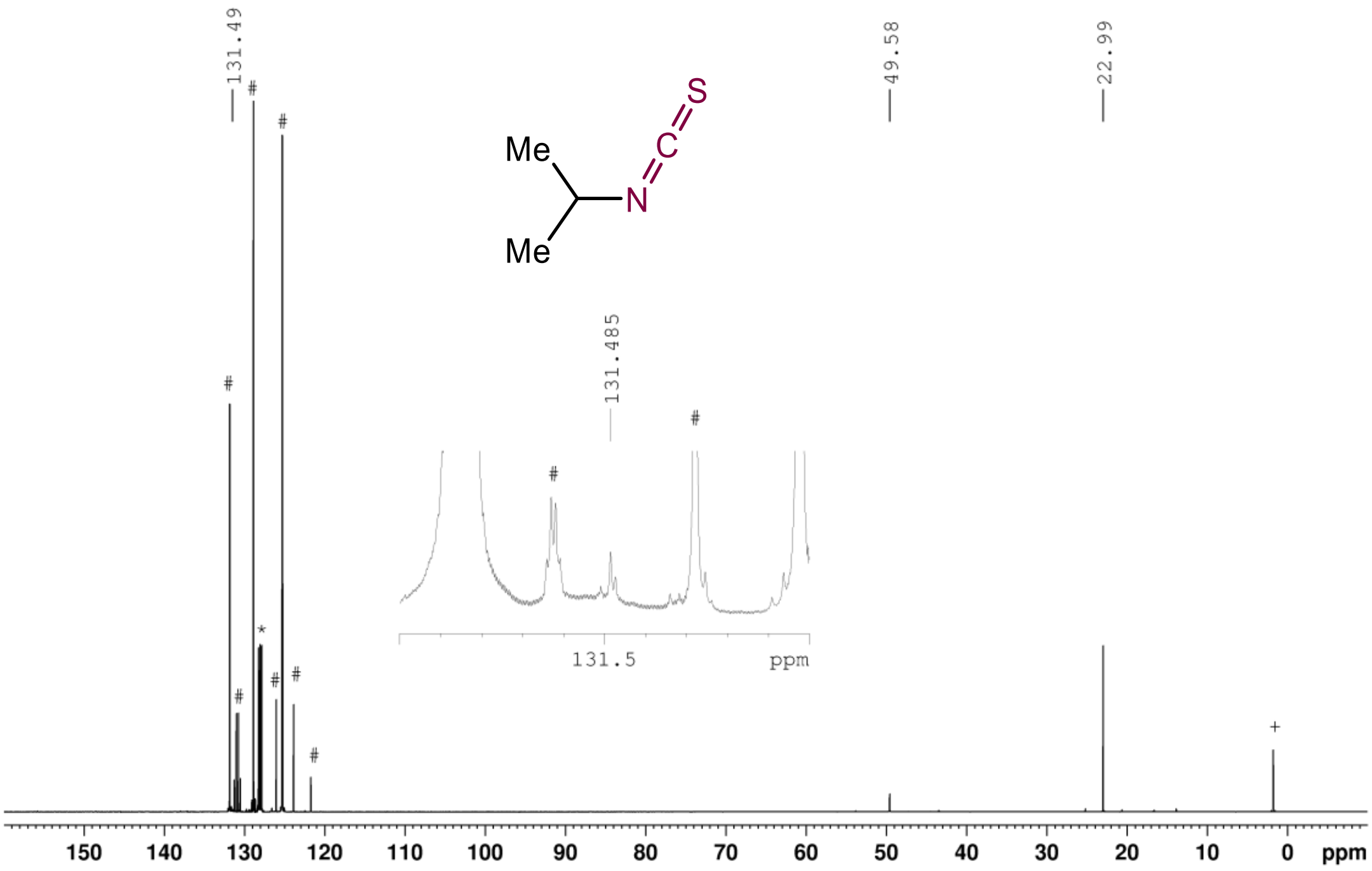

Figure S 12. ^{13}C NMR spectrum of 2-Isothiocyanatopropane (126 MHz, 298 K, benzene-d_6). Residual signals of deuterated solvent (*), TFT (#) and HMDSO (+) are marked.

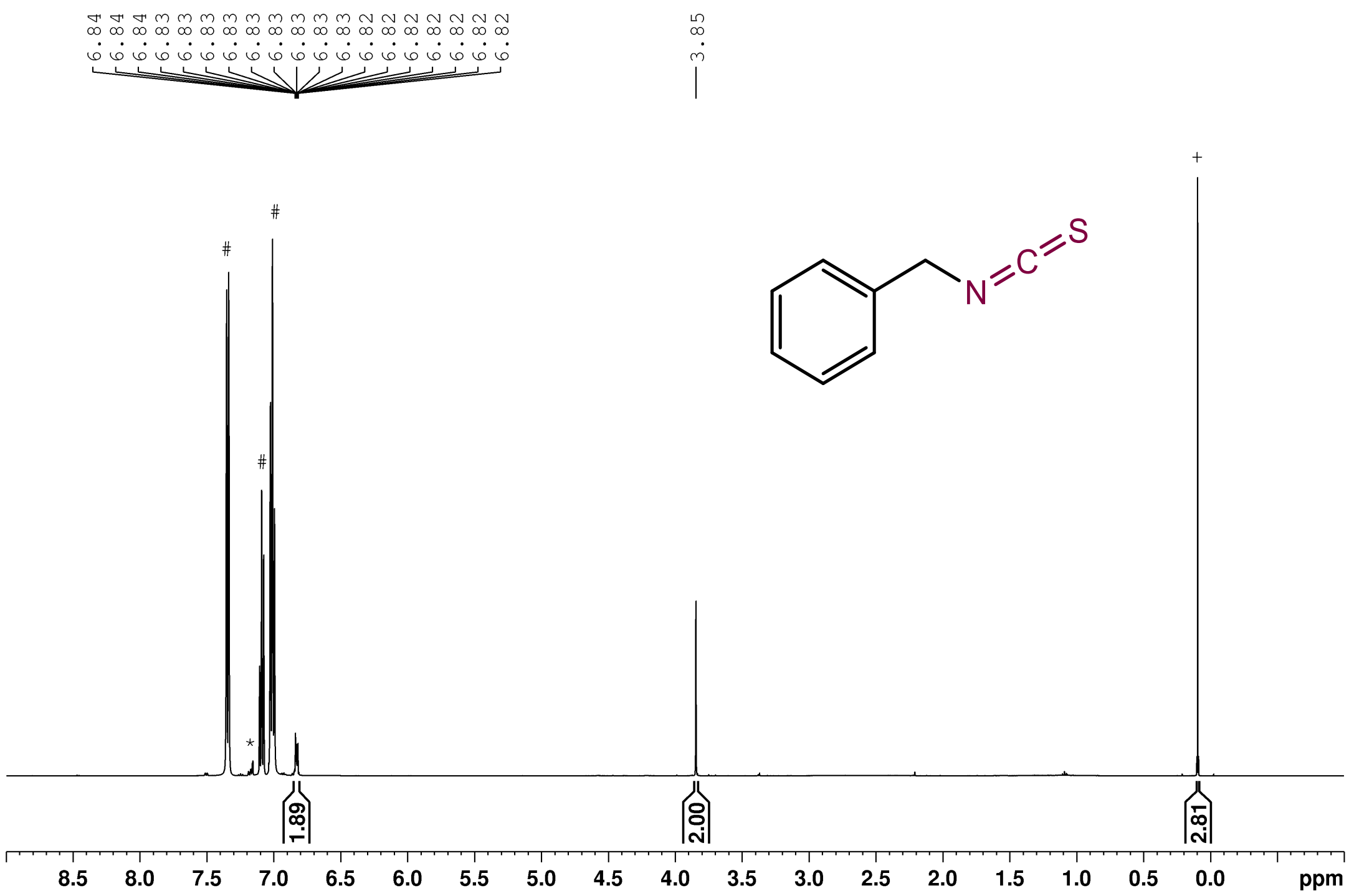

Figure S 13. ^{1}H NMR spectrum of (Isothiocyanatomethyl)benzene (500 MHz, 298 K, benzene-d_6). Residual signals of deuterated solvent (*), TFT (#) and HMDSO (+) are marked.

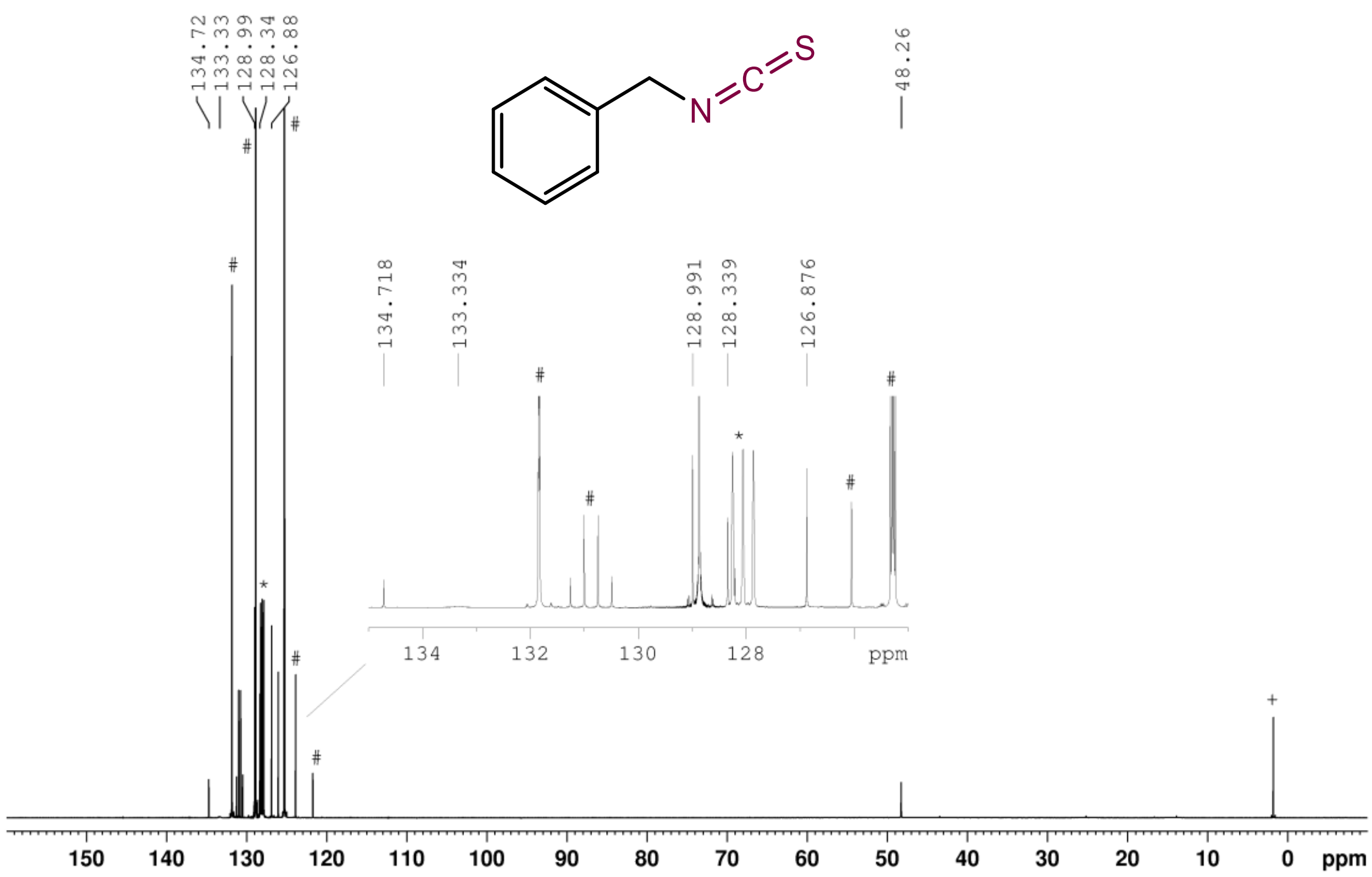

Figure S 14. ^{13}C NMR spectrum of (Isothiocyanatomethyl)benzene (126 MHz, 298 K, benzene-d_6). Residual signals of deuterated solvent (*), TFT (#) and HMDSO (+) are marked.

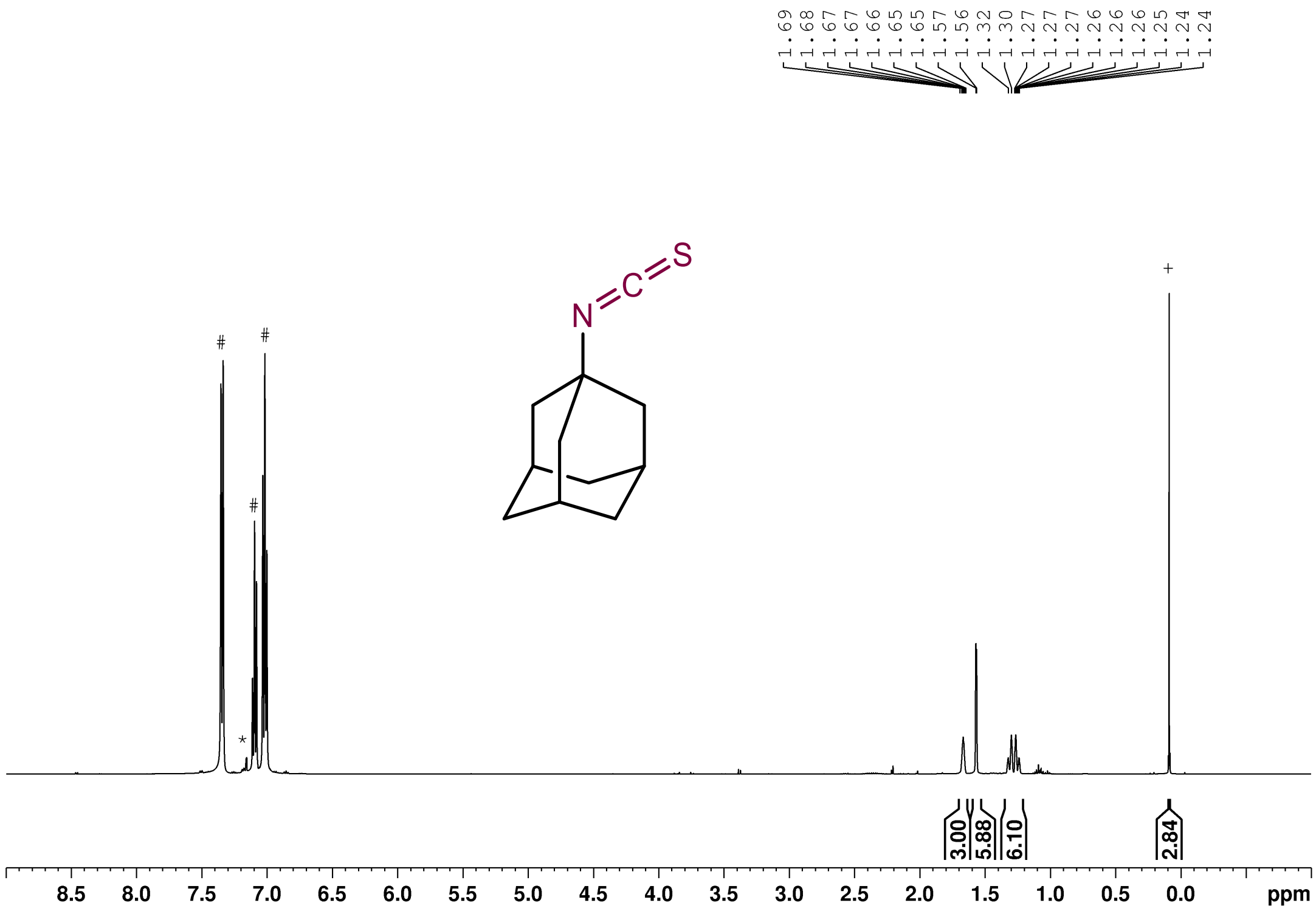

Figure S 15. ^{1}H NMR spectrum of (3s,5s,7s)-1-Isothiocyanatoadamantane (500 MHz, 298 K, benzene-d_6). Residual signals of deuterated solvent (*), TFT (#) and HMDSO (+) are marked.

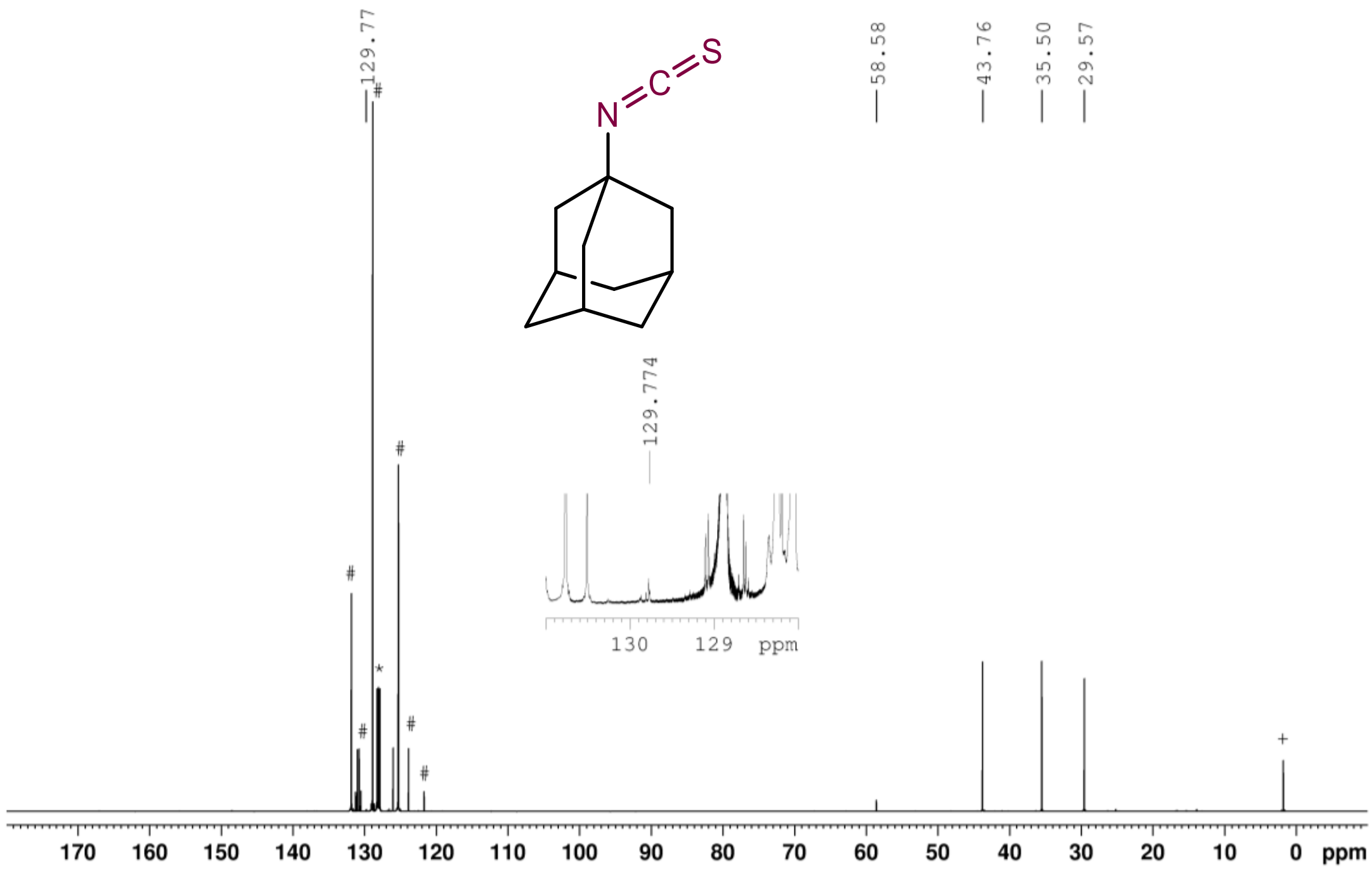

Figure S 16. ^{13}C NMR spectrum of (3s,5s,7s)-1-Isothiocyanatoadamantane (126 MHz, 298 K, benzene-d_6). Residual signals of deuterated solvent (*), TFT (#) and HMDSO (+) are marked.

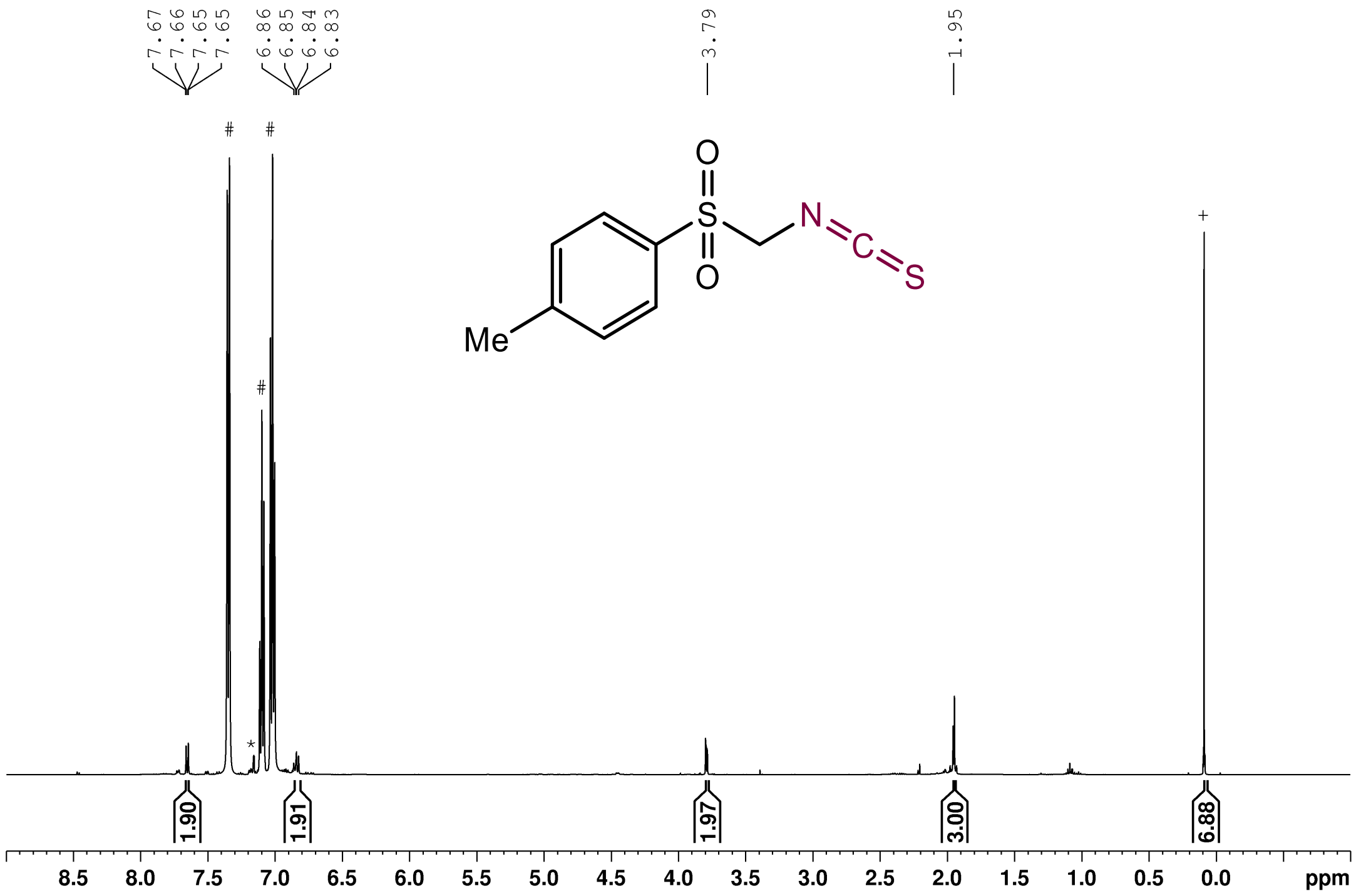

Figure S 17. ^{1}H NMR spectrum of 1-((Isothiocyanatomethyl)sulfonyl)-4-methylbenzene (500 MHz, 298 K, benzene-*d*6). Residual signals of deuterated solvent (*), TFT (#) and HMDSO (+) are marked.

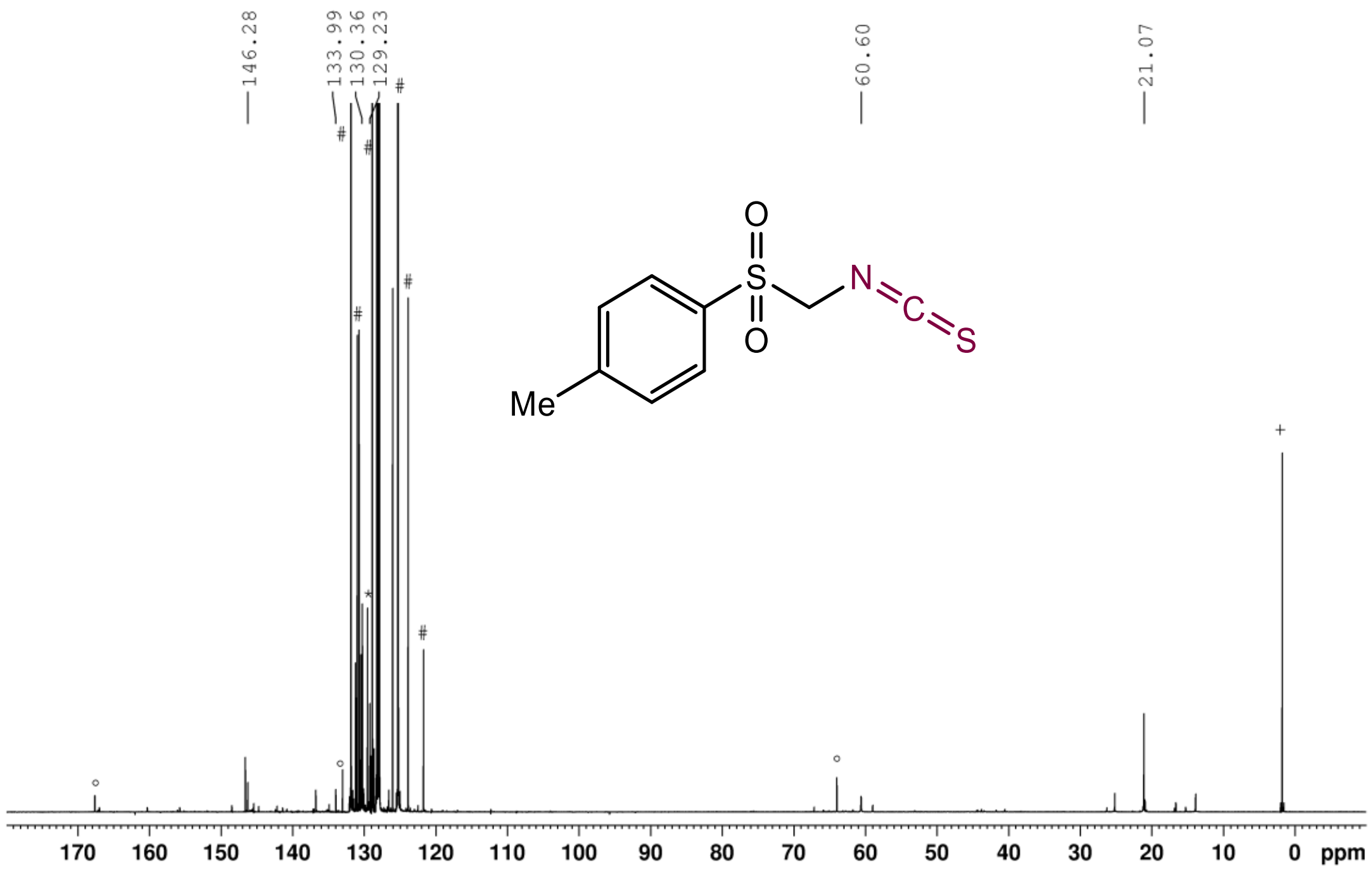

Figure S 18. ^{13}C NMR spectrum of 1-((Isothiocyanatomethyl)sulfonyl)-4-methylbenzene (126 MHz, 298 K, benzene-*d*6). Residual signals of deuterated solvent (*), TFT (#), HMDSO (+) and starting material (°) are marked.

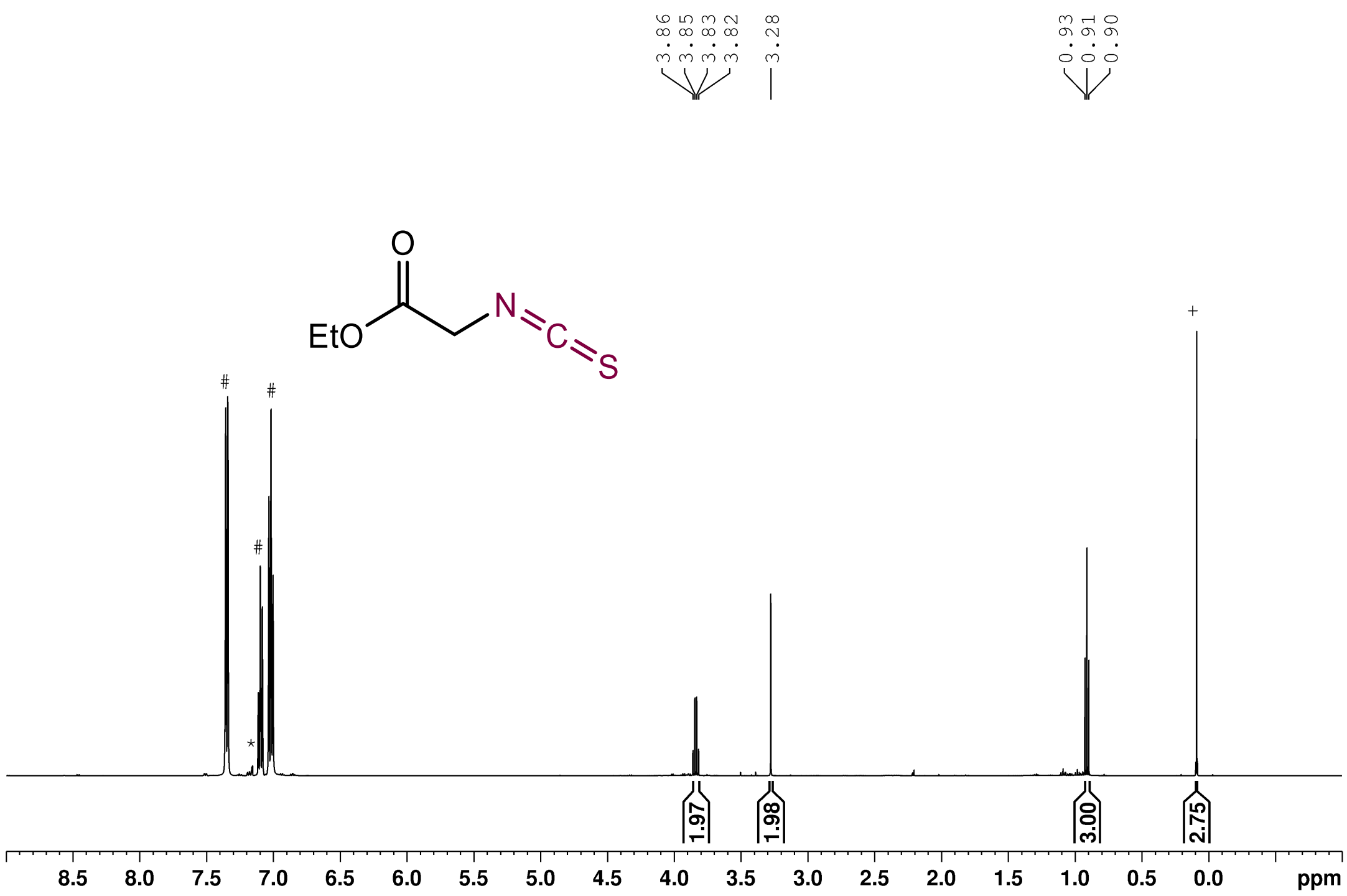

Figure S 19. ^{1}H NMR spectrum of Ethyl 2-isothiocyanatoacetate (500 MHz, 298 K, benzene-d_6). Residual signals of deuterated solvent (*), TFT (#) and HMDSO (+) are marked.

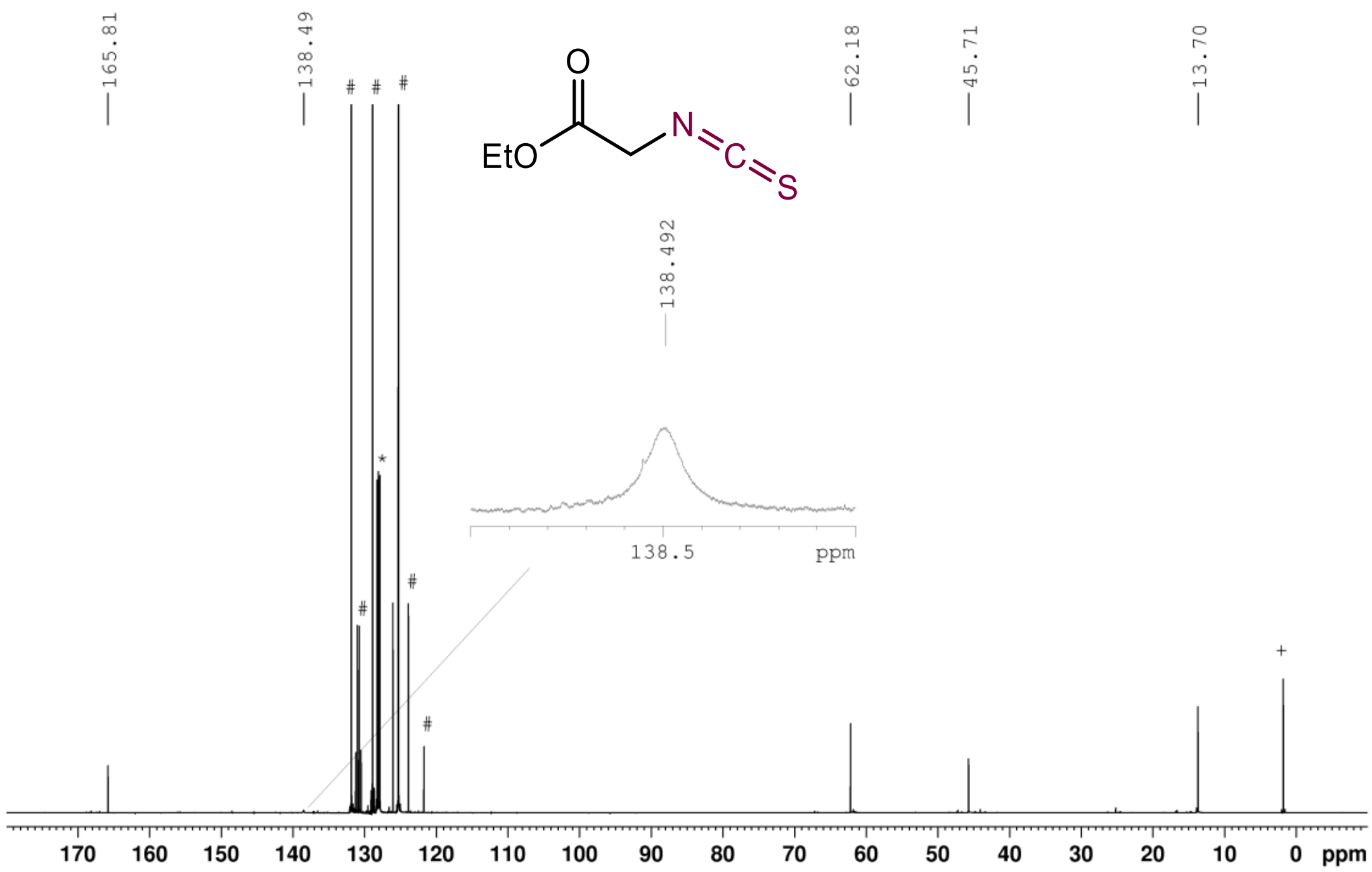

Figure S 20. ^{13}C NMR spectrum of Ethyl 2-isothiocyanatoacetate (126 MHz, 298 K, benzene-d_6). Residual signals of deuterated solvent (*), TFT (#) and HMDSO (+) are marked.

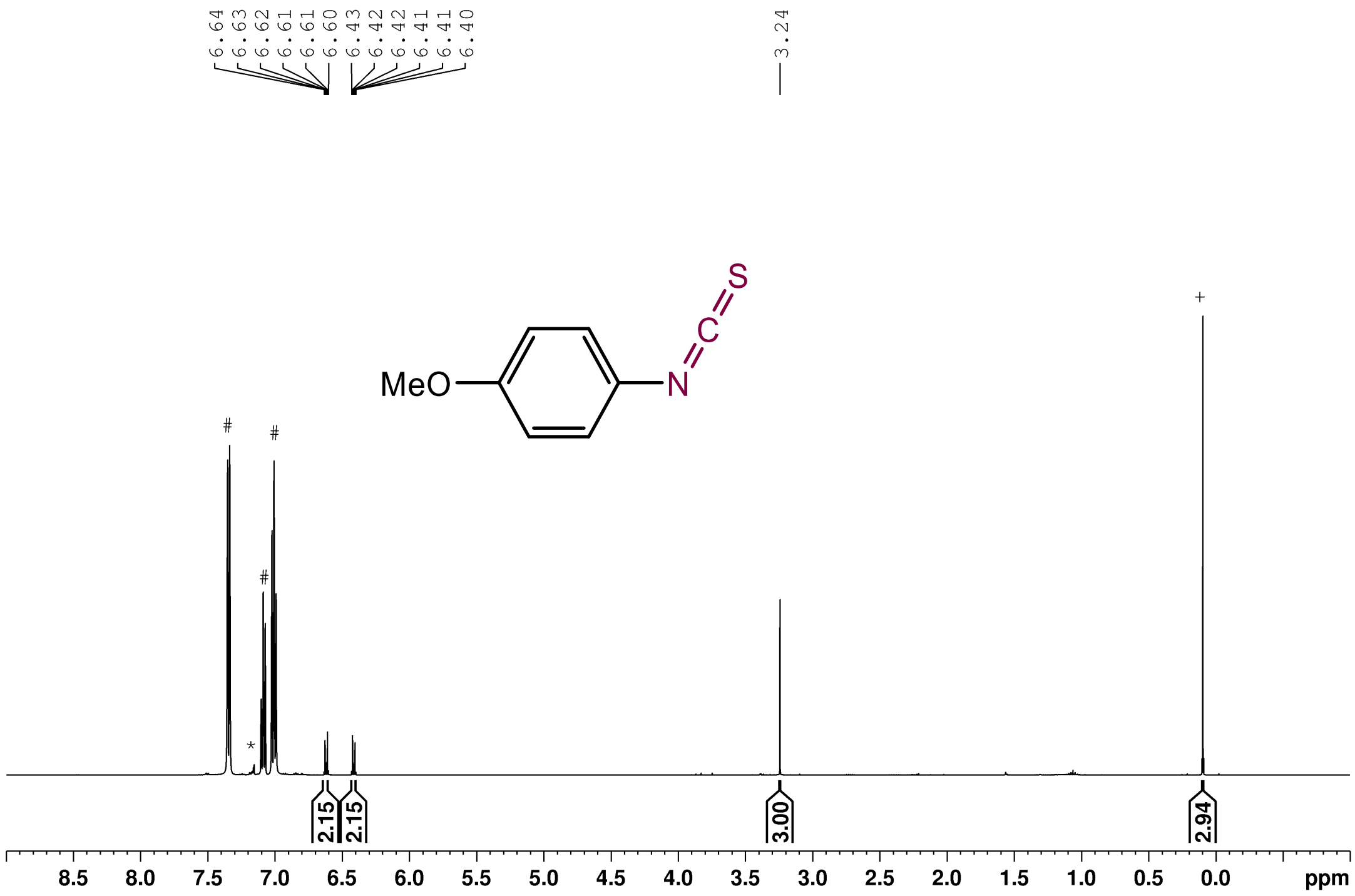

Figure S 21. ^{1}H NMR spectrum of 1-Isothiocyanato-4-methoxybenzene (500 MHz, 298 K, benzene-d_6). Residual signals of deuterated solvent (*), TFT (#) and HMDSO (+) are marked.

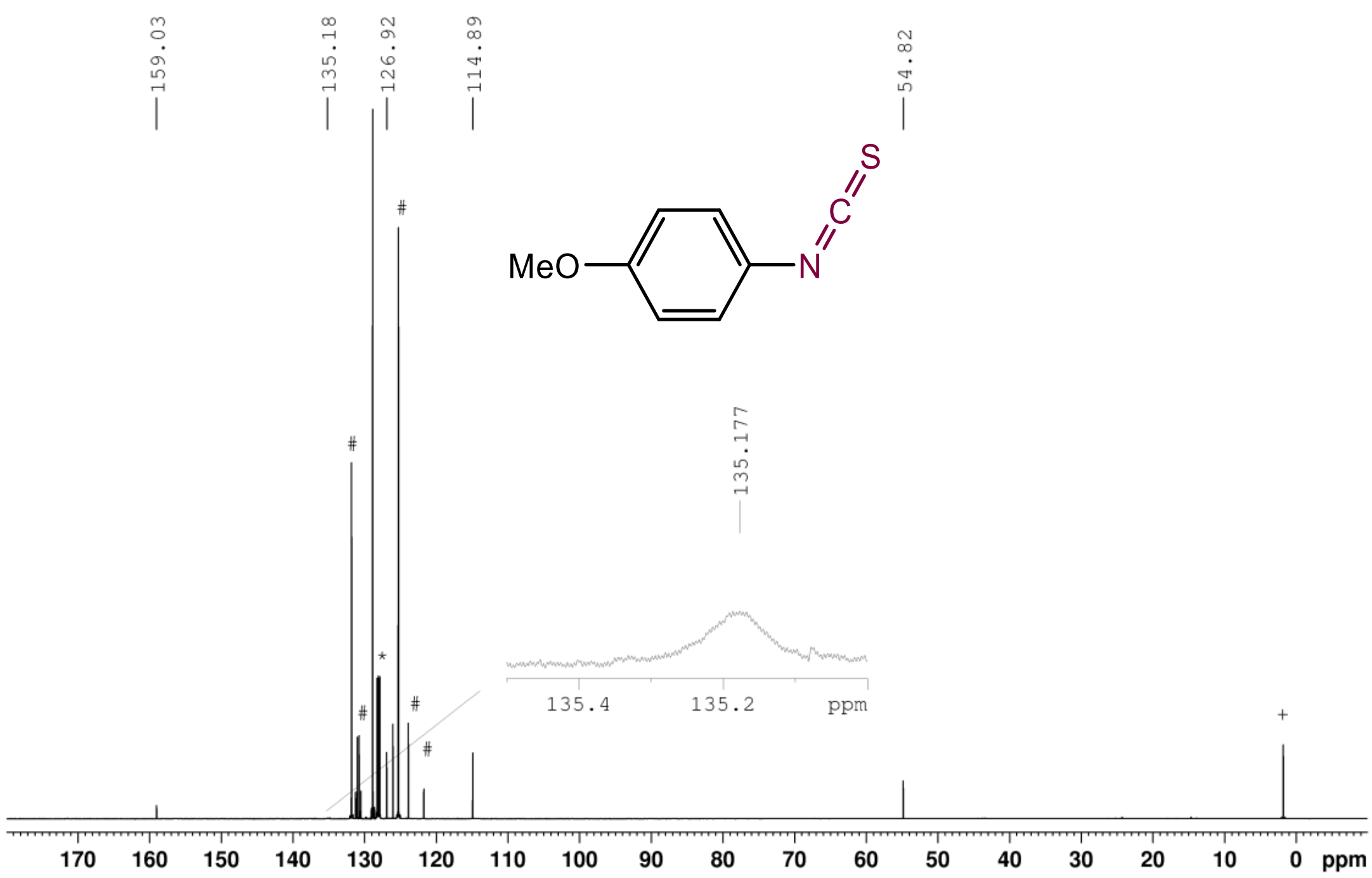

Figure S 22. ^{13}C NMR spectrum of 1-Isothiocyanato-4-methoxybenzene (126 MHz, 298 K, benzene-d_6). Residual signals of deuterated solvent (*), TFT (#) and HMDSO (+) are marked.

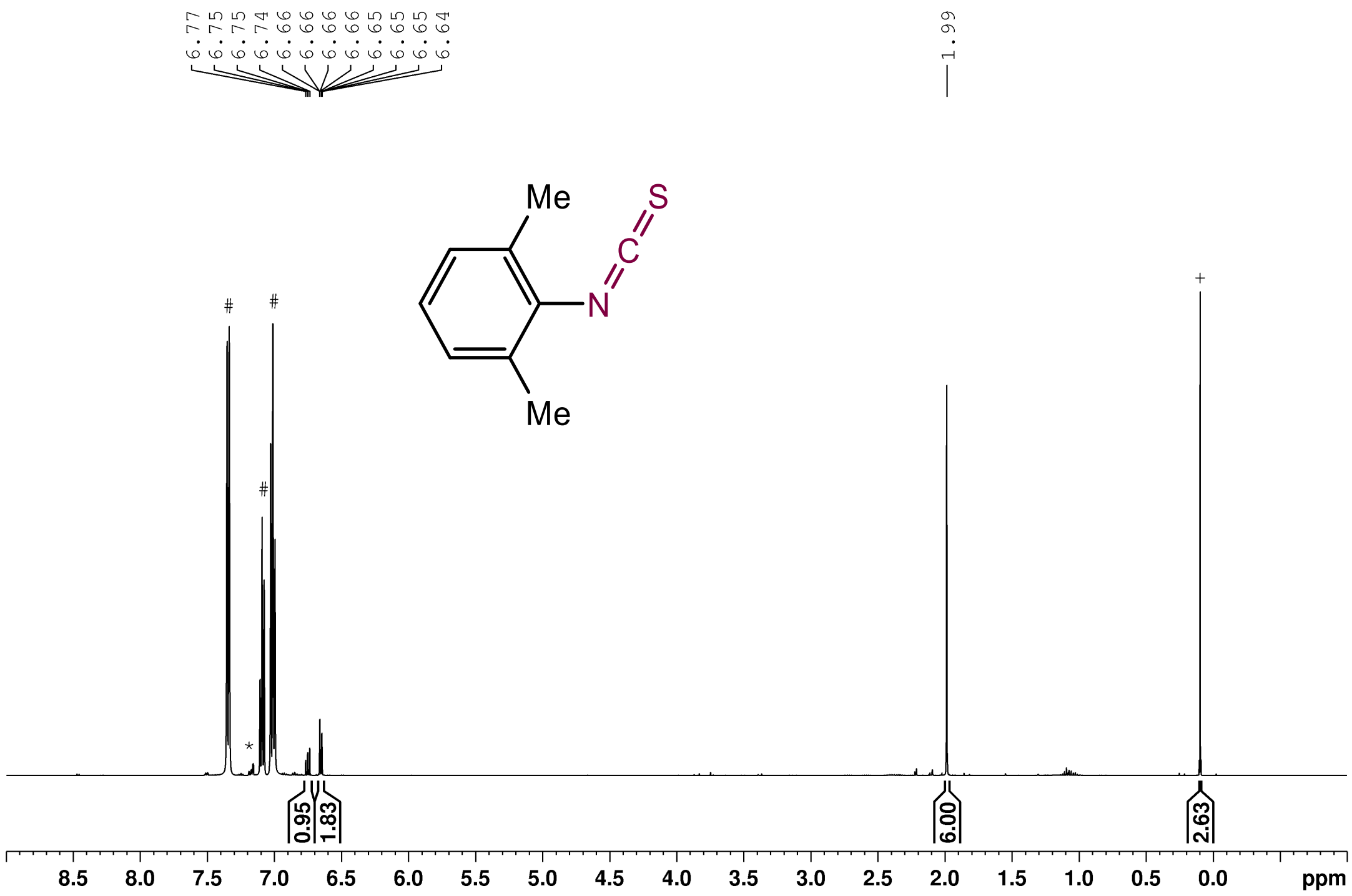

Figure S 23. ^{1}H NMR spectrum of 2-Isothiocyanato-1,3-dimethylbenzene (500 MHz, 298 K, benzene-d_6). Residual signals of deuterated solvent (*), TFT (#) and HMDSO (+) are marked.

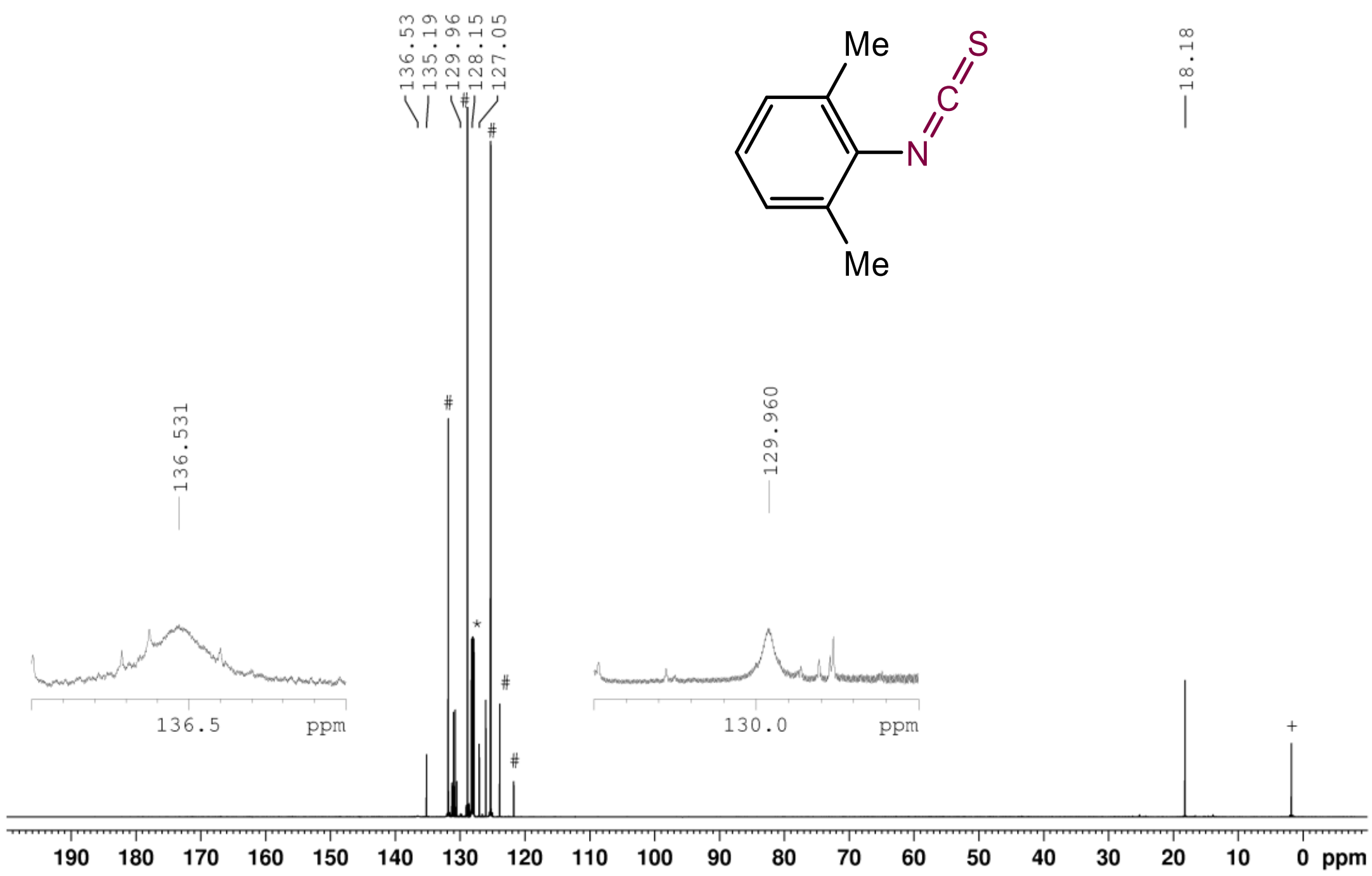

Figure S 24. ^{13}C NMR spectrum of 2-Isothiocyanato-1,3-dimethylbenzene (126 MHz, 298 K, benzene-d_6). Residual signals of deuterated solvent (*), TFT (#) and HMDSO (+) are marked.

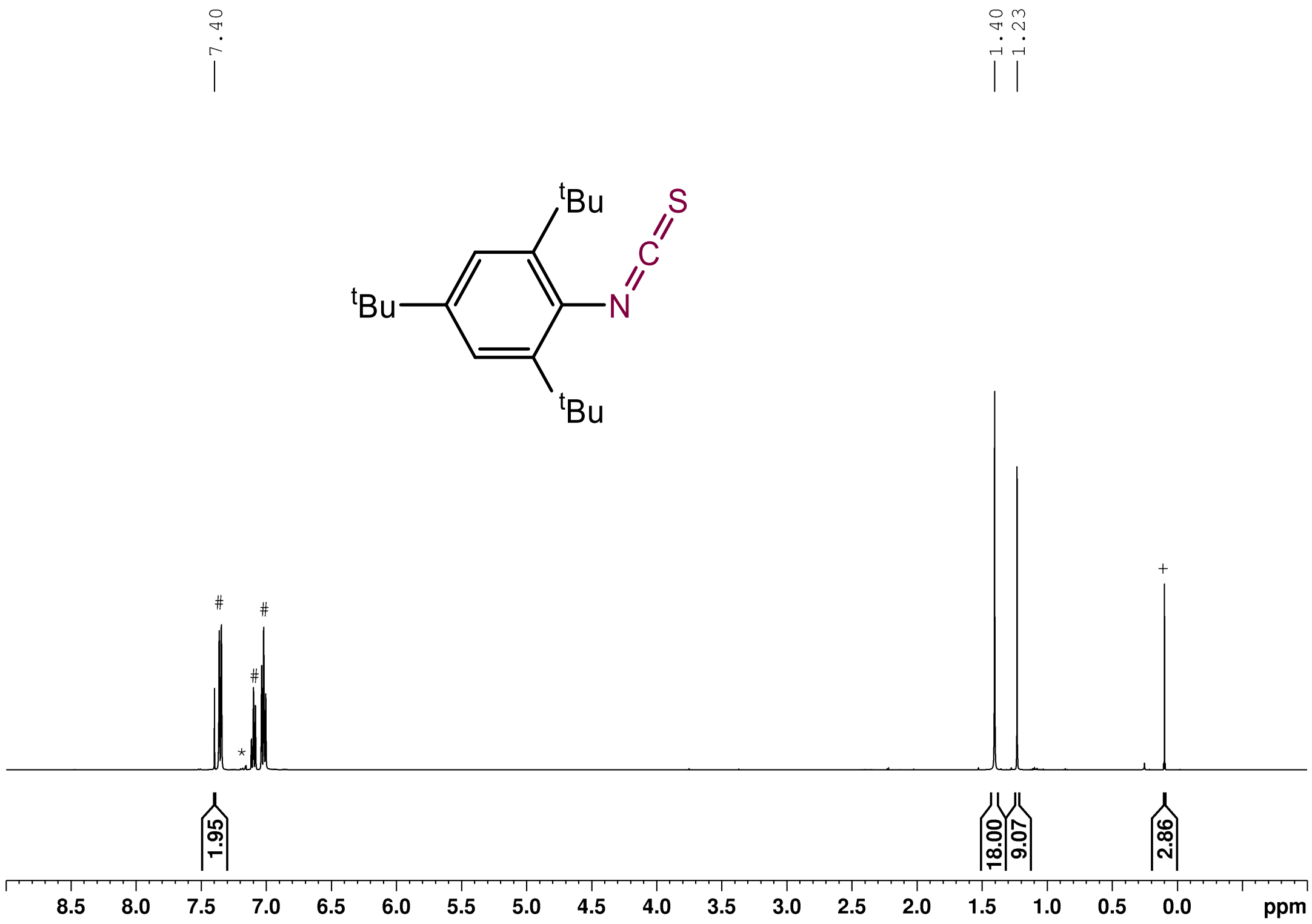

Figure S 25. ^{1}H NMR spectrum of 1,3,5-tri-*tert*-Butyl-2-isothiocyanatobenzene (500 MHz, 298 K, benzene-d_6). Residual signals of deuterated solvent (*), TFT (#) and HMDSO (+) are marked.

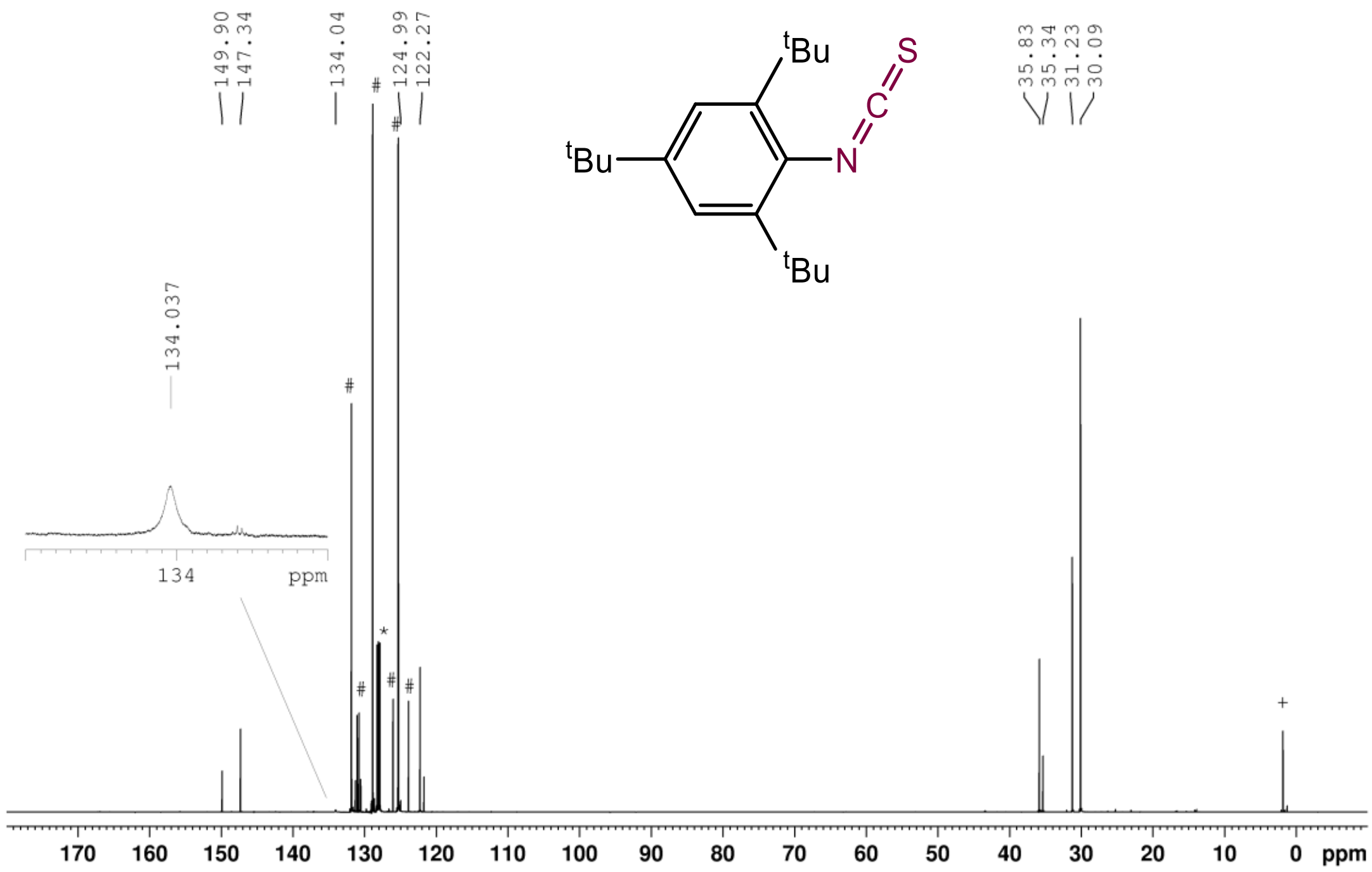

Figure S 26. ^{13}C NMR spectrum of 1,3,5-tri-*tert*-Butyl-2-isothiocyanatobenzene (126 MHz, 298 K, benzene-d_6). Residual signals of deuterated solvent (*), TFT (#) and HMDSO (+) are marked.

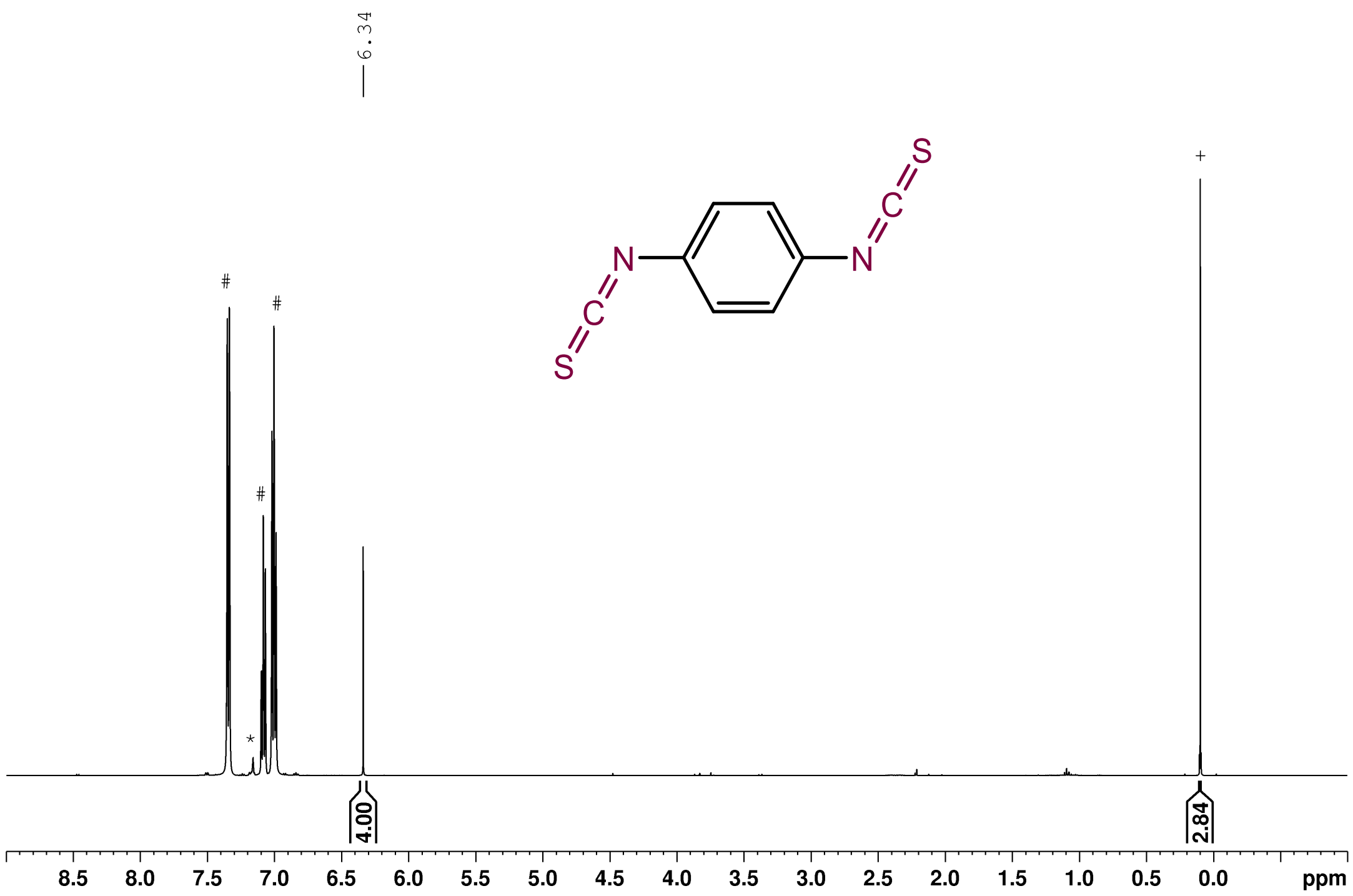

Figure S 27. ^{1}H NMR spectrum of 1,4-Diisothiocyanatobenzene (500 MHz, 298 K, benzene-d_6). Residual signals of deuterated solvent (*), TFT (#) and HMDSO (+) are marked.

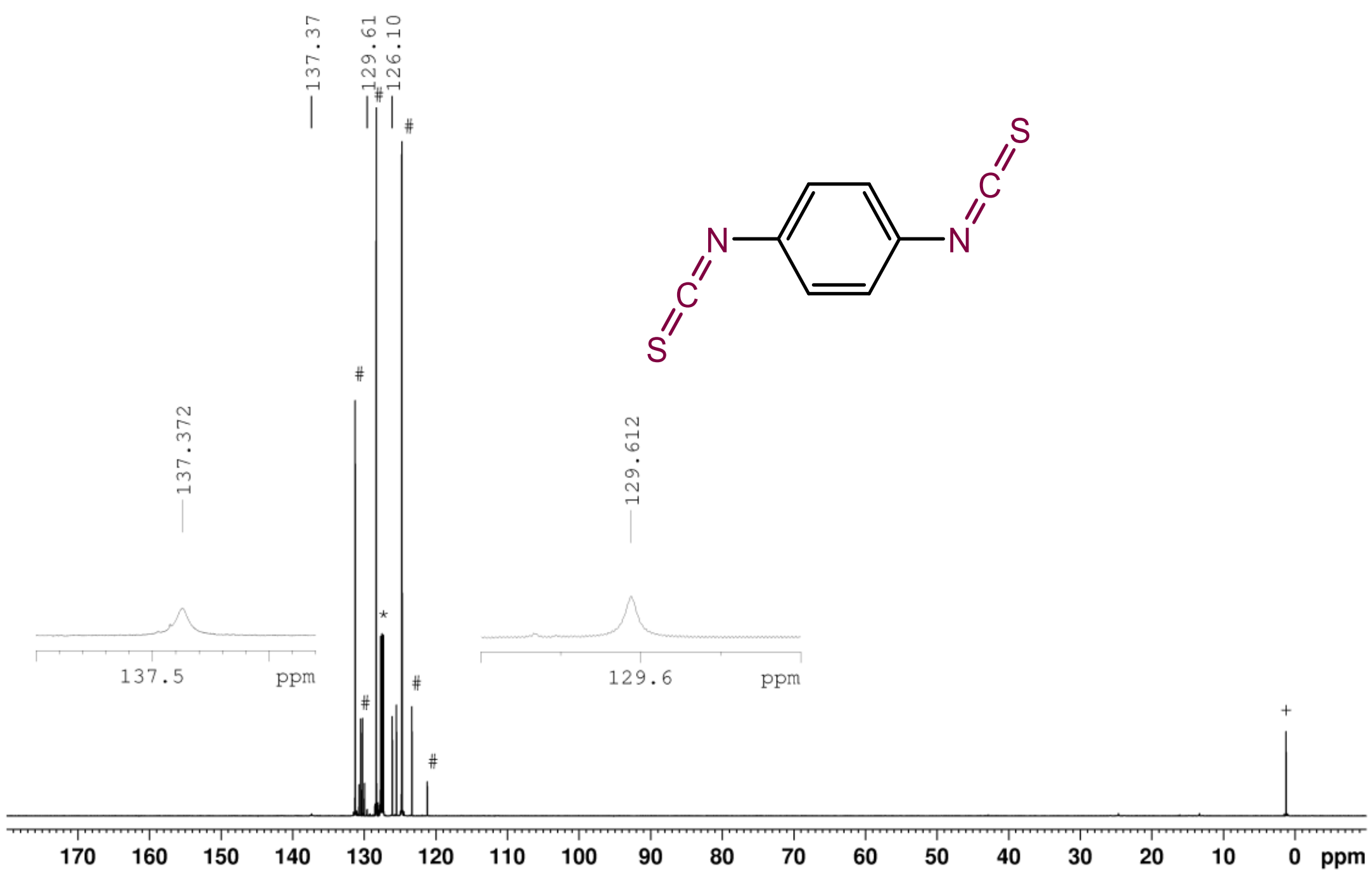

Figure S 28. ^{13}C NMR spectrum of 1,4-Diisothiocyanatobenzene (126 MHz, 298 K, benzene-d_6). Residual signals of deuterated solvent (*), TFT (#) and HMDSO (+) are marked.

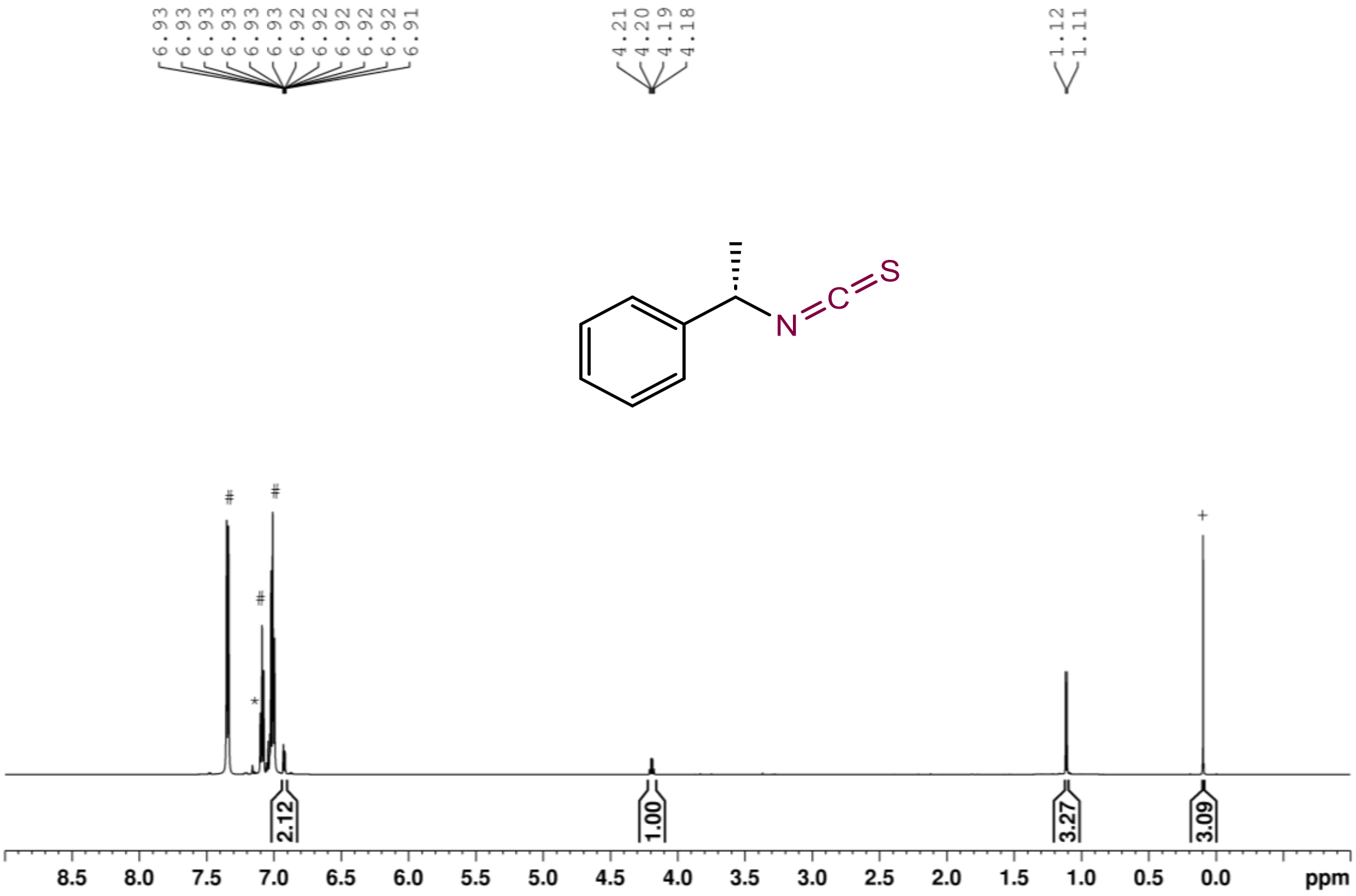

Figure S 29: ^{1}H NMR spectrum of (*S*)-(-)-α-Methylbenzylisothiocyanate (600 MHz, 298 K, benzene-d_6). Residual signals of deuterated solvent (*), TFT (#) and HMDSO (+) are marked.

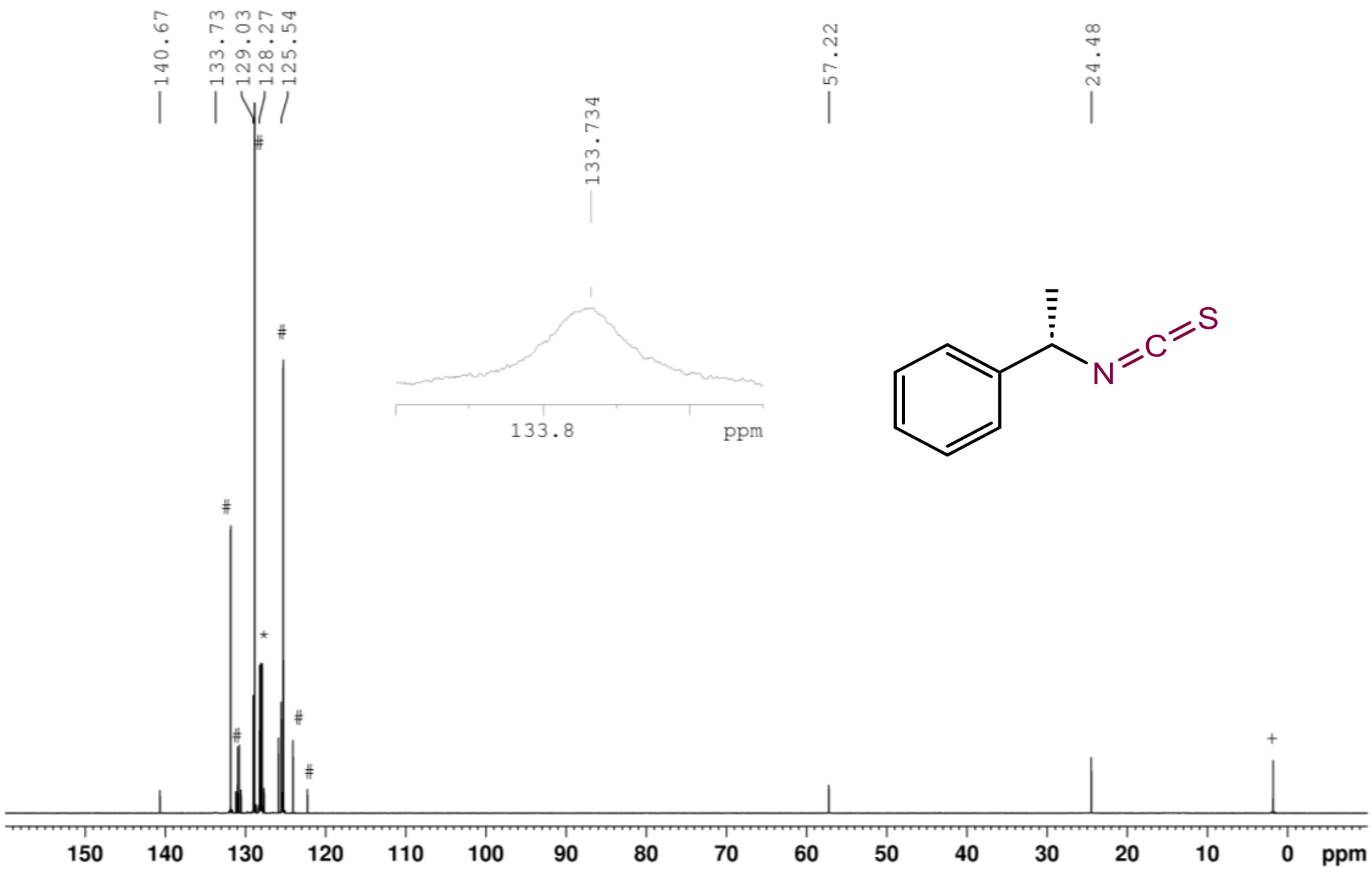

Figure S 30: ^{13}C NMR spectrum of (*S*)-(-)-α-Methylbenzylisothiocyanate (151 MHz, 298 K, benzene-d_6). Residual signals of deuterated solvent (*), TFT (#) and HMDSO (+) are marked.

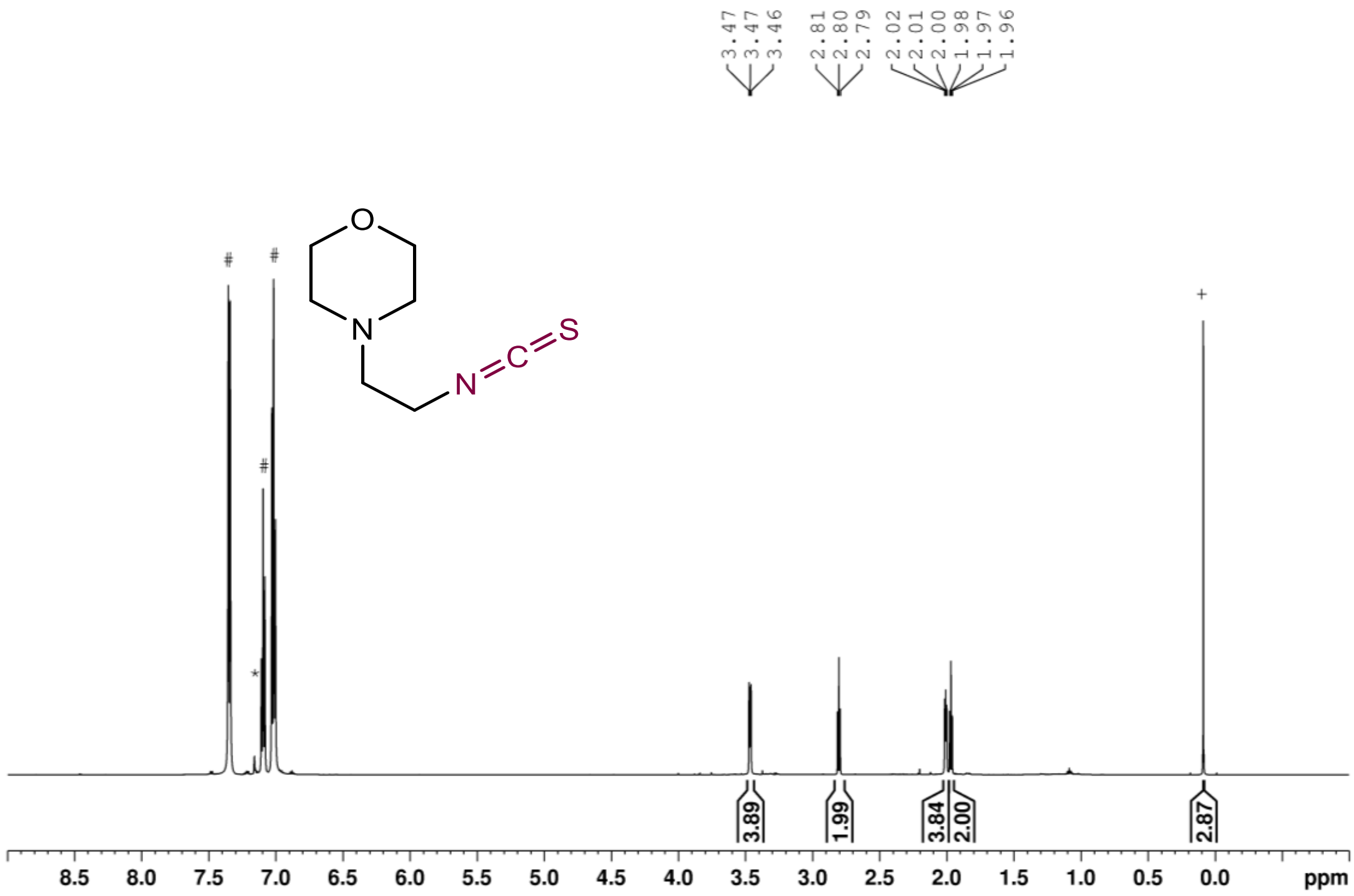

Figure S 31: ^{1}H NMR spectrum of 2-Morpholinoethylisothiocyanate (600 MHz, 298 K, benzene-d_6). Residual signals of deuterated solvent (*), TFT (#) and HMDSO (+) are marked.

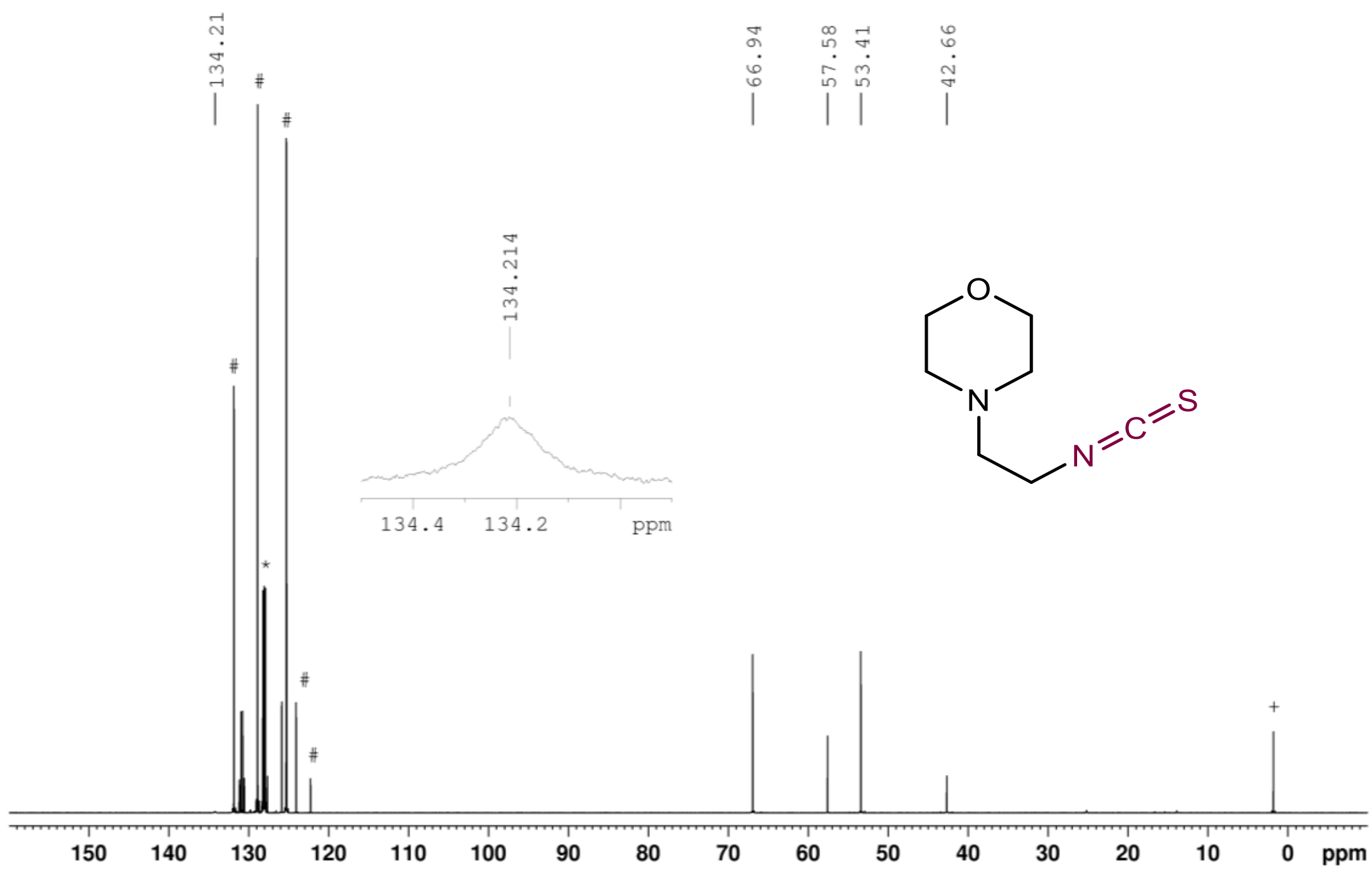

Figure S 32: ^{13}C NMR spectrum of 2-Morpholinoethylisothiocyanate (151 MHz, 298 K, benzene-d_6). Residual signals of deuterated solvent (*), TFT (#) and HMDSO (+) are marked.

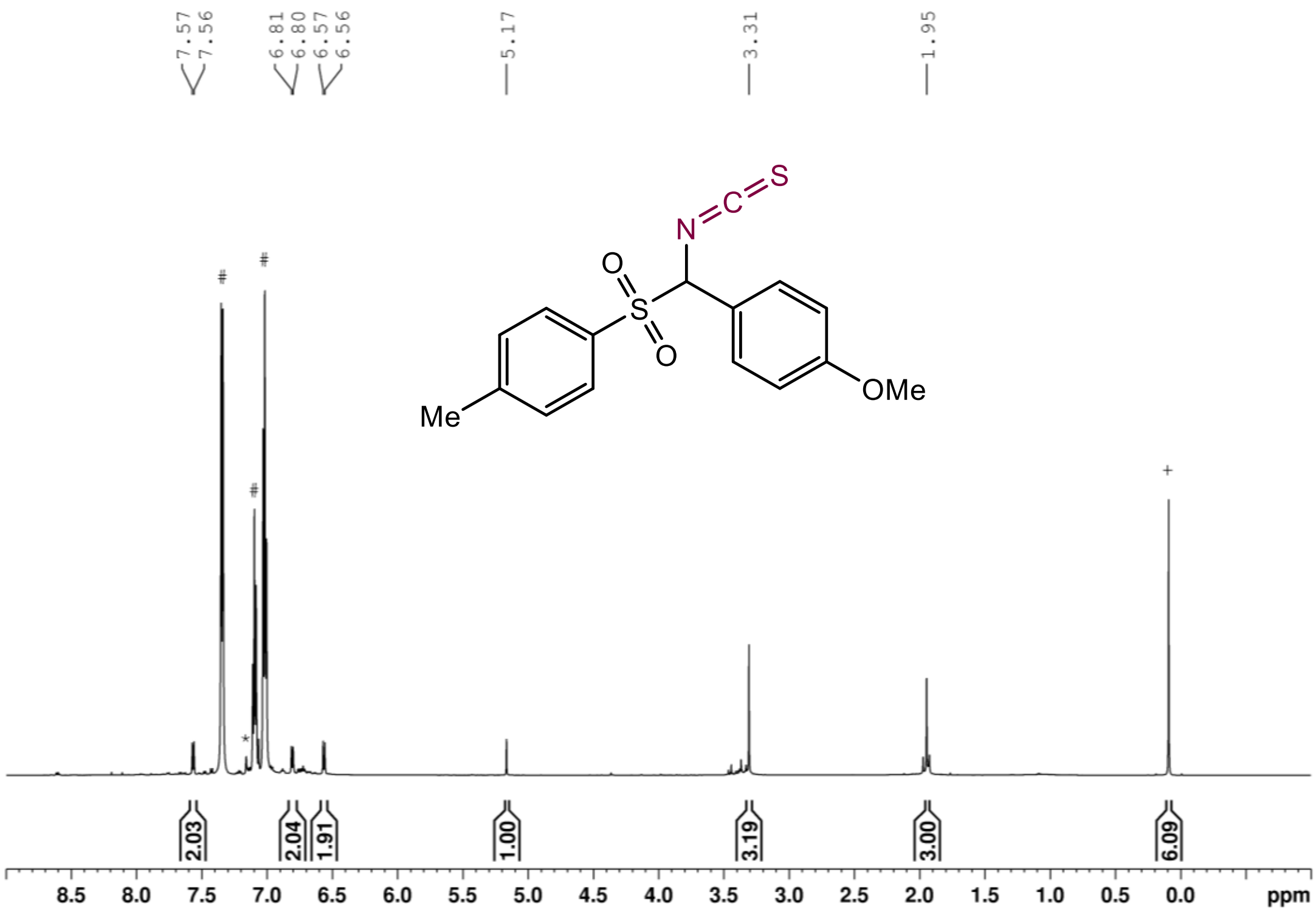

Figure S 33: ^{1}H NMR spectrum of α-Tosyl-(4-methoxybenzyl) isothiocyanate (600 MHz, 298 K, benzene-d6). Residual signals of deuterated solvent (*), TFT (#) and HMDSO (+) are marked.

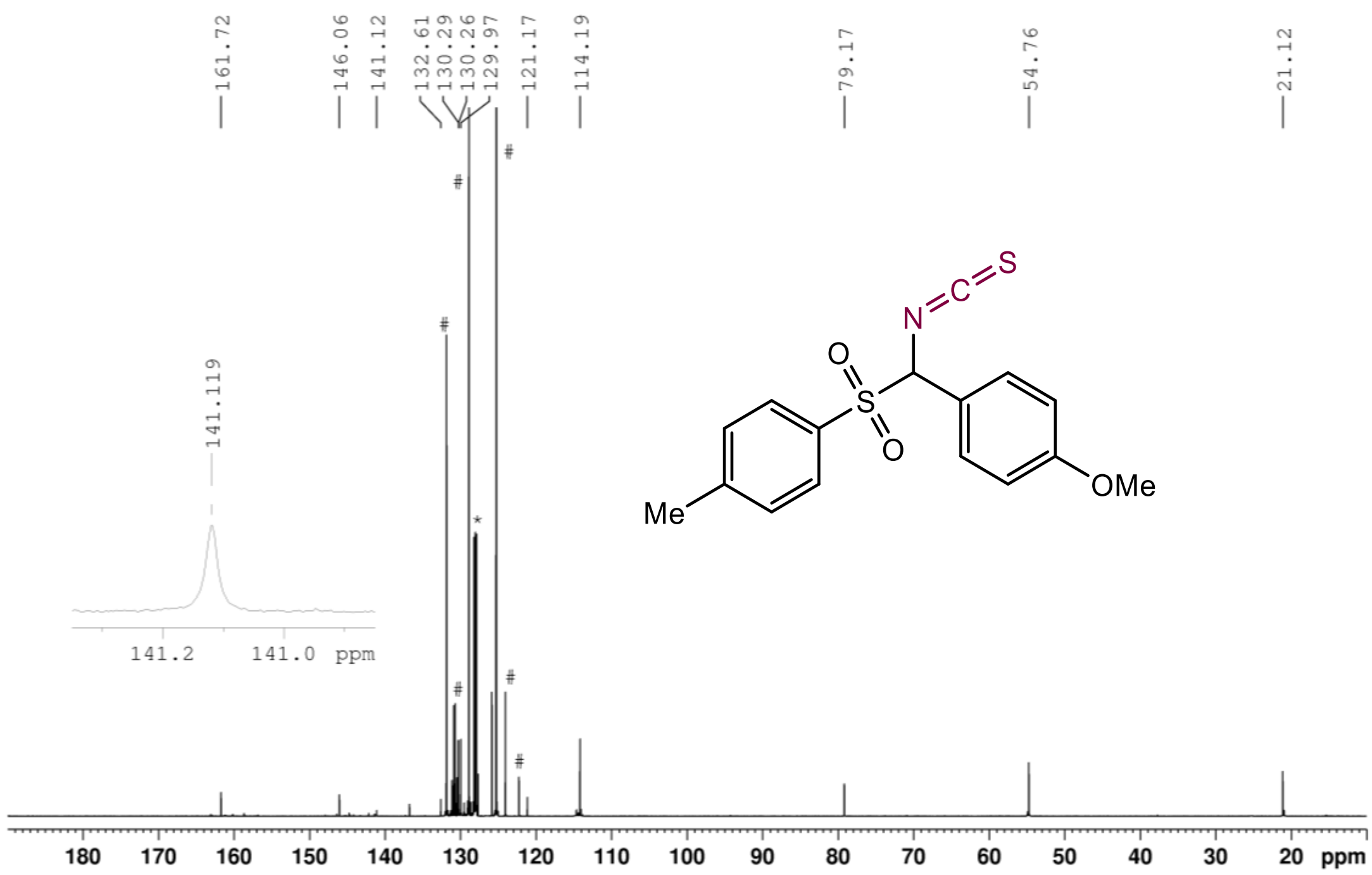

Figure S 34: ^{13}C NMR spectrum of α-Tosyl-(4-methoxybenzyl) isothiocyanate (151 MHz, 298 K, benzene-*d*6). Residual signals of deuterated solvent (*), TFT (#) and HMDSO (+) are marked.

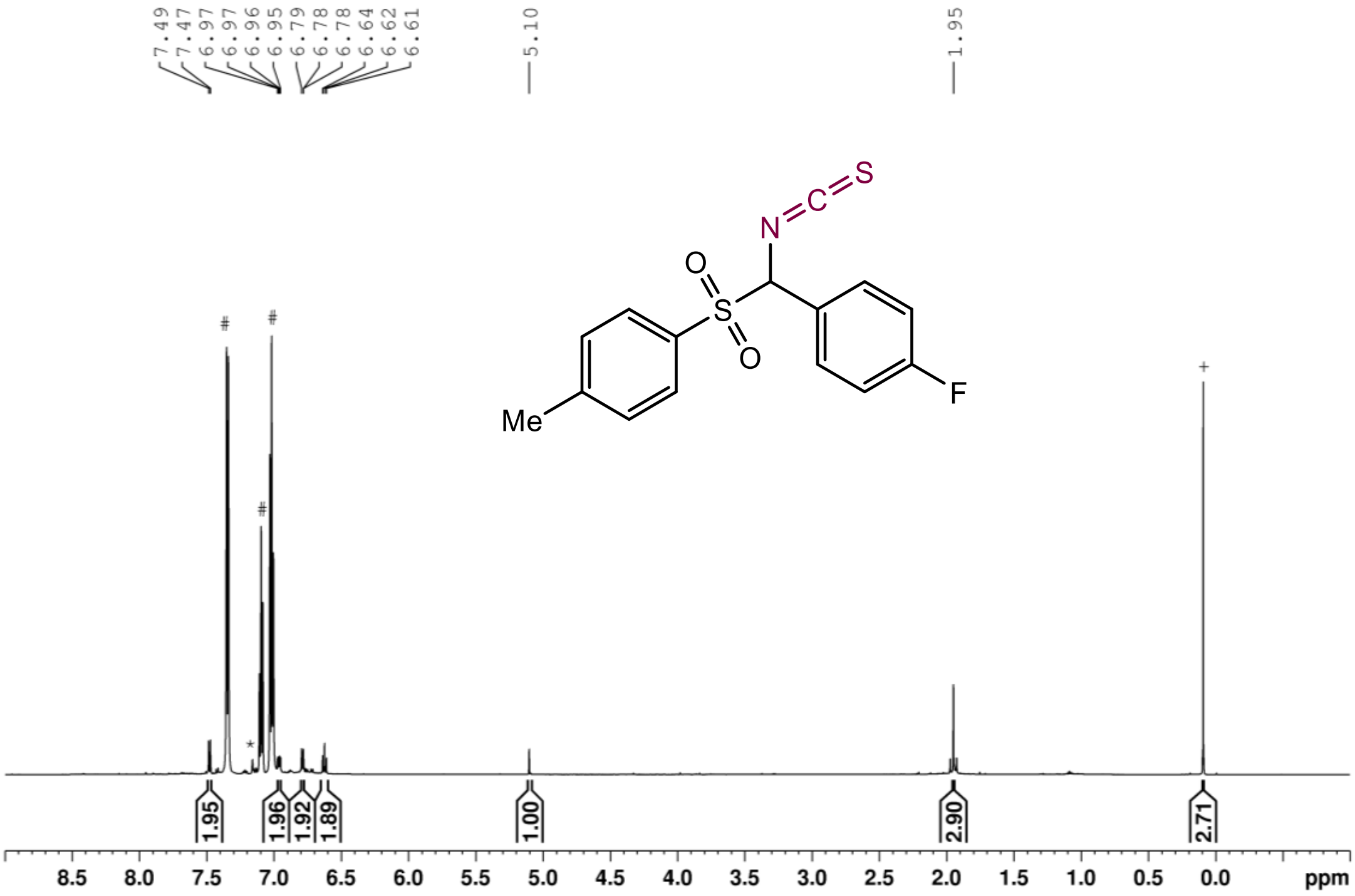

Figure S 35: ^{1}H NMR spectrum of α-Tosyl-(4-fluorobenzyl) isothiocyanate (600 MHz, 298 K, benzene-d_6). Residual signals of deuterated solvent (*), TFT (#) and HMDSO (+) are marked.

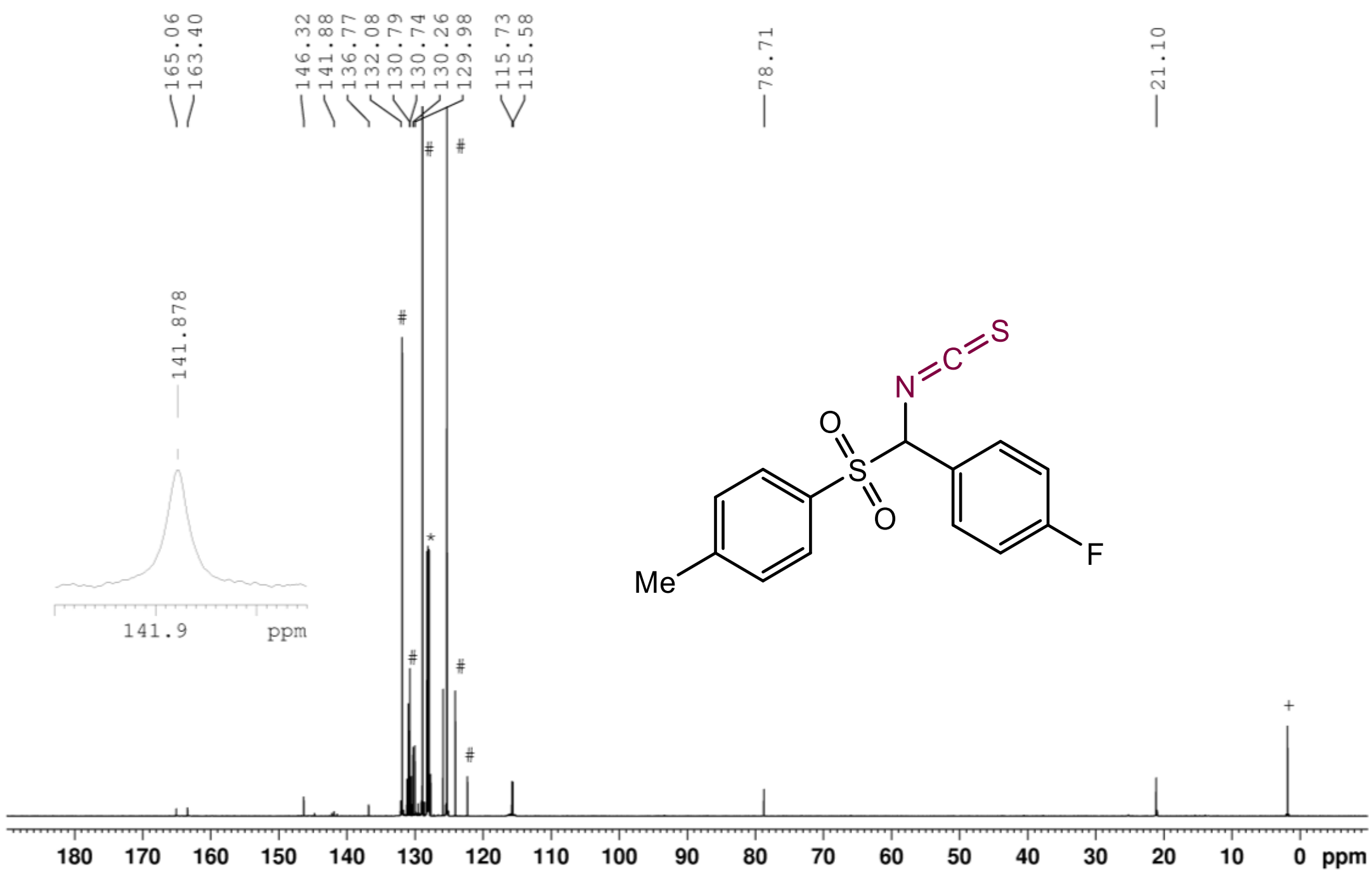

Figure S 36: ^{13}C NMR spectrum of α-Tosyl-(4-fluorobenzyl) isothiocyanate (151 MHz, 298 K, benzene-d_6). Residual signals of deuterated solvent (*), TFT (#) and HMDSO (+) are marked.

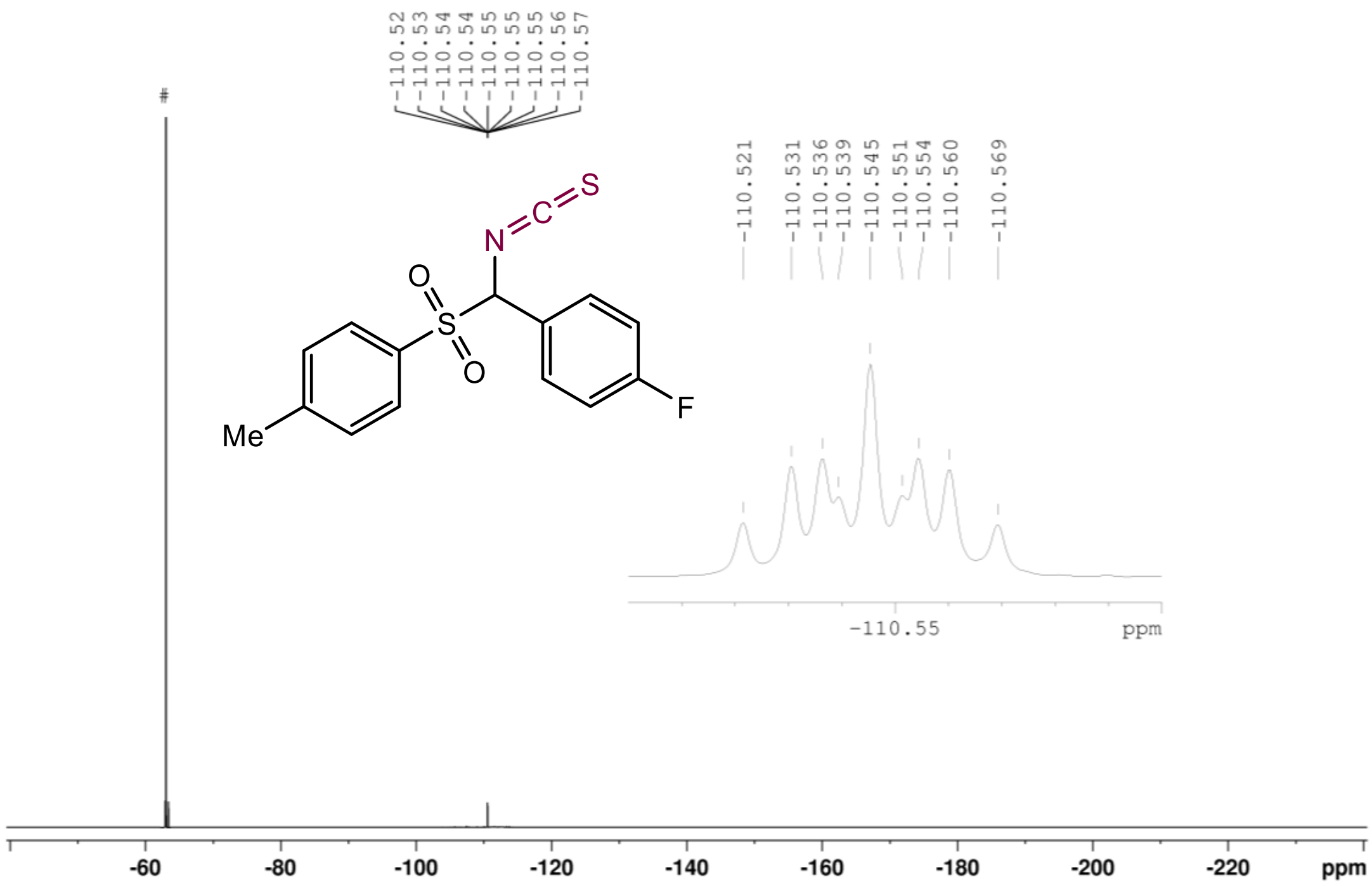

Figure S 37: ^{19}F NMR spectrum of α-Tosyl-(4-fluorobenzyl) isothiocyanate (565 MHz, 298 K, benzene-d_6). Residual signals TFT (#) are marked.

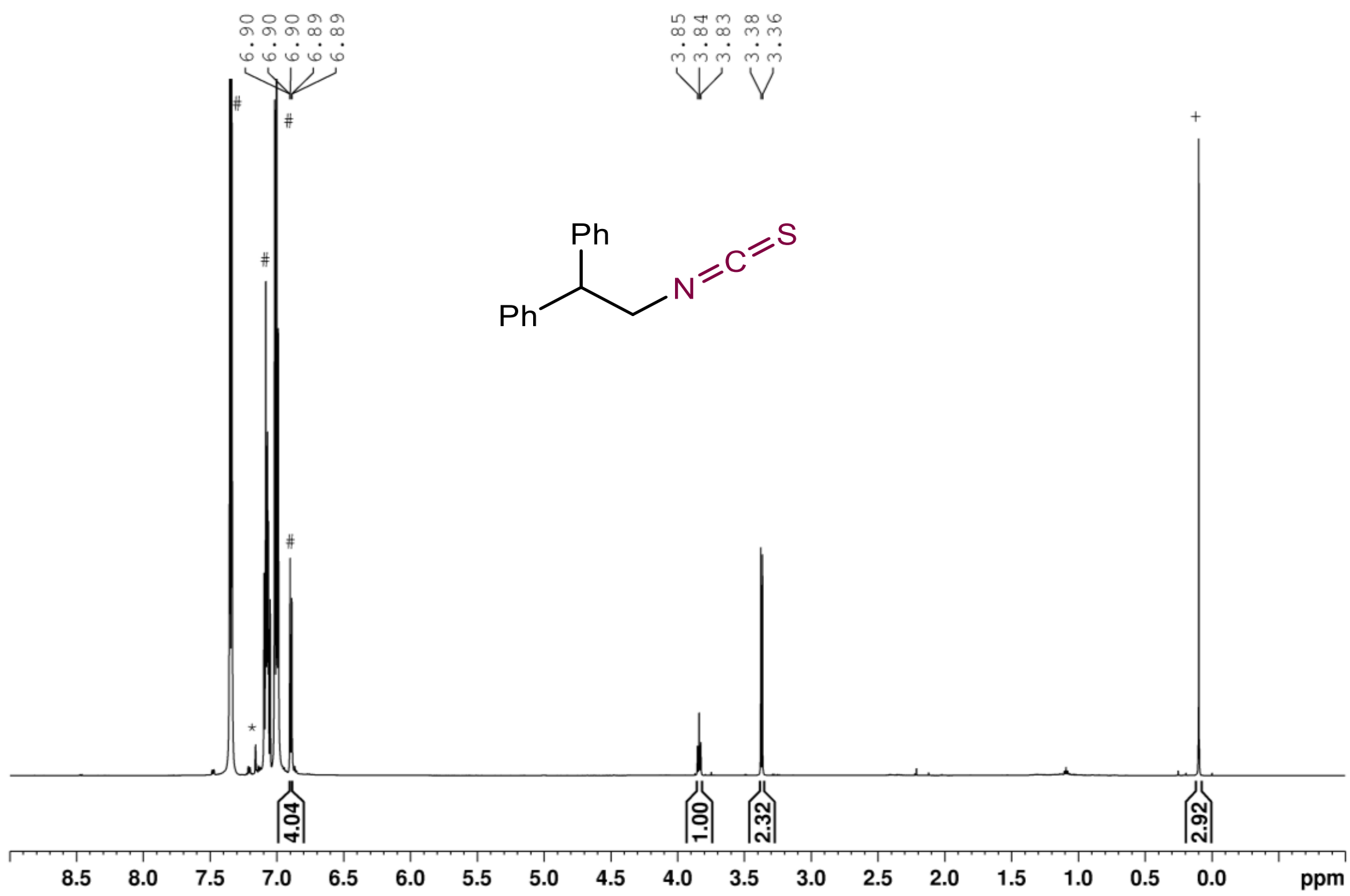

Figure S 38: ^{1}H NMR spectrum of 2,2-Diphenylethylisothiocyanate (600 MHz, 298 K, benzene-d$_6$). Residual signals of deuterated solvent (*), TFT (#) and HMDSO (+) are marked.

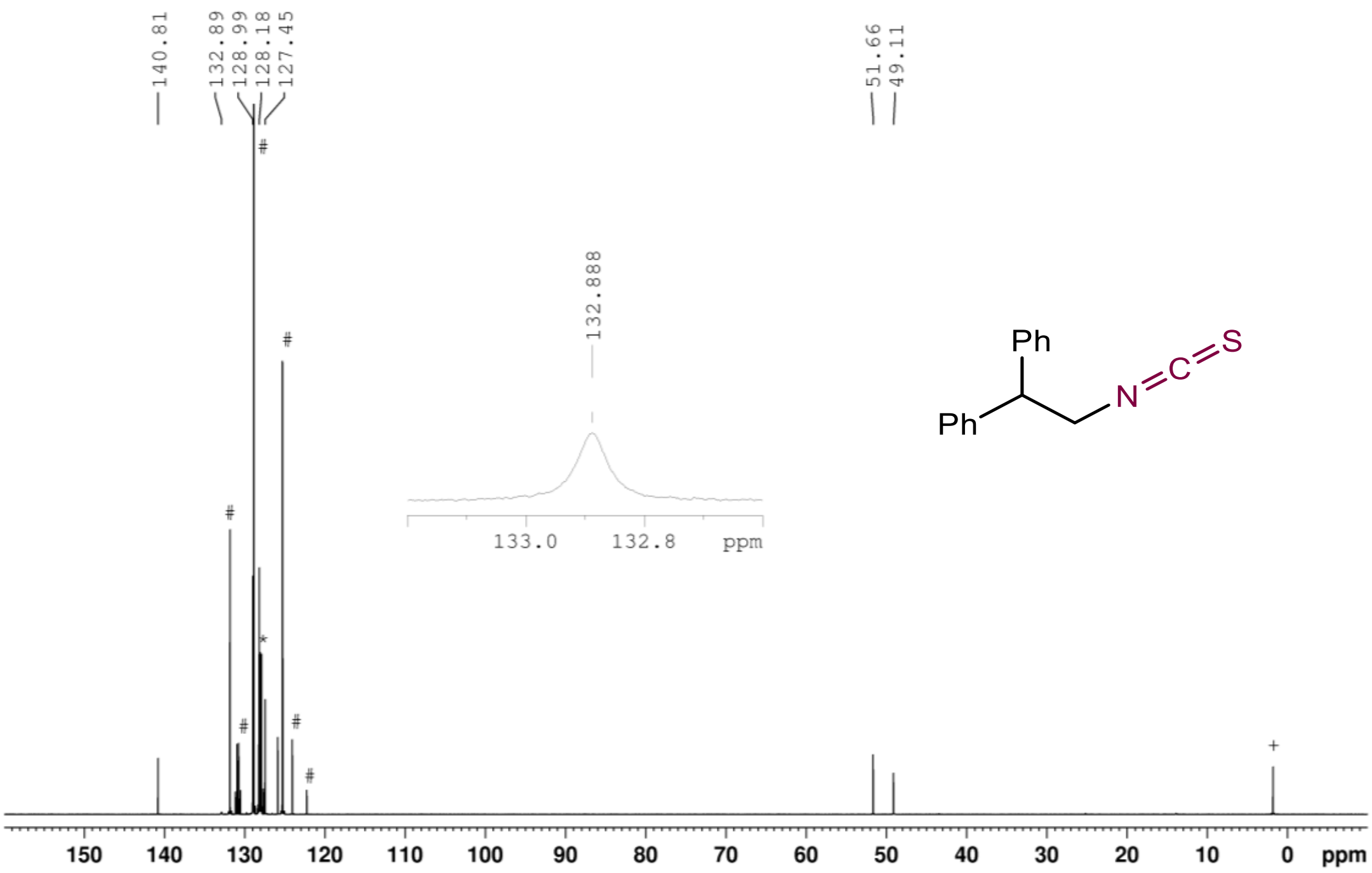

Figure S 39: ^{13}C NMR spectrum of 2,2-Diphenylethylisothiocyanate (151 MHz, 298 k, benzene-d$_6$). Residual signals of deuterated solvent (*), TFT (#) and HMDSO (+) are marked.

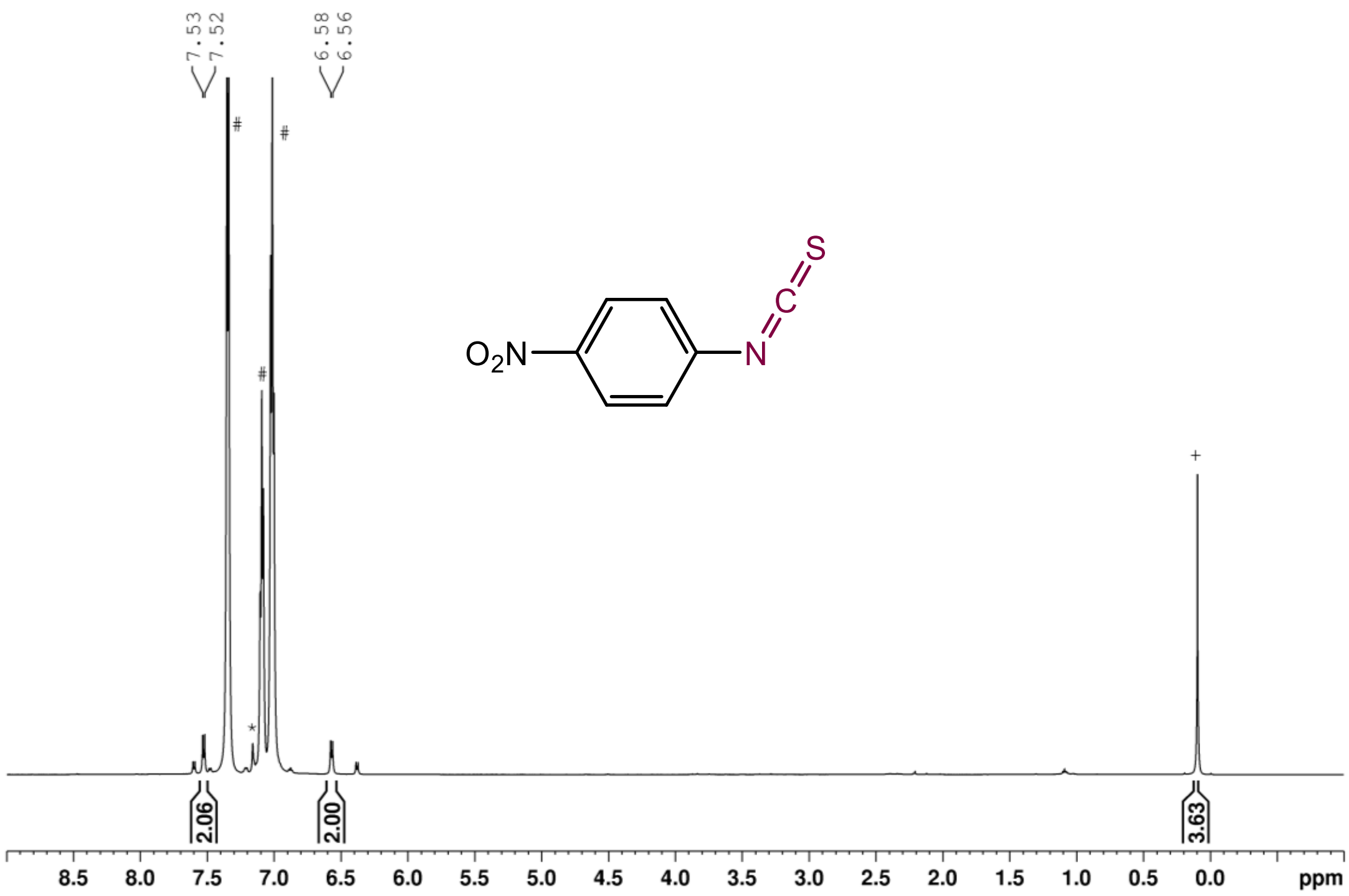

Figure S 40: ^{1}H NMR spectrum of 1-Isothiocyanato-4-nitrobenzene (600 MHz, 298 K, benzene-d_6). Residual signals of deuterated solvent (*), TFT (#) and HMDSO (+) are marked.

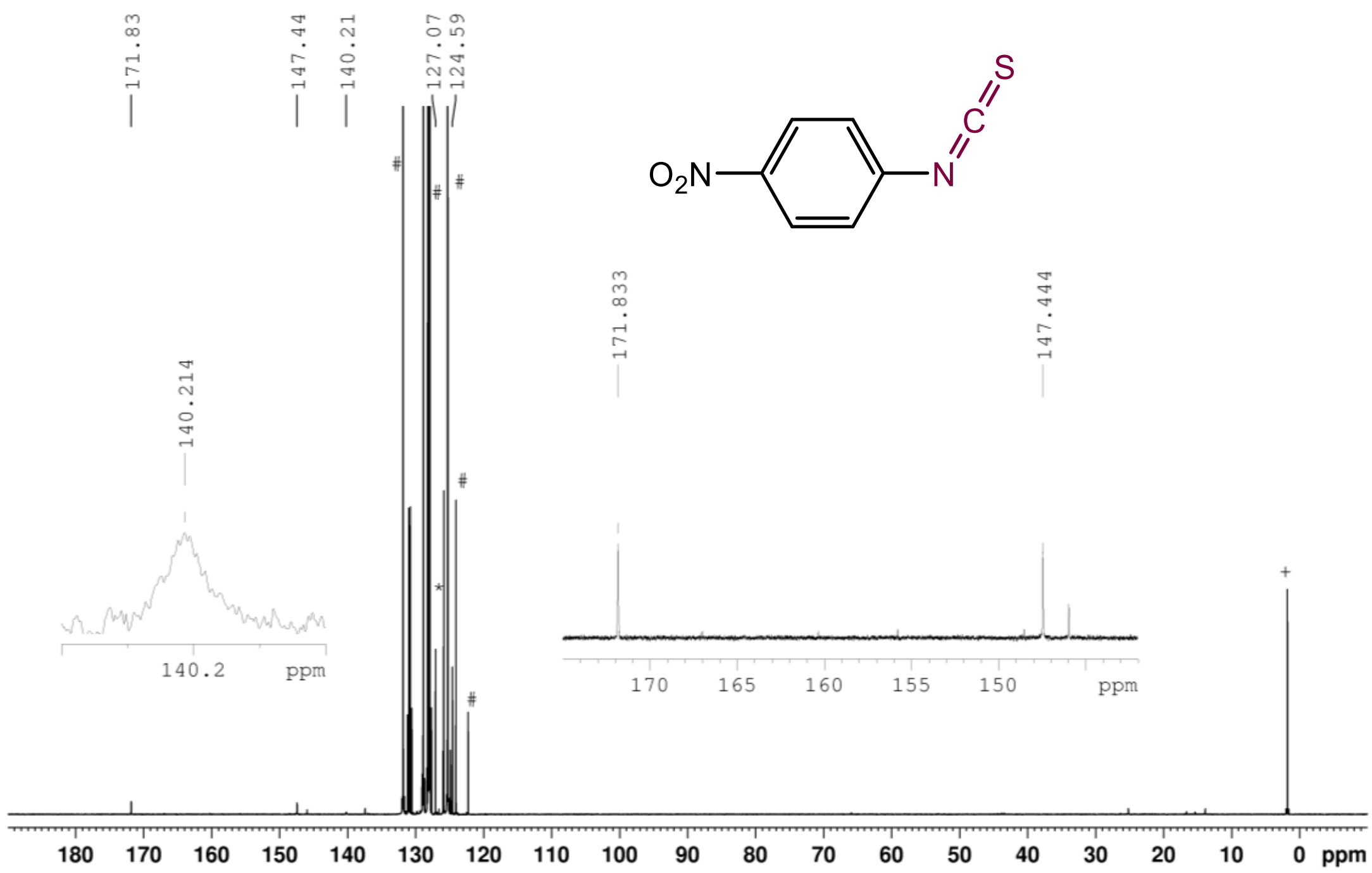

Figure S 41: ^{13}C NMR spectrum of 1-Isothiocyanato-4-nitrobenzene (151 MHz, 298 K, benzene-d_6). Residual signals of deuterated solvent (*), TFT (#) and HMDSO (+) are marked.

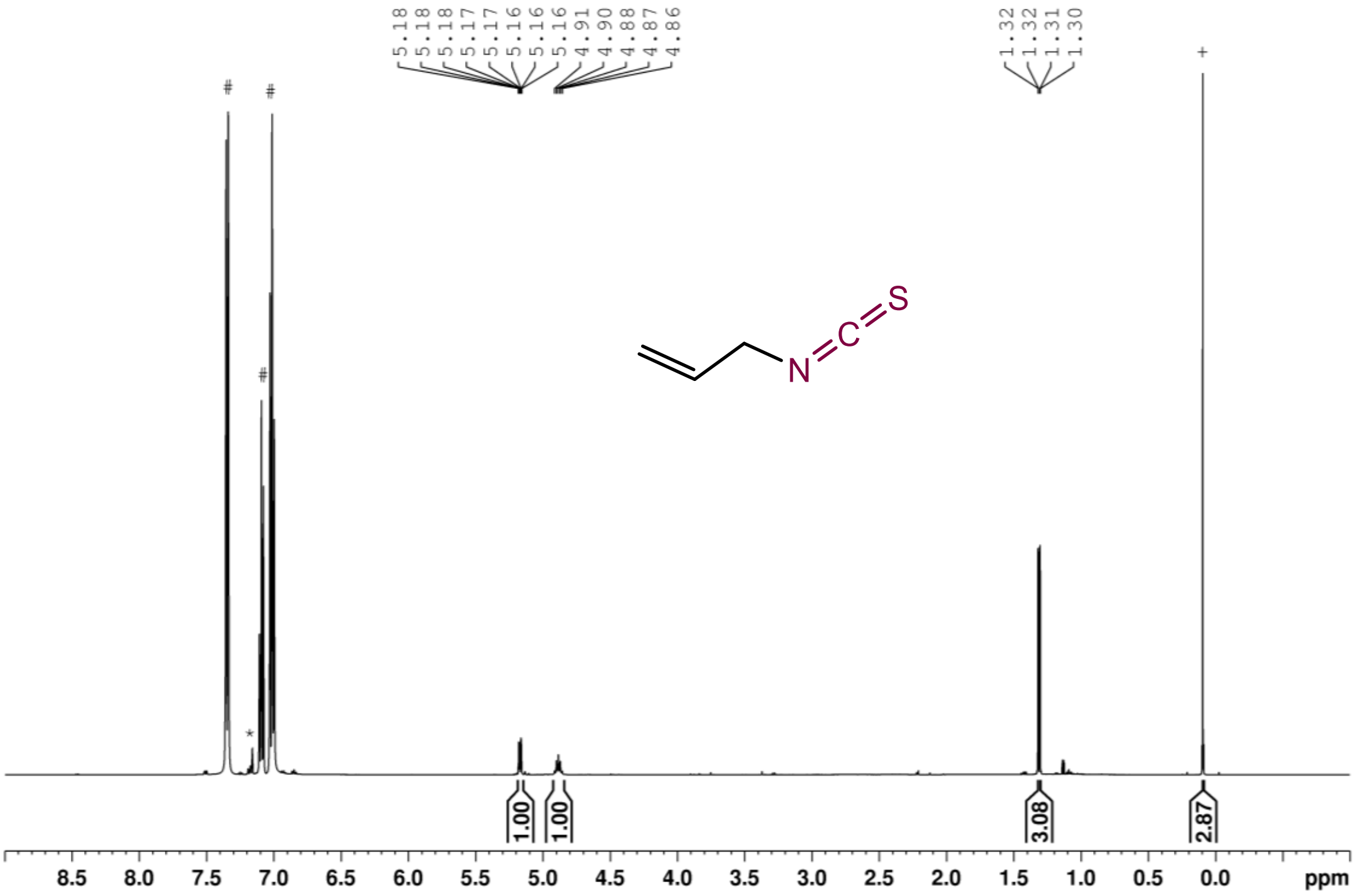

Figure S 42: ^{1}H NMR spectrum of Allylisothiocyanate (600 MHz, 298 K, benzene-d_6). Residual signals of deuterated solvent (*), TFT (#) and HMDSO (+) are marked.

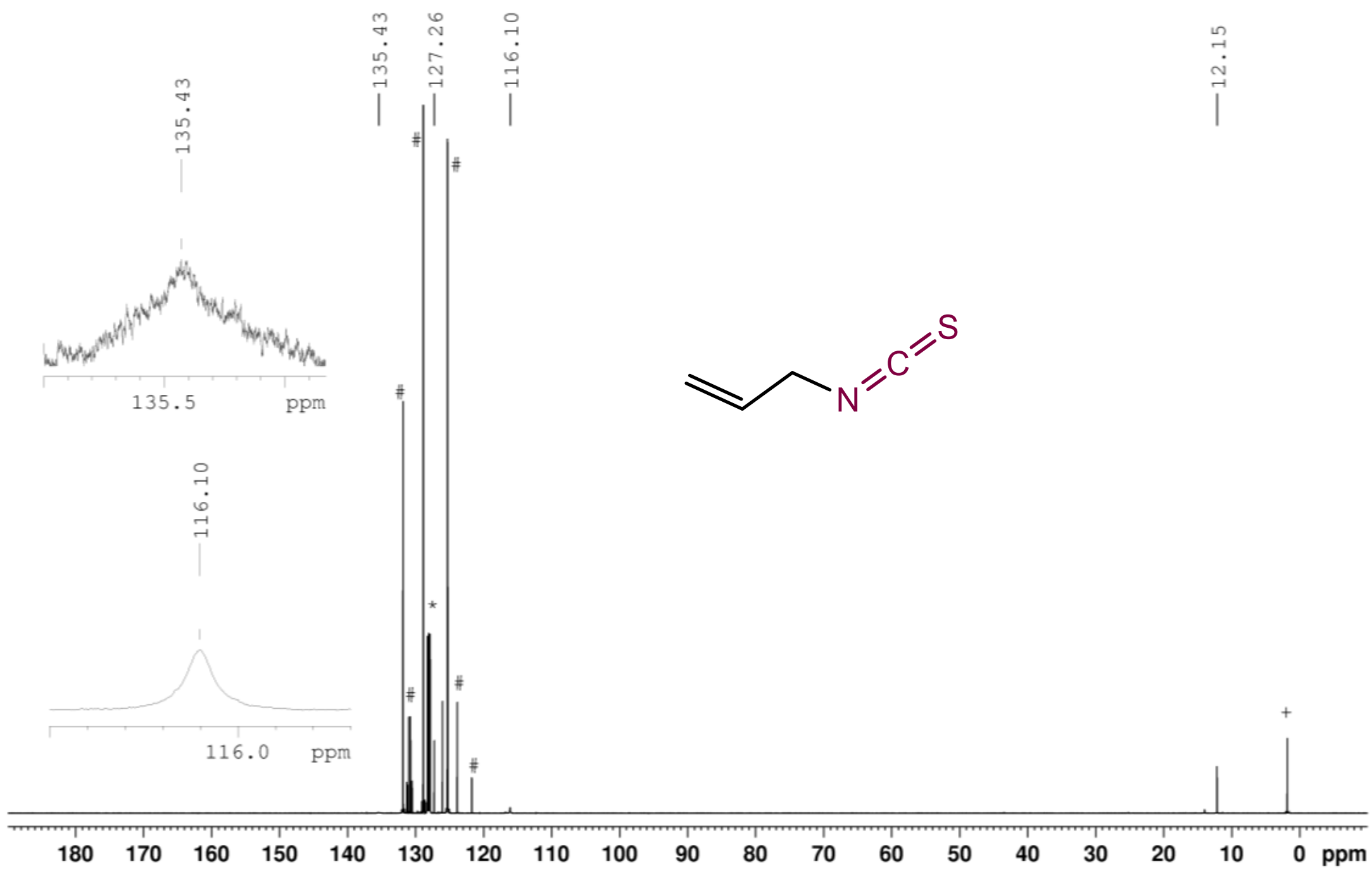

Figure S 43: ^{13}C NMR spectrum of Allylisothiocyanate (151 MHz, 298 K, benzene-d_6). Residual signals of deuterated solvent (*), TFT (#) and HMDSO (+) are marked.

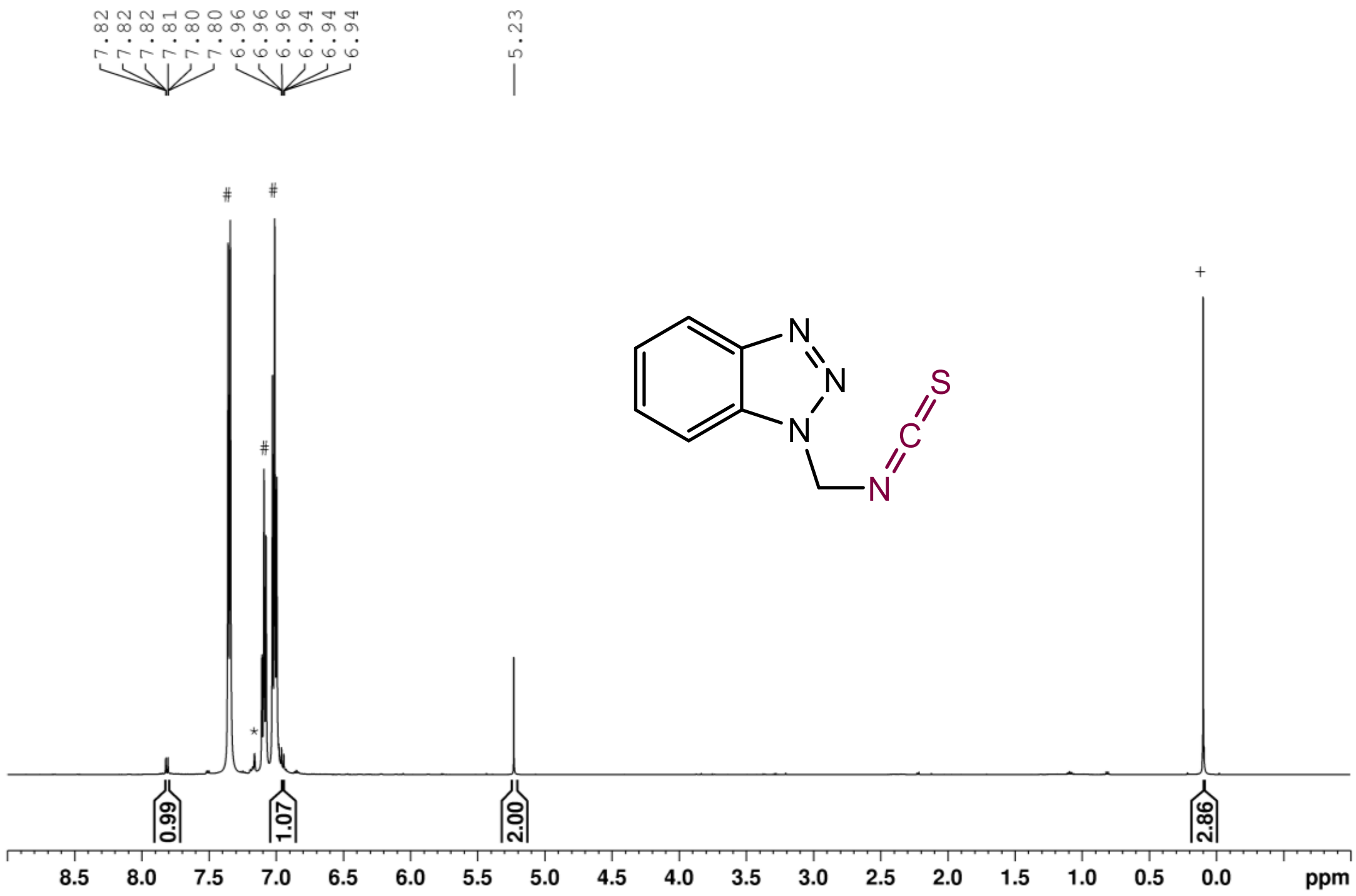

Figure S 44: ^{1}H NMR spectrum of (1H-Benzotriazol-1-yl)methylisothiocyanate (600 MHz, 298 K, benzene-d_6). Residual signals of deuterated solvent (*), TFT (#) and HMDSO (+) are marked.

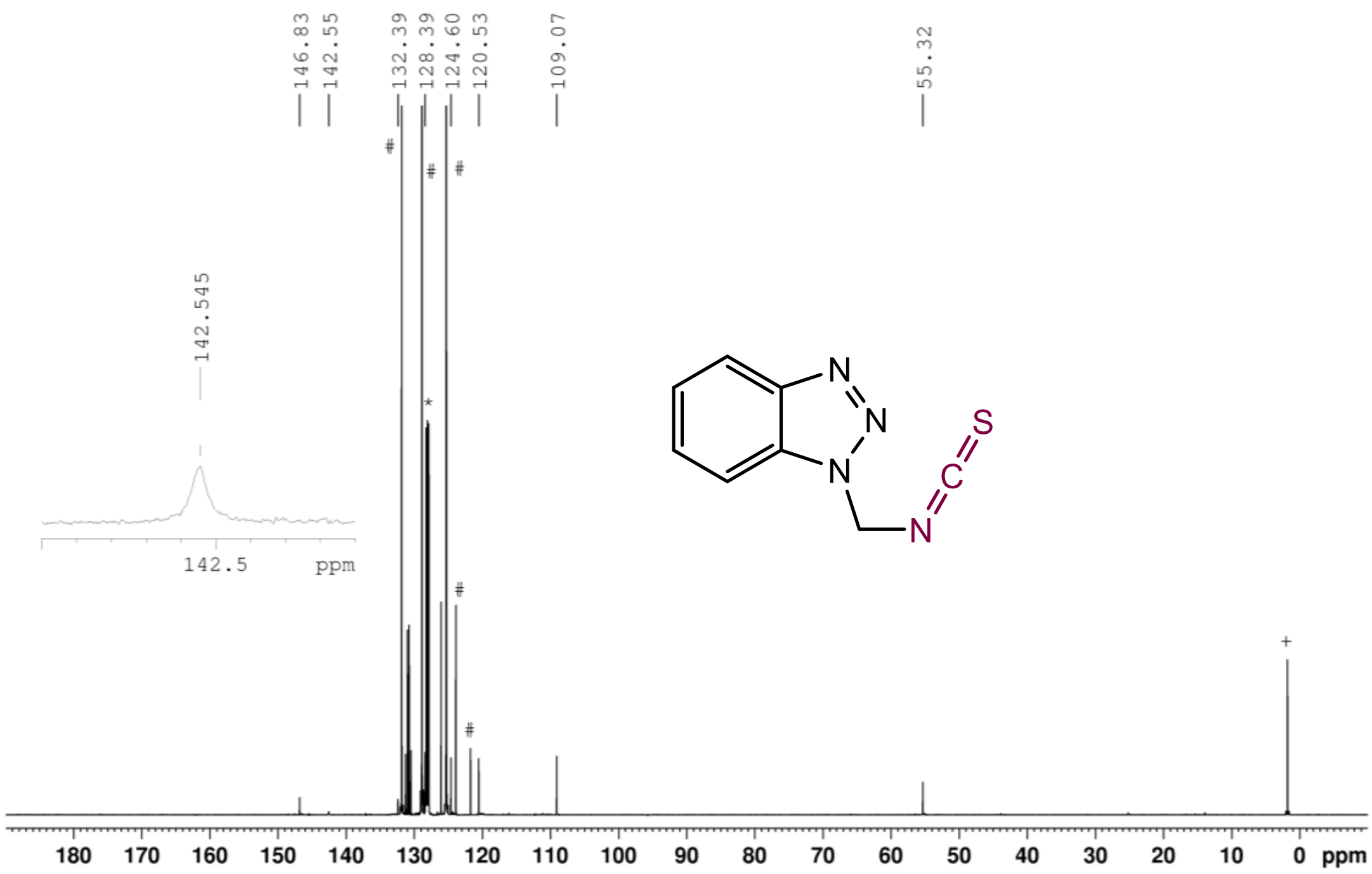

Figure S 45: ^{13}C NMR spectrum of (1H-Benzotriazol-1-yl)methylisothiocyanate (151 MHz, 298 K, benzene-d_6). Residual signals of deuterated solvent (*), TFT (#) and HMDSO (+) are marked.

6 Larger Scale Reactions

In order to demonstrate the applicability of **1-(S_2)$_2$** as a catalyst for the synthesis of isothiocyanates, a selection of isothiocyanates were synthesized and isolated on larger scale.

General Procedure C

A vial was charged with (^{Et}PDI)Ti(CH_2Ph)$_2$ (20.0 mg, 30.5 μmol, 0.05 equiv.) and sulfur (39.1 mg, 152.5 μmol, 0.25 equiv.) before adding benzene (250 μL) and α,α,α-trifluorotoluene (250 μL). Subsequently, the isonitrile (610.0 μmol, 1 equiv.) was added and the reaction was stirred at 23 °C for 48 hours. The crude product was purified by flash chromatography (*n*-pentane) and the product was stored at 2 °C.

Isothiocyanatocyclohexane

A vial was charged with with (^{Et}PDI)Ti(CH_2Ph)$_2$ (120.0 mg, 183.0 μmol, 0.05 equiv.) and sulfur (234.7 mg, 915.0 μmol, 0.25 equiv.) before adding benzene (1 mL) and α,α,α-trifluorotoluene (1 mL). Subsequently, the isonitrile (3.67 mmol, 1 equiv.) was added and the reaction was stirred at 23 °C for 48 hours. The crude product was purified by flash chromatography (*n*-pentane) to afford the product as a colorless oil (487.3 mg, 94%), which was stored at 2 °C. Additionally, excess sulfur was isolated (114.8 mg, 98%).

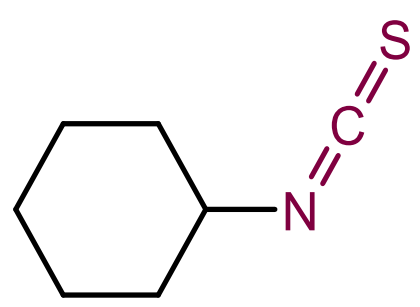

^{1}H NMR (500 MHz, 298 K, benzene-d_6) δ = 1.32-0.76 (m, 10H, C$H_{2,Cy}$), 2.84-2.79 (m, 1H, C*H*) ppm.

^{13}C NMR {H} (151 MHz, 298 K, benzene-d_6) δ = 23.25 (s, CH_2, *C*$H_2CH_2CH_2CH$), 25.03 (s, CH_2, *C*H_2CH_2CH), 32.02 (s, CH_2, *C*H_2CH), 55.27 (s, CH, *C*H), 131.72 (s, C_q, N*C*S) ppm.

The analytical data is consistent with previous reports.[4]

(Isothiocyanatomethyl)benzene

The title compound was synthesized following **General Procedure C** to afford the product as a colourless oil (83.0 mg, 91%).

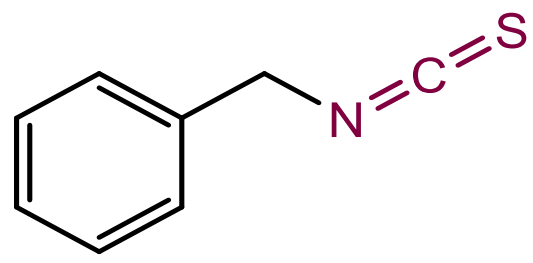

^{1}H NMR (400 MHz, 298 K, benzene-*d*$_6$) δ = 3.69 (s, 2H, CH_{2Ph}), 6.79-6.76 (m, 2H, CH_{Ar}), 6.99-6.97 (m, 3H, CH_{Ar},) ppm.

^{13}C NMR {H} (100 MHz, 298 K, benzene-*d*$_6$) δ = 48.13 (s, CH$_2$, CH$_{2Ph}$), 126.82 (s, CH, *o*/*m*-CH$_{Ph}$), 128.22 (s, CH, *p*-CH$_{Ph}$), 128.92 (s, CH, *o*/*m*-CH$_{Ph}$), 133.12 (s, br, C$_q$, N*C*S), 134.64 (s, C$_q$, C_{Ph}) ppm.

The analytical data is consistent with previous reports.[4]

(3s,5s,7s)-1-Isothiocyanatoadamantane

The title compound was synthesized following **General Procedure C** to afford the product as a colourless solid (108 mg, 92%).

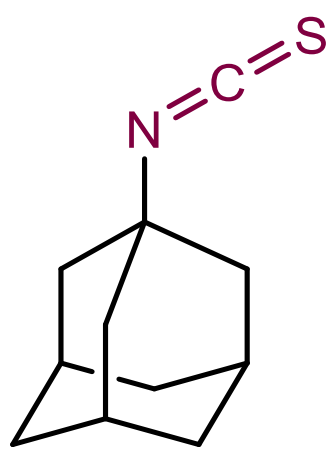

^{1}H NMR (400 MHz, 298 K, benzene-*d*$_6$) δ = 1.27-1.18 (m, 6H, CHCH_2CH), 1.57 (d, ^3J = 2.8 Hz, 6H, CH_2CNCS), 1.64-1.61 (m, 3H, C*H*) ppm.

^{13}C NMR {H} (100 MHz, 298 K, benzene-*d*$_6$) δ = 29.08 (s, CH, *C*H), 35.10 (s, CH$_2$, CHCH$_2$CH), 43.26 (s, CH$_2$, CH$_2$CNCS), 58.07 (s, C$_q$, *C*NCS), 131.52 (s, C$_q$, N*C*S) ppm.

The analytical data is consistent with previous reports.[7]

2-Isothiocyanato-1,3-dimethylbenzene

The title compound was synthesized following **General Procedure C** to afford the product as a colourless oil (79.5 mg, 80%).

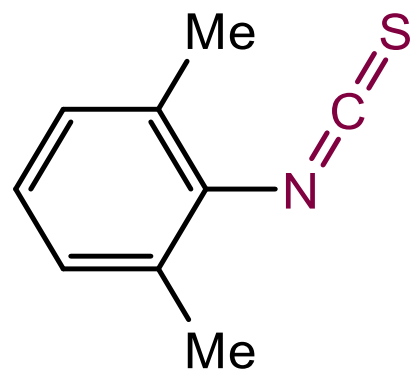

^{1}H NMR (400 MHz, 298 K, benzene-d_6) δ = 1.95 (s, 6H, CH_3), 6.59-6.57 (m, 2H, *m*-C*H*), 6.71-6.68 (m, 1H, *p*-C*H*) ppm.

^{13}C NMR {H} (100 MHz, 298 K, benzene-d_6) δ = 18.40 (s, CH_3, *C*H_3), 126.86 (s, CH, *p*-*C*H), 128.04 (s, CH, *m*-*C*H), 129.98 (s, C_q, *C*NCS), 135.19 (s, C_q, *C*CH_3),136.53 (s, C_q, N*C*S) ppm.

The analytical data is consistent with previous reports.[4]

(*S*)-(-)-α-Methylbenzylisothiocyanate

The title compound was synthesized following **General Procedure C** to afford the product as a colourless oil (78.4 mg, 79%).

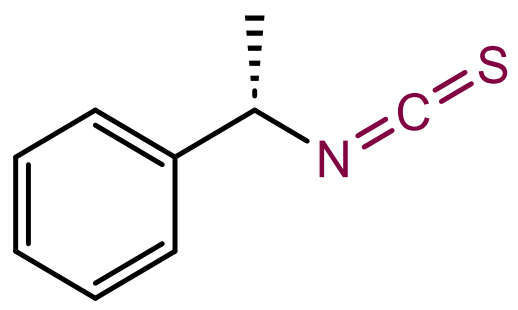

^{1}H NMR (400 MHz, 298 K, benzene-d_6) δ = 1.01 (d, ^3J = 6.8 Hz, 3H, C*H*), 4.05 (q, ^3J = 6.8 Hz, 1H, CH_3), 6.88-6.86 (m, 2H, *m*-CH_{Ar}), 7.02-6.97 (m, 3H, *o/p*-CH_{Ar}) ppm.

^{13}C NMR {H} (100 MHz, 298 K, benzene-d_6) δ = 24.63 (s, CH_3, *C*H_3), 57.01 (s, CH, *C*H), 125.52 (s, CH, *o*/*m*-*C*H_{Ph}), 128.14 (s, CH, *p*-*C*H_{Ph}), 128.96 (s, CH, *o*/*m*-*C*H_{Ph}), 133.79 (s, br, C_q, N*C*S), 140.58 (s, C_q, *C*$_{Ph}$) ppm.

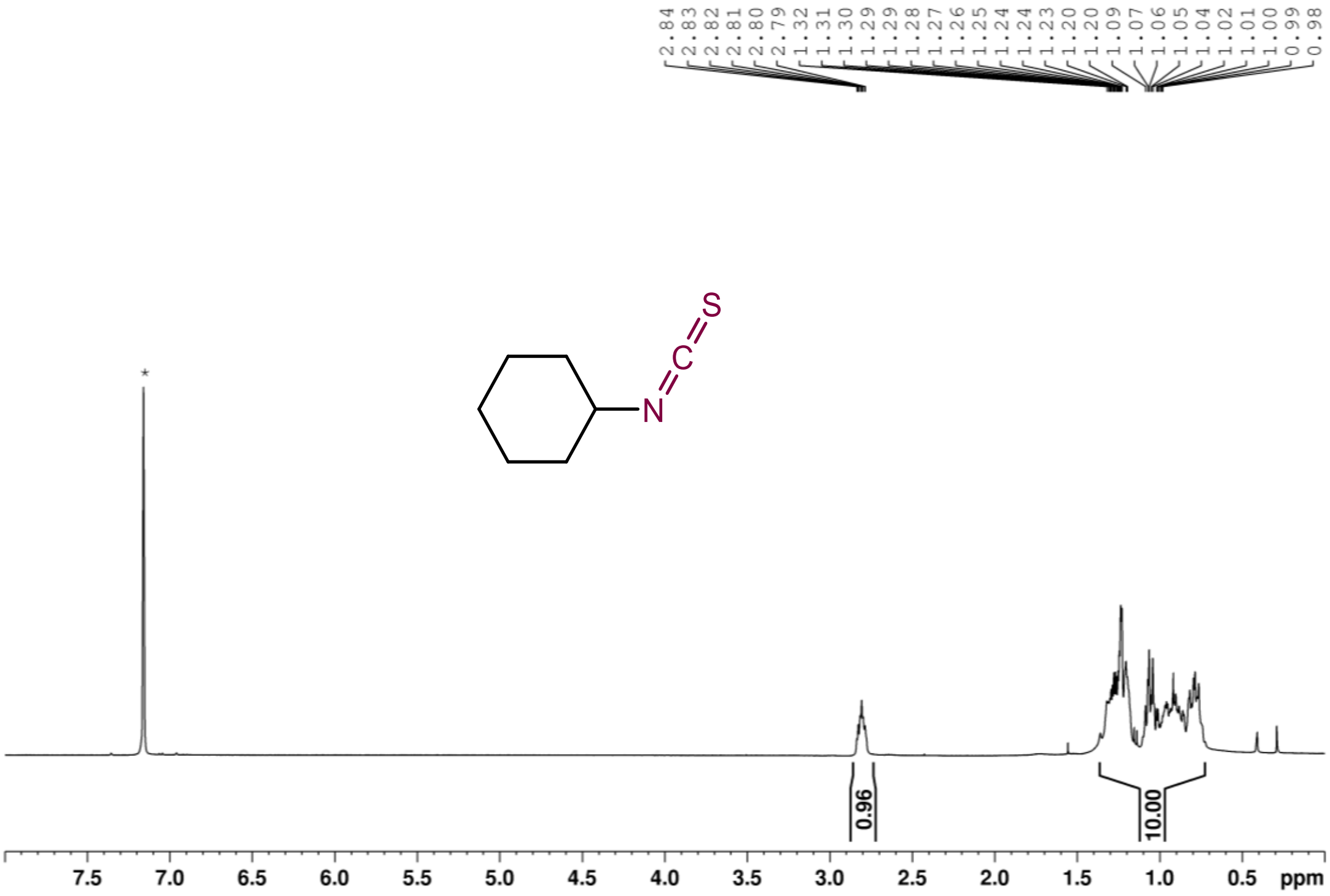

Figure S 46: ^{1}H NMR spectrum of Isothiocyanatocyclohexane (400 MHz, 298 K, benzene-d_6). Residual signals of deuterated solvent (*) are marked.

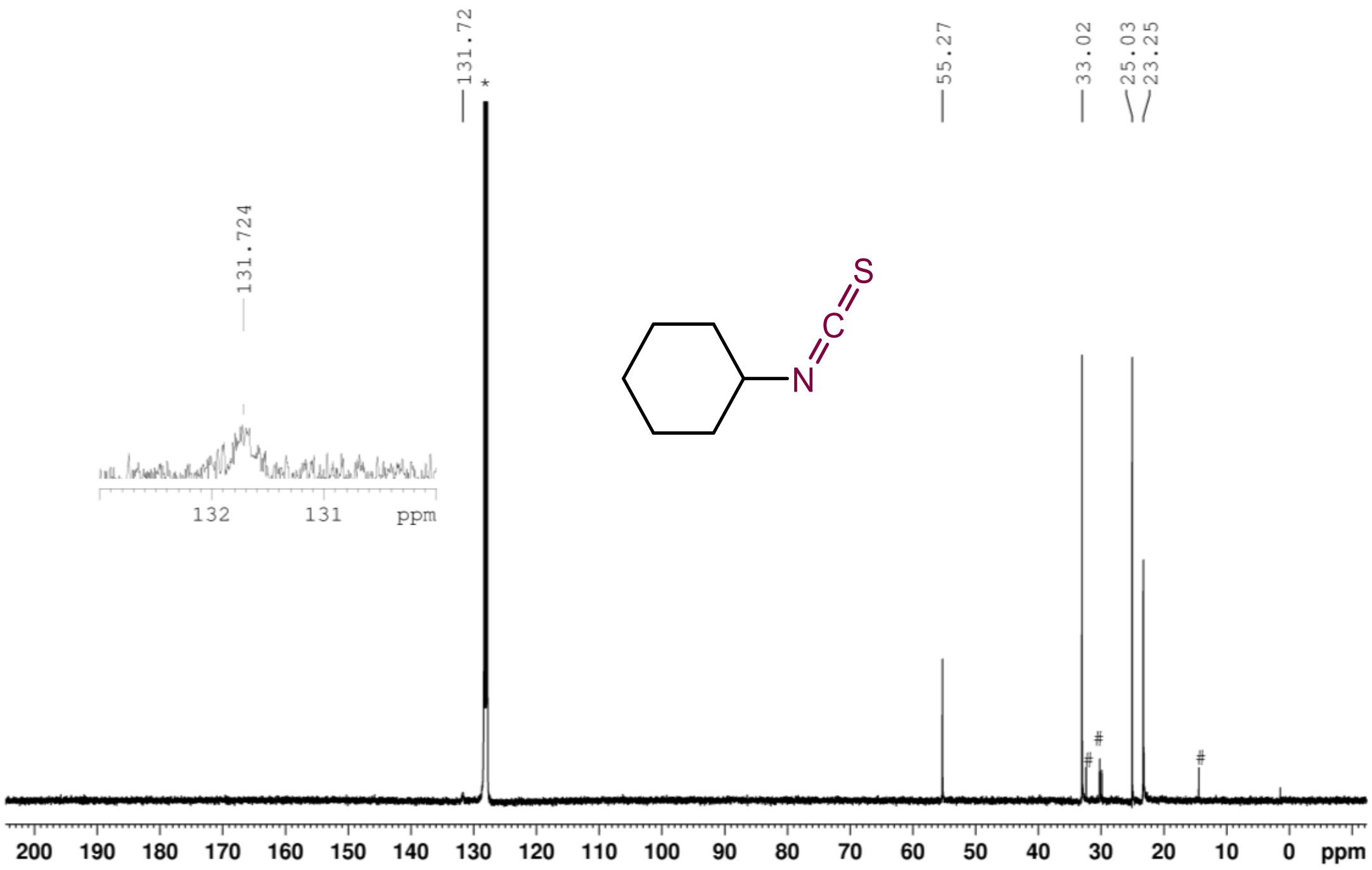

Figure S 47: ^{13}C NMR spectrum of Isothiocyanatocyclohexane (100 MHz, 298 K, benzene-d_6). Residual signals of deuterated solvent (*) and alkane impurities (#) are marked.

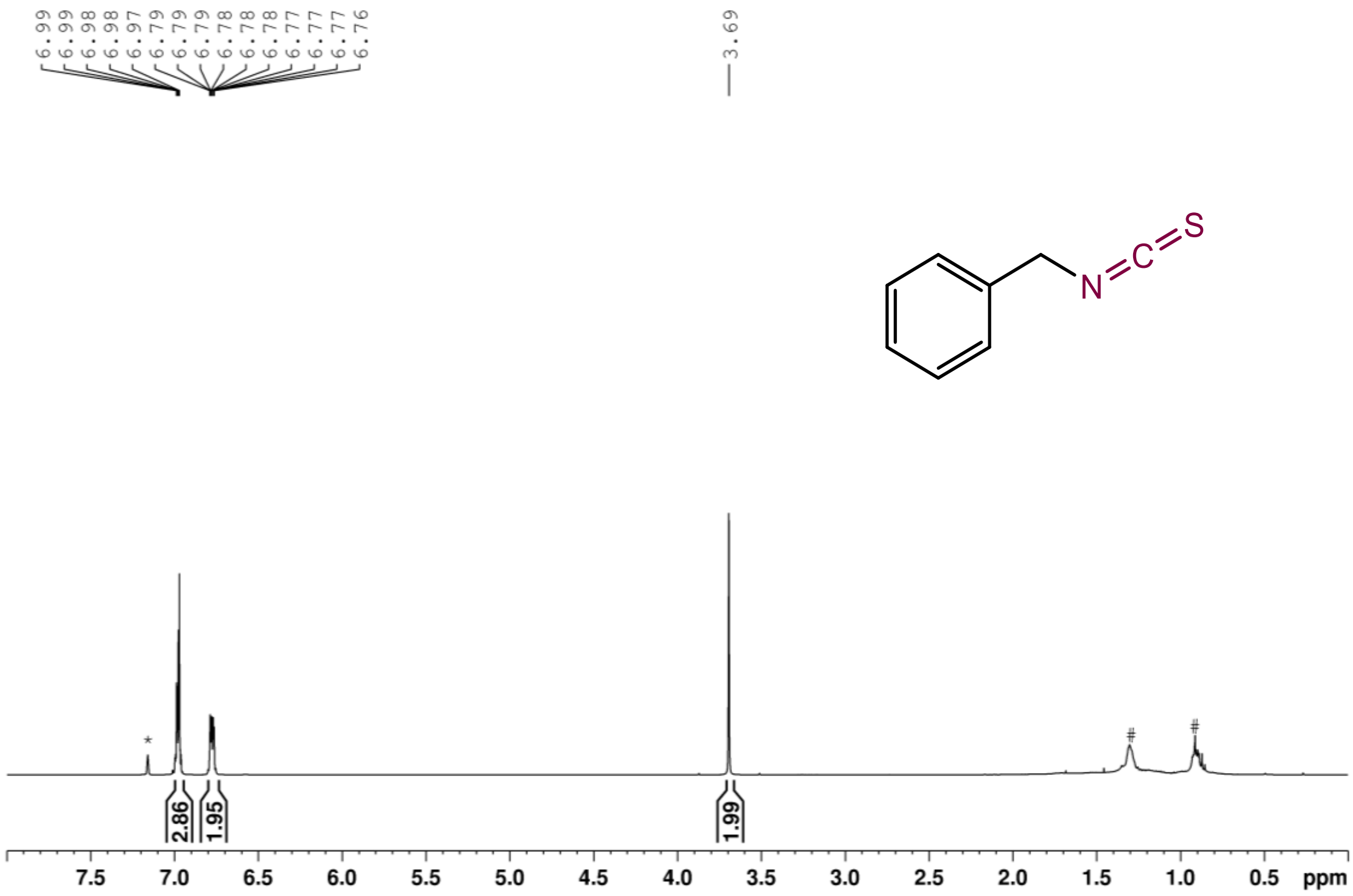

Figure S 48: ^{1}H NMR spectrum of (Isothiocyanatomethyl)benzene (400 MHz, 298 K, benzene-d_6). Residual signals of deuterated solvent (*) and alkane impurities (#) are marked.

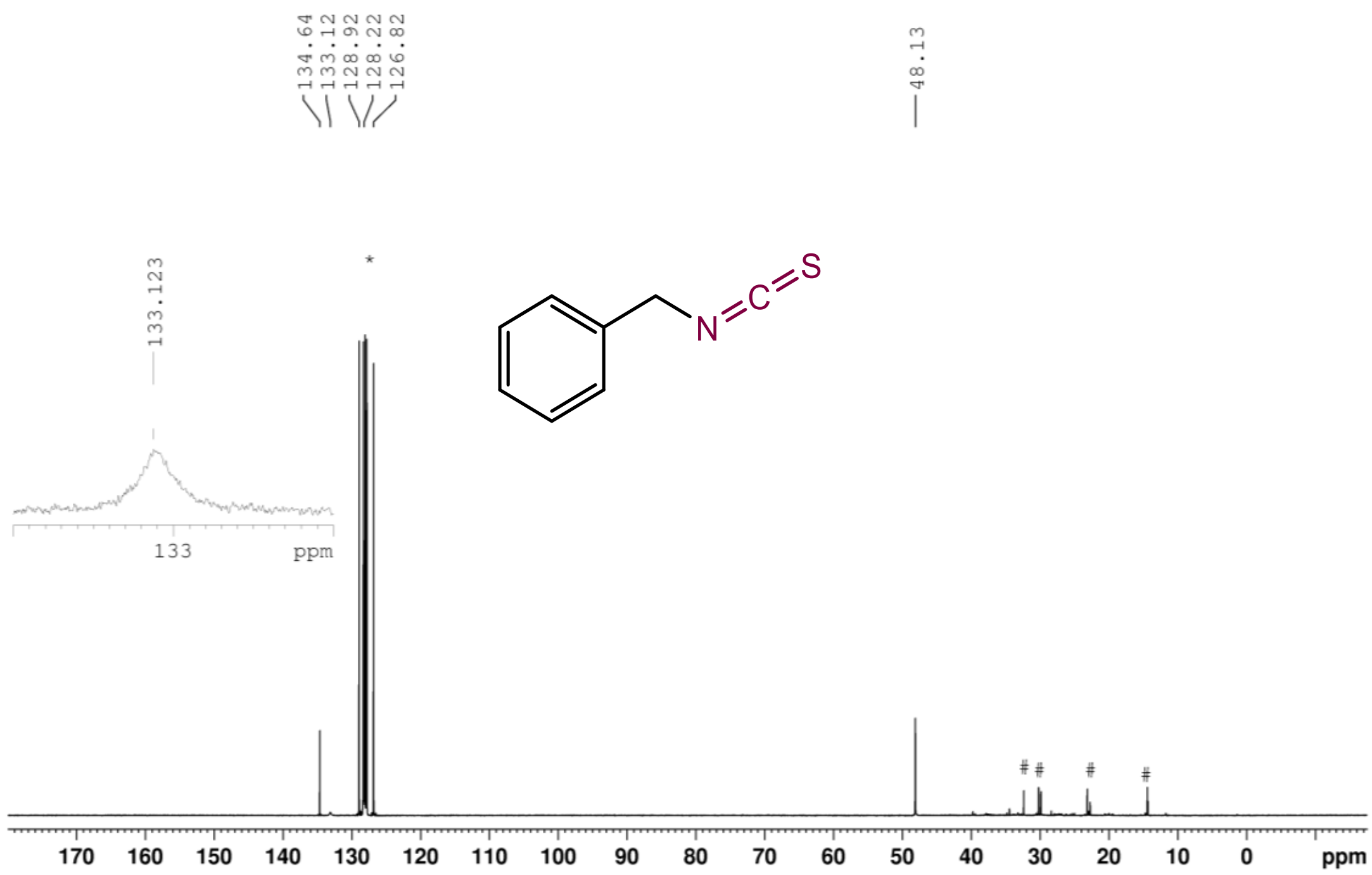

Figure S 49: ^{13}C NMR spectrum of (Isothiocyanatomethyl)benzene (100 MHz, 298 K, benzene-d_6). Residual signals of deuterated solvent (*) and alkane impurities (#) are marked.

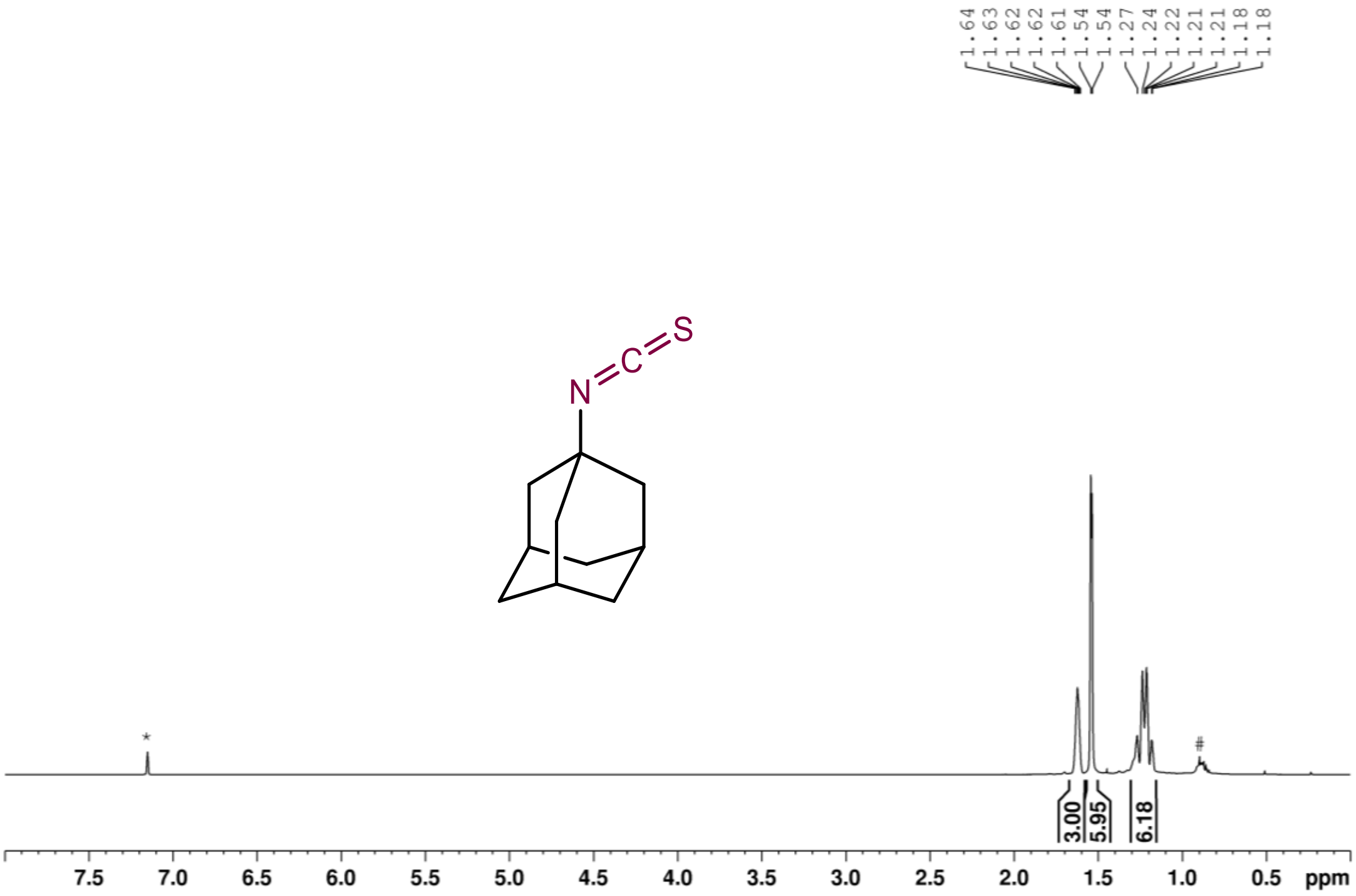

Figure S 50: ^{1}H NMR spectrum of (3s,5s,7s)-1-Isothiocyanatoadamantane (400 MHz, 298 K, benzene-d$_6$). Residual signals of deuterated solvent (*) and alkane impurities (#) are marked.

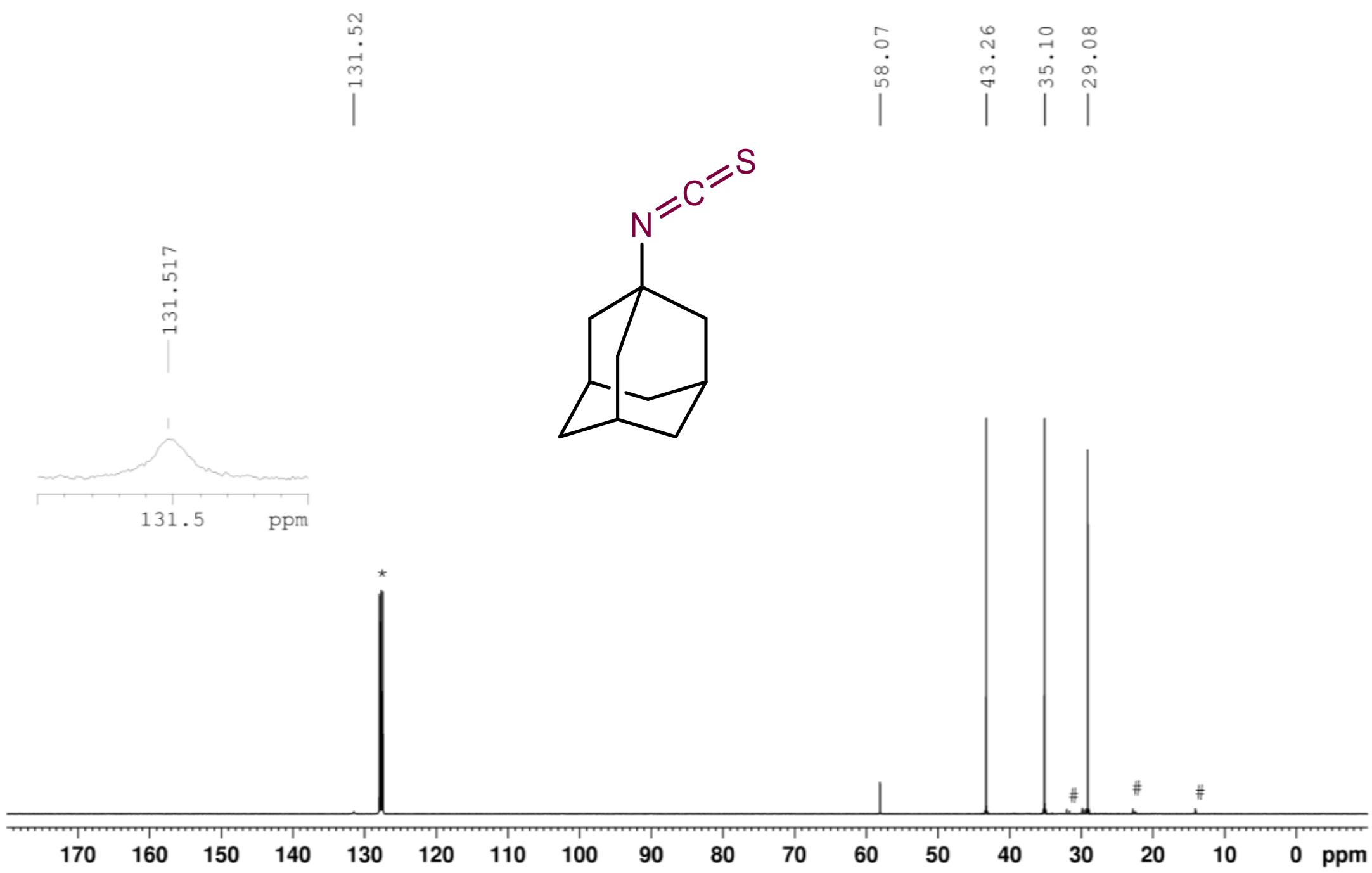

Figure S 51: ^{13}C NMR spectrum of (3s,5s,7s)-1-Isothiocyanatoadamantane (100 MHz, 298 K, benzene-d$_6$). Residual signals of deuterated solvent (*) and alkane impurities (#) are marked.

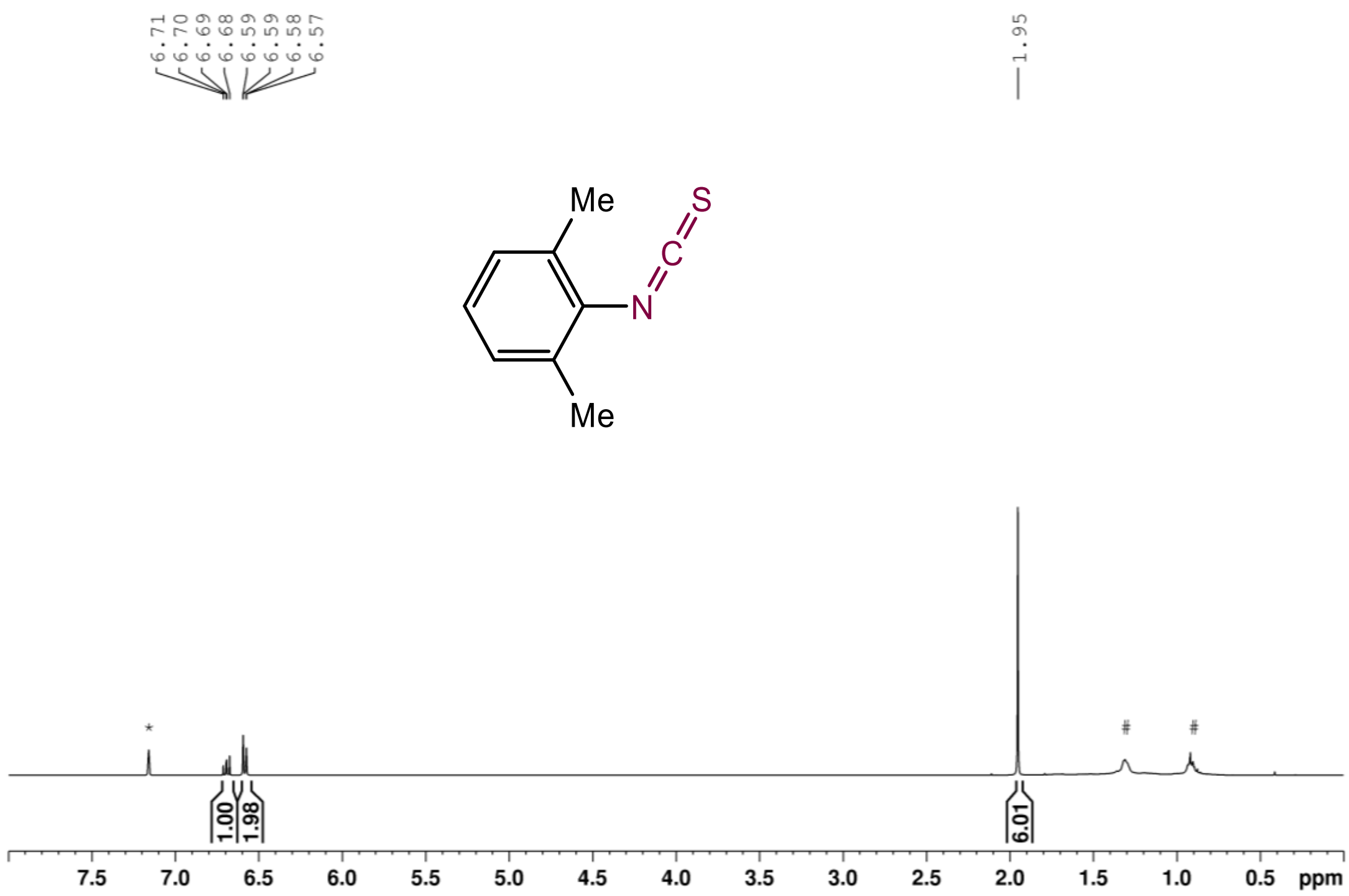

Figure S 52: ^{1}H NMR spectrum of 2-Isothiocyanato-1,3-dimethylbenzene (400 MHz, 298 K, benzene-d$_6$). Residual signals of deuterated solvent (*) and alkane impurities (#) are marked.

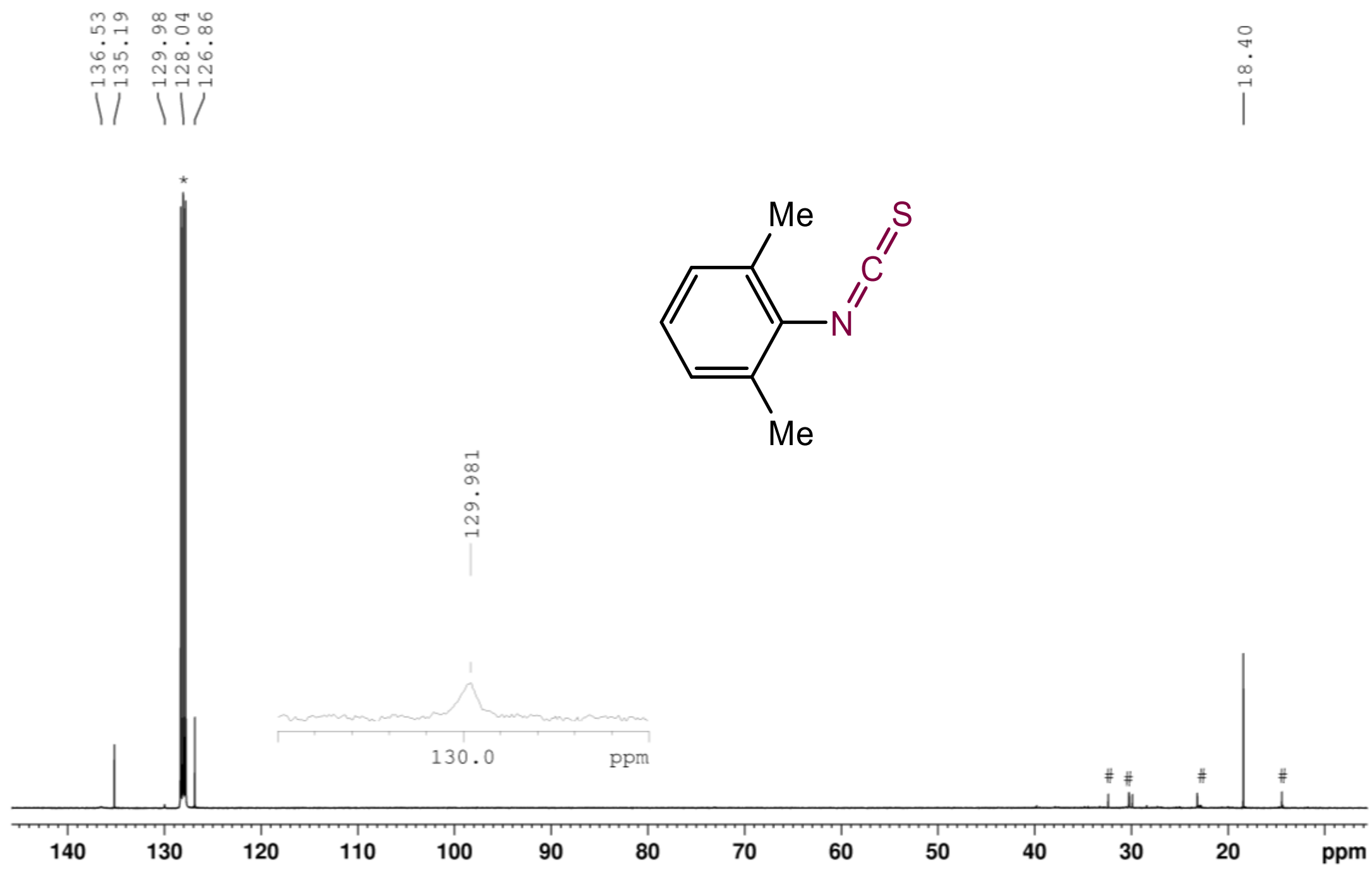

Figure S 53: ^{13}C NMR spectrum of 2-Isothiocyanato-1,3-dimethylbenzene (100 MHz, 298 K, benzene-d$_6$). Residual signals of deuterated solvent (*) and alkane impurities (#) are marked.

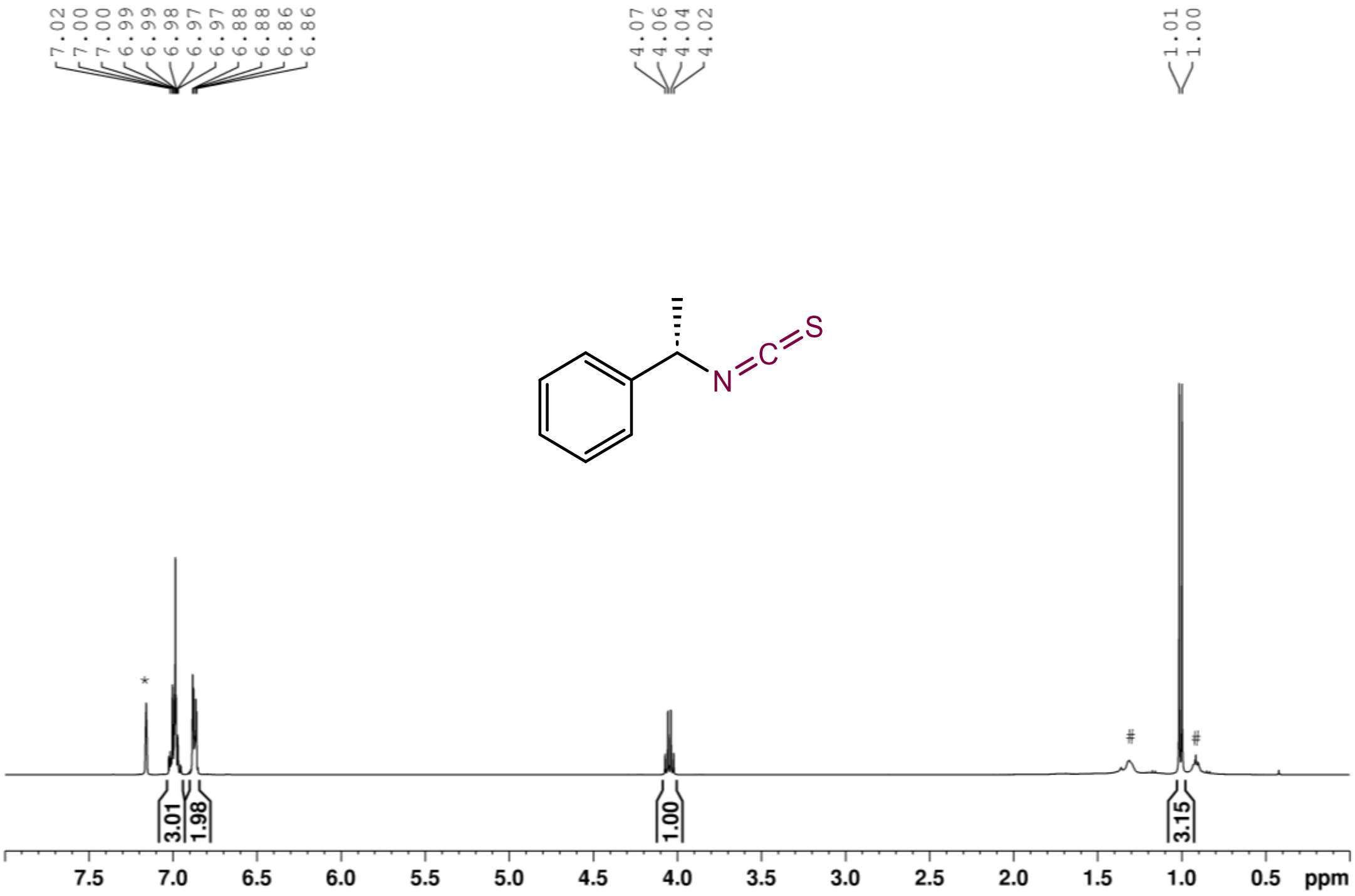

Figure S 54*:* ^{1}H NMR spectrum of (S)-(-)-α-Methylbenzylisothiocyanate (400 MHz, 298 K, benzene-d_6). Residual signals of deuterated solvent (*) and alkane impurities (#) are marked.

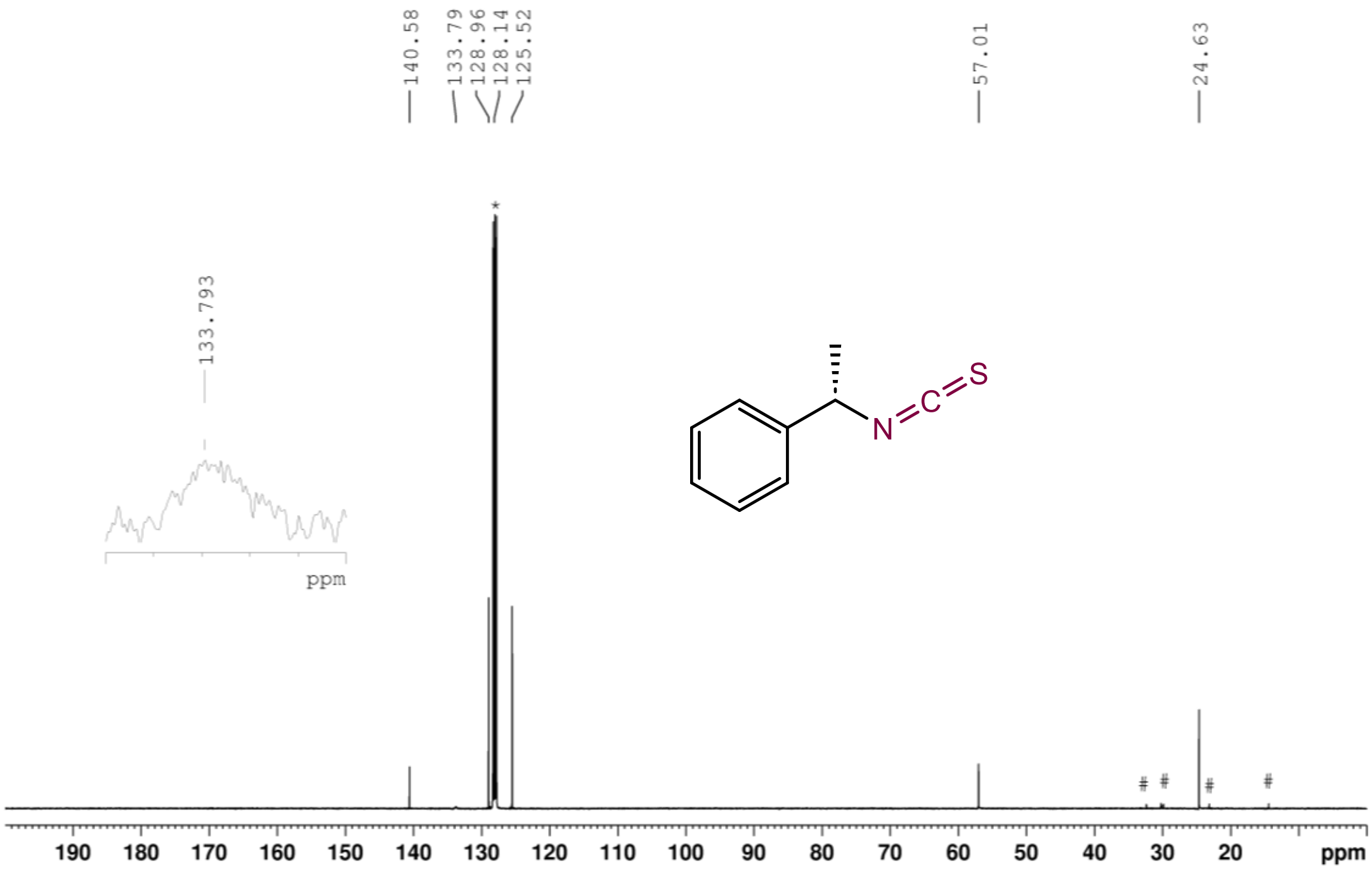

Figure S 55: ^{13}C NMR spectrum of (S)-(-)-α-Methylbenzylisothiocyanate (100 MHz, 298 K, benzene-d_6). Residual signals of deuterated solvent (*) and alkane impurities (#) are marked.

7 Derivatization of (S)-Methylbenzyl Isothiocyanate

In order to investigate whether the stereoinformation of the chiral substrate (S)-methylbenzyl isonitrile is retained during sulfur atom transfer as well as to demonstrate the feasibility of using the herein synthesized isothiocyanates for organic synthesis, a follow-up reaction was conducted. Following a literature-known procedure,[10] a J Young tube was charged with the (S)-(-)-α-methylbenzylisothiocyanate (24.0 mg, 147.0 µmol, 1 equiv.) as well as the chiral amine (S)-(-)-α-ethylbenzylamine (23.9 mg, 176.4 µmol, 1.2 equiv.) before adding benzene-d_6. The reaction was monitored via ^{1}H NMR spectroscopy and found complete after 2 hours at 23 °C.

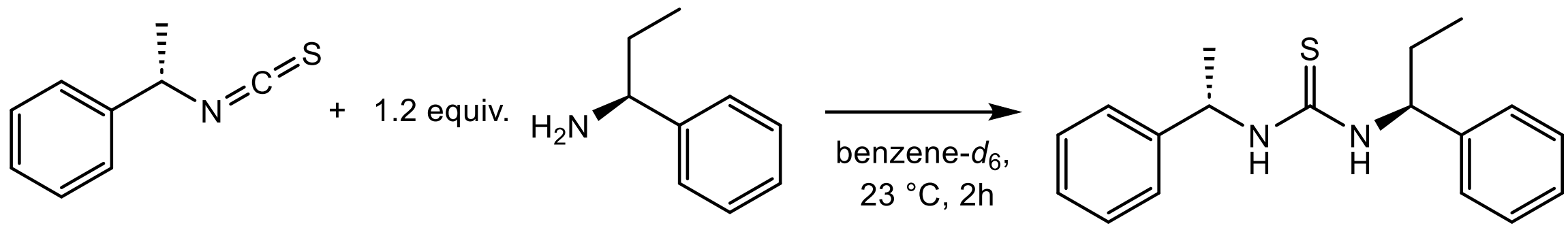

^{1}H NMR (600 MHz, 298 K, benzene-d_6) δ = 0.69 (t, ^3J = 7.3 Hz, 3H, CH_{3CH2}), 1.06 (s, br, 1H, NH), 1.33 (d, ^3J = 6.7 Hz, 3H, CH_{3CH}), 1.35-1.31 (m, 2H, CH_2) 1.55-1.48 (m, overlapping with (S)-(-)-α-ethylbenzylamine signal, assigned via ^{1}H,^{1}H-COSY, 1H, CH_{CH3}), 1.72-1.63 (m, 1H, CH_{CH2}), 5.22 (s, br, 2H, NH), 6.93-6.88 (m, 4H, CH_{Ar}), 7.01-6.98 (m, 6H, CH_{Ar}) ppm.

^{13}C NMR {H} (151 MHz, 298 K, benzene-d_6) δ = 10.67 (s, CH_3, CH$_{3CH2}$), 22.89 (s, CH_3, CH$_{3CH}$), 30.16 (s, CH_2, CH$_2$), 54.16 (s, CH, CH$_{CH3}$), 60.13 (s, CH, CH$_{CH2}$), 136.27 (s, CH, CH$_{Ar}$), 126.94 (s, CH, CH$_{Ar}$), 127.41 (s, br, CH$_{Ar}$), 127.41 (s, br, CH, CH$_{Ar}$), 128.94 (s, br, CH, CH$_{Ar}$), 128.99 (s, br, CH, CH$_{Ar}$), 142.22 (s, C_q, C_{qAr}), 143.58 (s, C_q, C_{qAr}), 181.49 (s, br, C_q, CS) ppm.

Only one diastereomer was detected in both the ^{1}H NMR as well as the ^{13}C NMR spectra.

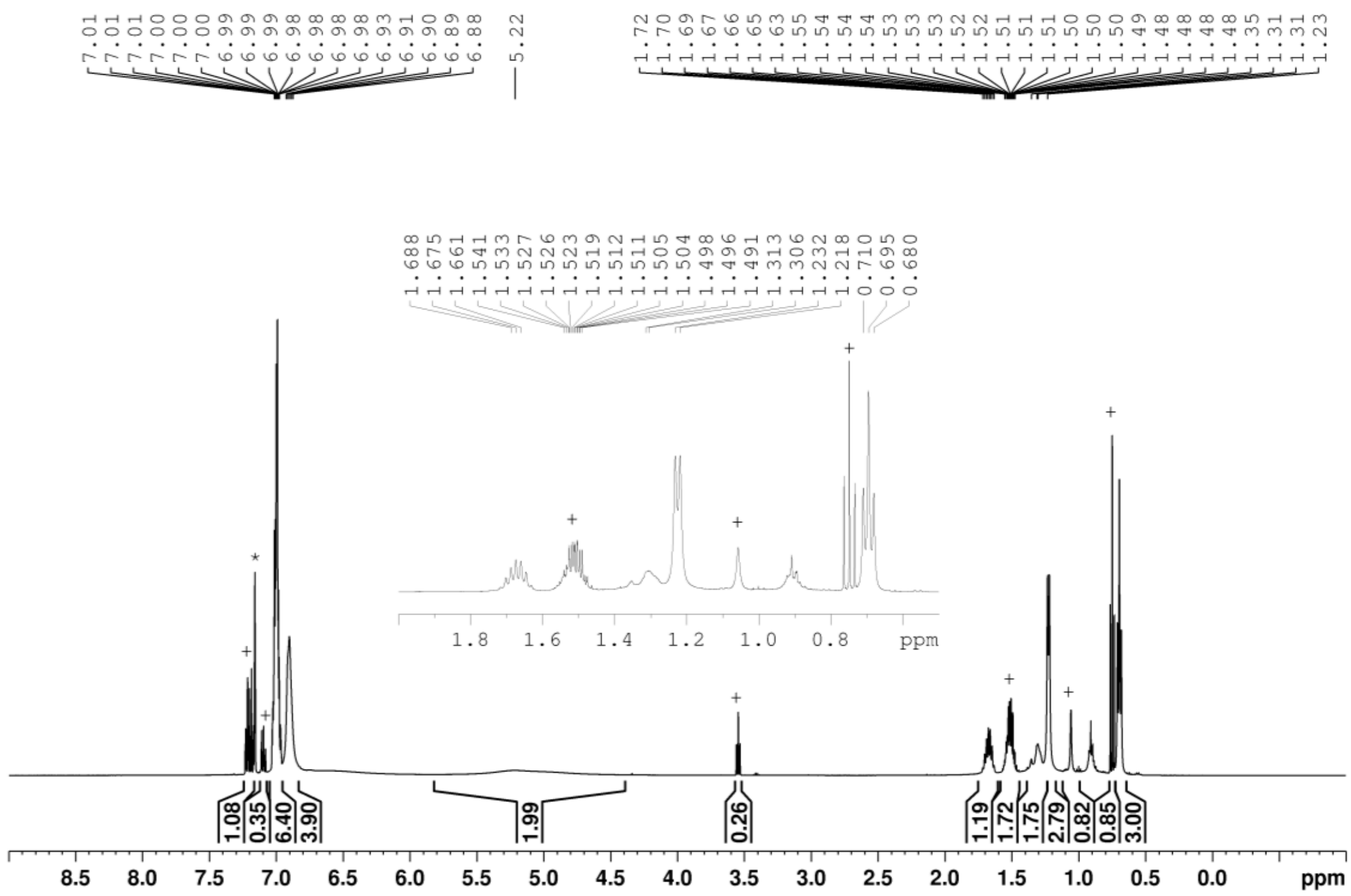

Figure S 56: ^{1}H NMR spectrum of (S)-(-)-α-Methylbenzyl-(S)-(-)-α-ethylbenzylthiourea (600 MHz, 298 K, benzene-d_6). Residual signals of deuterated solvent (*) and starting material -(S)-(-)-α-ethylbenzylamine (+) are marked.

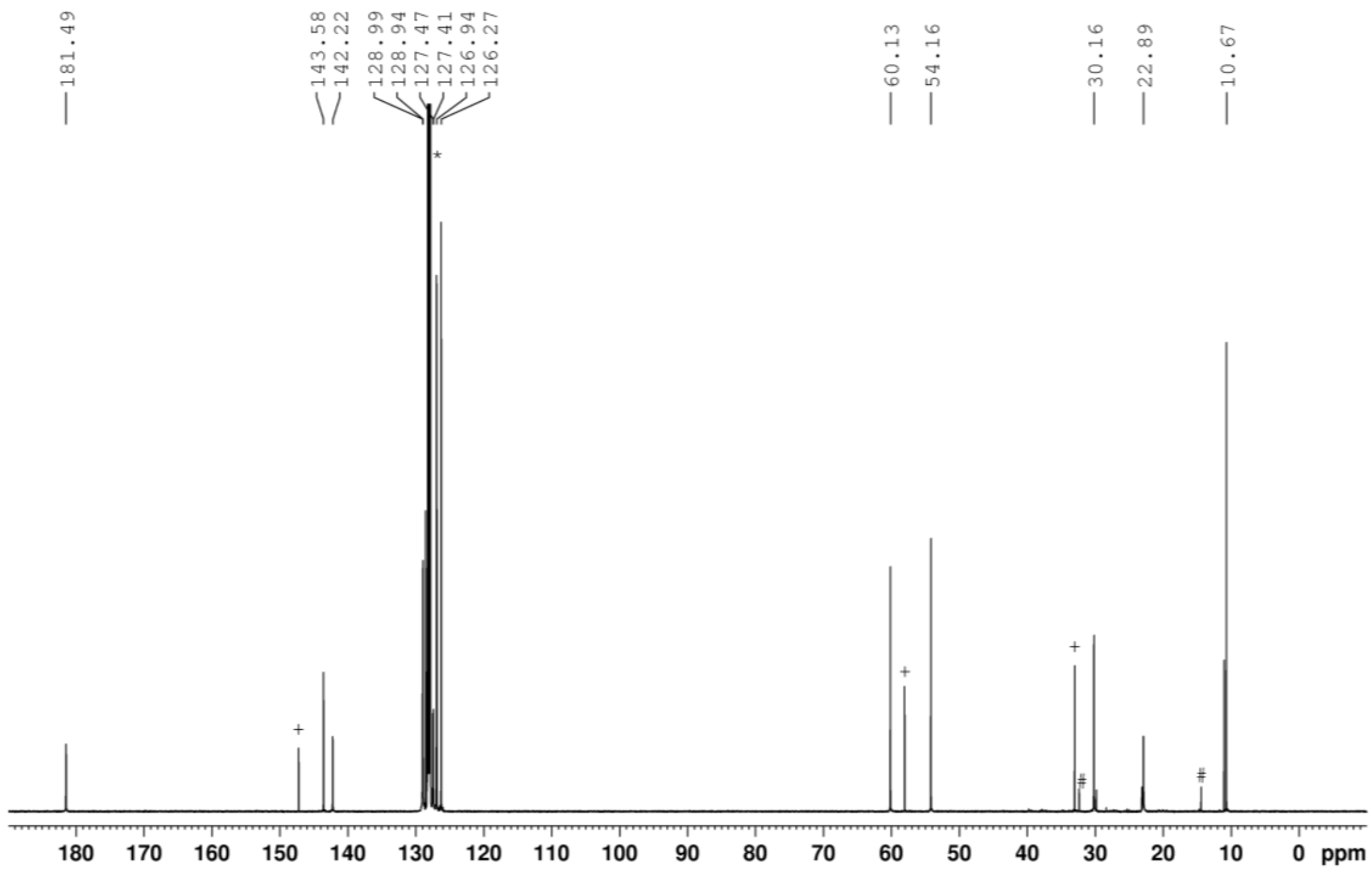

Figure S 57: ^{13}C NMR spectrum of (S)-(-)-α-Methylbenzyl-(S)-(-)-α-ethylbenzylthiourea (151 MHz, 298 K, benzene-d_6). Residual signals of deuterated solvent (*), alkane impurities (#)and starting material -(S)-(-)-α-ethylbenzylamine (#) are marked.

8 Single-Turnover Experiments

A J Young tube was charged with (^{Et}PDI)Ti(S_2)$_2$ (5.0 mg, 8.3 µmol, 1 equiv.), *tert*-butyl isocyanide (8.31 µL of a 1M stock solution in benzene-d_6, 8.31 µmol, 1 equiv.) HMDSO (5 µL, 23.5 µmol) as an internal standard, benzene-d_6 (250 µL) and TFT (250 µL). The reaction was conducted at 23 °C and monitored via ^{1}H-NMR spectroscopy. Full conversion to the isothiocyanate was observed after 2 hours (Figure S 58).

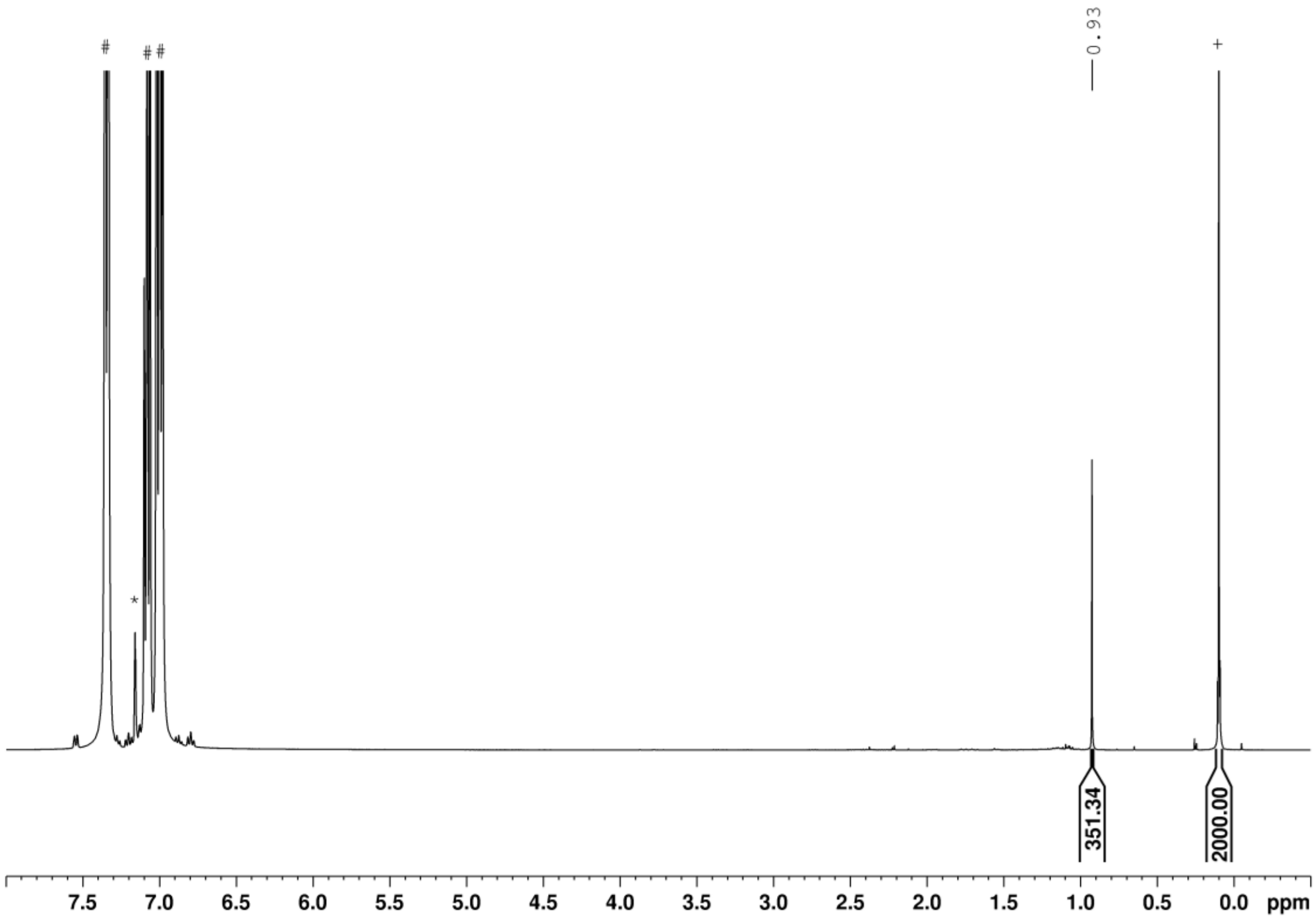

Figure S 58. ^{1}H NMR spectrum (400 MHz, 298 K, benzene-d_6) of one turnover of ^{Et}PDITi(S_2)$_2$ with 1 equiv *tert*-butyl isocycanide after 2 hours. Residual signals of deuterated solvent (*), TFT (#) and HMDSO (+) are marked.

Upon addition of a second equivalent of the isonitrile, 75% conversion was observed after a reaction time of 48 hours (Figure S 59).

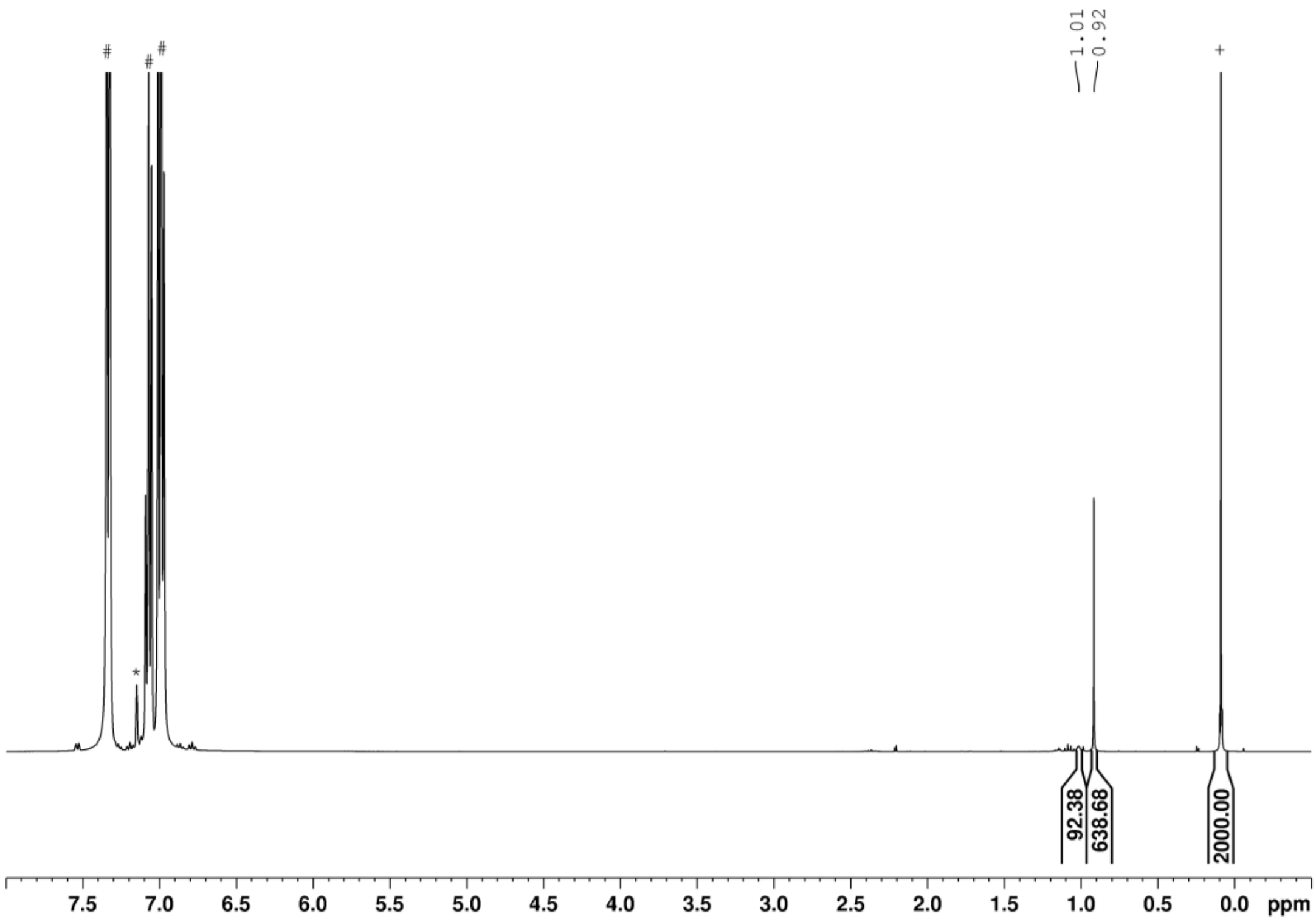

Figure S 59. ^{1}H NMR spectrum (400 MHz, 298 K, benzene-d_6) of one a second turnover of $^{Et}PDITi(S_2)_2$ with 2-Isothiocyanato-2-methylpropane after 48 hours. Residual signals of deuterated solvent (*), TFT (#) and HMDSO (+) are marked.

In an attempt to gain insights into the titanium complex after sulfur atom transfer, stoichiometric sulfur atom abstraction reactions were carried out using different reagents. Therefore, a J Young tube was charged with $(^{Et}PDI)Ti(S_2)_2$ (14.7 mg, 24.4 µmol, 1 equiv.) and either *tert*-butyl isocyanide (2.0 mg, 24.4 µmol, 1 equiv.) or PMe_3 (1.8 mg, 24.4 µmol, 1 equiv.) before adding HMDSO (5 µL, 23.525 µmol) as an internal standard as well as MeCN-d_3 (500 µL) and the reactions were conducted at 23 °C. ^{1}H-NMR spectroscopy revealed no selective formation of Ti-containing species and crystallization attempts afforded the free ligand. Instead, the ^{1}H NMR spectra of both substrates show significant decomposition of the titanium complex, resulting in the formation of free ligand (**Figure S *60*:** ^{1}H NMR spectrum (400 MHz, 298 K, MeCN-d_3) of the stoichiometric sulfur abstraction from $(^{Et}PDI)Ti(S_2)_2$ with $^{t}BuNC$ and PMe_3.Figure S 60, Figure S 61, Figure S 62).

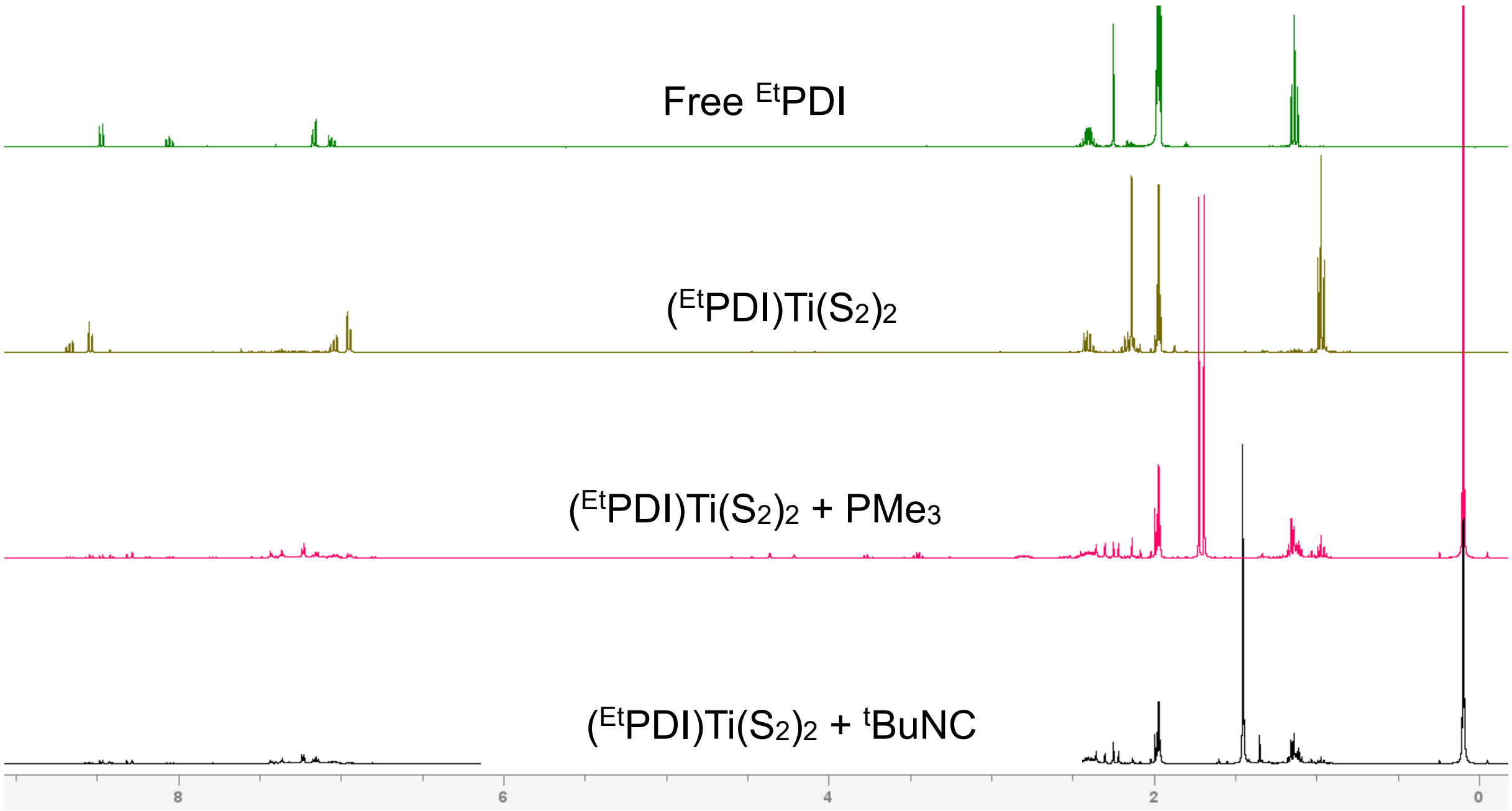

Figure S 60: ^{1}H NMR spectrum (400 MHz, 298 K, MeCN-d_3) of the stoichiometric sulfur abstraction from (^{Et}PDI)Ti(S_2)$_2$ with ^{t}BuNC and PMe_3.

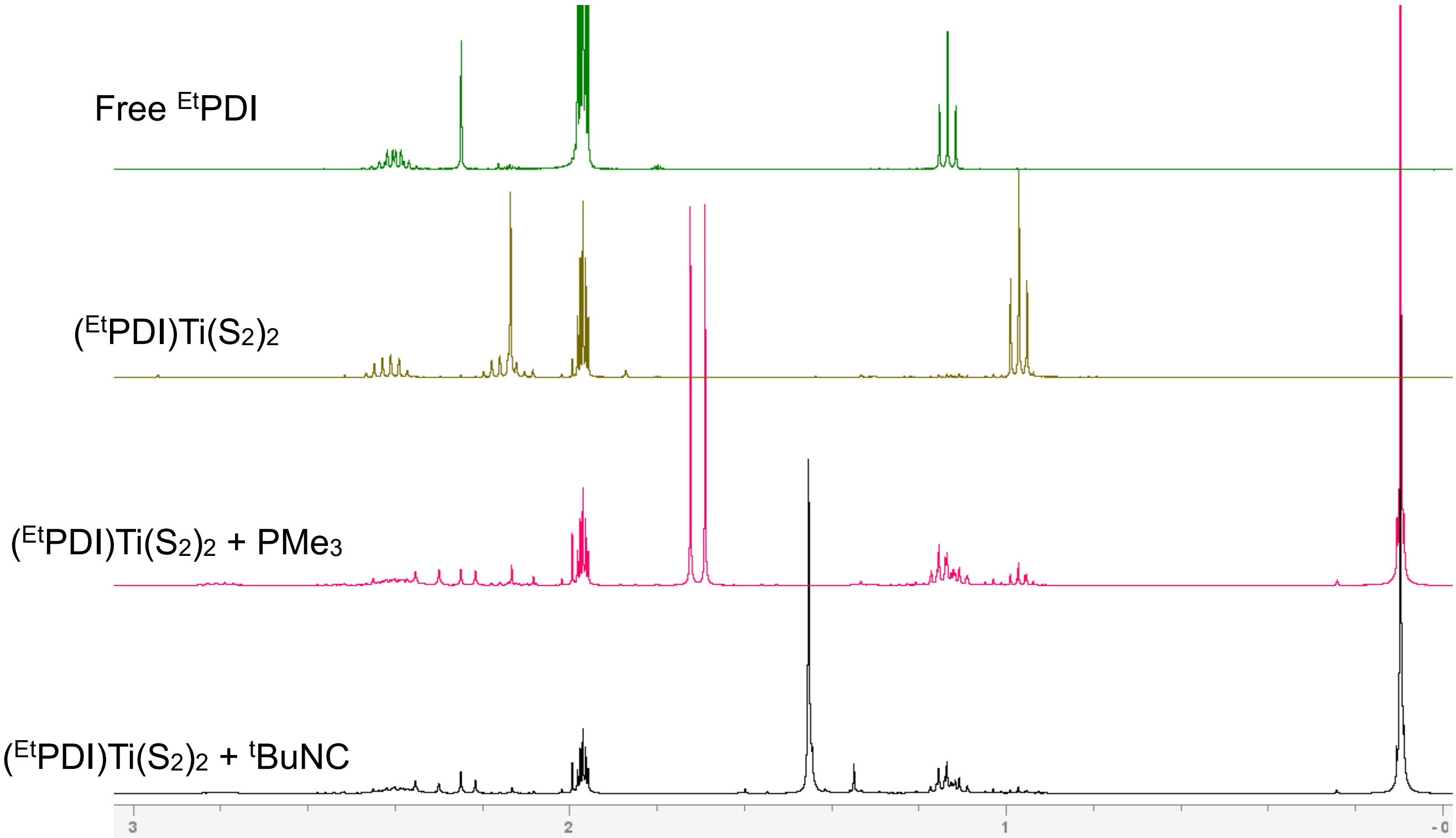

Figure S 61: Excerpt of the ^{1}H NMR spectrum (400 MHz, 298 K, MeCN-d_3) of the stoichiometric sulfur abstraction from (^{Et}PDI)Ti(S_2)$_2$ with ^{t}BuNC and PMe_3.

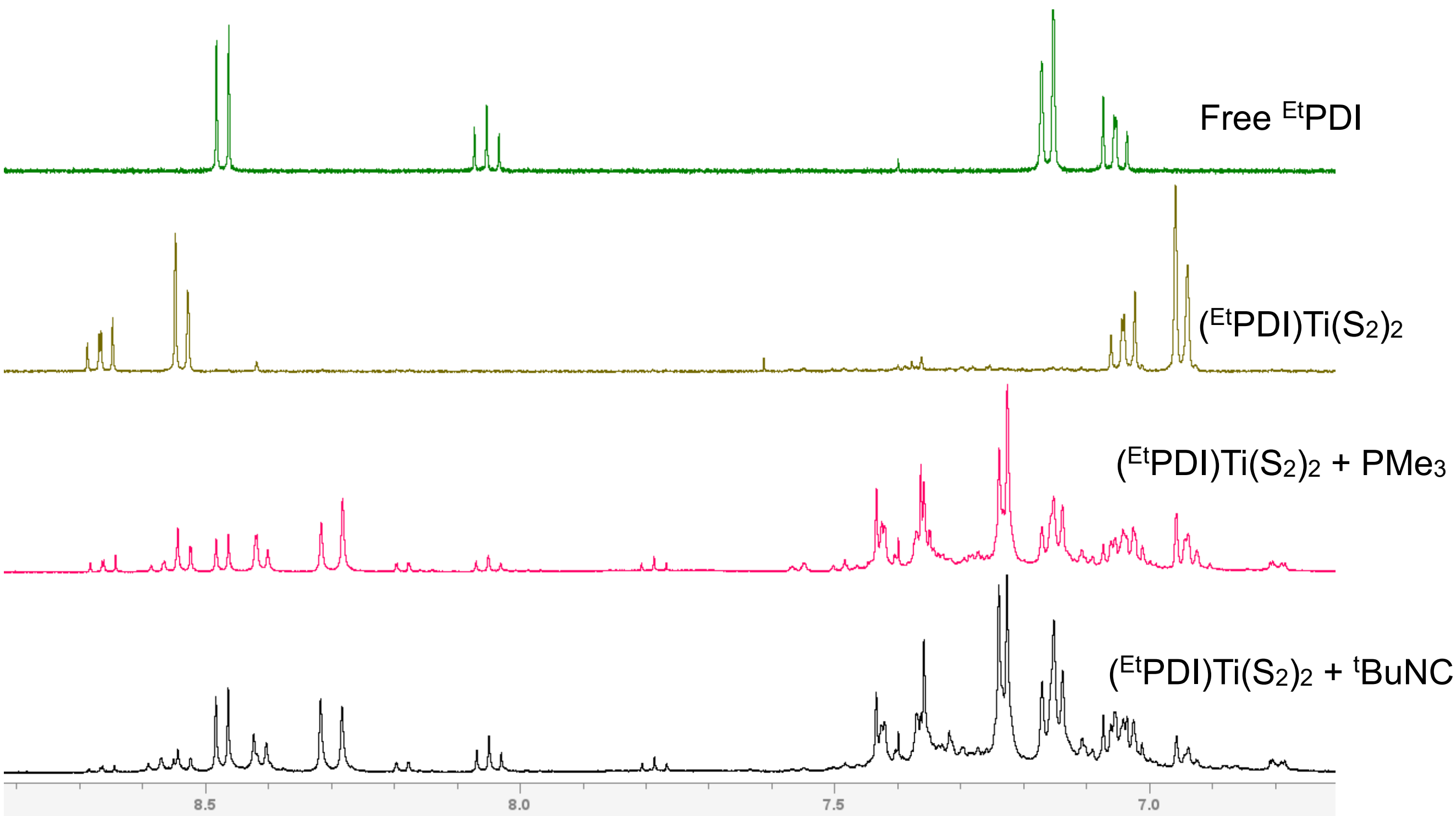

Figure S 62: Excerpt of the ^{1}H NMR spectrum (400 MHz, 298 K, MeCN-d_3) of the stoichiometric sulfur abstraction from (^{Et}PDI)Ti(S_2)$_2$ with ^{t}BuNC and PMe_3.

9 Kinetic Studies

Substrates

A stock solution of (^{Et}PDI)Ti(CH_2Ph)$_2$ (100 mg) in benzene-d_6 (2 mL) was prepared in an argon glovebox. A J Young tube was charged with S_8 (9.8 mg, 38.2 µmol, 0.25 equiv.), the stock solution of ^{Et}PDITi(CH_2Ph)$_2$ (100 µL, 7.6 µmol, 0.05 equiv.), benzene-d_6 (150 µL), TFT (250 µL), HMDSO (5 µL) and the respective isonitrile (^{t}BuNC: 17.3 µL, 152.5 µmol, 1 equiv.; ^{i}PrNC: 14.4 µL, 152.5 µmol, 1 equiv.; AdNC: 24.6 mg, 152.5 µmol, 1 equiv.) and product formation was monitored by ^{1}H NMR spectroscopy. The variance in time regarding the first data point is a result of different lock and shim duration of the NMR spectrometer.

Table S1. NMR Data Points for the plots of the different isonitrile substrates. The concentrations were obtained using HMDSO as internal standard.

time [h]	[^{t}BuNCS] [mmol/L]	time [h]	[^{i}PrNCS] [mmol/L]	time [h]	[AdNCS] [mmol/L]
0.37	11.77	0.32	8.01	0.37	36.71
0.5	16.30	0.5	11.58	0.5	50.28
0.75	25.37	0.75	16.07	0.75	71.56
1	33.97	1	20.29	1	89.47
1.25	42.11	1.25	24.19	1.25	104.54
1.5	49.82	1.5	27.91	1.5	117.58
1.75	57.08	1.75	31.42	1.75	129.01
2	63.81	2	34.77	2	138.74
2.25	70.02	2.25	37.99	2.25	147.06
2.5	75.94	2.5	41.09	2.5	153.38

In order to further compare the substrates, the initial reaction rates k_{obs} were determined by plotting the first four data points and determining the slope of a linear fit.

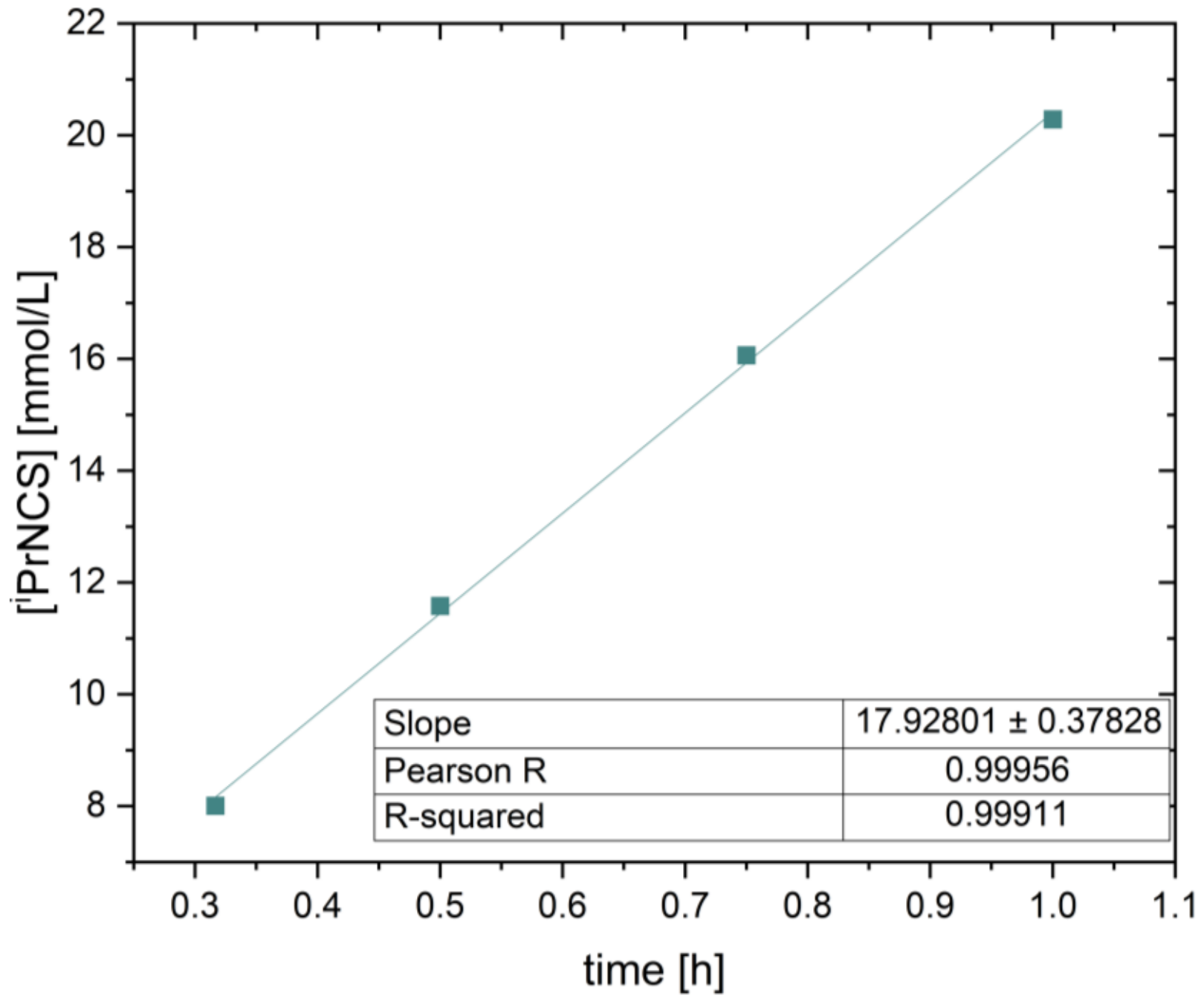

Figure S 63. Initial rate plot for the formation of ^{i}PrNCS.

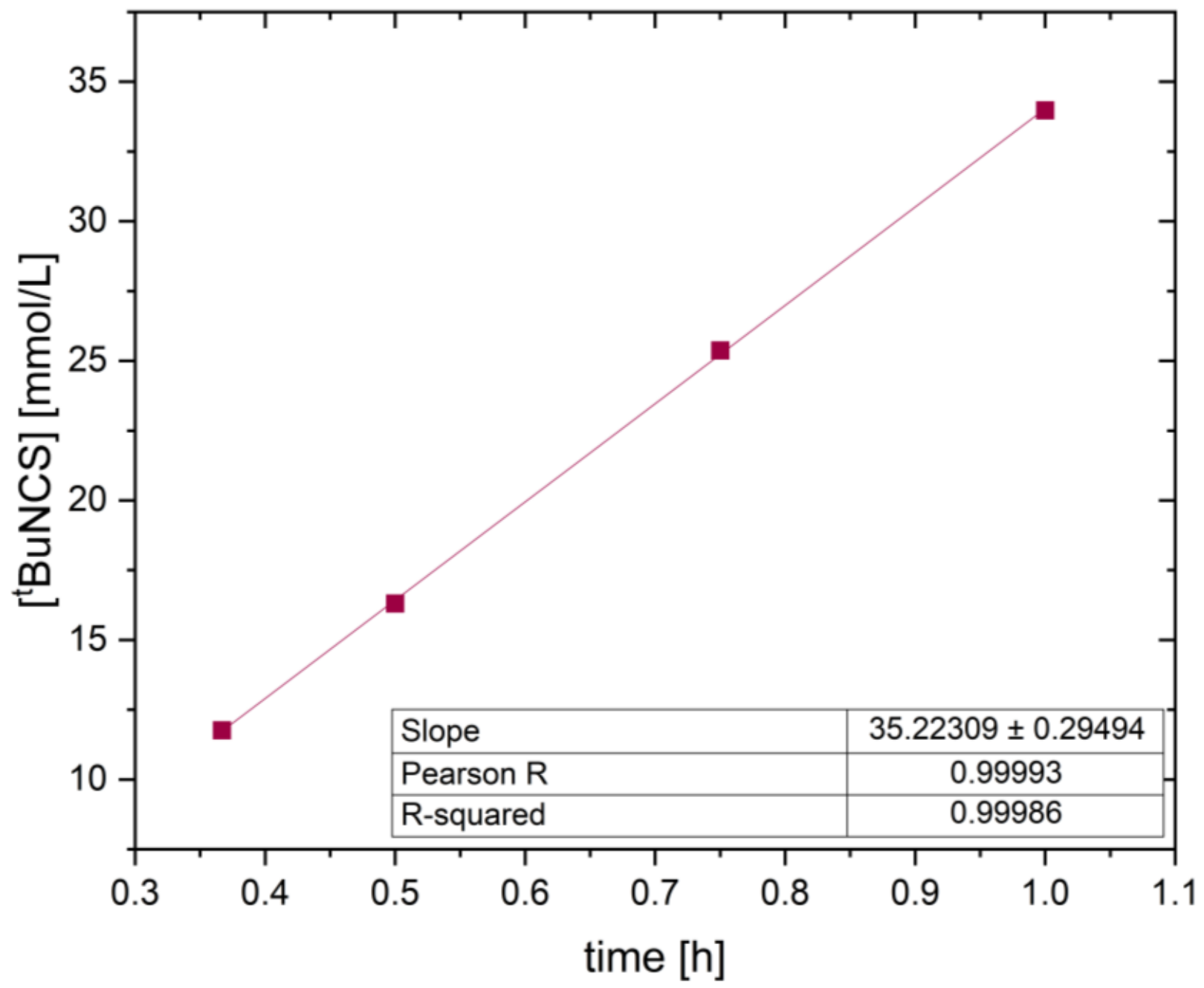

Figure S 64. Initial rate plot for the formation of ^{t}BuNCS.

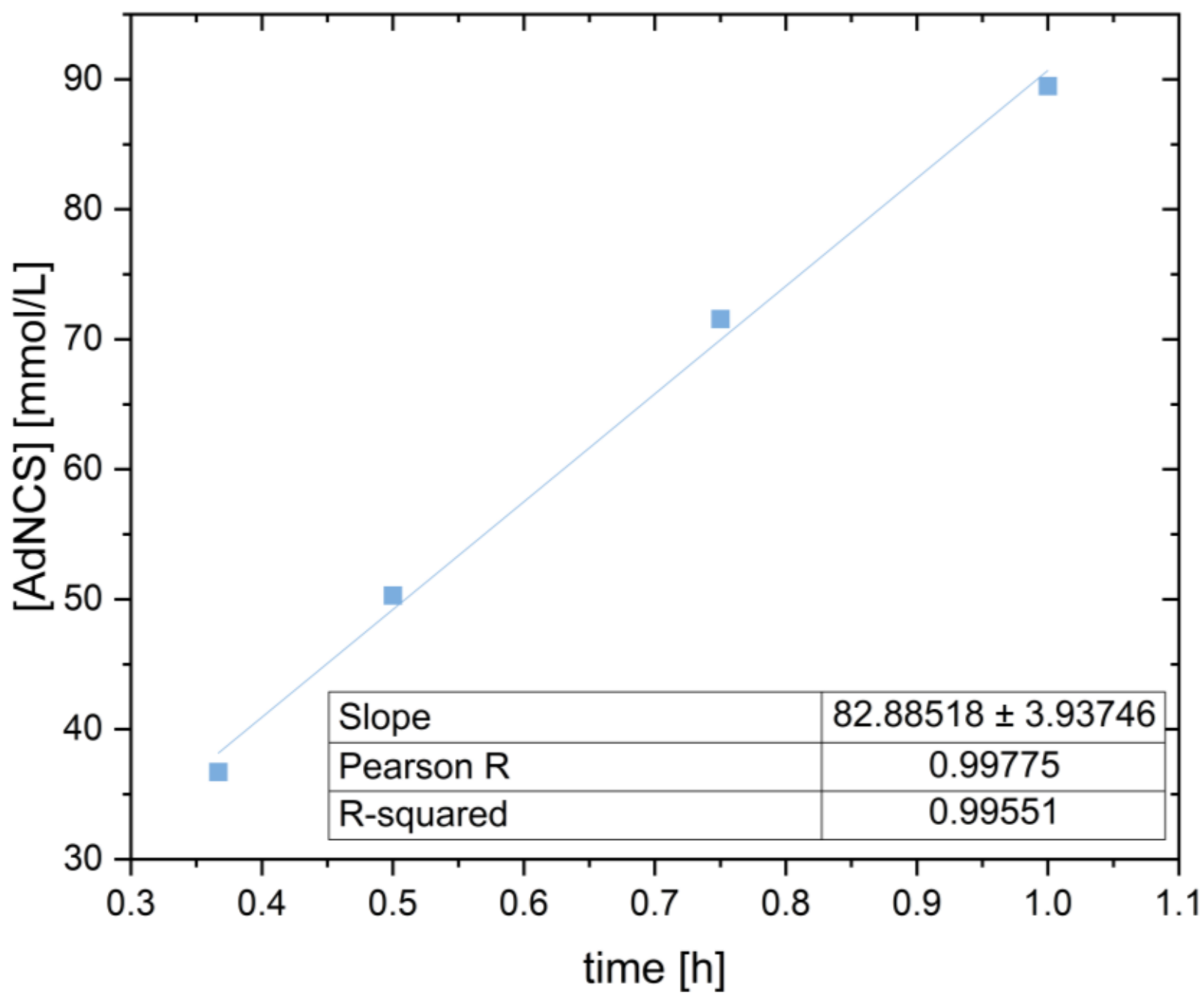

Figure S 65. Initial rate plot for the formation of AdNCS.

Order in Ti

A J Young tube was charged with S_8 (9.8 mg, 38.2 µmol), the respective amount of stock solution of ^{Et}PDITi(CH_2Ph)$_2$ (100 µL, 7.6 µmol), benzene-d_6 (150 µL), TFT (250 µL), HMDSO (5 µL) and ^{t}BuNC (17.3 µL, 152.5 µmol). The formation of ^{t}BuNCS was monitored by ^{1}H NMR spectroscopy. Isothiocyanate formation was plotted using the initial rates method in order to obtain the initial reaction rates k_{obs}.

[Ti] = 3.06 mmol/L		[Ti] = 9.16 mmol/L	
time [h]	[^{t}BuNCS] [mmol/L]	time [h]	[^{t}BuNCS] [mmol/L]
0.33	2.26	0.30	5.62
0.5	3.09	0.5	9.77
0.75	4.24	0.75	15.07
1	5.39	1	19.90
[Ti] = 15.26 mmol/L		[Ti] = 21.36 mmol/L	
time [h]	[^{t}BuNCS] [mmol/L]	time [h]	[^{t}BuNCS] [mmol/L]
0.37	11.77	0.33	20.45
0.5	16.30	0.5	27.98
0.75	25.37	0.75	41.04
1	33.97	1	53.39

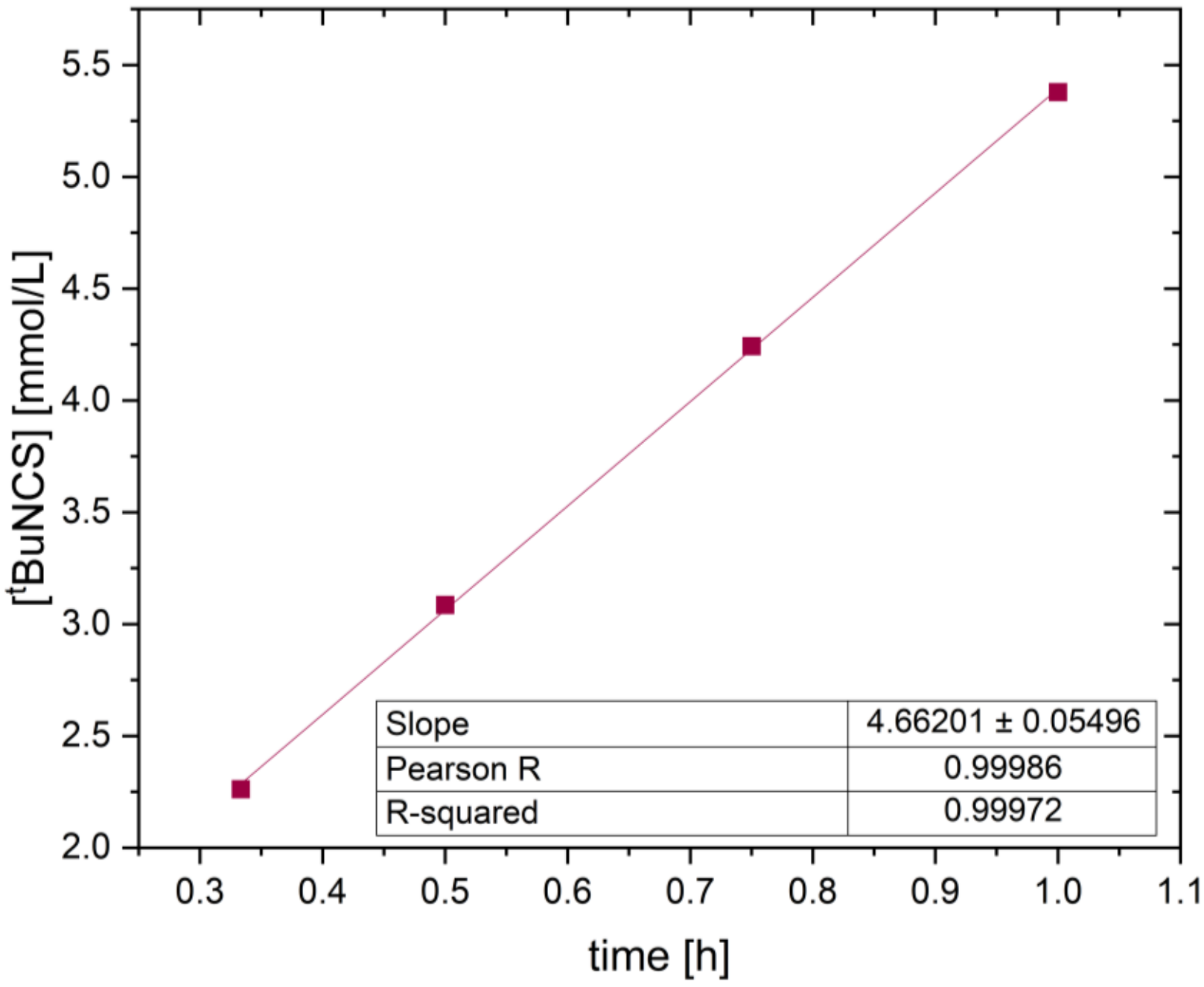

Figure S 66. Initial rate plot with [Ti] = 3.06 mmol/L.

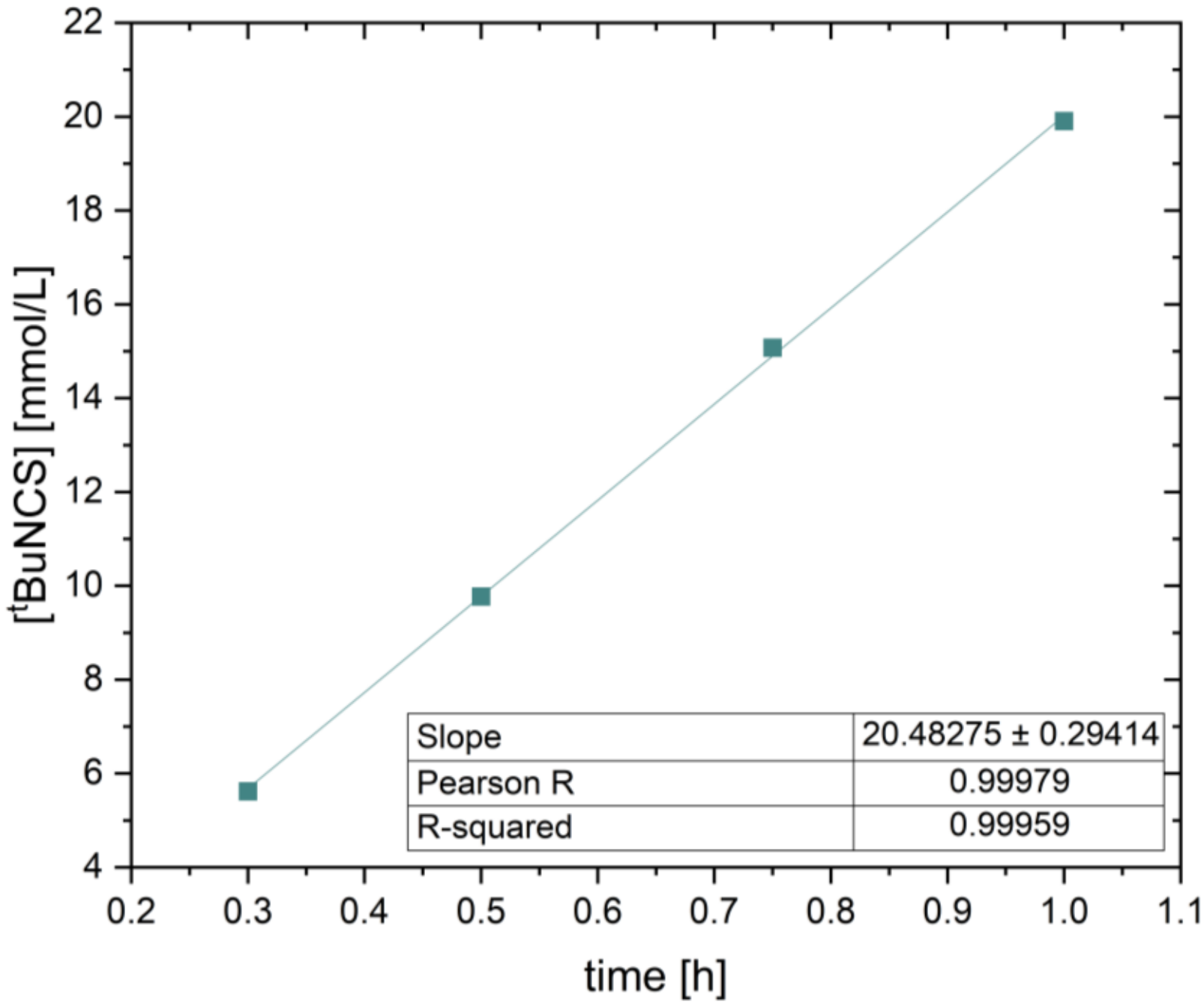

Figure S 67. Initial rate plot with [Ti] = 9.16 mmol/L.

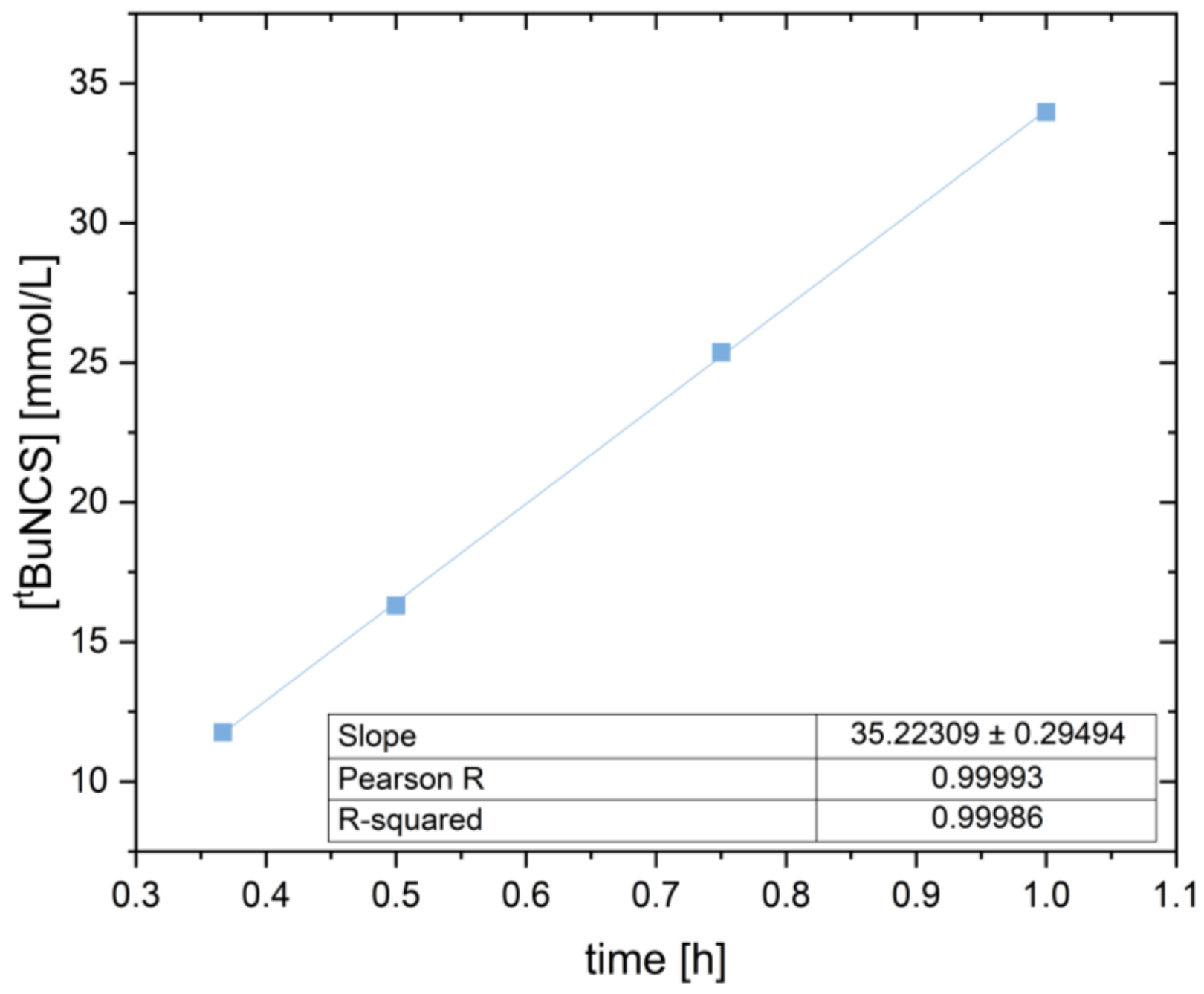

Figure S 68. Initial rate plot with [Ti] = 15.26 mmol/L.

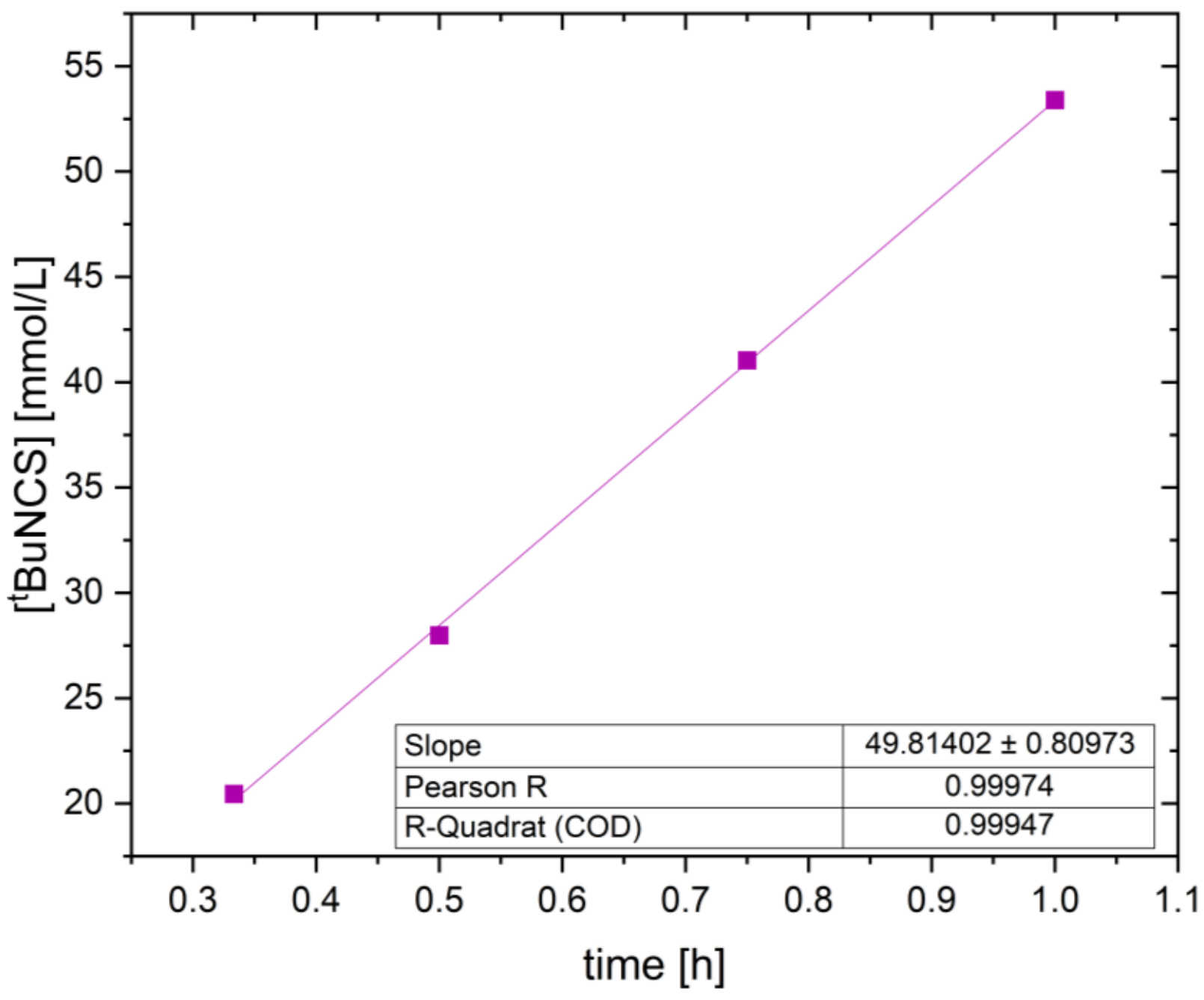

Figure S 69. Initial rate plot with [Ti] = 21.36 mmol/L.

The order in [Ti] was determined by plotting the resulting values for k_{obs} against the respective concentration [Ti], confirming a first order in [Ti] due to the linear correlation between k_{obs} and [Ti].

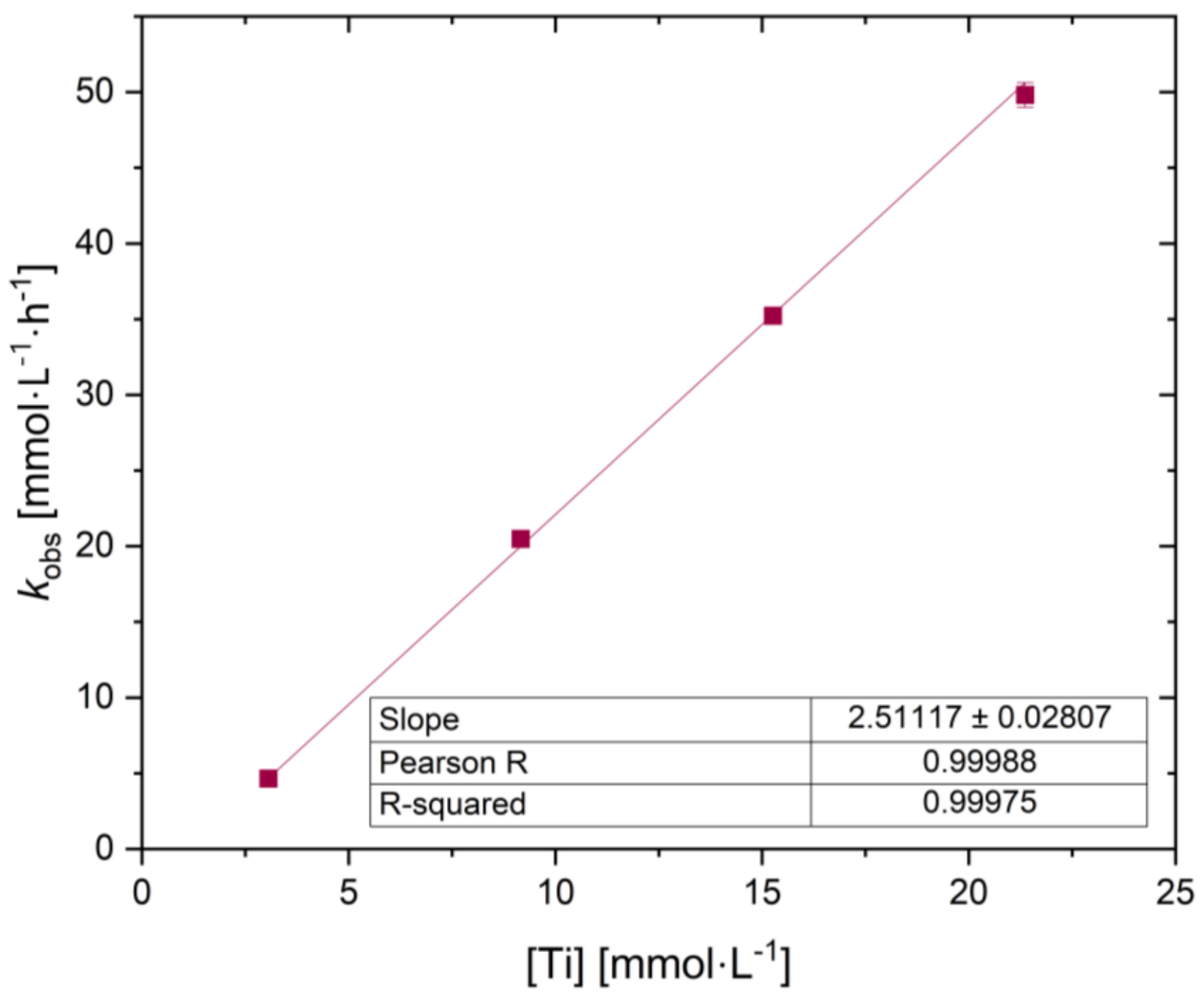

Figure S 70. Linear correlation between k_{obs} and [Ti].

Order in Isonitrile

A J Young tube was charged with S_8 (9.8 mg, 38.2 µmol), stock solution of ^{Et}PDITi(CH_2Ph)$_2$ (100 µL, 7.6 µmol,), benzene-d_6 (150 µL), TFT (250 µL), HMDSO (5 µL) and the respective amount of ^{t}BuNC (17.3 µL, 152.5 µmol, 1 equiv.). Isothiocyanate formation was plotted using the initial rates method in order to obtain the initial reaction rates k_{obs}.

[^{t}BuNC] = 203.32 mmol/L		[^{t}BuNC] = 305.00 mmol/L	
time [h]	[^{t}BuNCS] [mmol/L]	time [h]	[^{t}BuNCS] [mmol/L]
0.33	9.76	0.37	11.77
0.5	14.33	0.5	16.30
0.75	20.27	0.75	25.37
1	26.21	1	33.97
[^{t}BuNC] = 355.82 mmol/L		[^{t}BuNC] = 406.60 mmol/L	
time [h]	[^{t}BuNCS] [mmol/L]	time [h]	[^{t}BuNCS] [mmol/L]
0.35	9.01	0.33	8.08
0.5	15.65	0.5	16.08
0.75	25.35	0.75	28.00
1	35.90	1	39.69

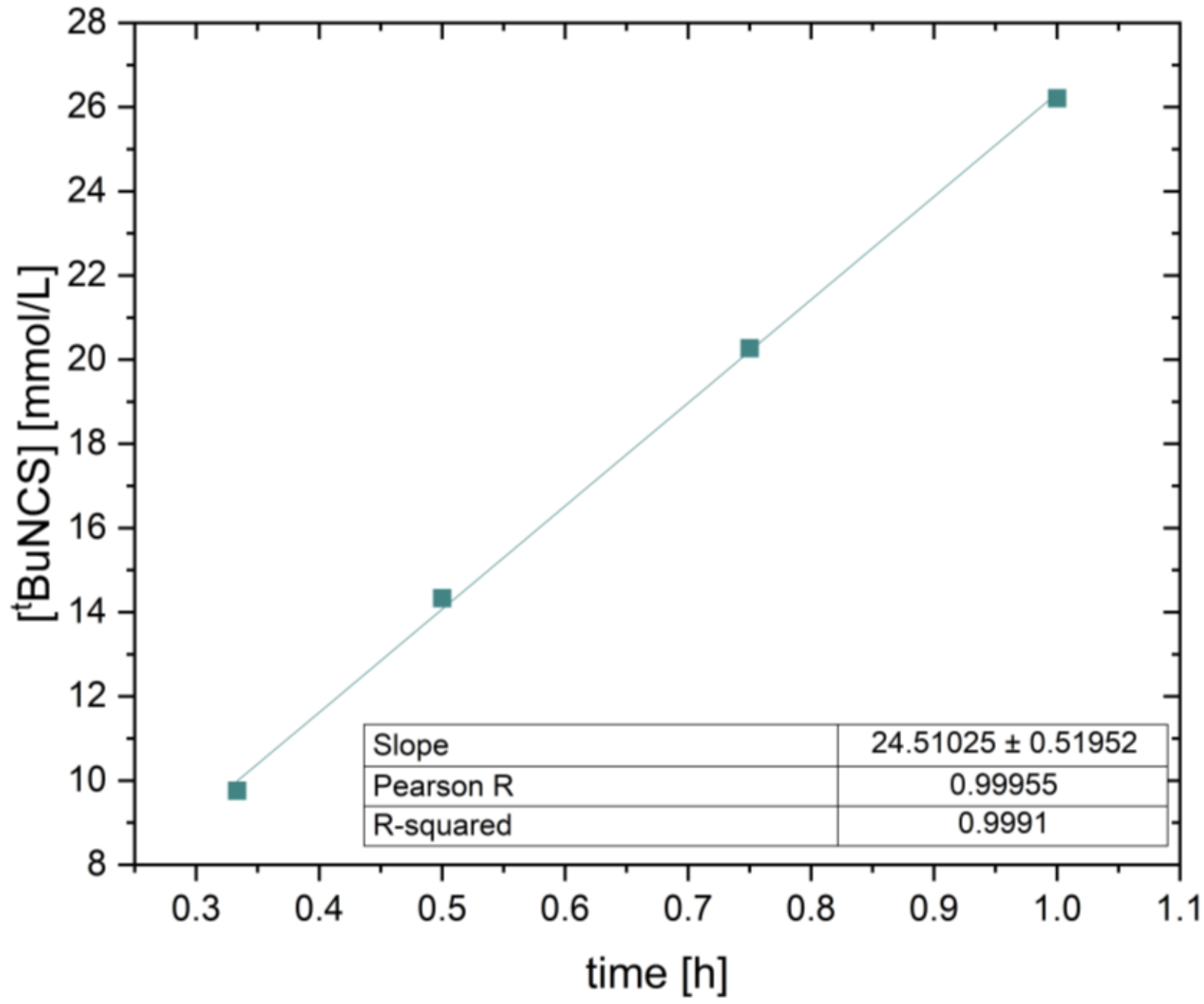

Figure S 71. Initial rate plot with [^{t}BuNC] = 203.32 mmol/L.

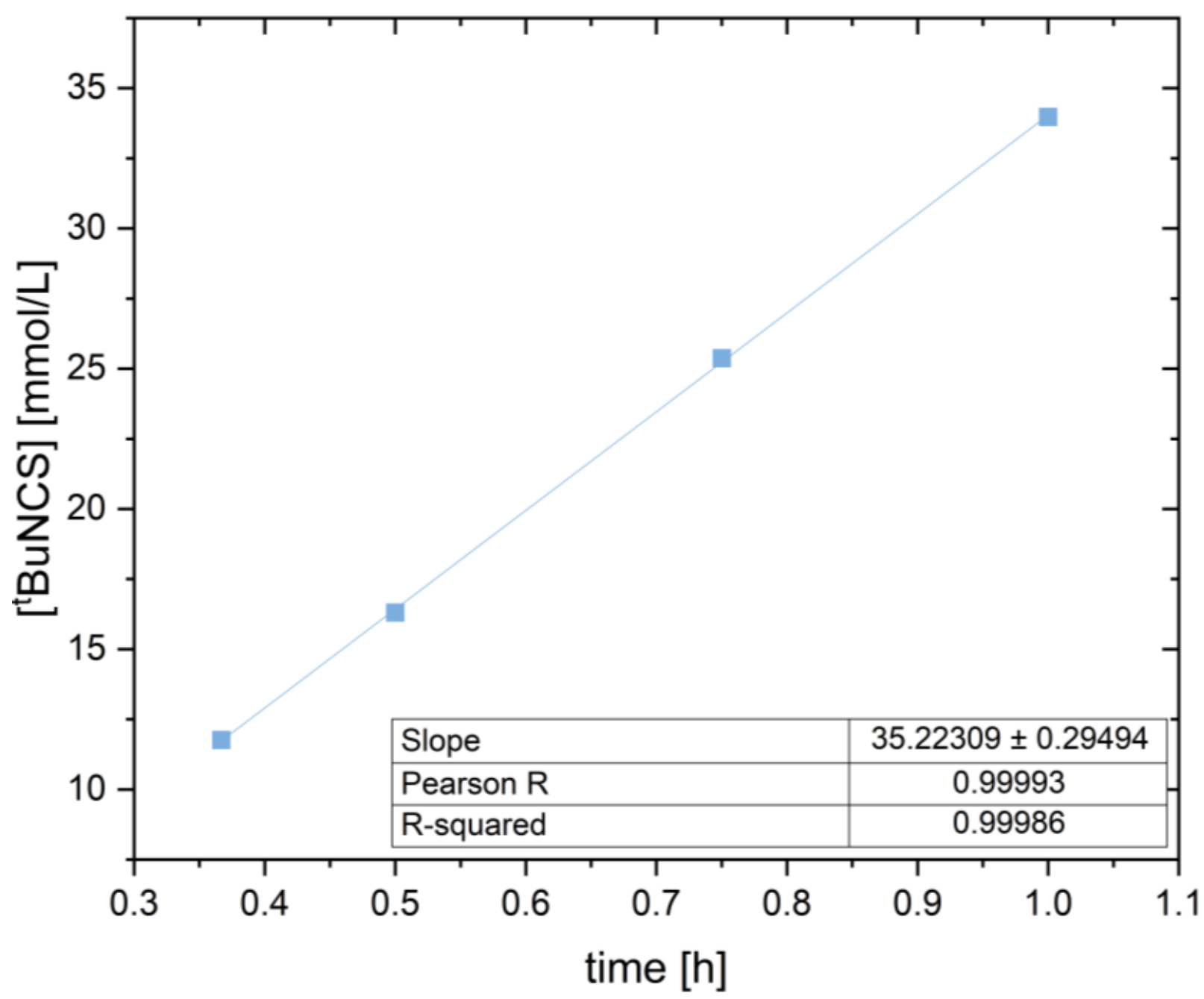

Figure S 72. Initial rate plot with [^{t}BuNC] = 305.00 mmol/L.

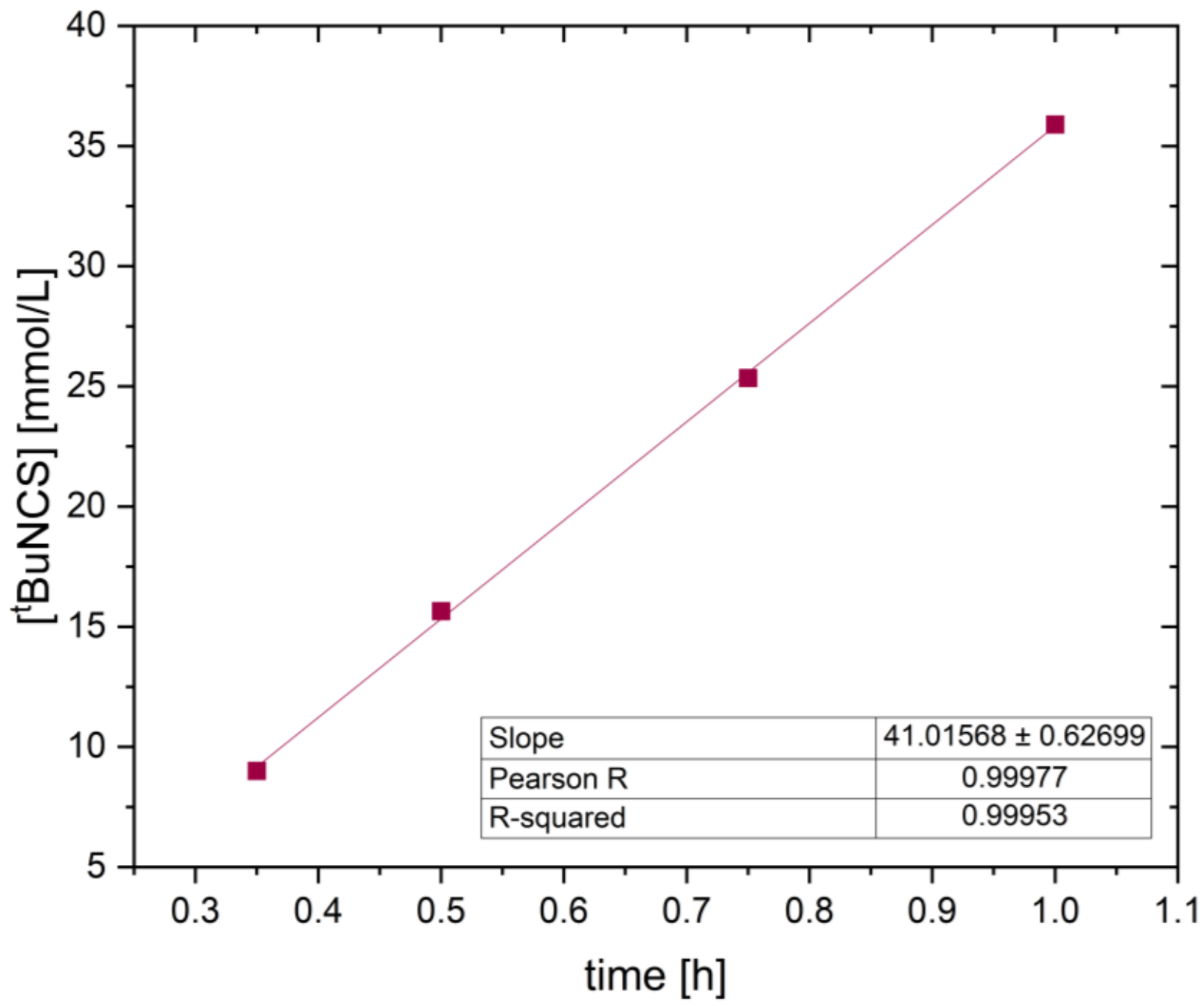

Figure S 73. Initial rate plot with [^{t}BuNC] = 355.82 mmol/L.

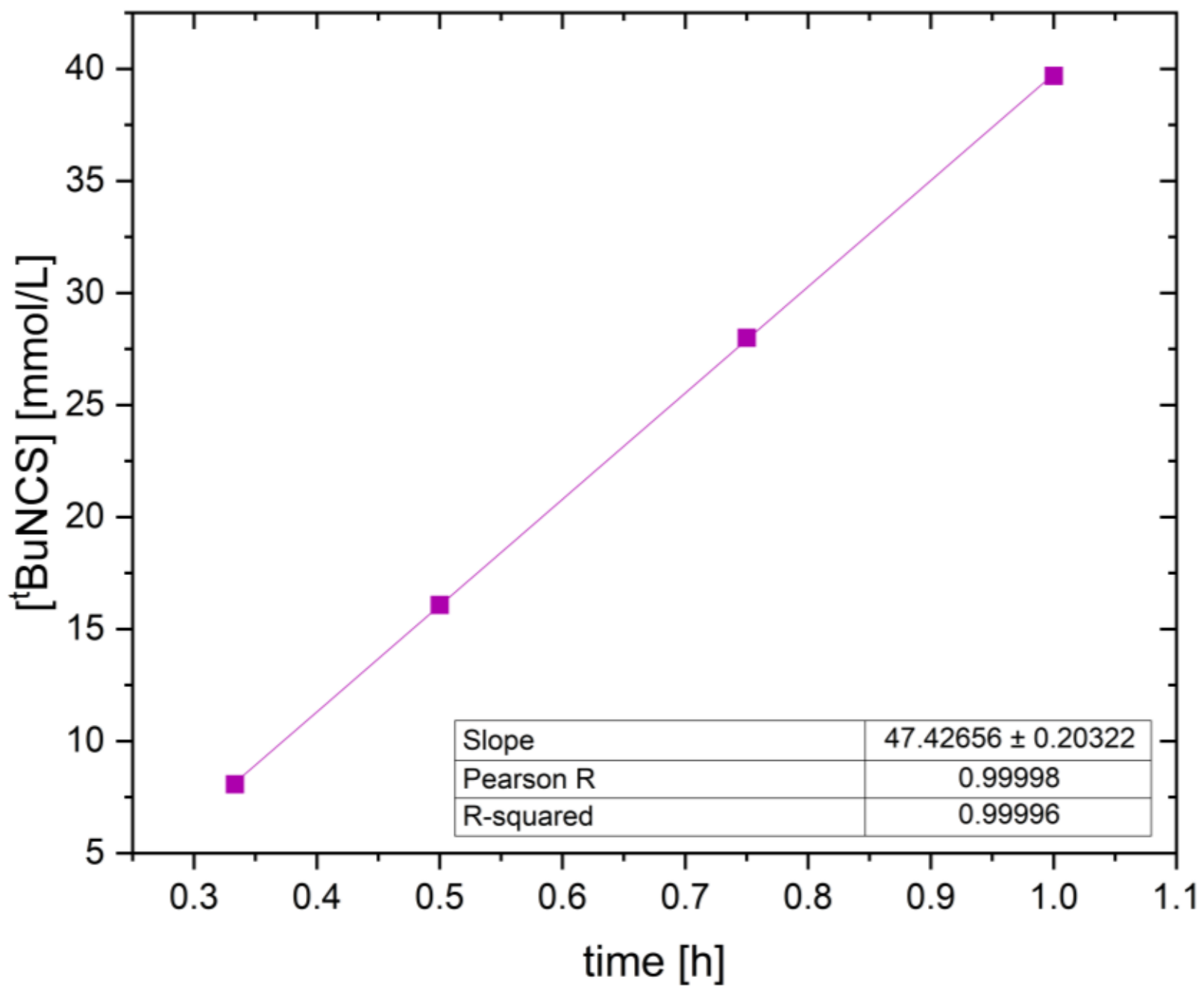

Figure S 74. Initial rate plot with [^{t}BuNC] = 406.60 mmol/L.

The order in [^{t}BuNC] was determined by plotting the resulting values for k_{obs} against the respective concentration [^{t}BuNC], confirming a first order in [^{t}BuNC] due to the linear correlation between k_{obs} and [^{t}BuNC].

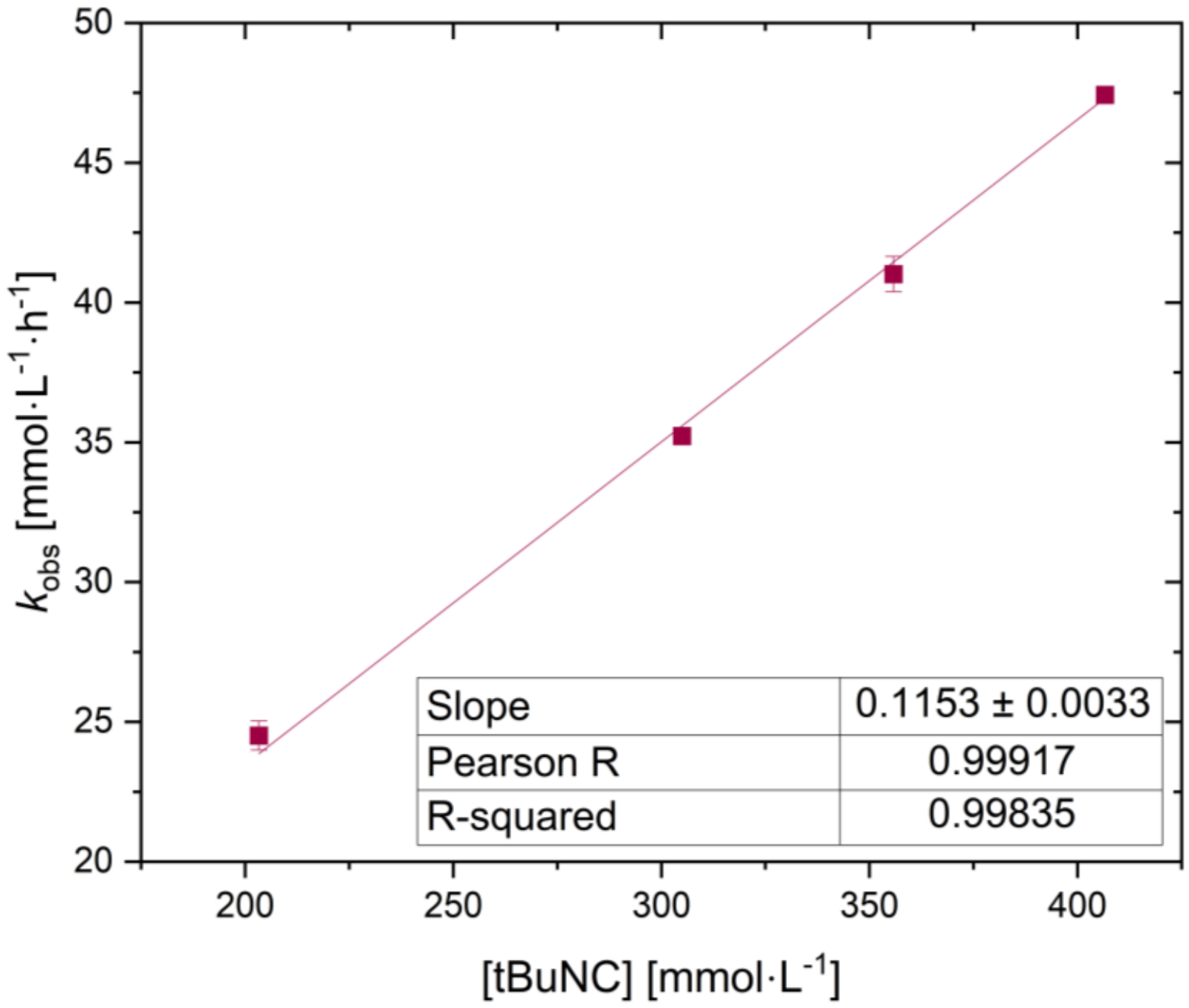

Figure S 75. Linear correlation between k_{obs} and [^{t}BuNC].

Order in Sulfur

A J Young tube was charged with S_8 (9.8 mg, 38.2 µmol), the respective amount of stock solution of ^{Et}PDITi(CH_2Ph)$_2$ (100 µL, 7.6 µmol), benzene-d_6 (150 µL), TFT (250 µL), HMDSO (5 µL) and ^{t}BuNC (17.3 µL, 152.5 µmol). Isothiocyanate formation was plotted using the initial rates method in order to obtain the initial reaction rates k_{obs}.

[S_8] = 57.18 mmol/L		[S_8] = 76.26 mmol/L	
time [h]	[^{t}BuNCS] [mmol/L]	time [h]	[^{t}BuNCS] [mmol/L]
0.28	9.65	0.37	11.77
0.5	17.93	0.5	16.30
0.75	27.07	0.75	25.37
1	35.51	1	33.97
[S_8] = 95.32 mmol/L		[S_8] = 114.38 mmol/L	
time [h]	[^{t}BuNCS] [mmol/L]	time [h]	[^{t}BuNCS] [mmol/L]
0.30	9.57	0.25	9.54
0.5	16.74	0.5	18.41
0.75	25.03	0.75	27.70
1	32.90	1	36.61

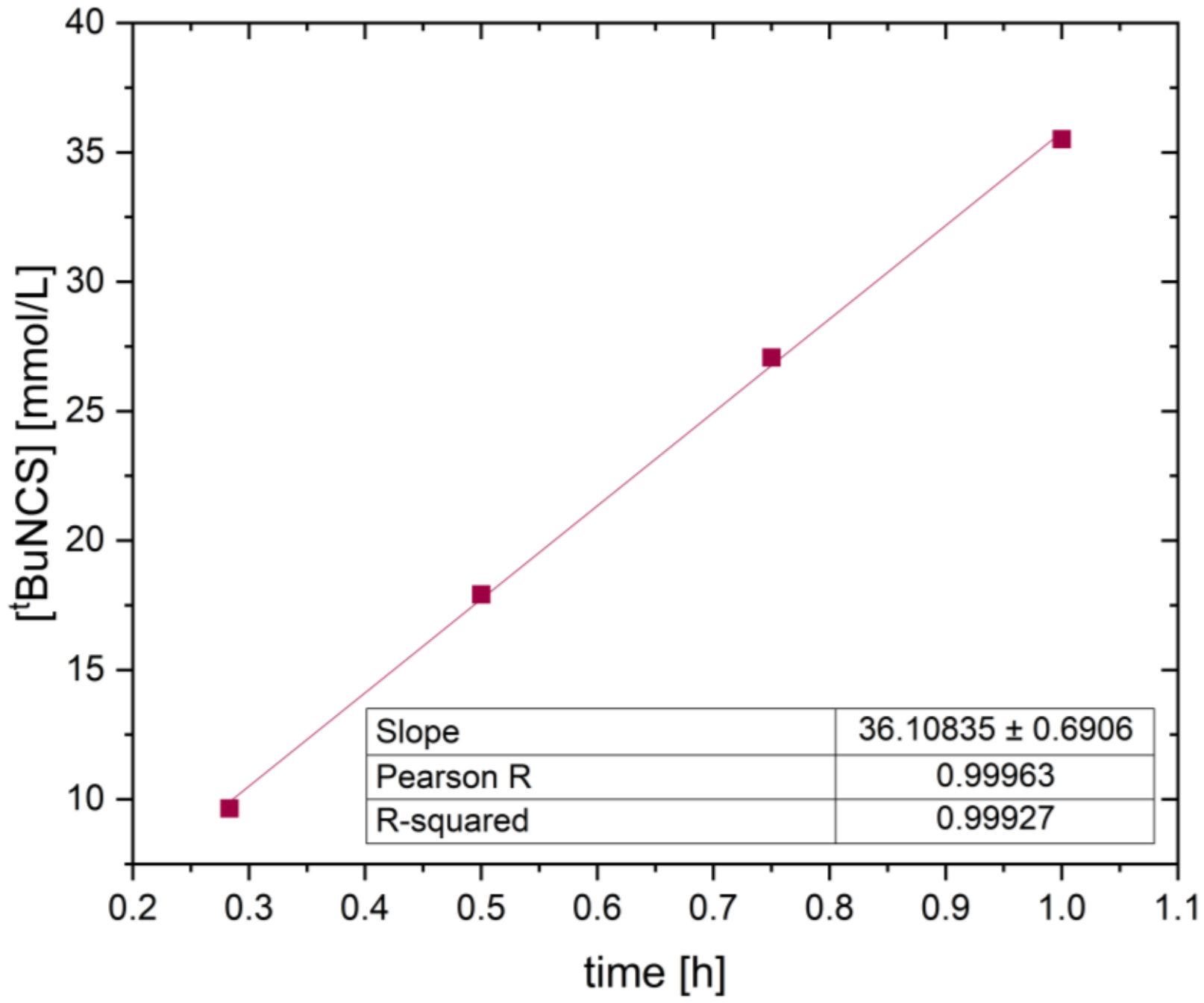

Figure S 76. Initial rate plot with [S_8] = 57.18 mmol/L.

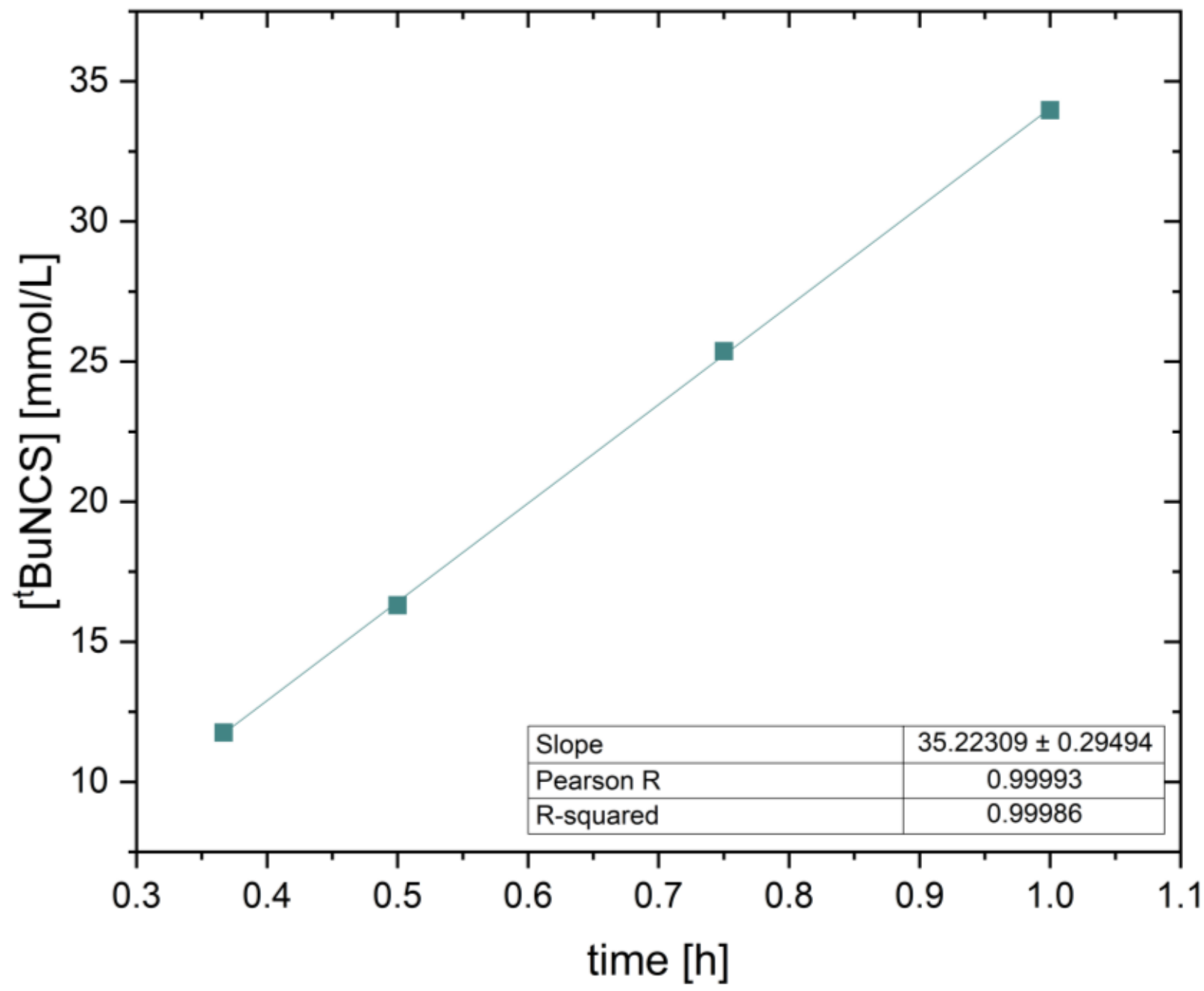

Figure S 77. Initial rate plot with [S_8] = 76.26 mmol/L.

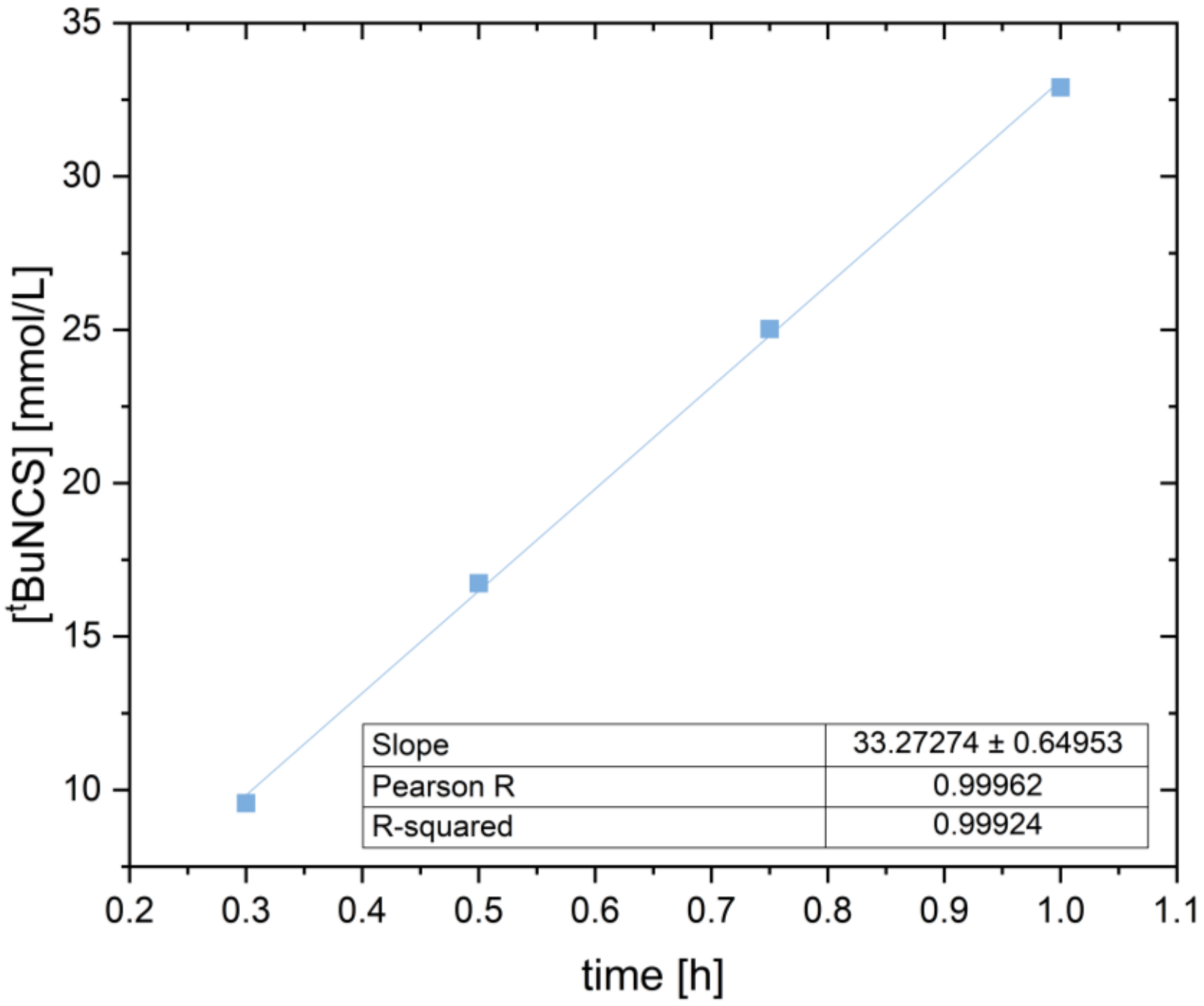

Figure S 78. Initial rate plot with [S_8] = 95.32 mmol/L.

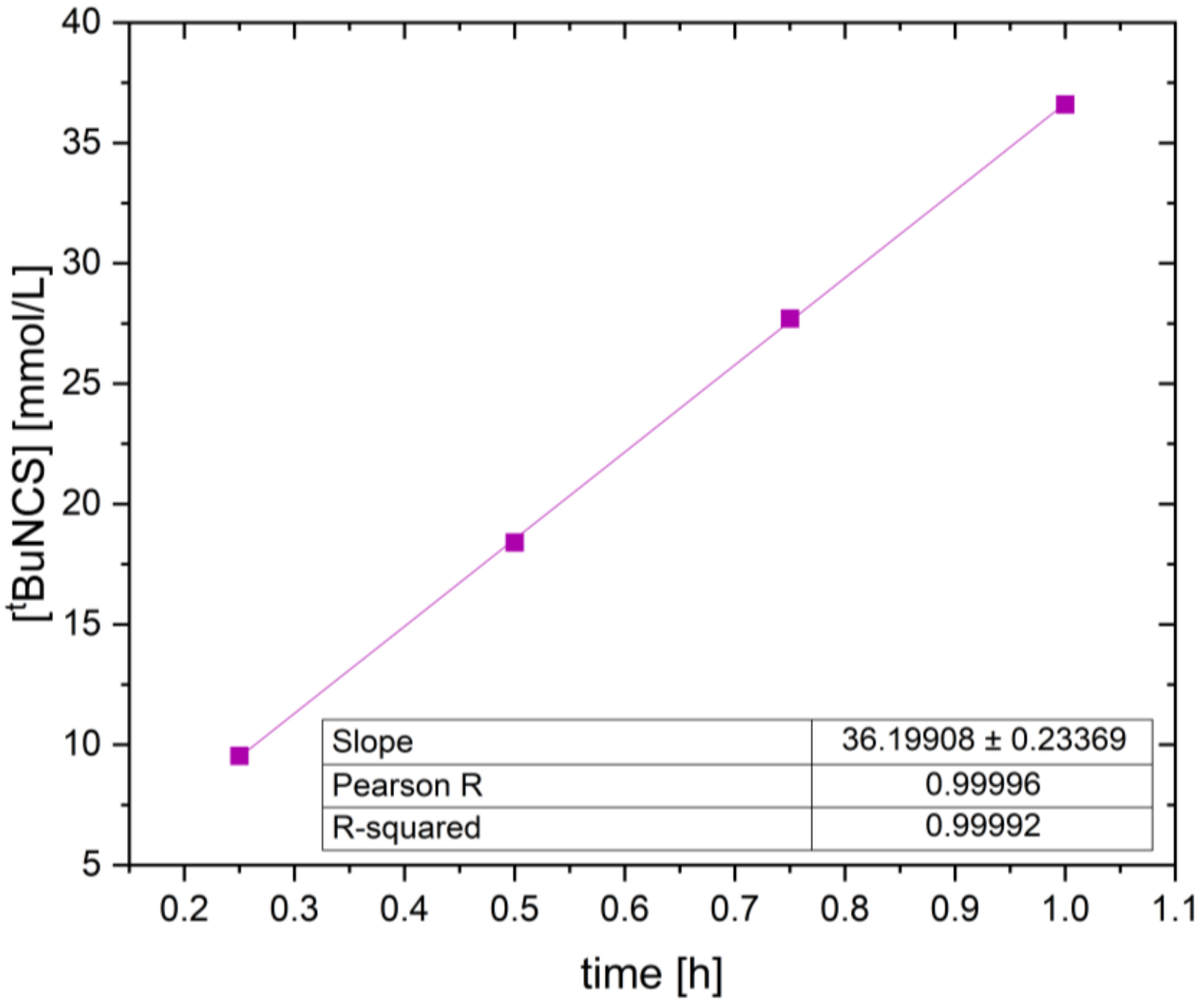

Figure S 79. Initial rate plot with [S_8] = 114.38 mmol/L.

The order in [S_8] was determined by plotting the resulting values for k_{obs} against the respective concentration [S_8], confirming a zeroth order in [S_8] due to constant value observed for k_{obs} with varying [^{t}BuNC].

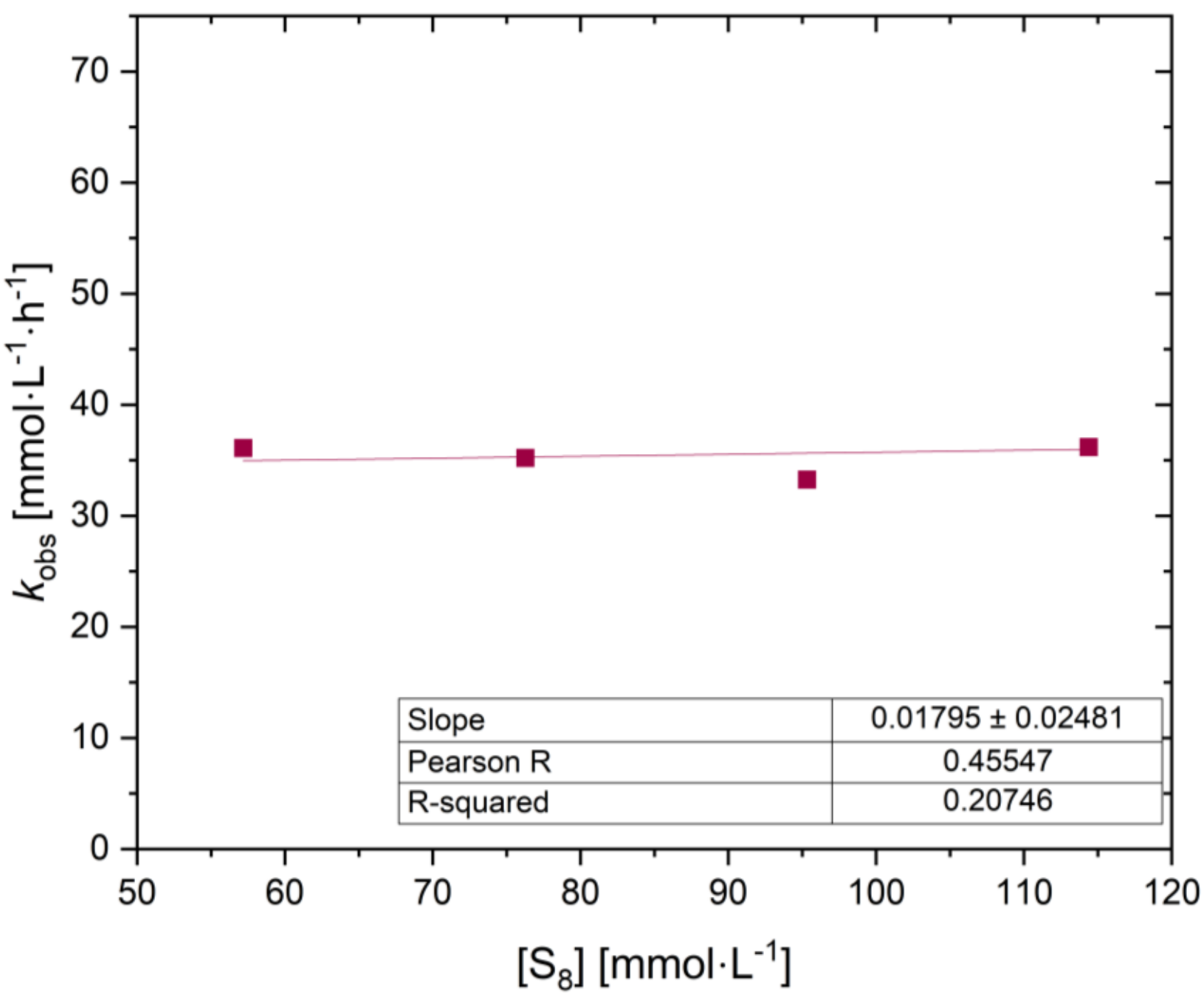

Figure S 80. Absence of correlation between k_{obs} and [S_8].

10 X-ray Crystallography

The crystal data of all compounds was collected on a Rigaku XtaLAB Synergy-R diffractometer with a HPA area detector and multi-layer mirror monochromated Cu-K_{α} radiation. Multi-scan absorption correction was applied to the data.[11,12] The structures were solved with SHELXT,[13] refined with the SHELXL14 using full matrix least squares minimization on F^2 [15] using using Olex2 as the graphical interface.[16] All non-hydrogen atoms were refined anisotropically. The hydrogen atoms were located in idealized positions and refined isotropically with a riding model.

Crystallographic data has been deposited with the Cambridge Crystallographic Data Centre as supplementary publication. These data can be obtained free of charge from The Cambridge Crystallographic Data Centre *via* www.ccdc.cam.ac.uk/data_request/cif.

Table S2. Crystallographic data and structure refinement.

	**(^{Et}PDI)TiCl$_2$(S$_2$)**	**(^{Et}PDI)Ti(S$_2$)$_2$**
CCDC	2530492	2530493
Empirical formula	$C_{29}H_{35}Cl_2N_3S_2Ti$	$C_{29}H_{35}N_3S_4Ti$
Formula weight	651.60	697.88
Temperature [K]	99.96(18)	100.0(4)
Crystal system	monoclinic	monoclinic
Space group (number)	$P2(1)/c$	$I2/a$
a [Å]	10.1526(2)	18.0037(3)
b [Å]	26.4814(5)	11.1311(3)
c [Å]	12.5856(2)	37.9336(7)
α [°]	90	90
β [°]	106.323(2)	98.364(2)
γ [°]	90	90
Volume [Å^3]	3247.31(11)	7521.1(3)
Z	4	8
ρ_{calc} [gcm^{-3}]	1.333	1.233
μ [mm^{-1}]	5.142	4.211
F(000)	1372	2948
Crystal size [mm^3]	0.143x0.053x 0.032	0.206×0.065×0.033
Crystal colour	clear green	green
Crystal shape	block	block
Radiation	CuK_α (λ= 1.54184 Å)	CuK_α (λ= 1.54184 Å)
2θ range [°]	4.0110 to 73.7410	4.7080 to 73.0190
Index ranges	−12 ≤ h ≤ 12<br>−29 ≤ k ≤ 32<br>−15 ≤ l ≤ 12	−21 ≤ h ≤ 22<br>−12 ≤ k ≤ 13<br>−36 ≤ l ≤ 45
Reflections collected	17024	20981
Independent reflections	6361<br>R_{int} = 0.0262<br>R_{sigma} = 0.0334	7348<br>R_{int} = 0.0484<br>R_{sigma} = 0.0522
Data / Restraints / Parameters	6361/19/351	7348/158/444
Absorption correction T_{min}/T_{max} (method)	0.637/1.000<br>(gaussian)	0.576/1.000<br>(gaussian)
Goodness-of-fit on F^2	1.046	1.080
Final *R* indexes [$I \geq 2\sigma(I)$]	R_1 = 0.0403<br>wR_2 = 0.1089	R_1 = 0.0558<br>wR_2 = 0.1563
Final *R* indexes [all data]	R_1 = 0.0509<br>wR_2 = 0.1143	R_1 = 0.0723<br>wR_2 = 0.1644
Largest peak/hole [eÅ^{-3}]	0.75/-0.36	0.66/−0.72

11 Quantum Chemical Calculations

DFT calculations were performed with the ORCA 6.1 program package.[17,18,19] Geometry optimizations and single-point calculations were carried out using the PBE0 functional in combination with Grimme's dispersion correction D4.[20,21] Alrichs' all-electron Gaussian triple zeta basis set def2-TZVP was employed on all atoms.[22] The RIJCOSX approximation was used to accelerate the calculations.[23,24,25] Final Gibbs free energies were then obtained by using the single-point electronic energies and the thermal corrections from the frequency calculations. For mechanistic considerations, stationary points were identified as intermediates or transition states by frequency calculations and the presence or absence of an imaginary frequency. Single-point calculations were conducted using the optimized geometries (PBE0-D4/def2-TZVP) with a larger TZVPP basis and a CPCM solvation model for benzene. Final Gibbs free energies were then obtained by using the single-point electronic energies and the thermal corrections from the frequency calculations. For potential energy surface scans PBE0-D4/def2-SVP was used. Chemcraft was used to visualize optimized geometries.[26]

Table S3. Solvent-corrected electronic energies (E_{el}; benzene) for PBE0-D4/def2-TZVPP, non-thermal ZPE corrections (ZPE), thermal enthalpy correction (H_{therm}), and entropy correction (-TS) for mechanistic calculations (PBE0-D4/def2-TZVP); all energies are given in Hartree; relative Gibbs energy is given with reference to $[(^{Et}PDI)Ti(S_2)_2]$ + ^tBuNC and using 1 molar solutions.

Compound	E_{el}	E_{ZPE}	H_{therm}	-TS	Final Gibbs	Relative Gibbs Energy [kcal·mol^{-1}]
$[(^{Et}PDI)Ti(S_2)_2]$	-3731.266745	0.586059	0.000944	0.104839	-3730.784581	0
$[(^{Et}PDI)Ti(S_2)_2(^tBuNC)]$	-3981.741013	0.720987	0.000944	0.115211	-3981.134294	13.422
$[(^{Et}PDI)Ti(S_2)(S)]$	-3333.169155	0.582130	0.000944	0.096572	-3332.682654	-6.599
^tBuNC	-250.466208	0.129705	0.000944	0.038555	-250.374114	/
^tBuNCS	-648.580751	0.132341	0.000944	0.039092	-648.486558	/
TS	-3981.718626	0.716749	0.000944	0.120807	-3981.121741	21.299

For determination of dispersion effects in the transition state, single point calculations were carried out for the ^tBuNC- and the $(^{Et}PDI)Ti(S_2)_2$ fragments were carried out and the dispersion correction was compared to that in the transition state as reported by Power and Nagase.[27]

Table S4. D4 dispersion correction in the transition state (PBE0-D4/def2-TZVPP).

D4 dispersion corr.	TS	Ti_fragment	nitrile_fragment	nitrile_Dep_interaction
Hartree	-0.147026	-0.125724	-0.010193	-0.011109
[kcal·mol^{-1}]	-92.259335	-78.891891	-6.396387	-6.971057

The highest occupied molecular orbital interactions can be rationalized with symmetry-adapted linear combinations (SALCs) of the overall four $(S_2)^{2-}$ π* orbitals, giving rise to σ, σ*, π and π* SALCs and their (partial) overlap with titanium-based orbitals. The corresponding Kohn-Sham molecular orbitals HOMO-3, HOMO-2, HOMO-1 and the HOMO show that HOMO-3 and HOMO-2 correspond to predominantly σ-type Ti−S bonding interactions, whereas HOMO-1 and HOMO exhibit minor π-type interactions and predominantly sulfur character. The other Kohn-Sham orbitals with high Ti-S σ-bonding character are depicted in Figure S 82.

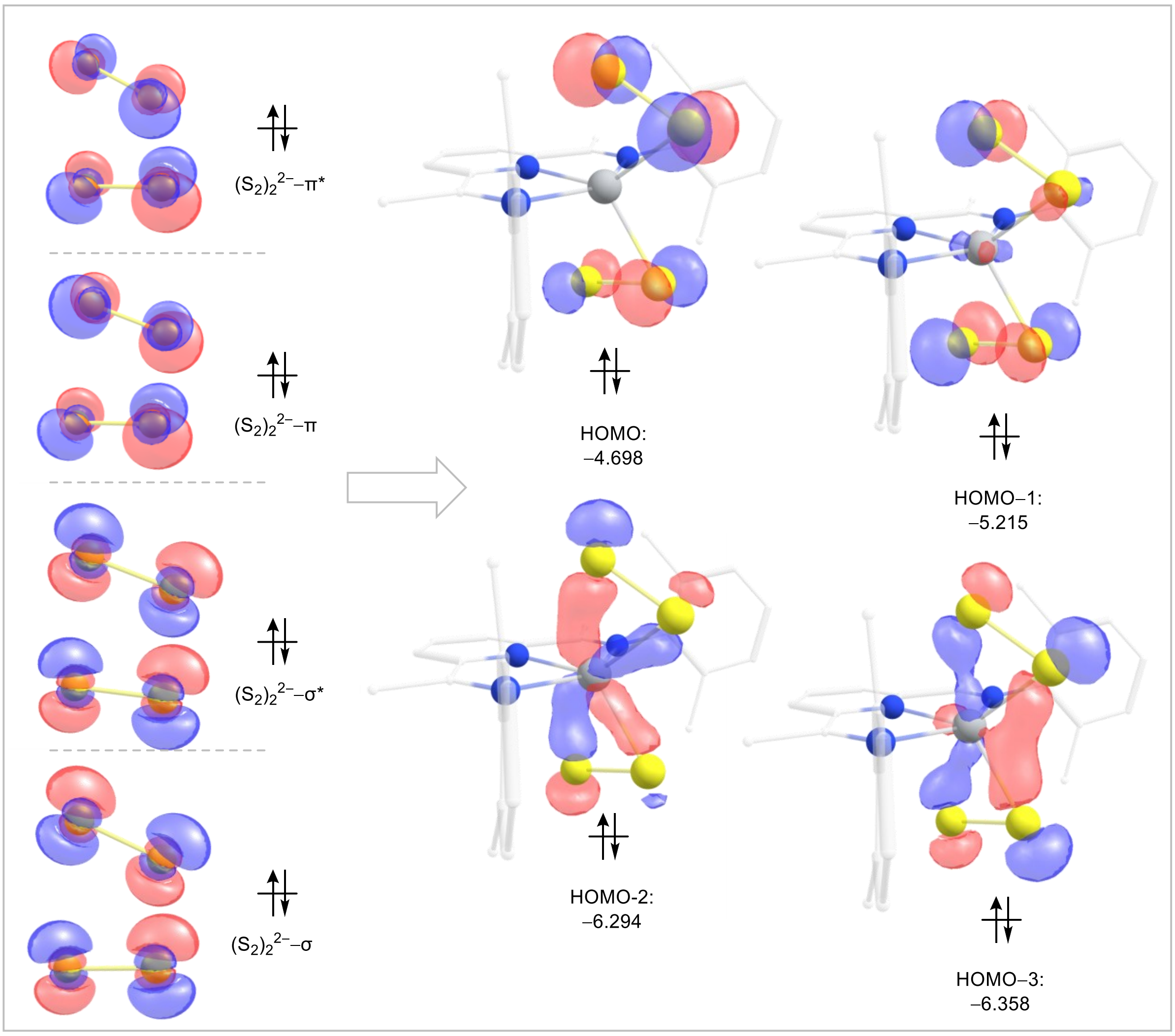

Figure S 81. Analysis of TiS_4 bonding in $(^{Me}PDI)Ti(S_2)_2$ by DFT with Kohn-Sham MOs; isosurface value 0.06.

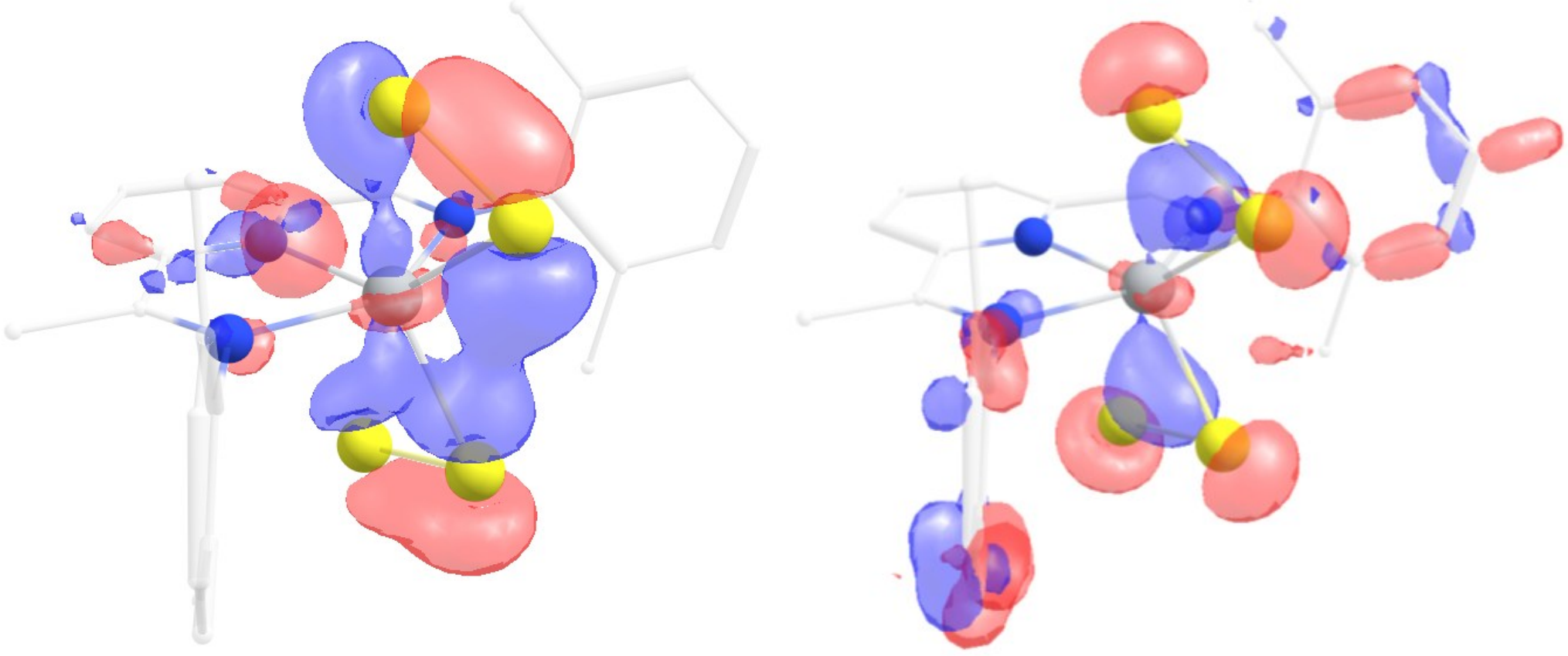

Figure S 82. Kohn-Sham MOs HOMO-8 (left) and HOMO-11 (right) featuring Ti−S σ-bonding character; isosurface value 0.05.

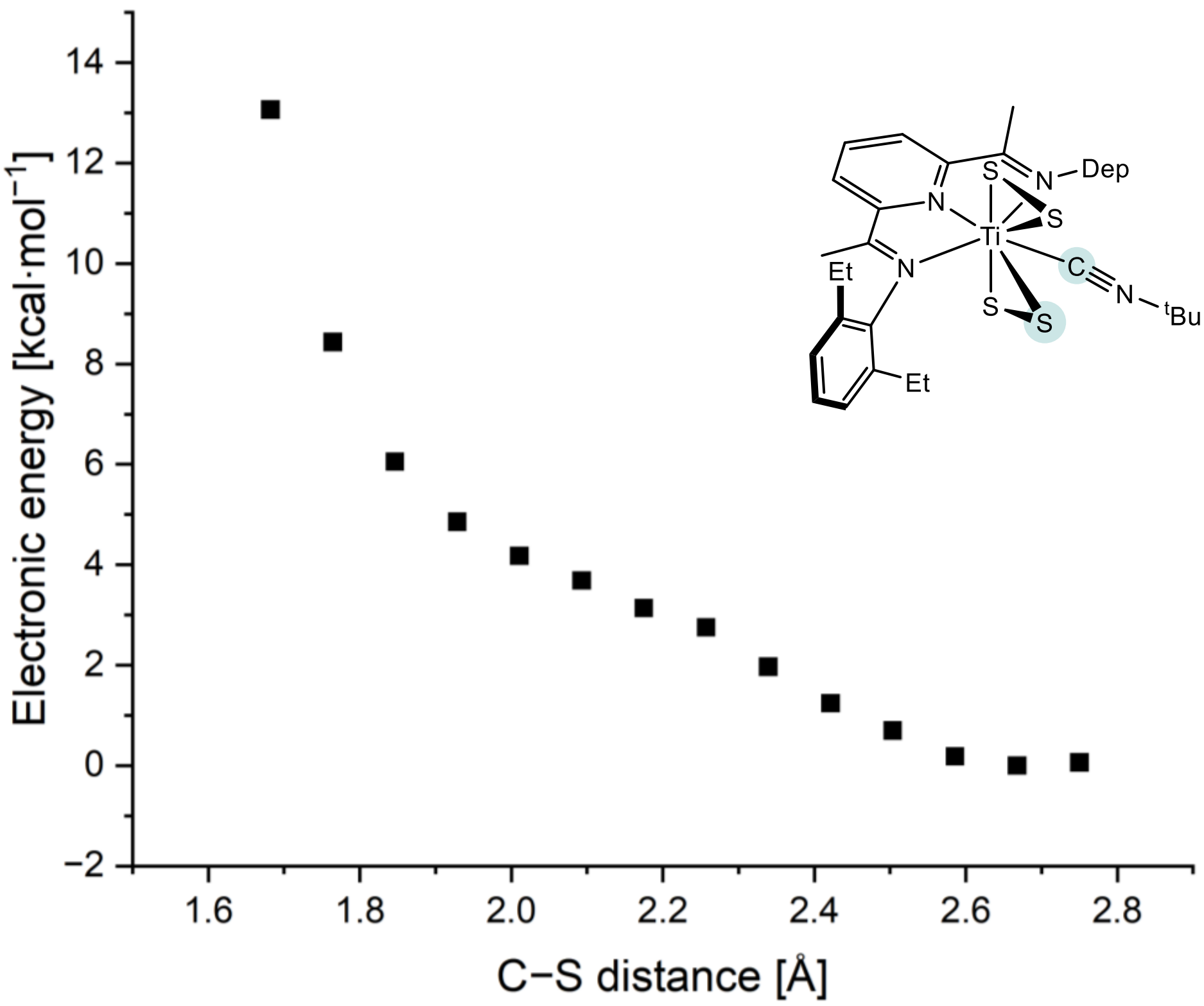

Figure S 83. Potential energy surface scan of [(^{Et}PDI)Ti(S_2)$_2$(^{t}BuNC)] along the C(isonitrile)-S bond (atoms marked in blue).

Cartesian Coordinates

$(^{Me}PDI)Ti(S_2)_2$

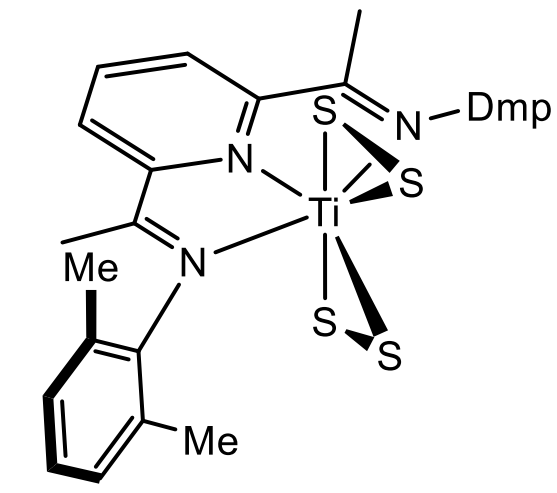

Ti	6.47795615162040	5.37345439285207	8.96088676678533
S	6.56530373284133	3.80918313310345	7.23312588100876
S	5.05495077364689	3.45839751408907	8.65341287827859
S	8.20341143236507	6.52186261087048	7.87547887199630
S	7.71149511868631	7.23899046757220	9.80428336701676
N	7.24948949974231	4.11637968725611	10.62197589519405
C	6.73951372334492	4.32627175526139	11.81706297929538
N	5.28838799863462	5.80234420845805	10.65426310776681
C	7.19381213257680	3.65076509384245	13.07395721649651
H	8.08450446843244	3.02434342238529	12.88985480861838
H	6.39716950156198	3.01207755342644	13.50826969759125
H	7.45190479963671	4.41439861576155	13.83705712009083
N	4.86118515789390	6.72072292800991	8.26298725913387
C	5.63704048235377	5.29235420939442	11.86298278338866
C	4.92661616605755	5.65383350396729	13.02653511791193
H	5.21715492929474	5.23921511373982	14.00070600203351
C	3.84780434565439	6.53957398546607	12.91796616580685
H	3.27862310130167	6.83165051062359	13.81220368166167
C	3.49772797182012	7.05211932135794	11.65898531476420
H	2.65489210267488	7.74692745424915	11.54974970006050
C	4.25184404692534	6.66588374576930	10.53614426033696
C	4.02104389769925	7.15614549116629	9.16264797268610
C	2.89743234638733	8.10407087789401	8.88552506512895
H	2.84184597935882	8.35309498576615	7.81131215898279
H	3.03587074269230	9.04272926859416	9.46110274139436
H	1.93023546013587	7.66503459100287	9.20506395683490
C	8.12063767980253	3.00820388939595	10.39736118258880
C	9.47658391888444	3.24295134471478	10.03913090233503
C	10.27825984487607	2.13381741537737	9.70956689136351
H	11.33058269691930	2.30889138524473	9.43622008752160
C	9.76328495563115	0.83150122358792	9.72634530461510
H	10.40171516977020	-0.02005417502099	9.44529551456691
C	8.43923413765670	0.61616298913670	10.12642429450623
H	8.03678192606694	-0.40810713413648	10.17059760574839
C	7.59878799896933	1.68541254776207	10.49414131994939
C	10.06526896424074	4.62173780011261	10.03580026680771
H	11.16735917393060	4.58056995779043	10.13529686635467
H	9.65170874293756	5.26139391743973	10.83946833427354
H	9.82244212916418	5.14854172811905	9.08344931596619
C	6.21931101974241	1.36962850062342	11.01458565987095
H	5.53321538346641	2.23364658774380	10.96232335821103
H	6.27148037806128	1.02062601024535	12.06977598745716
H	5.75803207603106	0.55221284583415	10.42646300681246
C	4.76159455976765	7.15216354407587	6.89318629120015
C	3.98908396421747	6.38661196748052	5.98125261212808
C	4.01028207499690	6.75926205180166	4.62331356674585
H	3.42677090294702	6.16200495657592	3.90534132847431
C	4.74680071568338	7.86731897261812	4.18373805656857
H	4.76509558174841	8.13122883596173	3.11520290744203
C	5.43607264656659	8.65213131822364	5.11550707431486
H	5.98190356256268	9.55033755647446	4.78629936840600
C	5.44724122426887	8.32434234083658	6.48652681054765
C	3.10467315705498	5.26382423702397	6.44718679774501
H	2.74865907056529	4.65719182678764	5.59239113367792
H	2.20575670716149	5.66308147004077	6.96804025795221
H	3.61798043332297	4.59433745572221	7.16830252812716
C	6.09479760559705	9.25860861237860	7.47310237655118

H	6.97737543045860	9.75759181946464	7.02760507377977
H	6.42375705537002	8.74916839178350	8.40000383253100
H	5.38065304821917	10.06173336689917	7.76381331459378

$(^{Et}PDI)Ti(S_2)_2$

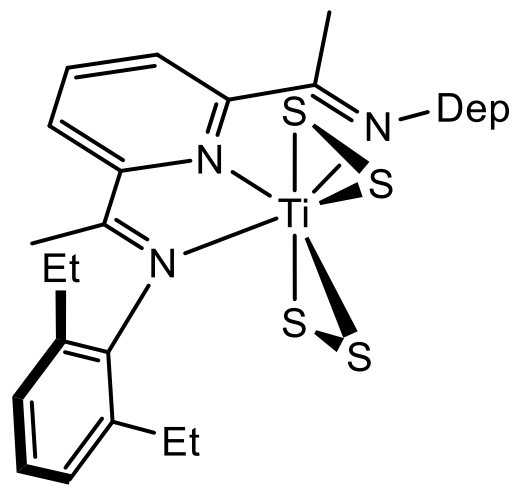

Ti	6.09347260979960	5.82670647382662	23.80131823177812
S	6.14196747829139	4.03040161085166	25.37758306959254
S	4.46863315669503	5.22995654900340	25.31975797230077
S	6.56148922655052	7.40020813298138	22.06743804549424
S	4.84827818823381	7.66095451110985	23.18042661456311
N	7.95347674238741	5.02560571660733	23.22437029060837
N	7.66785220160503	6.91797245355810	25.00474797986042
N	5.66014699597946	4.23991474442643	22.24486908283936
C	9.07022205479595	5.47423629820152	23.80212579292404
C	8.87923543392859	6.54960590508455	24.79009918844869
C	7.99925359789021	4.07704106785044	22.28606598040367
C	7.41010242462175	8.05161243686358	25.84526213732769
C	6.69176387952771	3.64397061400879	21.76504635099111
C	10.37812548729410	3.98398625079284	22.47193514578356
H	11.33494359834446	3.57280862229029	22.17482722414396
C	10.31180019620551	4.96586252174904	23.44452134243145
H	11.21122984812278	5.33520000740633	23.91744320779087
C	4.34889323098380	3.77709968522361	21.89252308726872
C	6.57658459204027	8.95865699804440	27.90357511129403
H	6.17192352540027	8.79889714918497	28.89773763140988
C	10.08178719367612	7.14344496695059	25.43154479470954
H	10.70049459744901	7.64644861979786	24.68287251348751
H	10.69238760676727	6.35887673867327	25.88481998302838
H	9.80714866912730	7.86388584019013	26.19752538273438
C	9.21079219175006	3.53182891568431	21.88248033598621
H	9.23921691879909	2.76351418120798	21.12237828547843
C	6.89999783953281	7.84718669532283	27.13485270058979
C	6.65606092591304	2.54152040376545	20.76815205908370
H	7.00089709504178	1.60952761469211	21.22539886058378
H	7.32541792135005	2.76306805841876	19.93373119469312
H	5.65137596106400	2.38752647018858	20.38317592638581
C	6.77100443464205	10.24242977261932	27.42982456052489
H	6.50640896311162	11.09885798562911	28.03980337457545
C	7.66386512380081	9.34612081810881	25.36357257713223
C	7.32662112040224	10.42347058244919	26.17834358983695
H	7.51182866221323	11.42813639437117	25.81582765082669
C	3.86066835579642	2.58236781350647	22.44658681628120
C	6.79118746718862	6.49067819683614	27.75997523474448
H	6.59698963845714	5.72860226173394	27.00642899613793
H	5.93755734084206	6.48887754218004	28.44294830727465
C	3.57272228724763	4.53102261673712	21.00116600085325
C	2.55742274492821	2.20361590115795	22.13650936318735
H	2.16613978678067	1.28573094817785	22.56033289655971
C	4.68002476823631	1.67065727024228	23.31960116659202
H	5.63092759744487	2.13994613786541	23.56662510383574
H	4.90420713688898	0.76138606516076	22.74711264714356
C	8.31867968374783	9.63323391539027	24.03949158638819
H	8.43255582820026	8.71462952930859	23.46585733450393
H	9.32672519128428	10.02060717398364	24.23573410964961
C	8.05155289753399	6.11193054237760	28.53047575470542
H	8.92132895036829	6.05953229360059	27.87010328350556
H	7.93319163581114	5.13117473427531	28.99868168725256
H	8.27616269878907	6.83791952242326	29.31691670822118
C	1.75908039600182	2.96254271995715	21.30309421201296
H	0.74315944255349	2.65138002339389	21.08720268785129

C	2.27619991369741	4.11019944442728	20.73201856809247
H	1.67270068049372	4.70003817991554	20.04970125336088
C	4.11625534654705	5.70406658673791	20.24505063748335
H	4.87342763902225	6.22505923265436	20.82940051392114
H	3.30367872179338	6.41404850302595	20.06951726264248
C	7.55988547851740	10.63265452338250	23.17819009952819
H	8.05690656847832	10.75245682772807	22.21227859740270
H	7.50189051137070	11.61922472819715	23.64353357508426
H	6.54325449507653	10.28319340198891	22.98991366008485
C	3.99790934297548	1.28401265929046	24.62392604391439
H	3.08466276076258	0.70784332119100	24.45896686334452
H	3.74157821237532	2.17366212843560	25.20177209425254
H	4.66806362350289	0.67153505957229	25.23240776610494
C	4.72414097496206	5.29411493222526	18.90803659111795
H	5.08929057106436	6.17240275022347	18.36917543571921
H	3.99444327502671	4.78558267103724	18.27161121458088
H	5.57092834289393	4.61692803452345	19.04757864975047

(^{Et}PDI)Ti(S_2)$_2$(^{t}BuNC)

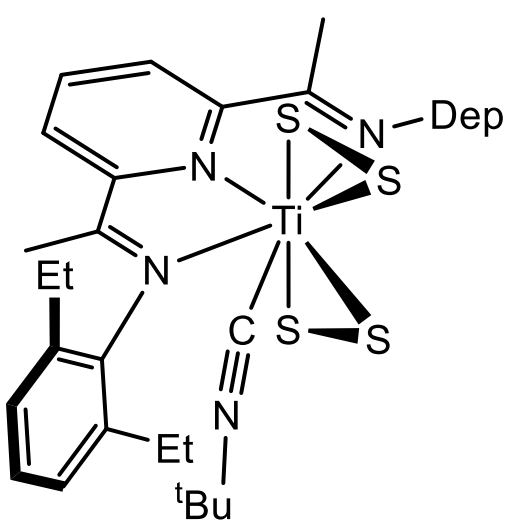

Ti	6.25665184938889	4.26295621586722	7.76935666354727
S	4.68618763728857	3.18638793039083	6.37750302826826
S	4.31119776477286	3.05457711596606	8.39383192531800
S	7.29104298087171	5.11721398908752	5.81605982188166
S	8.17050183194665	5.66421544185622	7.59018304116132
N	7.38387926997207	3.06983851099164	9.68650456406102
C	7.19484060265000	3.56552642378521	10.84543972700506
N	5.91779578618424	5.28782360161337	9.76557166130659
C	7.70898132336533	2.98455151638206	12.12050663791544
H	8.30046509801500	2.09194044237805	11.93571714775774
H	6.87637655546836	2.71768300254662	12.77745702343229
H	8.32807338410731	3.70916095980641	12.65469152511184
N	4.85612873486350	6.21132474612699	7.49955251697563
C	6.40301871775716	4.80425427848173	10.91485406441677
C	6.18530894713507	5.42965741296754	12.13818609181046
H	6.59239998574379	5.00345913277391	13.04329596773301
C	5.45087837581084	6.59455387145390	12.18233531726451
H	5.27009301718417	7.10366035242435	13.12097525953934
C	4.94962983560827	7.09352127424033	10.99782636523573
H	4.36661698456160	8.00259628790595	10.99086502815545
C	5.19634209050816	6.41494865358913	9.81099129378993
C	4.64313222115802	6.91518815655284	8.54067720623276
C	3.86928373018623	8.19002228384300	8.57040154662043
H	3.51151088274754	8.45574152531766	7.57980767219680
H	4.49297965530502	9.00278773740532	8.95123862631723
H	3.00991720224419	8.09704324248004	9.23879272362080
C	8.17825082367763	1.88725428966157	9.58567038294715
C	9.56787091959718	2.03346292584973	9.43290906506375
C	10.33975762100153	0.88872572119968	9.29715059182377
H	11.41104591332603	0.98347478268394	9.17013931474032
C	9.76625007960105	-0.37318846765312	9.33494571791651
H	10.38662680967153	-1.25687667826639	9.23432637738591
C	8.40453632210804	-0.49467083208116	9.51880712998543
H	7.95108539773783	-1.47902381209623	9.57326608750248
C	7.58392808725998	0.62508198570579	9.64725792434107
C	10.18577800622901	3.40122534168787	9.45305470714812
C	11.64303468286278	3.47988971190894	9.04490015802100
H	10.07364660177257	3.82249185733498	10.46110857405199
H	9.59504776292527	4.05292258989802	8.80193340313949
C	6.11320760876260	0.41023666914944	9.86504223940686
H	5.98702505092158	-0.22128847631324	10.75222066103975

C	5.42873104795366	-0.25176863858048	8.67666736169099
H	5.61579595352878	1.35777499670735	10.06833321863292
C	4.33685618125567	6.66573726926418	6.24873208476868
C	3.11380222517775	6.16871397203518	5.79021802705306
C	2.68412221900217	6.55045870543131	4.52160302922705
H	1.74509846312944	6.15279544156247	4.14955094601875
C	3.42662545949243	7.41604357084973	3.74283903040553
H	3.08578436174101	7.68814975588990	2.74991966693290
C	4.59686699133823	7.95778857906163	4.24901225115809
H	5.15740034768453	8.66738131799910	3.65283078934160
C	5.06720796074345	7.61007413513707	5.50935420485300
C	2.20385837470132	5.32660996699960	6.63689144493834
H	1.81721188226416	4.50626854703069	6.02644357977898
C	1.03035485691052	6.12436056366662	7.19655464067281
H	2.75852779113157	4.86412139214150	7.45350944707495
C	6.26775752681695	8.29600090705863	6.10031060526301
C	7.29175408712274	8.80679400275120	5.10359284184220
H	6.76397567102238	7.61895914583240	6.79702640095544
H	5.90940066049382	9.14198114429196	6.70409805685732
C	7.40007182552533	2.59245334943326	6.91361538024629
N	7.94894129837824	1.76487585100629	6.31585702991995
C	8.52219428374296	0.91811990280425	5.31139921460681
C	8.08835507976876	-0.51524344721487	5.58876265148792
C	10.03734693235063	1.05811333568639	5.39212194799998
C	7.99877524887838	1.39799879998150	3.96164991438805
H	8.43337625670248	-0.84261300452508	6.57071796725241
H	8.51637143116953	-1.17444337178034	4.83080969846497
H	7.00120309757972	-0.60473287492362	5.55110122947648
H	6.91023395428356	1.32654423476508	3.92255058625227
H	8.41929054248849	0.77723306560522	3.16770198275384
H	8.28381122870700	2.43643639899507	3.78450205874220
H	10.40516081689183	0.73356500150537	6.36693451373214
H	10.33654829427017	2.09610123874868	5.23345974874108
H	10.50023800827461	0.44006593452753	4.62023055994969
H	0.42845311283751	6.56816787643591	6.39878890169710
H	1.36783651163640	6.93808513025754	7.84352058047531
H	0.37619530936199	5.47849121379717	7.78890406714254
H	6.90603221469041	9.61492902793149	4.47704299737460
H	7.63423951880342	8.00124765236446	4.44973877359034
H	8.16356457053344	9.19759432649927	5.63422186858611
H	5.49993632760204	0.37857877317471	7.78874376782743
H	4.36760833753497	-0.40842165515390	8.88473660192033
H	5.87282240936760	-1.22479968234277	8.44992173663323
H	11.79938465464845	3.08365744248327	8.03769899766943
H	12.30213782453951	2.93610453811154	9.72708080947145
H	11.96852269561958	4.52271445179081	9.03924400493943

(^{Et}PDI)Ti(S_2)(S)

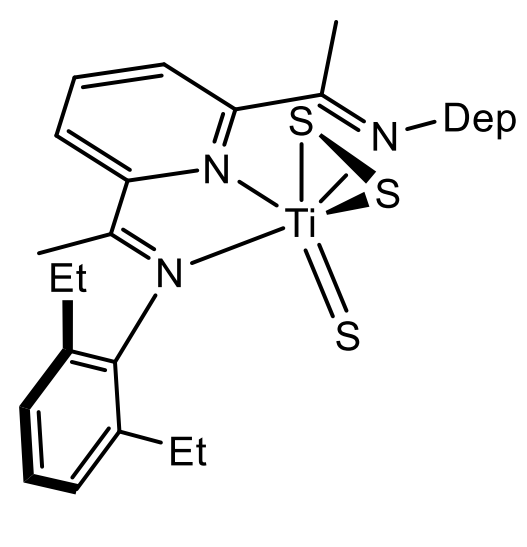

Ti	6.19657489779232	6.07018037647134	23.47388244480033
S	4.85202268546617	5.50863197366027	25.00237864599197
S	7.07762994916671	7.34681138150778	21.50882459929061
S	5.27023527703682	7.85632021955400	22.45730443192945
N	8.04284689976780	5.02977941669677	23.15520648168182
N	7.71293793279346	6.97453078036395	24.79664504073184
N	5.76951997575641	4.32112312074072	22.19419036768924
C	9.16359371743050	5.50069434343545	23.71472703719825
C	8.94155210334321	6.60672206809489	24.66475574388845
C	8.07767099028086	3.99529486304663	22.30785809580237
C	7.34759674741113	7.96230551124760	25.76345914415390
C	6.75326543391443	3.57932985564809	21.81282758422115

C	10.46354317355818	3.88141614360566	22.51613859053885
H	11.42382404167042	3.43089902768793	22.25754932482824
C	10.40766833261907	4.94750332749546	23.41022995836393
H	11.31643025850240	5.34213610282508	23.86515749192266
C	4.42262953682445	3.95002234666306	21.89750854974029
C	6.55125986557440	8.50809866810705	27.96382016911698
H	6.24891611399770	8.19148599452303	28.96617406672545
C	10.10140336883521	7.20294332082037	25.38084618116090
H	10.76579052704853	7.69261868308102	24.65012958321276
H	10.69323897462091	6.42804829581879	25.89000508750918
H	9.77908235932658	7.95001832559889	26.11491554099331
C	9.28630240762774	3.38850305776919	21.96139990776741
H	9.30522021619842	2.54754325816191	21.26788041015397
C	6.98346070591401	7.53732712058626	27.05458625951379
C	6.64132027338937	2.36966679810449	20.95310937991961
H	7.05353639890264	1.48863182378995	21.46913515748514
H	7.22941934583895	2.51139528332564	20.03269050263511
H	5.60020722285002	2.16825996274410	20.67674740987952
C	6.49325429035298	9.85060659295725	27.61131207679822
H	6.13909220924720	10.59265517814548	28.33131436621011
C	7.33863825053866	9.32135374260907	25.39424450308127
C	6.89481477243578	10.24955346077894	26.34029327476751
H	6.86483480886899	11.30787644362323	26.07386466396157
C	3.80802065499900	2.96731217762719	22.70523818398606
C	7.09375105420686	6.10465541832379	27.50758355286948
H	7.09601545104731	5.43502538675802	26.63731527314710
H	6.18169158264250	5.84948762733605	28.07031077531891
C	3.74088842372968	4.58611541043472	20.84922948837393
C	2.46430199557352	2.67733375230211	22.46334765352883
H	1.95620693378867	1.93651870694233	23.08315906776104
C	4.59629911792186	2.25314228959972	23.77418990875739
H	5.25151196169614	2.99392599379614	24.25982666501274
H	5.26660956189733	1.52065979472464	23.28699187048495
C	7.84559197564610	9.75826336500004	24.04463154167483
H	7.72537668563601	8.93551544556190	23.32295400753348
H	8.93850095597665	9.91767211316566	24.12613930442975
C	8.31255727469296	5.84689932872100	28.38830506427530
H	9.25416786554959	6.05606501087530	27.85758368687490
H	8.34192382867241	4.79566949454109	28.71650758106077
H	8.30117398350853	6.47716298043037	29.29190160358389
C	1.75710172298657	3.31978381778529	21.44930202667454
H	0.70273285748769	3.08329701324909	21.28557438295911
C	2.39617856496877	4.25395087643300	20.64605015671508
H	1.84959918411486	4.74912341484906	19.83837407000411
C	4.42384822646039	5.53553140194208	19.90426888466990
H	5.32072873882857	5.97180247177777	20.36897821737788
H	3.74437269816731	6.37803548266585	19.69855795590300
C	7.20245344798690	11.00947813558953	23.47109797489347
H	7.56817652670282	11.18444938758988	22.44766153328839
H	7.42736419702103	11.91568344219029	24.05547378152935
H	6.10823552715063	10.90034527748185	23.41660775325551
C	3.78013966842403	1.54494642371053	24.84028125975600
H	3.17628903709728	0.71741376896861	24.43596907219082
H	3.10056259475801	2.24471502183669	25.35128572825307
H	4.44673453871810	1.11486938459312	25.60348850082228
C	4.81383802770161	4.86957817413286	18.58806670755997
H	5.31108580673049	5.59061457825472	17.92009507924532
H	3.93684368463277	4.46683193125866	18.05579308488359
H	5.51069157797391	4.03173883026112	18.75034652767759

Transition state (^{Et}PDI)Ti(S_2)(SS-CN^{t}Bu)

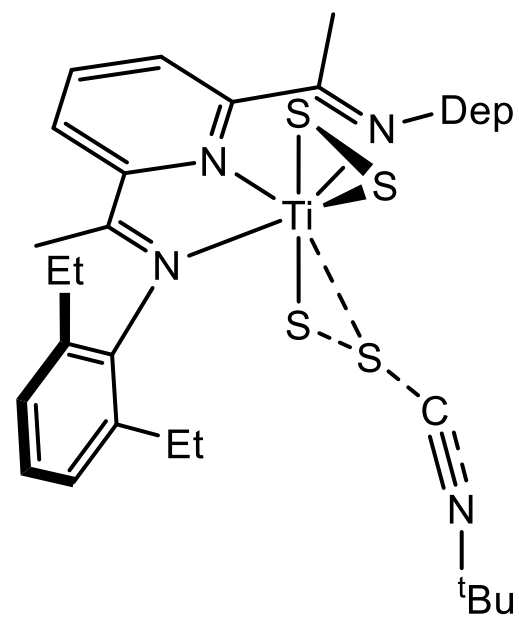

Imaginary frequency: -312.77 cm^{-1}

Ti	6.08246076462666	5.61722653149075	24.21048514658960
S	6.73843450419926	3.78780009072504	25.47122990941578
S	4.66439914664929	4.75434888662840	25.85920893050849
S	6.10627314834636	7.34791506623935	22.52715079441539
S	4.58931750084496	7.36795730788200	23.91000037606707
N	7.87739150502407	5.13651916519377	23.20902019554870
N	7.71320690884423	6.85790832957192	25.15820814811099
N	5.55327846191655	4.15892249108033	22.57545789073508
C	9.02536504109181	5.68313829874519	23.61941177870793
C	8.89796497113727	6.66275968065994	24.70487913794202
C	7.85878815125456	4.26077724201390	22.20082459204482
C	7.48704562539921	7.89769880107676	26.11506794891238
C	6.53534355142702	3.71716797197128	21.87654024430236
C	10.23089302043818	4.43362457536503	21.97716739459296
H	11.15890903000005	4.15463173030459	21.49408599044148
C	10.23023499602560	5.34572860271695	23.01774350298938
H	11.15356483215190	5.79402971773543	23.35745280368748
C	4.25263557104064	3.58731389891946	22.39447422149178
C	6.93988964034351	8.58035632092784	28.34511760113632
H	6.72034046250257	8.31630418866491	29.37415329528389
C	10.12210316822922	7.36641976491358	25.18017854462759
H	10.51080932088521	8.02557323387294	24.39874546076116
H	10.90252364238470	6.64178272392408	25.42355534094385
H	9.90964141014509	7.96531856793964	26.06226428110459
C	9.03111430667670	3.88535705868415	21.55901810951151
H	9.00482628419988	3.17150241579027	20.74744235523794
C	7.20870611035708	7.55631751722555	27.44635136914316
C	6.42925128423560	2.67340384728587	20.81876465740370
H	6.92210295224864	1.75478643091655	21.15052041145873
H	6.92389077328037	3.00397997847621	19.90282134163020
H	5.38996333919290	2.44558373863914	20.59602984477318
C	6.95807905551183	9.90652005497494	27.95695911985948
H	6.74157782685484	10.69107530863458	28.67295434618825
C	7.54250864997746	9.23879868305344	25.70498629950786
C	7.27007538636735	10.22263266940946	26.65042816519271
H	7.30668795796087	11.26180139787947	26.34403927802162
C	3.96926648765109	2.32824735167494	22.94287998352142
C	7.28250576870977	6.15270222436601	27.96177550839048
H	6.87734757984953	5.44726987429056	27.23669581855991
H	6.65821219968937	6.08339743446593	28.85615364116320
C	3.29158673163489	4.29415251672514	21.65886562083493
C	2.68384952114772	1.81973277261679	22.77896667639042
H	2.45274047465801	0.84960706552588	23.20319148626609
C	4.99191547855621	1.48215660763567	23.64925890719795
H	5.91551303846187	2.04348003669381	23.79074404477556
H	5.22129976453236	0.62119161549158	23.00706980707417
C	7.90319551690734	9.67762993599780	24.31177562239128
H	8.01763706808093	8.81388809433342	23.65847871757122
H	8.87271672326526	10.19138619612365	24.35324734277145
C	8.70557445457647	5.72822636414916	28.30646454164066
H	9.33957609105661	5.70841871099515	27.41684466216010
H	8.70854667354155	4.71940346783898	28.72446695355093
H	9.16356724215522	6.40531115008971	29.03288327874454
C	1.71346831812901	2.51078965003854	22.08144682940126
H	0.72119496427708	2.09040550928488	21.96224948113145
C	2.02747060147319	3.73452875197857	21.51938314534853
H	1.28513768391907	4.27571461697817	20.94137912515200

C	3.59743235918912	5.56900252010231	20.93503748299304
H	4.31389543154791	6.17083092358054	21.49302599065446
H	2.67800640464772	6.15679722835395	20.86118145398363
C	6.87002848029027	10.59963172152814	23.67775337725915
H	7.16348666272699	10.84975043454908	22.65590193060455
H	6.75761607662672	11.53410432440151	24.23161850103283
H	5.89914176454819	10.10423580968724	23.63744577983831
C	4.54532292274958	0.98660281315346	25.01788848943907
H	3.66081338920878	0.34793644823101	24.96019478521220
H	4.32448862248716	1.83387663022274	25.66829951673190
H	5.34546644937672	0.40850504679810	25.48444449461565
C	4.14688799063958	5.33086033814735	19.53248654208927
H	4.31032297327872	6.28279116545466	19.02261648754869
H	3.46325558143499	4.72919583981781	18.92744040931088
H	5.10723465568393	4.81150518247983	19.56599263517012
H	-1.25901057607424	7.14731124107069	25.47449381627466
H	-1.20217555297064	4.85773507037398	24.29946808080202
H	-0.35189457998906	6.95617640179174	23.08621123308797
C	-0.24910324803800	6.95592091888287	25.84181663676219
C	-0.19903650641050	4.71166511425814	24.70505829672105
C	0.63889660475733	6.75379507764276	23.49814612950829
C	0.49152967220845	6.06400607149816	24.85088864239806
H	-0.28381178313953	4.21578383818255	25.67353128112859
H	0.26648388372265	7.91018903289639	25.95951259652479
H	-0.31930955873683	6.47457176321756	26.81863273148548
H	1.18948287471754	6.11641777421235	22.80669423443301
H	1.18238968785149	7.69370834663291	23.60056672505304
H	0.36434465315255	4.06642167854607	24.02983114804769
N	1.80760241669273	5.83008385041038	25.35883339501186
C	2.90927156375956	5.65109515703111	25.68094715387722

^{t}BuNC

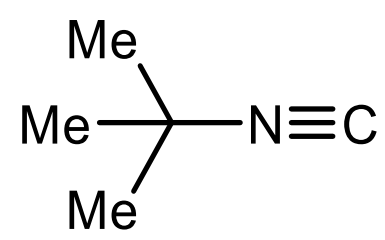

C	-6.89325096710719	1.51741994634276	-0.03910488182412
C	-5.37109163732317	1.53390123903493	-0.12380936814569
N	-7.41066656047967	1.89204966945737	-1.32503791754261
C	-7.39484219669739	2.53336890468760	0.98112895762729
C	-7.40787757692335	0.11903436933859	0.28296124830364
H	-5.00824427419106	2.53040983827494	-0.38274629698921
H	-5.01743286724583	0.82825701552288	-0.87791615118617
H	-4.94963292866193	1.25055306334989	0.84271755010967
H	-7.07019128538263	-0.59975266107684	-0.46604024614898
H	-8.49906229206540	0.10500122263682	0.31095583765641
H	-7.03083093950812	-0.19249073060046	1.25914020040063
H	-7.05044156055518	3.53791523265530	0.72835740661245
H	-7.01430538006015	2.27381255557417	1.97101896051244
H	-8.48581568201356	2.53878891534606	1.01812620429414
C	-7.82964385178532	2.19507141945596	-2.36523150367990

^{t}BuNCS

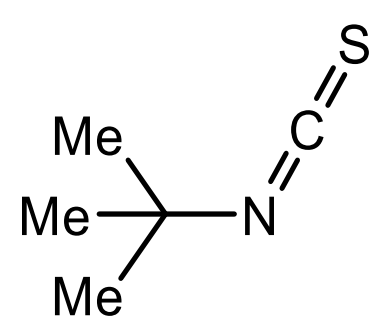

C	-6.92790075148340	1.53615345746564	-0.04775091700139
C	-5.40592532270933	1.55719629969405	-0.15257372970073
N	-7.46675129378981	1.93838804862655	-1.30969579245596
C	-7.41186712860123	2.52295754280068	1.01032929236086
C	-7.43138625809077	0.12805304024525	0.25396094936209
H	-5.04852192953195	2.56070794074247	-0.39184745126960
H	-5.06211772217445	0.87077251435366	-0.92884948198244
H	-4.96866840561165	1.25106552782439	0.80011771585173
H	-7.10471255225253	-0.57278870671856	-0.51683120172779
H	-8.52195328508190	0.10929121107061	0.30131350131161

H	-7.03650958783382	-0.20506383648300	1.21602963976766
H	-7.07290422388703	3.53462953404775	0.77890339869618
H	-7.01436752277820	2.23845144679661	1.98675727805423
H	-8.50213810067417	2.52561326769277	1.06675805795625
C	-7.84230223231551	2.26941669633505	-2.36661967369255
S	-8.38181503391747	2.70245714281098	-3.79989155763228

12 References

[1] N. Rahimi, B. de Bruin, P. H. M. Budzelaar, Balance between Metal and Ligand Reduction in Diiminepyridine Complexes of Ti, *Organometallics* **2017,** *17*, 3189–3198.

[2] S. Primpke, A. Deinert, F. Ehlers, P. Vana, A Kinetic Investigation of the Initialization of Catalyzed Chain Growth of Styrene: The Reaction of Cp* 2 ZrCl 2 with Dibenzylmagnesium, *Macromol. Chem. Phys.* **2014,** *6*, 544–554.

[3] P. Fritsche, C. Czernetzki, M. L. Heldner, L. Hörlin, I. Krummenacher, G. Hierlmeier, Controlling Reductive Elimination Pathways in Ti(IV) Pincer Complexes: Concerted versus Radical Meschanisms via Ligand Design, *J. Am. Chem. Soc.* **2026**, *148*, 4694–4705.

[4] W. Adam, R. M. Bargon, S. G. Bosio, W. D. Schenk, D. Stalke, Direct Synthesis of Isothiocyanates from Isonitriles by Molybdenum-Catalyzed Sulfur Transfer with Elemental Sulfur, *J. Org. Chem.* **2002**, *67*, 7037-7041.

[5] Z. Fu, W. Yuan, N. Chen, Z. Yang, J. Xu, $Na_2S_2O_8$-mediated efficient synthesis of isothiocyanates from primary amines in water, *Green Chem.* **2018**, *20*, 4484-4491.

[6] J. Ma, F. Li, C. Wang, Z. Wang, C. Du, L. Wang, Synthesis of Isothiocyanates from Primary Amines via Visible-Light Photocatalysis, *Org. Lett.* **2023**, *25*, 5692-5696.

[7] R. Nickisch, P. Conen, S. M. Gabrielsen, M. A. R. Meier, A more sustainable isothiocyanate synthesis by amine catalyzed sulfurization of isocyanides with elemental sulfur, *RSC Adv.* **2021**, *11*, 3134-3142.

[8] N. S. Habib, A. Rieker, Metathesis of Aryl Isothiocyanates: A Novel Method for the Synthesis of Sterically Hindered Aryl Isothiocyanates, *Synthesis* **1984**, *10*, 825-827.

[9] Y.-Y. Liao, J.-C. Deng, Y.-P. Ke, X.-L. Zhong, L. Xu, R.-Y. Tang, W. Zheng, Isothiocyanation of amines using the Langlois reagent, *Chem. Commun.* **2017**, *53*, 6073-6076.

[10] T. B. Nguyen, L. Ermolenko, A. Al-Mourabit, Three-Component Reaction between Isocyanides, Aliphatic Amines and Elemental Sulfur: Preparation of Thioureas under Mild Conditions with Complete Atom Economy, *Synthesis* **2014**, *46*, 3172-3179.

[11] G. M. Sheldrick, SABADS, Bruker AXS, Madison, USA, **2007**.

[12] CrysAlisPro. Scale3 Abspack, Rigaku Oxford Diffraction, **2019**.

[13] G. M. Sheldrick, SHELXT - Integrated space-group and crystal-structure determination, *Acta Cryst . A* **2015,** *71*, 3–8.

[14] G. M. Sheldrick, Crystal structure refinement with SHELXL, *Acta Cryst. C* **2015,** *71*, 3-8.

[15] G. M. Sheldrick, A short history of SHELX, *Acta Cryst. A* **2008,** *64*, 112–122.

[16] O. V. Dolomanov, L. J. Bourhis, R. J. Gildea, J. A. K. Howard, H. Puschmann, OLEX2: A complete structure solution, refinement and analysis program, *J. Appl. Crystallogr.* **2009,** *42*, 339–341.

[17] F. Neese The ORCA program system, WIREs Comput. *Mol. Sci.* **2012**, 2, 73–78.

[18] F. Neese, F. Wennmohs, U. Becker, C. Riplinger, The ORCA quantum chemistry program package, *J. Chem. Phys*. **2020**, *152*, 224108.

[19] F. Neese, Software Update: The ORCA Program System—Version 6.0, *WIREs Comput. Mol. Sci.* **2025**, *15*.

[20] E. Caldeweyher, C. Bannwarth, S. Grimme, Extension of the D3 dispersion coefficient model, *J. Chem. Phys.* **2017**, *147*, 34112.

[21] E. Caldeweyher, S. Ehlert, A. Hansen, H. Neugebauer, S. Spicher, C. Bannwarth, S. Grimme, A generally applicable atomic-charge dependent London dispersion correction, *J. Chem. Phys.* **2019**, *150*, 154122.

[22] A. Schäfer, C. Huber, R. Ahlrichs, Fully optimized contracted Gaussian basis sets of triple zeta valence quality for atoms Li to Kr, *J. Chem. Phys.* **1994**, *100*, 5829-5835.

[23] S. Kossmann, F. Neese, Comparison of two efficient approximate Hartee–Fock approaches, *Chem. Phys. Lett.* **2009**, *481*, 240-243.

[24] F. Neese, F. Wennmohs, A. Hansen, U. Becker, U. Efficient, approximate and parallel Hartree–Fock and hybrid DFT calculations. A 'chain-of-spheres' algorithm for the Hartree–Fock exchange, *Chem. Phys.* **2009**, *356*, 98-109.

[25] F. Neese, An improvement of the resolution of the identity approximation for the formation of the Coulomb matrix, *J. Comput. Chem*. **2003**, *24*, 1740-1747.

[26] G. A. Zhurko and D. A. Zhurko, "ChemCraft, Tool for Treatment of the Chemical Data." http://www.chemcraftprog.com.

[27] D. J. Liptrot, J.-D. Guo, S. Nagase, P. P. Power, Dispersion Forces, Disproportionation, and Stable High-Valent Late Transition Metal Alkyls, *Angew. Chem. Int. Ed.* **2016**, *55*, 14766–14769.
